# Identification of a novel lymphangiogenesis signature associated with immune cell infiltration in colorectal cancer based on bioinformatics analysis

**DOI:** 10.1186/s12920-023-01781-8

**Published:** 2024-01-02

**Authors:** Hong Liu, Huiwen Shi, Yinggang Sun

**Affiliations:** 1 Department of General Surgery, Wuxi Fifth People’s Hospital Affiliated to Jiangnan University, Wuxi, Jiangsu China; 2 Department of General Surgery, No.971 Hospital of PLA Navy, Qingdao, China; 3 Department of General Surgery, The 960th Hospital of Joint Logistics Support Force of Chinese People’s Liberation Army, Jinan, China

**Keywords:** Colorectal cancer, Lymphangiogenesis, Prognosis, Immune infiltration, WGCNA

## Abstract

**Background:**

Lymphangiogenesis plays an important role in tumor progression and is significantly associated with tumor immune infiltration. However, the role and mechanisms of lymphangiogenesis in colorectal cancer (CRC) are still unknown. Thus, the objective is to identify the lymphangiogenesis-related genes associated with immune infiltration and investigation of their prognosis value.

**Methods:**

mRNA expression profiles and corresponding clinical information of CRC samples were obtained from The Cancer Genome Atlas (TCGA) and Gene Expression Omnibus (GEO) databases. The lymphangiogenesis-related genes (LymRGs) were collected from the Molecular Signatures database (MSigDB). Lymphangiogenesis score (LymScore) and immune cell infiltrating levels were quantified using ssGSEA. LymScore) and immune cell infiltrating levels-related hub genes were identified using weighted gene co-expression network analysis (WGCNA). Univariate Cox and LASSO regression analyses were performed to identify the prognostic gene signature and construct a risk model. Furthermore, a predictive nomogram was constructed based on the independent risk factor generated from a multivariate Cox model.

**Results:**

A total of 1076 LymScore and immune cell infiltrating levels-related hub genes from three key modules were identified by WGCNA. Lymscore is positively associated with natural killer cells as well as regulator T cells infiltrating. These modular genes were enriched in extracellular matrix and structure, collagen fibril organization, cell-substrate adhesion, etc. NUMBL, TSPAN11, PHF21A, PDGFRA, ZNF385A, and RIMKLB were eventually identified as the prognostic gene signature in CRC. And patients were divided into high-risk and low-risk groups based on the median risk score, the patients in the high-risk group indicated poor survival and were predisposed to metastasis and advanced stages. NUMBL and PHF21A were upregulated but PDGFRA was downregulated in tumor samples compared with normal samples in the Human Protein Atlas (HPA) database.

**Conclusion:**

Our finding highlights the critical role of lymphangiogenesis in CRC progression and metastasis and provides a novel gene signature for CRC and novel therapeutic strategies for anti-lymphangiogenic therapies in CRC.

**Supplementary Information:**

The online version contains supplementary material available at 10.1186/s12920-023-01781-8.

## Introduction

Colorectal cancer (CRC), encompassing both rectal adenocarcinoma (READ) and colon adenocarcinoma (COAD), stands as the most prevalent cancer and the third leading cause of cancer-related deaths, accounting for over 1.9 million new cases and 935,000 deaths in 2020 [1]. It has been estimated that CRC will escalate to 3.2 million new cases and 1.6 billion deaths by 2040 [2]. Notably, CRC often serves as a marker of socioeconomic development, exhibiting increased incidence rates during periods of significant national transition [3, 4]. Several risk factors contribute to the prevalence of CRC, including reduced physical activity, excessive body weight, alcohol consumption, smoking, and the intake of red or processed meat [5, 6]. Over the past few decades, CRC has also emerged as an early-onset cancer, affecting individuals under the age of 50 [7]. Despite advancements in early screening methods and effective treatment strategies that have improved the prognosis for CRC patients, it continues to pose a serious threat to public health, constituting a substantial burden [8–10].

Previous studies have established that lymphangiogenesis is linked to lymphatic metastasis, distant metastasis, and adverse clinical outcomes in various types of tumors [11–13]. Moreover, the presence of functional lymphatic vessels plays a pivotal role in regulating the formation of inflammatory and immune microenvironments within tumors [14, 15]. The lymphatic system is essential for the collection and cycling of tissue-extravasated fluids, macromolecules, and immune cells into the bloodstream, especially mediates tumor metastasis and provides more chances for immunocytes to migrate through lymphatic vessels [16–18]. Lymphangiogenesis, extracellular matrix remodeling, and immunosuppressive cell enlisting in lymph nodes are indispensable for pre-metastatic niche formation [19]. Lymphangiogenesis occurs during the initial stage of metastasis in various types of malignant tumors, and recent evidence suggests that lymphatic vessels are involved in shaping antitumor immunity [20, 21]. Antigens, cytokines, and danger signals are drained from the tumor to sentinel lymph modes to promote T-cell infiltration [22]. Various tumor-associated immune cells play an important role in lymphangiogenesis, for example, tumor-associated macrophages (TAMs) and tumor-associated neutrophils (TANs) act as the important drivers of lymphangiogenesis [23–25]. Lymphangiogenesis is an intricate process governed by a multitude of factors and activated by numerous genes. Lymphangiogenesis-related genes (LRGs) have the potential to serve as prognostic indicators in human tumors. Lymph Node Metastasis Associated Transcript 1 (LNMAT1) promotes lymphatic metastasis and acts as a potential therapeutic target for LN-metastatic bladder cancer therapy [26]. Vascular endothelial growth factor C (VEGF-C), interleukin-4 (IL-4), colony-stimulating factor 2 (CSF2), prospero homeobox 1 (PROX1), and TEK receptor Tyrosine Kinase (TEK) is significantly associated with lymph node metastasis in renal cell carcinoma (RCC) [27, 28]. Collagen and calcium-binding EGF domain-1 (CCBE1) and neuropilin-2 (NRP2) promote lymphangiogenesis and lymphatic metastasis in CRC and can be used as therapeutic targets in CRC [29–31]. The above studies have confirmed that lymphangiogenesis is involved in tumor metastasis and poor clinical outcomes, targeting lymphangiogenesis emerges as a potential and effective therapeutic strategy. However, there are still numerous LRGs associated with the prognosis of CRC that remain unknown.

In the present study, a weight gene co-expression network analysis (WGCNA) was performed to identify the lymphangiogenesis and immune-related modules and screened the hub genes based on The Cancer Genome Atlas (TCGA) and Gene Expression Omnibus (GEO) databases. Then, the prognostic values of those hub genes were investigated using univariate Cox and Least Absolute Shrinkage and Selection Operator (LASSO) regression analyses. NUMBL, TSPAN11, PHF21A, PDGFRA, ZNF385A, and RIMKLB were selected as the lymphangiogenesis and immune-related signature that could be used for prognosis and prediction of therapeutic responses in CRC.

## Methods

### Data collection and processing

The gene expression data (fragments per kilobase per million mapped reads (FPKM) standardized data) and corresponding clinical information of 583 CRC patients from TCGA-COAD and TCGA-READ were downloaded from The Cancer Genome Atlas (TCGA, https://portal.gdc.cancer.gov/) database. Besides, the gene expression microarray data and corresponding clinical data of 232 CRC patients from the GSE17538 dataset were downloaded from the Gene Expression Omnibus (GEO, https://www.ncbi.nlm.nih.gov/geo/) database. The GSE17538 dataset was generated using the GLP570 platforms. Additionally, the 17 lymphangiogenesis-related genes (LymRGs) were collected from the Molecular Signatures Database (MSigDB, https://www.gsea-msigdb.org/gsea/msigdb/) and used for subsequent analyses (Table S1-2).

### Lymphangiogenesis score (LymScore) calculating and immune cell infiltration analysis

Single sample gene set enrichment analysis (ssGSEA) was performed to quantitatively calculate the LymScore of 17 LymRGs in each CRC sample using the GSVA package in R. Besides, the immune cell infiltration level in each CRC sample also was quantified using the GSVA R package based on the 28 immune genes sets (Table S3).

### Weighted gene co-expression network analysis (WGCNA) and identification of hub models and hub genes

WGCNA is a biology method used to illustrate the correlation patterns between gene modules and clinical traits [32]. Here, the WGCNA R package was utilized to construct a co-expression network and identify the hub genes that relate to LymScore and immune cell infiltration. The input genes for network construction were selected based on the median absolute deviation (MAD), and then the soft threshold power (β) was estimated using a nearly scale-free topology to construct a scale-free network. The topological overlap matrix (TOM) similarity was used to determine the distance between each gene pair. Hierarchical clustering analysis with the average method was used to establish the cluster tree, and then the variant set of genes was stratified into different modules with a minimum of 30 genes in each module. The first principal component of each module’s expression was summarized as a module eigengene (ME), and Pearson’s correlations between MEs and LymScore as well as immune cell infiltration were estimated to identify the significant modules with the greatest absolute module significance (MS) for further analyses. For each module, module membership (MM) was characterized with the correlation coefficient between ME and gene expression, and gene significance (GS) value was used to quantify the correlation between individual genes and clinical traits. Genes with MM > 0.6 and | GS | > 0.4 were identified as hub genes in the module.

### Gene ontology (GO) annotation and Kyoto encyclopedia of genes and genomes (KEGG) pathway enrichment analysis

The clusterProfiler R package was utilized to explore the biological functions of hub genes [33], which included Gene ontology (GO) and the Kyoto Encyclopedia of Genes and Genomes (KEGG). GO consists of three terms, including biological process (BP), molecular function (MF), and cellular component (CC). *P*-value < 0.05 was considered as the statistical significance.

### Construction and validation of a lym-risk score model

To assess the correlation between the hub genes and survival status of patients, 583 CRC patients from TCGA were divided into a training set and a test set on a 7:3 ratio, then, a univariate Cox analysis was performed using survminer R package to identify the survival-related genes. Genes with *P*-value < 0.05 were incorporated into a LASSO regression model using the glmnet R package to shrink the number of genes. Then, the Lym-risk score was calculated as follows, Lym-risk score = (-0.41902565) * TSPAN11 expression + (-0.27690515) * PDGFRA expression + 0.03754875 * ZNF386A expression + 0.3706381 * NUMBL expression + 0.4806545 * PHF21A expression + 0.48551082 * RIMKLB expression. Then, the patients from the training set, test set, and external validation set were classified into high- and low-Lym-risk groups based on the median Lym-risk score. The overall survival (OS) of each patient between high- and low-Lym-risk groups was determined using Kaplan-Meier curves and the log-rank test.

### Construction of a predictive nomogram

The clinical characteristics (age, gender, T/N/M stages, grade) and risk score were incorporated into a multivariate Cox model to identify the independent factors in CRC. Then, the rms R package was performed to construct a predictive nomogram. The accuracy of the nomogram was detected using the calibration curve.

### Validation of Lym-related signature via human protein atlas (HPA) databases

The protein levels of the Lym-related signature were investigated based on the immunohistochemical (IHC) staining images in CRC tissues that were downloaded from the HPA online database (https://www.proteinatlas.org/).

### Statistical analysis

Statistical analyses were performed by using R (version 4.0.2) software packages, and *P* < 0.05 was considered statistically significant.

## Results

### Construction of a co-expression network of Lymscore and immune cell infiltration in CRC

The LymScore and the immune cell infiltration level in each CRC sample were quantified (Table S2-3). Then, we constructed a co-expression network to identify the significant modules that were associated with the LymScore and immune cell infiltration in CRC. Firstly, we evaluate the global gene expression patterns using hierarchical clustering to eliminate the outlier samples that would impact the subsequent analyses. Here, no outlier samples were removed according to the sample clustering results (Figure S1A). Then, a trait heatmap was constructed that showed the distribution of samples according to the corresponding clinical features (Figure S1B). Afterward, seventeen modules were generated from the TCGA cohort based on the differential expression of genes (Figure S2A). The optimal β = 10 was considered the soft threshold ensuring that the network was scale-free (scale-free R^2^ = 0.85, Figure S2B). The adjacency matrix transformed to the topological overlap matrix (TOM) and 17 modules were ensured using a cutoff of 0.25 and a minimum module size of 100 (Figure S2C-1D).

**Fig. 1:**
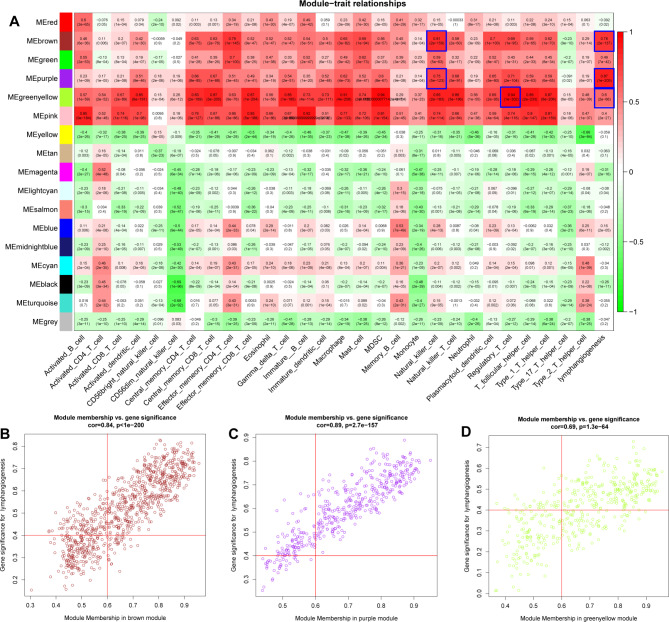
Identification of LymScore and immune cell infiltration-related hub genes in CRC based on WGCNA. **A**. A heatmap indicating the correlation between modules and LymScore and immune cell infiltration levels. **B-D**. Scatter plot indicating the memberships and the gene significance for lymphangiogenesis in brown, purple, and green-yellow modules

### Identification of LymScore and immune cell infiltration-related hub genes in CRC

Based on the co-expression network of Lymscore and immune cell infiltration, a heatmap was used to describe the relationship between modules and LymScore and immune cell infiltration (Fig. 1A). As a result, brown (Nature killer cells, r = 0.81, *p* = 2e-159; LymScore, r = 0.78, *p* = 2e-137), purple (Nature killer cells, r = 0.75, *p* = 5e-123; LymScore, r = 0.87, *p* = 1e-205), and green-yellow (Regulator T cells, r = 0.94, *p* = 1e-302; LymScore, r = 0.60, *p* = 2e-66) modules were significantly associated with LymScore and immune cell infiltration in CRC with the *p*-value < 0.05 and |r| >0.5 (Table S4). A total of 1076 hub genes with MM > 0.6 and | GS | > 0.4 were identified as hub genes from three modules (Fig. 1B-D, Table S4).

### Functional analysis of hub genes

**Fig. 2:**
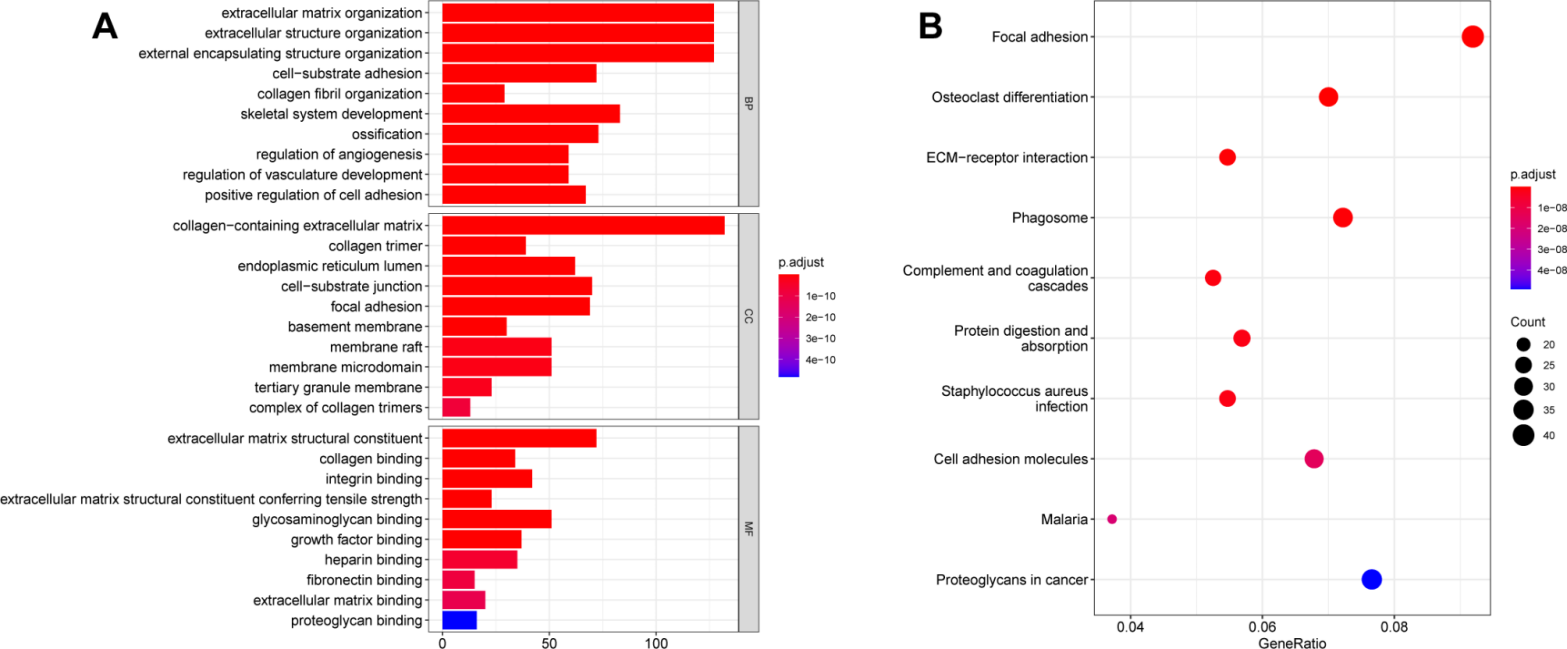
Functional analysis of hub genes. **A**. GO annotation analysis of hub genes from brown, purple, and green-yellow modules. GO annotation comprises biological process (BP), cellular component (CC), and molecular function (MF) terms. **B**. KEGG enrichment analysis of hub genes from brown, purple, and green-yellow modules

We further conducted the GO and KEGG enrichment analyses to investigate the potential mechanisms of the above modules. A total of 1,217 GO terms and 43 KEGG pathways were enriched (Table S5). As shown in Fig. 2A, the top 10 BP terms included extracellular matrix and structure, external encapsulating structure, collagen fibril organization, cell-substrate adhesion, skeletal system development, ossification, regulation of angiogenesis and vasculature development, and positive regulation of cell adhesion. For CC analysis, such as the collagen-containing extracellular matrix, collagen trimer, endoplasmic reticulum lumen cell-substrate junction, and focal adhesion were enriched. For MF analysis, several MF were identified, such as extracellular matrix structural constituent, collagen binding, integrin binding, and extracellular matrix structural constituent conferring tensile strength. KEGG pathway enrichment analysis revealed that several pathways were enriched in CRC, including focal adhesion, ECM-receptor interaction, phagosome, complement, and coagulation cascades, protein digestion and absorption, and cell adhesion molecules (Fig. 2B).

### Construction of the LymScore and immune cell infiltration-related prognostic signature

**Fig. 3:**
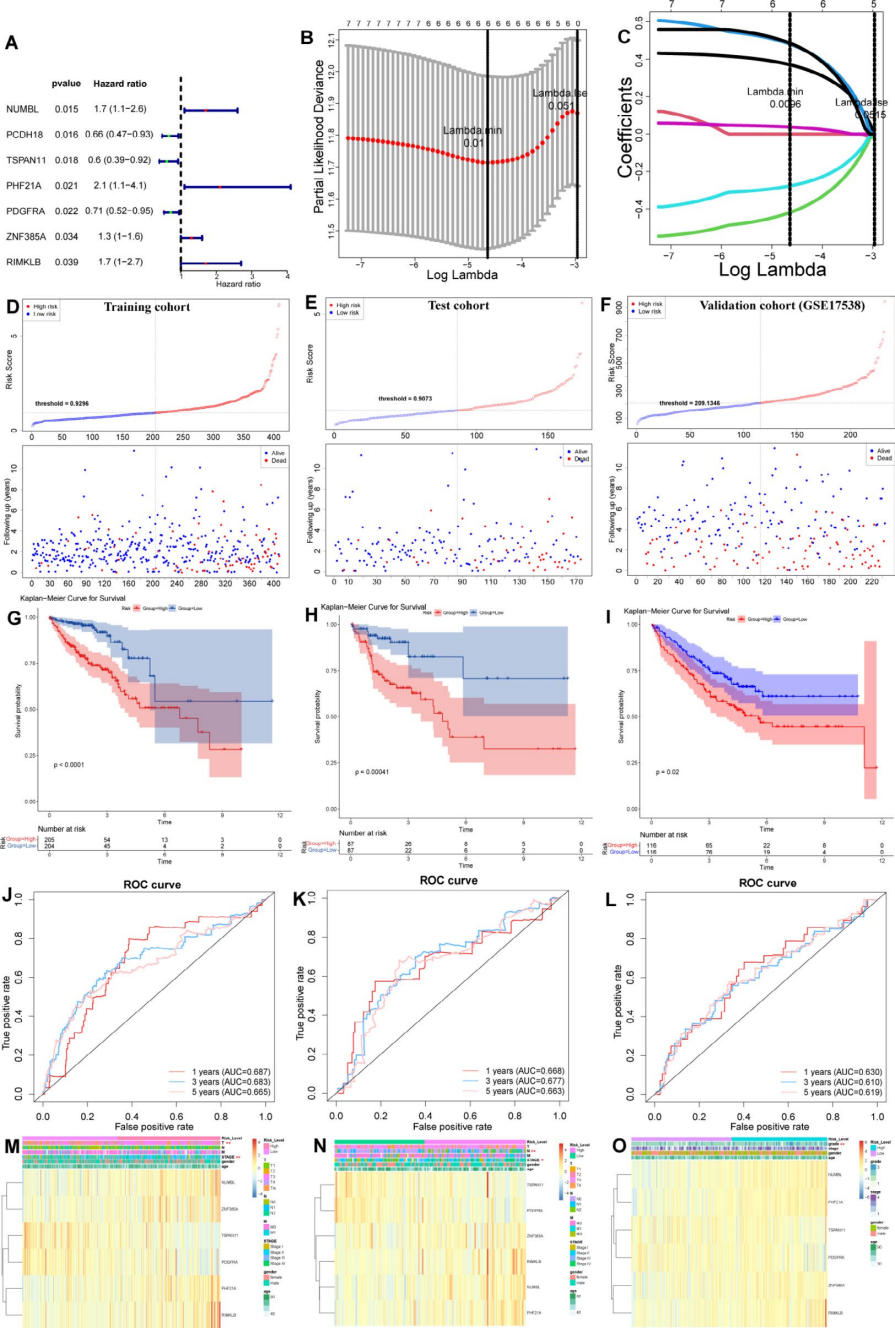
Construction of the LymScore and immune cell infiltration-related prognostic signature. **A**. Forest plot showing the univariate Cox analysis for identifying survival-associated genes. **B**. LASSO coefficient profiles of the seven survival-associated genes in the TCGA cohort. **C**. Selection of the optimal parameter (lambda) in the LASSO model. **D**-**F**. CRC patients of training, test, and external validation cohorts were distributed into high-risk and low-risk groups based on risk score, and scatter plots showing the survival status. **G**-**I**. Kaplan-Meier analysis for OS of high-risk and low-risk groups both in three cohorts. **M**-**O**. Heatmap showing the correlation between clinical characteristics (age, gender, **T**/**N**/M stages) and risk score both in three cohorts

We also investigated the prognostic values of above 1076 hub genes by conducting a univariate Cox model. The 583 CRC patients from TCGA were distributed into a training set (n = 409) and test set (n = 174) with 7:3. And the univariate Cox results indicated that NUMBL, PCDH18, TSPAN11, PHF21A, PDGFRA, ZNF385A, and RIMKLB were closely related to prognosis (Fig. 3A, Table S6). Those genes were incorporated into a LASSO regression model to select the prognostic signature for CRC, resulting in six genes (NUMBL, TSPAN11, PHF21A, PDGFRA, ZNF385A, and RIMKLB) were finally identified for the LymScore and immune cell infiltration-related risk model construction (Fig. 3B-C). CRC patients in each cohort were distributed into high- and low-risk groups with the median value of risk score (Fig. 3D-F), Kaplan-Meier curves indicated that CRC patients in the high-risk score group showed poor survival compared to those in the low-risk score group (Fig. 3G-I). The AUC values of ROC curves for 1-, 3-, and 5-year OS prediction were more than 0.6 in both three cohorts (Fig. 3J-L). Correlation analysis between the risk score and clinical characteristics (age, gender, T/N/M stages) revealed that the high-risk score is closely related to tumor metastasis and advanced stage (Fig. 3M-O, Table S7).

### Validation of the hub gene expression based on the HPA database

**Fig. 4:**
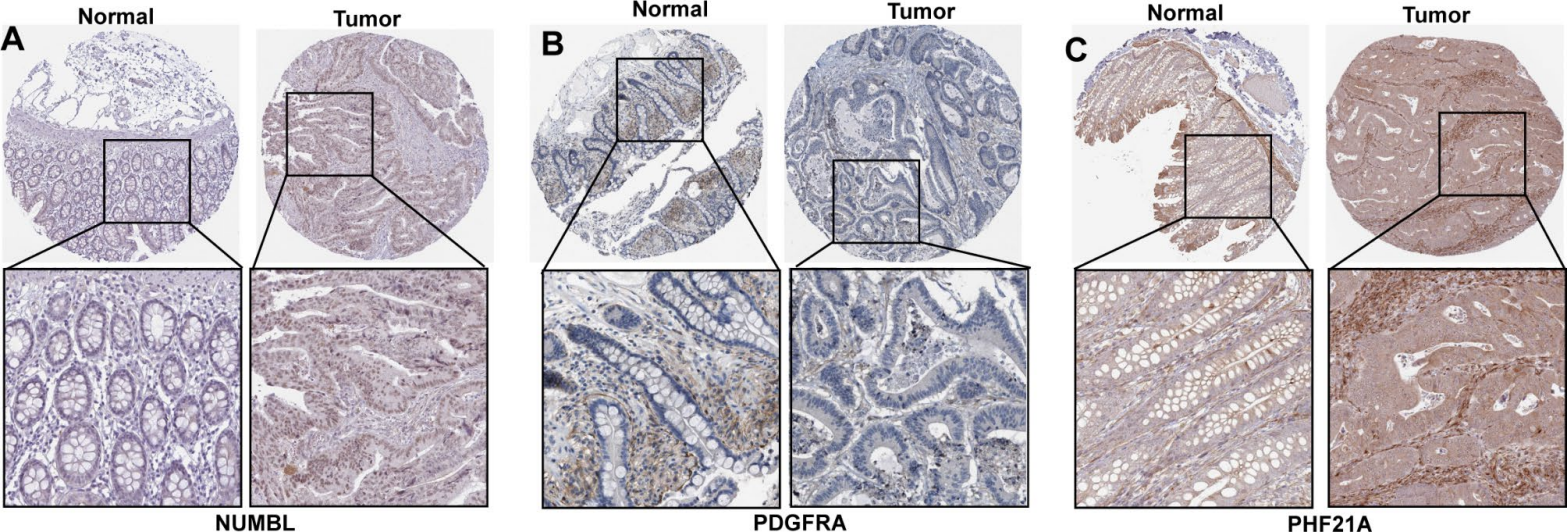
Validation of the hub gene expression based on the HPA database. **A-C**. protein levels of NUMBL, PDGFRA, and PHF21A in CRC tumor specimens from the Human Protein Atlas database

To validate the protein expression levels of prognostic hub genes, the IHC images of the tumor and normal tissues, indicated that NUMBL and PHF21A were strongly expressed in tumor tissues compared with normal tissues (Fig. 4A and C), but PDGFRA was deeply stained in normal tissues compared with tumor tissues (Fig. 4B). The results demonstrated that NUMBL and PHF21A might act as risk factors, while PDGFRA might function as the protective factor.

### Development of a predictive nomogram

**Fig. 5:**
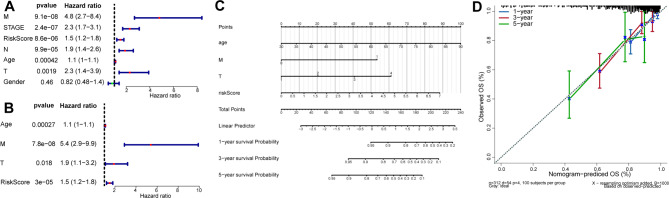
Development of a predictive nomogram. **A-B**. Forest plots showing the univariate and multivariate Cox analyses for identifying independent risk factors. **C**. A nomogram for predicting 1-, 3-, and 5-year OS probability in CRC. **D**. The calibration curve shows the performance of the nomogram for predicting 1-, 3-, and 5-year OS

Finally, we also constructed a nomogram to predict 1-, 3-, and 5-year OS probability in the training set by including age, gender, and T/N/M stages that were selected as the independent risk factor in CRC based on univariate and multivariate Cox analyses (Fig. 5A-C). The calibration curve indicated the good performance of the nomogram for predicting 1-, 3-, and 5-year OS (Fig. 5D).

## Discussion

CRC is one of the most common malignant tumors worldwide among men and women, with the aging population, dietary habits, obesity, lack of physical exercise, and smoking, CRC has been frequently diagnosed, in China with increased incidence and mortality in recent years [34, 35]. Despite the inspiring improvements in diagnosis and therapy of CRC so far, the survival rate in patients with CRC remains low, the overall survival for advanced CRC without metastasis to 3 years [8], and metastatic CRC exhibits approximately 70–75% of patients survive beyond 1 year, 30–35% beyond 3 years, and fewer than 20% beyond 5 years from diagnosis [36]. We need to identify novel biomarkers and mechanisms for the development and optimization of CRC preventive strategies.

Lymphangiogenesis is an important event during the metastasis in CRC [37], and anti-lymphangiogenic therapies become novel therapeutic strategies [38]. However, the role of lymphangiogenesis contributes to the progression of CRC remains unclear. It has been found that the interaction between the tumor and lymphatic system affects tumor immunity, such as tumor microenvironment and the immune response, and then regulates tumor metastasis [39, 40]. Nevertheless, the biomarkers that are associated with the interaction between the lymphatic system and tumor immune microenvironment are still unknown. In the present study, we construct a WGCNA to identify three modules associated with LymScore and the immune cell infiltration level, then 1076 hub genes from the modules. The biological function analysis has indicated that those genes were associated with extracellular matrix and structure, cell-substrate adhesion, regulation of angiogenesis and vasculature development, positive regulation of cell adhesion, and so on. The extracellular matrix proteins, such as polydom and heparinase, were involved in lymphatic vessel remodeling or lymphangiogenesis [41, 42].

Then, with the survival analysis, the prognostic valuable biomarkers (NUMBL, PCDH18, TSPAN11, PHF21A, PDGFRA, ZNF385A, and RIMKLB) were selected and then NUMBL, TSPAN11, PHF21A, PDGFRA, ZNF385A, and RIMKLB were identified as the LymScore and immune cell infiltration-related gene signature. We also demonstrated that high expression of NUMBL and PHF21A, but low expression of PDGFRA in tumor tissues compared with normal tissues. NUMB-like (NUMBL) is a docking protein with a PTB domain that serves as a Notch signaling inhibitor, the aberrant expression of NUMBL can promote carcinogenesis by the dysregulation of the WNT-Notch signaling cycle [43]. Besides, NUMBL plays a crucial role in various processes, such as targeting proteins for ubiquitination and endocytosis, asymmetric cell division, cell migration, and cell adhesion [44–46]. TSPAN11 is one number of the Tetraspanins (TSPANs), which are a class of four transmembrane segmented proteins, TSPANs play dual roles in pan-cancer [47]. A previous study has demonstrated that TSPAN11 was downregulated in pan-cancer and positively associated with CD4 + T cells, macrophages, neutrophils, and dendritic cells [48]. PHD finger protein 21 A (PHF21A), also known as BHC80, is a molecule that recognizes unmethylated H3K4 and plays an important role in neuronal and craniofacial development [49, 50]. It has been found that PHF21A serves as a biomarker for the progression and prognosis of hepatocellular carcinoma (HCC) [51]. PDGFRA is a classical proto-oncogene that encodes receptor tyrosine kinases responding to platelet-derived growth factor (PDGF) [52]. Previous study has revealed that high frequency of PDGFRA mutations in high-grade gliomas [53], and overexpression of PDGFRA in oral cancer patients [54]. Moreover, PDGFRA serves as a therapeutic target in young CRC patients [55]. Zinc finger protein 385 A (ZNF385A) is an RNA-binding Cys2 His2 (C2H2) zinc finger protein that is involved in the regulation of cell cycle and apoptosis and acts as a prognostic biomarkers that are associated with immunosuppression in HCC [56]. RimK-like family member B (RIMKLB) is an enzyme that post-translationally modulates ribosomal protein S6 and contributes to the regulation of immune cell development [57]. RIMKLB has been identified as a prognostic biomarker in CRC [58–61]. Our results were consistent with previous findings. Furthermore, based on the LymScore and immune cell infiltration-related gene signature, patients were divided into high-risk and low-risk groups. The patients with high-risk scores showed poor survival, tumor metastasis, and more severe stages compared to those with low-risk scores. We also constructed and validated a predictive nomogram for predicting 1-, 3-, and 5-year OS probability in CRC. Our finding confirmed that lymphangiogenesis is significantly associated with poor prognosis [62, 63].

Despite our findings elucidating the crucial role of lymphangiogenesis in CRC and identifying the prognostic value of LymScore and immune cell infiltration-related genes, there remain some limitations. Firstly, our analyses were performed based on TCGA and GEO online databases, more explicit clinical data should be collected for validating the expression levels of risk signatures and their prognostic values in the following study. Secondly, our study does not include experimental evidence to demonstrate the expression levels and regulatory mechanisms of risk signatures, we further explore the expression levels and regulatory mechanisms of risk signatures in vitro and in vivo in the following study.

## Conclusion

Our finding provides lymphangiogenesis and immune cell infiltration level-related biomarkers for predicting the prognosis of CRC patients and supplies novel therapeutic strategies for anti-lymphangiogenic therapies in CRC.

### Electronic supplementary material

Below is the link to the electronic supplementary material.

**Supplementary Material 1: Table S1** Lymphangiogenesis-related genes. **Table S2** lymphangiogenesis score. **Table S3** ssGSEA of immune cell infiltration. **Table S4** Hub modules. **Table S5** Biological functions. **Table S6** Univariate Cox analysis. **Table S7** Correlation risk score and clinical characteristics

**Supplementary Material 2: Figure S1**. Construction of a WGCNA. A. Sample clustering of genes from the TCGA database to identify outliers. B. Clustering dendrogram of CRC samples and associated clinical traits that included Lymphangiogenesis score (LymScore) and immune cell infiltration levels

**Supplementary Material 3: Figure S2**. Construction of a co-expression network of Lymscore and immune cell infiltration. A. Clustering dendrograms of genes based on the topological overlap and together with assigned module colors. B. The scale-free fit index for soft-thresholding powers. C. Clustering dendrograms of module eigengenes based on the topological overlap. D. Hierarchical clustering dendrograms of genes based on optimal soft-thresholding power

## Data Availability

The datasets used in this study are available from the public databases and can be found here: TCGA (https://portal.gdc.cancer.gov/), GEO (https://www.ncbi.nlm.nih.gov/geo/), and MSigDB, (https://www.gsea-msigdb.org/gsea/msigdb/). The supplementary files are stored at the Git-hub (https://github.com/DrSun2023/Supplementary-Tables.git).
