# Supplementary material for: Identification of a novel lymphangiogenesis signature associated with immune cell infiltration in colorectal cancer based on bioinformatics analysis
Source: BMC Med Genomics. 2024 Jan 2;17:2. doi: 10.1186/s12920-023-01781-8 (PMC10763205; doi:10.1186/s12920-023-01781-8)
Supplement: Supplementary file 1 — Supplementary Material 1: Table S1 Lymphangiogenesis-related genes. Table S2 lymphangiogenesis score. Table S3 ssGSEA of immune cell infiltration. Table S4 Hub modules. Table S5 Biological functions. Table S6 Univariate Cox analysis. Table S7 Correlation risk score and clinical characteristics [file 12920_2023_1781_MOESM1_ESM.docx]

Table S1 Lymphangiogenesis-related genes.

| gene | lymphangiogenesis |
| --- | --- |
| PDPN | lymphangiogenesis |
| CCBE1 | lymphangiogenesis |
| CLEC14A | lymphangiogenesis |
| EPHA2 | lymphangiogenesis |
| VASH1 | lymphangiogenesis |
| FOXC1 | lymphangiogenesis |
| FOXC2 | lymphangiogenesis |
| FLT4 | lymphangiogenesis |
| SOX18 | lymphangiogenesis |
| PPP3CB | lymphangiogenesis |
| PROX1 | lymphangiogenesis |
| PTPN14 | lymphangiogenesis |
| BMPR2 | lymphangiogenesis |
| TIE1 | lymphangiogenesis |
| VEGFC | lymphangiogenesis |
| ACVR2B | lymphangiogenesis |
| ACVRL1 | lymphangiogenesis |

Table S2 lymphangiogenesis score

| ID | Lymphangiogenesis |
| --- | --- |
| TCGA-AH-6643-11A-01R-1830-07 | 0.904396 |
| TCGA-AA-3655-11A-01R-1723-07 | 0.735839 |
| TCGA-AA-3489-11A-01R-1839-07 | 0.865688 |
| TCGA-A6-2685-11A-01R-A32Z-07 | 0.818178 |
| TCGA-A6-2683-11A-01R-A32Z-07 | 0.827373 |
| TCGA-AA-3531-11A-01R-A32Z-07 | 0.894838 |
| TCGA-AA-3660-11A-01R-1723-07 | 0.738484 |
| TCGA-AA-3663-11A-01R-1723-07 | 0.930229 |
| TCGA-A6-2675-11A-01R-1723-07 | 1.089266 |
| TCGA-AA-3525-11A-01R-A32Z-07 | 0.69344 |
| TCGA-AA-3496-11A-01R-1839-07 | 1.056267 |
| TCGA-AG-3731-11A-01R-1736-07 | 0.878648 |
| TCGA-AG-3725-11A-01R-1736-07 | 0.673531 |
| TCGA-AG-3742-11A-01R-1660-07 | 0.917117 |
| TCGA-AF-2691-11A-01R-A32Z-07 | 0.898044 |
| TCGA-AZ-6598-11A-01R-1774-07 | 0.954648 |
| TCGA-A6-2678-11A-01R-A32Z-07 | 0.886876 |
| TCGA-A6-2679-11A-01R-A32Z-07 | 0.885366 |
| TCGA-AA-3527-11A-01R-A32Z-07 | 0.86345 |
| TCGA-AZ-6600-11A-01R-1774-07 | 1.030999 |
| TCGA-AA-3517-11A-01R-A32Z-07 | 0.934679 |
| TCGA-A6-5659-11A-01R-1653-07 | 0.724386 |
| TCGA-AZ-6605-11A-01R-1839-07 | 1.206643 |
| TCGA-A6-2680-11A-01R-A32Z-07 | 0.821356 |
| TCGA-AA-3516-11A-01R-A32Z-07 | 0.790688 |
| TCGA-A6-5667-11A-01R-1723-07 | 0.871745 |
| TCGA-AZ-6603-11A-02R-1839-07 | 1.12385 |
| TCGA-AA-3713-11A-01R-1723-07 | 0.719047 |
| TCGA-AF-5654-11A-11R-1660-07 | 0.862597 |
| TCGA-AF-2689-11A-01R-A32Z-07 | 1.038238 |
| TCGA-A6-5662-11A-01R-1653-07 | 1.033718 |
| TCGA-AA-3511-11A-01R-1839-07 | 0.782451 |
| TCGA-AA-3662-11A-01R-1723-07 | 0.819844 |
| TCGA-AA-3520-11A-01R-A32Z-07 | 0.843014 |
| TCGA-A6-2682-11A-01R-A32Z-07 | 0.786145 |
| TCGA-AA-3522-11A-01R-A32Z-07 | 0.765595 |
| TCGA-AZ-6601-11A-01R-1774-07 | 1.01583 |
| TCGA-A6-5665-11A-01R-1653-07 | 0.804696 |
| TCGA-AA-3514-11A-01R-A32Z-07 | 0.734065 |
| TCGA-AF-2692-11A-01R-A32Z-07 | 1.024926 |
| TCGA-AG-3732-11A-01R-1660-07 | 1.030212 |
| TCGA-AA-3534-11A-01R-A32Z-07 | 0.796473 |
| TCGA-AF-3400-11A-01R-A32Z-07 | 0.763789 |
| TCGA-A6-2684-11A-01R-A32Z-07 | 0.728084 |
| TCGA-A6-2686-11A-01R-A32Z-07 | 0.860868 |
| TCGA-F4-6704-11A-01R-1839-07 | 0.927994 |
| TCGA-AA-3518-11A-01R-1672-07 | 0.936711 |
| TCGA-A6-2671-11A-01R-A32Z-07 | 0.98217 |
| TCGA-AZ-6599-11A-01R-1774-07 | 1.076748 |
| TCGA-AA-3712-11A-01R-1723-07 | 0.761058 |
| TCGA-AA-3697-11A-01R-1723-07 | 0.972674 |
| TCGA-DC-4745-01A-01R-A32Z-07 | 0.833631 |
| TCGA-DY-A1DC-01A-31R-A155-07 | 0.868817 |
| TCGA-AG-3732-01A-11R-1660-07 | 0.792395 |
| TCGA-AF-3911-01A-01R-1736-07 | 0.878248 |
| TCGA-AH-6549-01A-11R-1830-07 | 0.950997 |
| TCGA-AG-3887-01A-01R-1119-07 | 0.846113 |
| TCGA-DY-A1DD-01A-21R-A155-07 | 0.852955 |
| TCGA-EI-6881-01A-11R-A32Z-07 | 0.848135 |
| TCGA-AF-5654-01A-01R-1660-07 | 0.785885 |
| TCGA-AH-6544-01A-11R-1830-07 | 0.646061 |
| TCGA-DY-A1H8-01A-21R-A155-07 | 0.635711 |
| TCGA-EI-6514-01A-11R-1736-07 | 0.948812 |
| TCGA-AG-4001-01A-02R-1119-07 | 1.083851 |
| TCGA-EI-6917-01A-11R-1928-07 | 0.928475 |
| TCGA-AG-3608-01A-01R-0826-07 | 0.778918 |
| TCGA-DC-6160-01A-11R-1660-07 | 0.770862 |
| TCGA-EI-7002-01A-11R-1928-07 | 0.857529 |
| TCGA-AG-3999-01A-01R-1119-07 | 0.990294 |
| TCGA-AG-A002-01A-01R-A002-07 | 0.935653 |
| TCGA-BM-6198-01A-11R-1736-07 | 0.894975 |
| TCGA-F5-6864-01A-11R-1928-07 | 1.00733 |
| TCGA-AG-3583-01A-01R-0821-07 | 0.714904 |
| TCGA-CK-4950-01A-01R-1723-07 | 0.821845 |
| TCGA-G4-6294-01A-11R-1774-07 | 0.705372 |
| TCGA-A6-3807-01A-01R-1022-07 | 0.914336 |
| TCGA-AA-3846-01A-01R-1022-07 | 0.748368 |
| TCGA-F4-6703-01A-11R-1839-07 | 1.29292 |
| TCGA-AZ-4313-01A-01R-1410-07 | 0.796426 |
| TCGA-F4-6461-01A-11R-1774-07 | 1.117158 |
| TCGA-AA-3811-01A-01R-1022-07 | 1.007992 |
| TCGA-G4-6303-01A-11R-1774-07 | 1.00414 |
| TCGA-QG-A5Z2-01A-11R-A28H-07 | 0.50693 |
| TCGA-CA-5256-01A-01R-1410-07 | 0.697176 |
| TCGA-G4-6317-01A-11R-1723-07 | 0.61473 |
| TCGA-AA-3488-01A-01R-1410-07 | 0.761386 |
| TCGA-CM-5341-01A-01R-1410-07 | 0.803176 |
| TCGA-F4-6854-01A-11R-1928-07 | 0.948886 |
| TCGA-AA-3947-01A-01R-1022-07 | 0.773403 |
| TCGA-CM-6162-01A-11R-1653-07 | 1.154299 |
| TCGA-AA-3662-01A-01R-1723-07 | 0.864589 |
| TCGA-A6-2678-01A-01R-0821-07 | 0.852825 |
| TCGA-CM-6675-01A-11R-1839-07 | 0.620069 |
| TCGA-AY-4071-01A-01R-1113-07 | 0.859258 |
| TCGA-AA-3976-01A-01R-1022-07 | 0.862501 |
| TCGA-CA-5797-01A-01R-1653-07 | 0.877853 |
| TCGA-AA-A01R-01A-21R-A083-07 | 0.744618 |
| TCGA-A6-5664-01A-21R-1839-07 | 1.10366 |
| TCGA-AA-A02H-01A-01R-A089-07 | 0.932983 |
| TCGA-AA-3862-01A-01R-1022-07 | 0.822338 |
| TCGA-D5-7000-01A-11R-A32Z-07 | 0.923516 |
| TCGA-CA-6716-01A-11R-1839-07 | 0.914347 |
| TCGA-AA-A01G-01A-01R-A002-07 | 0.754439 |
| TCGA-D5-6535-01A-11R-1723-07 | 0.887209 |
| TCGA-CK-6748-01A-11R-1839-07 | 1.118143 |
| TCGA-D5-5539-01A-01R-1653-07 | 0.942413 |
| TCGA-A6-4105-01A-02R-1774-07 | 0.824659 |
| TCGA-G4-6307-01A-11R-1723-07 | 0.569827 |
| TCGA-EI-6512-01A-11R-1736-07 | 0.854283 |
| TCGA-AH-6897-01A-11R-1928-07 | 0.657233 |
| TCGA-F5-6813-01A-11R-1830-07 | 0.960821 |
| TCGA-AG-3591-01A-01R-1736-07 | 0.749735 |
| TCGA-AG-3605-01A-01R-0826-07 | 0.683681 |
| TCGA-A6-6137-01A-11R-1774-07 | 0.81489 |
| TCGA-AA-3860-01A-02R-0905-07 | 0.873973 |
| TCGA-AA-3986-01A-02R-1022-07 | 0.818833 |
| TCGA-CM-6165-01A-11R-1653-07 | 0.948171 |
| TCGA-CM-4750-01A-01R-1410-07 | 0.736704 |
| TCGA-AA-3520-01A-01R-0821-07 | 0.956117 |
| TCGA-G4-6625-01A-21R-1774-07 | 0.937617 |
| TCGA-DM-A28M-01A-12R-A16W-07 | 0.686241 |
| TCGA-D5-6920-01A-11R-1928-07 | 0.791672 |
| TCGA-DM-A288-01A-11R-A16W-07 | 0.760906 |
| TCGA-AA-A02O-01A-21R-A16W-07 | 0.793594 |
| TCGA-AA-A02E-01A-01R-A00A-07 | 0.865954 |
| TCGA-AA-3710-01A-01R-1022-07 | 0.782083 |
| TCGA-AZ-4614-01A-01R-1410-07 | 0.630001 |
| TCGA-G4-6320-01A-11R-1723-07 | 0.660708 |
| TCGA-AA-3856-01A-01R-0905-07 | 0.760912 |
| TCGA-A6-6138-01A-11R-1774-07 | 1.03556 |
| TCGA-A6-2685-01A-01R-1410-07 | 1.136625 |
| TCGA-QG-A5YX-01A-11R-A28H-07 | 0.556448 |
| TCGA-DM-A1D8-01A-11R-A155-07 | 0.667466 |
| TCGA-AM-5821-01A-01R-1653-07 | 0.895798 |
| TCGA-CK-5916-01A-11R-1653-07 | 0.842462 |
| TCGA-NH-A50T-01A-11R-A28H-07 | 0.602411 |
| TCGA-WS-AB45-01A-11R-A41B-07 | 1.495926 |
| TCGA-AA-A00Z-01A-01R-A002-07 | 0.760195 |
| TCGA-CM-6674-01A-11R-1839-07 | 0.953783 |
| TCGA-AA-3833-01A-01R-0905-07 | 0.919296 |
| TCGA-A6-2680-01A-01R-1410-07 | 0.904802 |
| TCGA-CM-5862-01A-01R-1653-07 | 0.992121 |
| TCGA-AA-3555-01A-01R-0821-07 | 0.902448 |
| TCGA-DM-A28G-01A-11R-A16W-07 | 0.68363 |
| TCGA-5M-AATE-01A-11R-A41B-07 | 0.697261 |
| TCGA-DM-A28H-01A-11R-A16W-07 | 0.640923 |
| TCGA-AA-A024-01A-02R-A00A-07 | 0.718775 |
| TCGA-CM-4747-01A-01R-1410-07 | 0.911168 |
| TCGA-G4-6314-01A-11R-1723-07 | 1.112466 |
| TCGA-AA-3930-01A-01R-1022-07 | 0.894878 |
| TCGA-CA-6718-01A-11R-1839-07 | 1.031241 |
| TCGA-AD-6963-01A-11R-1928-07 | 0.756765 |
| TCGA-AA-3982-01A-02R-1022-07 | 0.768179 |
| TCGA-5M-AATA-01A-31R-A41B-07 | 1.023937 |
| TCGA-CM-6680-01A-11R-1839-07 | 0.911376 |
| TCGA-DM-A1D6-01A-21R-A155-07 | 0.614374 |
| TCGA-AZ-6603-01A-11R-1839-07 | 0.947875 |
| TCGA-A6-6142-01A-11R-1774-07 | 1.156891 |
| TCGA-AZ-4315-01A-01R-1410-07 | 0.68594 |
| TCGA-AD-6895-01A-11R-1928-07 | 0.937428 |
| TCGA-CA-5796-01A-01R-1653-07 | 0.778819 |
| TCGA-AA-3510-01A-01R-1410-07 | 0.796182 |
| TCGA-AA-3527-01A-01R-0821-07 | 0.878123 |
| TCGA-F4-6807-01A-11R-1839-07 | 1.076917 |
| TCGA-G4-6310-01A-11R-1723-07 | 0.951506 |
| TCGA-AA-A00K-01A-02R-A002-07 | 0.879704 |
| TCGA-D5-6529-01A-11R-1774-07 | 0.989898 |
| TCGA-AA-A01I-01A-02R-A089-07 | 0.785875 |
| TCGA-AA-A01Z-01A-11R-A083-07 | 0.811938 |
| TCGA-G4-6588-01A-11R-1774-07 | 0.724509 |
| TCGA-CK-5912-01A-11R-1653-07 | 0.918591 |
| TCGA-CM-6679-01A-11R-1839-07 | 1.026953 |
| TCGA-AA-3519-01A-02R-0821-07 | 0.81282 |
| TCGA-AZ-6607-01A-11R-1839-07 | 1.188382 |
| TCGA-CM-5344-01A-21R-1723-07 | 0.908371 |
| TCGA-AA-3831-01A-01R-0905-07 | 0.833218 |
| TCGA-A6-2672-01A-01R-0826-07 | 0.880338 |
| TCGA-DM-A0X9-01A-11R-A155-07 | 0.757873 |
| TCGA-D5-6541-01A-11R-1723-07 | 0.957289 |
| TCGA-A6-4107-01A-02R-1410-07 | 0.845948 |
| TCGA-AA-3875-01A-01R-0905-07 | 0.729421 |
| TCGA-G4-6295-01A-11R-1723-07 | 0.749834 |
| TCGA-DM-A28F-01A-11R-A32Y-07 | 0.740902 |
| TCGA-CM-4743-01A-01R-1723-07 | 0.853211 |
| TCGA-F4-6808-01A-11R-1839-07 | 0.673549 |
| TCGA-AA-A00U-01A-01R-A002-07 | 0.812589 |
| TCGA-AA-A017-01A-01R-A00A-07 | 0.889579 |
| TCGA-AA-A01X-01A-21R-A083-07 | 0.820526 |
| TCGA-CM-5861-01A-01R-1653-07 | 0.886908 |
| TCGA-D5-5537-01A-21R-1928-07 | 0.769087 |
| TCGA-AA-3968-01A-01R-1022-07 | 0.903211 |
| TCGA-D5-6530-01A-11R-1723-07 | 0.716769 |
| TCGA-AA-3818-01A-01R-0905-07 | 0.800822 |
| TCGA-G4-6315-01A-11R-1723-07 | 0.784626 |
| TCGA-AA-3562-01A-02R-0821-07 | 0.841919 |
| TCGA-AA-3872-01A-01R-1022-07 | 1.16658 |
| TCGA-AA-3845-01A-01R-1022-07 | 0.888894 |
| TCGA-AA-A010-01A-01R-A089-07 | 0.918558 |
| TCGA-AA-3529-01A-02R-0821-07 | 0.712882 |
| TCGA-A6-2675-01A-02R-1723-07 | 0.97772 |
| TCGA-AG-A023-01A-01R-A00A-07 | 1.078305 |
| TCGA-DY-A1DF-01A-11R-A155-07 | 0.93292 |
| TCGA-AA-3952-01A-01R-1022-07 | 0.884535 |
| TCGA-AA-3532-01A-01R-0821-07 | 0.866793 |
| TCGA-AA-3989-01A-01R-1022-07 | 0.936928 |
| TCGA-AA-3530-01A-01R-1022-07 | 0.686958 |
| TCGA-NH-A5IV-01A-42R-A37K-07 | 0.6057 |
| TCGA-AA-3521-01A-01R-0821-07 | 0.88574 |
| TCGA-AA-3554-01A-01R-0826-07 | 0.976201 |
| TCGA-D5-5538-01A-01R-1653-07 | 0.935268 |
| TCGA-AG-A02X-01A-01R-A00A-07 | 0.689956 |
| TCGA-AF-2692-01A-01R-0821-07 | 0.765339 |
| TCGA-AG-3599-01A-02R-0826-07 | 0.72639 |
| TCGA-AG-A016-01A-01R-A002-07 | 0.812586 |
| TCGA-AF-A56L-01A-31R-A39D-07 | 0.809295 |
| TCGA-AG-3578-01A-01R-0821-07 | 0.797576 |
| TCGA-AG-3574-01A-01R-0821-07 | 0.832181 |
| TCGA-F5-6465-01A-11R-1736-07 | 1.007611 |
| TCGA-AG-3892-01A-01R-1119-07 | 0.663114 |
| TCGA-AG-3587-01A-01R-0821-07 | 0.848465 |
| TCGA-AG-A00Y-01A-02R-A002-07 | 0.769143 |
| TCGA-AG-3896-01A-01R-1119-07 | 0.943091 |
| TCGA-AG-3594-01A-02R-0821-07 | 0.78269 |
| TCGA-EI-7004-01A-11R-1928-07 | 1.33812 |
| TCGA-F5-6863-01A-11R-1928-07 | 0.966823 |
| TCGA-F5-6814-01A-31R-1928-07 | 0.867774 |
| TCGA-D5-6532-01A-11R-1723-07 | 0.748912 |
| TCGA-DM-A0XD-01A-12R-A155-07 | 0.713582 |
| TCGA-AA-3492-01A-01R-1410-07 | 0.736983 |
| TCGA-AA-A022-01A-21R-A16W-07 | 0.788701 |
| TCGA-AD-6899-01A-11R-1928-07 | 0.894747 |
| TCGA-AA-3844-01A-01R-1022-07 | 0.773243 |
| TCGA-AD-6901-01A-11R-1928-07 | 1.024298 |
| TCGA-AA-A01P-01A-21R-A083-07 | 1.091256 |
| TCGA-G4-6293-01A-11R-1723-07 | 0.7974 |
| TCGA-AA-3715-01A-01R-0905-07 | 0.950014 |
| TCGA-AY-A71X-01A-12R-A37K-07 | 0.505127 |
| TCGA-DM-A28C-01A-11R-A32Y-07 | 0.744964 |
| TCGA-AA-3560-01A-01R-0821-07 | 0.700279 |
| TCGA-AA-3552-01A-01R-0821-07 | 0.776556 |
| TCGA-CM-6166-01A-11R-1653-07 | 0.793013 |
| TCGA-AZ-5403-01A-01R-1653-07 | 0.995327 |
| TCGA-G4-6299-01A-11R-1774-07 | 0.917851 |
| TCGA-AA-3496-01A-21R-1839-07 | 0.917671 |
| TCGA-A6-5661-01A-01R-1653-07 | 0.720651 |
| TCGA-G4-6304-01A-11R-1928-07 | 0.552342 |
| TCGA-CM-5864-01A-01R-1653-07 | 0.823633 |
| TCGA-AY-A8YK-01A-11R-A41B-07 | 0.712838 |
| TCGA-DM-A28K-01A-21R-A32Y-07 | 0.766951 |
| TCGA-CK-5913-01A-11R-1653-07 | 0.839577 |
| TCGA-AZ-6605-01A-11R-1839-07 | 1.141669 |
| TCGA-CM-4751-01A-02R-1839-07 | 0.849864 |
| TCGA-CM-6677-01A-11R-1839-07 | 0.845382 |
| TCGA-G4-6627-01A-11R-1774-07 | 1.040897 |
| TCGA-CI-6622-01A-11R-1830-07 | 0.784724 |
| TCGA-F5-6861-01A-11R-1928-07 | 0.784269 |
| TCGA-AG-A036-01A-12R-A083-07 | 0.853318 |
| TCGA-AG-3898-01A-01R-1119-07 | 0.921426 |
| TCGA-AG-A011-01A-01R-A002-07 | 0.777159 |
| TCGA-AG-3575-01A-01R-0821-07 | 0.903409 |
| TCGA-DC-6683-01A-11R-1830-07 | 0.848912 |
| TCGA-EI-6882-01A-11R-1928-07 | 0.809818 |
| TCGA-DT-5265-01A-21R-1830-07 | 0.982293 |
| TCGA-DY-A0XA-01A-11R-A155-07 | 0.811788 |
| TCGA-AG-3600-01A-01R-0826-07 | 0.802248 |
| TCGA-DC-5869-01A-01R-1660-07 | 0.871752 |
| TCGA-CL-5918-01A-11R-1660-07 | 0.81507 |
| TCGA-DY-A1DG-01A-11R-A32Y-07 | 0.591228 |
| TCGA-CL-4957-01A-01R-1736-07 | 0.867433 |
| TCGA-AG-3586-01A-02R-0821-07 | 0.84706 |
| TCGA-DC-4749-01A-01R-1736-07 | 0.673295 |
| TCGA-G5-6235-01A-11R-1736-07 | 0.685885 |
| TCGA-AG-3612-01A-01R-0826-07 | 0.921143 |
| TCGA-AG-3725-01A-11R-1736-07 | 0.762622 |
| TCGA-EI-6509-01A-11R-1736-07 | 0.983073 |
| TCGA-AH-6547-01A-11R-1830-07 | 0.911631 |
| TCGA-AF-6672-01A-11R-1830-07 | 0.842507 |
| TCGA-AG-A01N-01A-01R-A00A-07 | 0.836573 |
| TCGA-AF-2691-01A-01R-0821-07 | 0.810731 |
| TCGA-AG-3883-01A-02R-0905-07 | 1.001514 |
| TCGA-DC-5337-01A-01R-1660-07 | 0.731896 |
| TCGA-AG-3592-01A-02R-1736-07 | 0.856534 |
| TCGA-AF-2690-01A-02R-1736-07 | 1.15243 |
| TCGA-F5-6811-01A-11R-1830-07 | 0.861652 |
| TCGA-AF-3400-01A-01R-0821-07 | 1.158982 |
| TCGA-AG-A008-01A-01R-A002-07 | 0.740263 |
| TCGA-AA-3544-01A-01R-1873-07 | 1.02395 |
| TCGA-A6-A567-01A-31R-A28H-07 | 0.831547 |
| TCGA-CM-6676-01A-11R-1839-07 | 0.763461 |
| TCGA-AA-3867-01A-01R-1022-07 | 1.02198 |
| TCGA-CA-6719-01A-11R-1839-07 | 1.074292 |
| TCGA-NH-A50V-01A-11R-A28H-07 | 0.942873 |
| TCGA-AA-A01C-01A-01R-A00A-07 | 0.817586 |
| TCGA-AA-A02F-01A-01R-A089-07 | 0.917021 |
| TCGA-AA-A03F-01A-11R-A16W-07 | 0.747098 |
| TCGA-AA-A00O-01A-02R-A089-07 | 1.21392 |
| TCGA-AZ-4615-01A-01R-1410-07 | 0.786372 |
| TCGA-AA-3854-01A-01R-0905-07 | 0.753793 |
| TCGA-AA-A01V-01A-23R-A083-07 | 0.714729 |
| TCGA-AA-A00D-01A-01R-A002-07 | 0.994054 |
| TCGA-A6-5657-01A-01R-A32Z-07 | 1.069601 |
| TCGA-AA-3522-01A-01R-0821-07 | 0.720305 |
| TCGA-AA-3877-01A-01R-1022-07 | 0.935455 |
| TCGA-D5-6923-01A-11R-A32Z-07 | 1.03218 |
| TCGA-AZ-6599-01A-11R-1774-07 | 0.667117 |
| TCGA-D5-6922-01A-11R-1928-07 | 0.887281 |
| TCGA-AA-3692-01A-01R-0905-07 | 0.734599 |
| TCGA-4T-AA8H-01A-11R-A41B-07 | 0.549201 |
| TCGA-AA-A01Q-01A-01R-A002-07 | 0.613033 |
| TCGA-AA-3979-01A-01R-1022-07 | 0.702634 |
| TCGA-A6-A565-01A-31R-A28H-07 | 0.969333 |
| TCGA-A6-5667-01A-21R-1723-07 | 0.834779 |
| TCGA-AA-3842-01A-01R-1022-07 | 0.845333 |
| TCGA-A6-A5ZU-01A-11R-A28H-07 | 0.958096 |
| TCGA-AA-3870-01A-01R-1022-07 | 1.019882 |
| TCGA-NH-A6GC-01A-12R-A41B-07 | 0.953957 |
| TCGA-AA-3511-01A-21R-1839-07 | 0.924232 |
| TCGA-CM-6167-01A-11R-1653-07 | 1.352259 |
| TCGA-CM-5348-01A-21R-1723-07 | 0.967651 |
| TCGA-AA-3949-01A-01R-1022-07 | 1.014885 |
| TCGA-AY-A54L-01A-11R-A28H-07 | 0.608801 |
| TCGA-A6-2671-01A-01R-1410-07 | 1.009668 |
| TCGA-NH-A8F8-01A-72R-A41B-07 | 0.96667 |
| TCGA-AA-3516-01A-02R-0826-07 | 0.881016 |
| TCGA-G4-6628-01A-11R-1839-07 | 0.962912 |
| TCGA-AD-6964-01A-11R-1928-07 | 1.001386 |
| TCGA-AD-6888-01A-11R-1928-07 | 0.503812 |
| TCGA-AA-3973-01A-01R-1022-07 | 0.930533 |
| TCGA-AA-3680-01A-01R-0905-07 | 0.793169 |
| TCGA-AA-3514-01A-02R-0821-07 | 0.993431 |
| TCGA-QG-A5YW-01A-11R-A28H-07 | 0.748051 |
| TCGA-AY-A69D-01A-11R-A37K-07 | 0.679029 |
| TCGA-5M-AAT5-01A-21R-A41B-07 | 0.663841 |
| TCGA-AA-3673-01A-01R-0905-07 | 0.846216 |
| TCGA-AA-3855-01A-01R-1022-07 | 0.792549 |
| TCGA-CM-6172-01A-11R-1653-07 | 0.812679 |
| TCGA-AZ-6600-01A-11R-1774-07 | 1.046857 |
| TCGA-F4-6463-01A-11R-1723-07 | 1.107106 |
| TCGA-AA-3971-01A-01R-1022-07 | 0.703813 |
| TCGA-AA-3666-01A-02R-0905-07 | 0.835652 |
| TCGA-D5-6898-01A-11R-1928-07 | 0.874164 |
| TCGA-D5-6537-01A-11R-1723-07 | 0.617486 |
| TCGA-SS-A7HO-01A-21R-A37K-07 | 0.751537 |
| TCGA-CK-5915-01A-11R-1653-07 | 0.686887 |
| TCGA-AA-A00L-01A-01R-A002-07 | 0.877672 |
| TCGA-G4-6321-01A-11R-1723-07 | 0.705649 |
| TCGA-AA-3489-01A-21R-1839-07 | 1.093493 |
| TCGA-AA-A01S-01A-21R-A083-07 | 0.552447 |
| TCGA-DM-A1HB-01A-21R-A180-07 | 0.795918 |
| TCGA-D5-6931-01A-11R-1928-07 | 0.805569 |
| TCGA-T9-A92H-01A-11R-A37K-07 | 0.644758 |
| TCGA-AD-6965-01A-11R-1928-07 | 0.792413 |
| TCGA-DM-A1D7-01A-11R-A155-07 | 0.73767 |
| TCGA-A6-6140-01A-11R-1774-07 | 0.765574 |
| TCGA-AA-3672-01A-01R-0905-07 | 0.898324 |
| TCGA-AA-3553-01A-01R-0821-07 | 0.910931 |
| TCGA-A6-A56B-01A-31R-A28H-07 | 0.93588 |
| TCGA-D5-6927-01A-21R-1928-07 | 0.870648 |
| TCGA-AA-A00E-01A-01R-A002-07 | 0.928287 |
| TCGA-AA-3814-01A-01R-0905-07 | 0.880211 |
| TCGA-A6-2676-01A-01R-0826-07 | 0.862792 |
| TCGA-AA-A02K-01A-03R-A32Y-07 | 0.631702 |
| TCGA-F4-6570-01A-11R-1774-07 | 1.071998 |
| TCGA-DM-A1D9-01A-11R-A155-07 | 0.693739 |
| TCGA-CK-4951-01A-01R-1410-07 | 0.948516 |
| TCGA-AA-3956-01A-02R-1022-07 | 0.877428 |
| TCGA-A6-2683-01A-01R-0821-07 | 0.732525 |
| TCGA-AA-3713-01A-21R-1723-07 | 0.784293 |
| TCGA-AA-3955-01A-02R-1022-07 | 0.766699 |
| TCGA-AZ-6598-01A-11R-1774-07 | 0.760781 |
| TCGA-F4-6704-01A-11R-1839-07 | 1.109564 |
| TCGA-CM-6163-01A-11R-1653-07 | 0.793742 |
| TCGA-AA-3869-01A-01R-1022-07 | 0.908681 |
| TCGA-AA-3538-01A-01R-0821-07 | 0.891909 |
| TCGA-DM-A1DA-01A-11R-A155-07 | 0.658944 |
| TCGA-AA-3494-01A-01R-1410-07 | 0.746879 |
| TCGA-A6-2677-01A-01R-0821-07 | 0.620187 |
| TCGA-CM-6168-01A-11R-1653-07 | 1.058371 |
| TCGA-AA-3852-01A-01R-0905-07 | 0.89763 |
| TCGA-AA-3851-01A-01R-1022-07 | 0.767464 |
| TCGA-A6-6654-01A-21R-1839-07 | 1.229665 |
| TCGA-DM-A28E-01A-11R-A32Y-07 | 0.674479 |
| TCGA-AA-3509-01A-01R-1410-07 | 0.904483 |
| TCGA-AA-3970-01A-01R-1022-07 | 0.798131 |
| TCGA-CK-5914-01A-11R-1653-07 | 0.808799 |
| TCGA-A6-5666-01A-01R-1653-07 | 0.713309 |
| TCGA-4N-A93T-01A-11R-A37K-07 | 0.585133 |
| TCGA-CM-6171-01A-11R-1653-07 | 0.734639 |
| TCGA-G4-6309-01A-21R-1839-07 | 0.650136 |
| TCGA-AA-3697-01A-01R-1723-07 | 0.75132 |
| TCGA-G4-6586-01A-11R-1774-07 | 0.656133 |
| TCGA-AZ-4616-01A-21R-1839-07 | 0.805307 |
| TCGA-D5-6928-01A-11R-1928-07 | 1.016408 |
| TCGA-AA-A02J-01A-01R-A00A-07 | 0.641477 |
| TCGA-D5-6538-01A-11R-1723-07 | 0.765292 |
| TCGA-NH-A6GB-01A-11R-A37K-07 | 0.753139 |
| TCGA-A6-2682-01A-01R-1410-07 | 0.878669 |
| TCGA-AA-A02Y-01A-43R-A32Y-07 | 0.580893 |
| TCGA-AA-3850-01A-01R-1022-07 | 0.904086 |
| TCGA-CK-6747-01A-11R-1839-07 | 0.798665 |
| TCGA-A6-6782-01A-11R-1839-07 | 1.047167 |
| TCGA-CM-5860-01A-01R-1653-07 | 1.071213 |
| TCGA-AA-A00R-01A-01R-A002-07 | 0.974476 |
| TCGA-A6-6653-01A-11R-1774-07 | 0.827185 |
| TCGA-AD-6890-01A-11R-1928-07 | 0.758244 |
| TCGA-D5-6539-01A-11R-1723-07 | 0.816129 |
| TCGA-AD-A5EK-01A-11R-A28H-07 | 0.797958 |
| TCGA-A6-3808-01A-01R-1022-07 | 1.050215 |
| TCGA-AA-3506-01A-01R-1410-07 | 0.807598 |
| TCGA-G4-6298-01A-11R-1723-07 | 0.92147 |
| TCGA-AZ-6608-01A-11R-1839-07 | 0.615521 |
| TCGA-AA-3678-01A-01R-0905-07 | 0.772436 |
| TCGA-A6-5665-01A-01R-1653-07 | 0.547898 |
| TCGA-G4-6306-01A-11R-1774-07 | 0.611276 |
| TCGA-F4-6806-01A-11R-1839-07 | 0.833348 |
| TCGA-AA-A01T-01A-21R-A16W-07 | 0.757539 |
| TCGA-A6-6651-01A-21R-1839-07 | 1.22593 |
| TCGA-AA-3812-01A-01R-0905-07 | 0.956103 |
| TCGA-F4-6809-01A-11R-1839-07 | 1.080437 |
| TCGA-AD-5900-01A-11R-1653-07 | 0.84742 |
| TCGA-D5-6930-01A-11R-1928-07 | 0.920515 |
| TCGA-AA-A03J-01A-21R-A16W-07 | 0.814881 |
| TCGA-AA-3815-01A-01R-1022-07 | 0.799448 |
| TCGA-CM-6169-01A-11R-1653-07 | 1.207344 |
| TCGA-F4-6855-01A-11R-1928-07 | 1.119352 |
| TCGA-AG-3894-01A-01R-1119-07 | 0.869977 |
| TCGA-AG-3885-01A-01R-0905-07 | 0.867423 |
| TCGA-AG-3882-01A-01R-0905-07 | 0.921483 |
| TCGA-AG-3726-01A-02R-0905-07 | 1.017425 |
| TCGA-AF-4110-01A-02R-1736-07 | 1.039804 |
| TCGA-AH-6644-01A-21R-1830-07 | 1.029933 |
| TCGA-AG-A02N-01A-11R-A083-07 | 0.590433 |
| TCGA-AG-3609-01A-02R-0826-07 | 1.001355 |
| TCGA-AG-A01J-01A-01R-A00A-07 | 0.759723 |
| TCGA-AG-4022-01A-01R-1736-07 | 0.939998 |
| TCGA-AG-4021-01A-01R-1736-07 | 0.937618 |
| TCGA-G5-6233-01A-11R-1736-07 | 0.8773 |
| TCGA-AF-6655-01A-11R-1830-07 | 0.938713 |
| TCGA-A6-A566-01A-11R-A28H-07 | 1.336764 |
| TCGA-CA-6715-01A-21R-1839-07 | 0.769392 |
| TCGA-NH-A6GA-01A-11R-A37K-07 | 0.796612 |
| TCGA-AA-A004-01A-01R-A00A-07 | 1.148416 |
| TCGA-AD-6889-01A-11R-1928-07 | 0.645851 |
| TCGA-D5-6536-01A-11R-1723-07 | 1.14876 |
| TCGA-G4-6322-01A-11R-1723-07 | 0.797795 |
| TCGA-AA-A029-01A-01R-A00A-07 | 0.645161 |
| TCGA-AZ-5407-01A-01R-1723-07 | 0.653947 |
| TCGA-AA-A00N-01A-02R-A00A-07 | 1.06575 |
| TCGA-D5-6540-01A-11R-1723-07 | 0.896569 |
| TCGA-AA-3548-01A-01R-1873-07 | 0.75756 |
| TCGA-A6-6649-01A-11R-1774-07 | 0.988517 |
| TCGA-DM-A282-01A-12R-A16W-07 | 0.704841 |
| TCGA-AA-3655-01A-02R-1723-07 | 0.812259 |
| TCGA-G4-6311-01A-11R-1723-07 | 0.889558 |
| TCGA-G4-6297-01A-11R-1723-07 | 1.095714 |
| TCGA-5M-AAT6-01A-11R-A41B-07 | 1.093846 |
| TCGA-D5-6534-01A-21R-1928-07 | 1.232282 |
| TCGA-DM-A1DB-01A-11R-A155-07 | 0.618706 |
| TCGA-CM-6678-01A-11R-1839-07 | 0.892892 |
| TCGA-AA-3660-01A-01R-1723-07 | 0.767963 |
| TCGA-DM-A0XF-01A-11R-A155-07 | 0.62118 |
| TCGA-QG-A5Z1-01A-11R-A28H-07 | 0.990302 |
| TCGA-AG-4015-01A-01R-1119-07 | 0.788213 |
| TCGA-AG-3731-01A-11R-1736-07 | 1.108288 |
| TCGA-AG-3611-01A-01R-0826-07 | 0.590321 |
| TCGA-EI-6885-01A-11R-1928-07 | 0.988005 |
| TCGA-AG-4007-01A-01R-1119-07 | 0.945673 |
| TCGA-AG-3581-01A-01R-0821-07 | 0.929202 |
| TCGA-AA-3526-01A-02R-A32Z-07 | 0.727798 |
| TCGA-CA-5255-01A-11R-1839-07 | 0.610921 |
| TCGA-AA-3977-01A-01R-1022-07 | 0.762409 |
| TCGA-AA-A01F-01A-01R-A002-07 | 0.782984 |
| TCGA-D5-5540-01A-01R-1653-07 | 0.625056 |
| TCGA-5M-AAT4-01A-11R-A41B-07 | 0.748076 |
| TCGA-AA-3864-01A-01R-1022-07 | 0.77852 |
| TCGA-CA-6717-01A-11R-1839-07 | 1.0394 |
| TCGA-CM-6161-01A-11R-1653-07 | 0.862035 |
| TCGA-AA-3819-01A-01R-0905-07 | 0.734616 |
| TCGA-AA-3861-01A-01R-1022-07 | 0.666251 |
| TCGA-D5-6926-01A-11R-1928-07 | 1.017926 |
| TCGA-AA-3531-01A-01R-0821-07 | 0.751951 |
| TCGA-AA-3685-01A-02R-A32Z-07 | 0.731351 |
| TCGA-AA-3712-01A-21R-1723-07 | 0.905631 |
| TCGA-AA-3837-01A-01R-0905-07 | 0.828776 |
| TCGA-AA-3664-01A-01R-0905-07 | 0.729261 |
| TCGA-D5-6929-01A-31R-1928-07 | 0.834128 |
| TCGA-A6-6648-01A-11R-1774-07 | 0.800886 |
| TCGA-F4-6569-01A-11R-1774-07 | 1.267478 |
| TCGA-AA-3966-01A-01R-1113-07 | 1.040142 |
| TCGA-AZ-4684-01A-01R-1410-07 | 0.86919 |
| TCGA-3L-AA1B-01A-11R-A37K-07 | 0.892524 |
| TCGA-AA-A00A-01A-01R-A002-07 | 0.920272 |
| TCGA-AA-3950-01A-02R-1022-07 | 1.00792 |
| TCGA-DM-A285-01A-11R-A16W-07 | 0.810577 |
| TCGA-CM-5349-01A-21R-1723-07 | 0.949408 |
| TCGA-AA-A00J-01A-02R-A002-07 | 0.937107 |
| TCGA-AY-6196-01A-11R-1723-07 | 1.256321 |
| TCGA-AA-3534-01A-01R-0821-07 | 0.875301 |
| TCGA-G4-6302-01A-11R-1723-07 | 1.306262 |
| TCGA-AG-3901-01A-01R-1119-07 | 1.005906 |
| TCGA-DC-6158-01A-11R-1660-07 | 1.077436 |
| TCGA-F5-6571-01A-12R-1830-07 | 0.997194 |
| TCGA-AG-3582-01A-01R-0821-07 | 0.887977 |
| TCGA-EI-6508-01A-11R-1736-07 | 0.714326 |
| TCGA-CL-5917-01A-11R-1660-07 | 0.79926 |
| TCGA-AF-2687-01A-02R-1736-07 | 1.080599 |
| TCGA-EF-5831-01A-01R-1660-07 | 0.951887 |
| TCGA-DC-6157-01A-11R-1660-07 | 0.883602 |
| TCGA-AG-A00C-01A-01R-A002-07 | 0.622382 |
| TCGA-AF-A56N-01A-12R-A39D-07 | 0.906001 |
| TCGA-DY-A1DE-01A-11R-A155-07 | 0.874075 |
| TCGA-AG-A01L-01A-01R-A002-07 | 0.764404 |
| TCGA-AF-2693-01A-02R-1736-07 | 0.930621 |
| TCGA-DC-6681-01A-11R-A32Z-07 | 0.945247 |
| TCGA-AH-6903-01A-11R-1928-07 | 0.772912 |
| TCGA-AG-A025-01A-01R-A00A-07 | 0.955251 |
| TCGA-AG-3902-01A-01R-A32Z-07 | 0.685805 |
| TCGA-AG-3893-01A-01R-1119-07 | 0.898441 |
| TCGA-F5-6812-01A-11R-1830-07 | 1.093668 |
| TCGA-AG-3881-01A-01R-0905-07 | 1.165902 |
| TCGA-AG-A014-01A-02R-A002-07 | 0.923597 |
| TCGA-AG-3580-01A-01R-0821-07 | 0.748508 |
| TCGA-EI-6884-01A-11R-1928-07 | 0.899643 |
| TCGA-AF-6136-01A-11R-1830-07 | 0.798172 |
| TCGA-AF-3913-01A-02R-1119-07 | 0.82608 |
| TCGA-AA-3663-01A-01R-1723-07 | 0.766616 |
| TCGA-AA-3549-01A-02R-0821-07 | 0.976126 |
| TCGA-AA-3561-01A-01R-0821-07 | 0.761198 |
| TCGA-G4-6626-01A-11R-1774-07 | 0.668516 |
| TCGA-AA-3821-01A-01R-1022-07 | 0.7049 |
| TCGA-CK-4952-01A-01R-1723-07 | 0.781537 |
| TCGA-A6-2681-01A-01R-1410-07 | 1.047679 |
| TCGA-AY-6197-01A-11R-1723-07 | 0.698136 |
| TCGA-AY-6386-01A-21R-1723-07 | 0.721989 |
| TCGA-AU-3779-01A-01R-1723-07 | 0.814137 |
| TCGA-CM-4744-01A-01R-A32Z-07 | 0.661783 |
| TCGA-AD-A5EJ-01A-11R-A28H-07 | 0.784835 |
| TCGA-AG-A02G-01A-01R-A00A-07 | 0.783749 |
| TCGA-DC-6154-01A-31R-1928-07 | 0.897818 |
| TCGA-AG-3584-01A-01R-0821-07 | 0.820764 |
| TCGA-AG-A032-01A-01R-A00A-07 | 0.881055 |
| TCGA-G5-6641-01A-11R-A32Z-07 | 0.668073 |
| TCGA-AG-3890-01A-01R-1119-07 | 0.810816 |
| TCGA-AG-3742-01A-11R-1660-07 | 0.92829 |
| TCGA-AG-A026-01A-01R-A00A-07 | 0.978437 |
| TCGA-F5-6702-01A-11R-1830-07 | 1.143335 |
| TCGA-G5-6572-01A-11R-1830-07 | 0.991553 |
| TCGA-F5-6464-01A-11R-1736-07 | 1.238376 |
| TCGA-EF-5830-01A-01R-1660-07 | 0.739987 |
| TCGA-DC-6682-01A-11R-1830-07 | 0.765334 |
| TCGA-AG-3727-01A-01R-0905-07 | 1.047548 |
| TCGA-AG-A01Y-01A-41R-A083-07 | 0.909108 |
| TCGA-AG-3598-01A-01R-0826-07 | 0.737246 |
| TCGA-CM-4748-01A-01R-1410-07 | 0.803299 |
| TCGA-AA-3517-01A-01R-0821-07 | 0.757561 |
| TCGA-D5-5541-01A-01R-1653-07 | 0.834791 |
| TCGA-A6-2686-01A-01R-A32Z-07 | 0.69466 |
| TCGA-NH-A50U-01A-33R-A37K-07 | 0.850219 |
| TCGA-AA-3543-01A-01R-0826-07 | 0.827554 |
| TCGA-AA-3518-01A-02R-0826-07 | 0.731791 |
| TCGA-AA-3984-01A-02R-1022-07 | 0.920957 |
| TCGA-CM-4752-01A-01R-1410-07 | 0.944361 |
| TCGA-AA-3684-01A-02R-0905-07 | 1.092126 |
| TCGA-AA-3941-01A-01R-1022-07 | 0.767323 |
| TCGA-A6-5660-01A-01R-1653-07 | 0.877786 |
| TCGA-A6-6652-01A-11R-1774-07 | 0.847054 |
| TCGA-CM-5868-01A-01R-1653-07 | 0.795261 |
| TCGA-CM-6164-01A-11R-1653-07 | 0.921557 |
| TCGA-AZ-6606-01A-11R-1839-07 | 0.768739 |
| TCGA-DM-A1HA-01A-11R-A155-07 | 0.665039 |
| TCGA-G4-6323-01A-11R-1723-07 | 0.590571 |
| TCGA-AA-3542-01A-02R-1873-07 | 0.756374 |
| TCGA-D5-6924-01A-11R-1928-07 | 0.982476 |
| TCGA-F4-6856-01A-11R-1928-07 | 0.820548 |
| TCGA-QG-A5YV-01A-11R-A28H-07 | 0.684496 |
| TCGA-AA-3688-01A-01R-0905-07 | 0.818816 |
| TCGA-AA-3980-01A-02R-1022-07 | 0.816637 |
| TCGA-AA-3525-01A-02R-0826-07 | 0.678086 |
| TCGA-AA-3994-01A-01R-1113-07 | 0.885038 |
| TCGA-AY-4070-01A-01R-1113-07 | 0.834792 |
| TCGA-AA-3495-01A-01R-1410-07 | 0.727987 |
| TCGA-D5-6531-01A-11R-1723-07 | 0.842271 |
| TCGA-CA-5254-01A-21R-1839-07 | 0.837043 |
| TCGA-QL-A97D-01A-12R-A41B-07 | 0.691983 |
| TCGA-AA-A02R-01A-01R-A00A-07 | 0.874373 |
| TCGA-CK-6751-01A-11R-1839-07 | 0.927246 |
| TCGA-F4-6805-01A-11R-1839-07 | 1.065675 |
| TCGA-AA-3696-01A-01R-0905-07 | 0.847018 |
| TCGA-A6-5662-01A-01R-1653-07 | 0.981574 |
| TCGA-AZ-6601-01A-11R-1774-07 | 0.860961 |
| TCGA-AA-3556-01A-01R-0821-07 | 0.825524 |
| TCGA-DM-A1D4-01A-21R-A155-07 | 0.57675 |
| TCGA-RU-A8FL-01A-11R-A37K-07 | 0.495926 |
| TCGA-AA-3975-01A-01R-1022-07 | 0.945919 |
| TCGA-F4-6460-01A-11R-1774-07 | 1.03303 |
| TCGA-AZ-4308-01A-01R-1410-07 | 0.971271 |
| TCGA-AA-3972-01A-01R-1022-07 | 0.844454 |
| TCGA-AA-3858-01A-01R-0905-07 | 0.867868 |
| TCGA-AA-3679-01A-02R-0905-07 | 0.83589 |
| TCGA-AA-A01D-01A-01R-A00A-07 | 1.034524 |
| TCGA-AY-5543-01A-01R-1653-07 | 0.673953 |
| TCGA-AU-6004-01A-11R-1723-07 | 0.832536 |
| TCGA-DM-A28A-01A-21R-A32Y-07 | 0.828894 |
| TCGA-AA-3848-01A-01R-0905-07 | 0.75208 |
| TCGA-AA-3667-01A-01R-0905-07 | 0.750578 |
| TCGA-F5-6810-01A-11R-1830-07 | 1.073733 |
| TCGA-AG-A01W-01A-21R-A083-07 | 0.835948 |
| TCGA-AG-3878-01A-02R-0905-07 | 1.050419 |
| TCGA-EI-6507-01A-11R-1736-07 | 0.887371 |
| TCGA-CI-6621-01A-11R-1830-07 | 1.013006 |
| TCGA-AG-A020-01A-21R-A083-07 | 0.72789 |
| TCGA-AG-4008-01A-01R-1119-07 | 0.999554 |
| TCGA-AG-3602-01A-02R-0826-07 | 0.758068 |
| TCGA-AG-3909-01A-01R-1119-07 | 0.787722 |
| TCGA-DC-6156-01A-11R-1660-07 | 1.139178 |
| TCGA-EI-6510-01A-11R-1736-07 | 0.668291 |
| TCGA-AG-3728-01A-01R-0905-07 | 1.069164 |
| TCGA-AG-A015-01A-01R-A002-07 | 0.751229 |
| TCGA-CI-6620-01A-11R-1830-07 | 0.964669 |
| TCGA-AH-6643-01A-11R-1830-07 | 1.029249 |
| TCGA-EI-6513-01A-21R-1736-07 | 0.782614 |
| TCGA-EI-6506-01A-11R-1736-07 | 0.77736 |
| TCGA-AG-4005-01A-01R-1119-07 | 0.945542 |
| TCGA-AF-A56K-01A-32R-A39D-07 | 1.099138 |
| TCGA-AA-A00F-01A-01R-A002-07 | 0.970933 |
| TCGA-AA-3866-01A-01R-1022-07 | 0.898133 |
| TCGA-AA-A01K-01A-01R-A00A-07 | 0.981597 |
| TCGA-CM-5863-01A-21R-1839-07 | 1.003914 |
| TCGA-D5-6932-01A-11R-1928-07 | 1.011043 |
| TCGA-AA-3675-01A-02R-0905-07 | 0.814785 |
| TCGA-AA-3681-01A-01R-0905-07 | 0.830235 |
| TCGA-AA-A00Q-01A-01R-A002-07 | 0.881138 |
| TCGA-AM-5820-01A-01R-1653-07 | 0.842453 |
| TCGA-CK-6746-01A-11R-1839-07 | 0.740166 |
| TCGA-AA-A00W-01A-01R-A002-07 | 0.720086 |
| TCGA-AZ-4323-01A-21R-1839-07 | 1.043432 |
| TCGA-CM-4746-01A-01R-1410-07 | 0.656668 |
| TCGA-AG-A00H-01A-01R-A00A-07 | 0.889993 |
| TCGA-EI-6511-01A-11R-1736-07 | 0.930221 |
| TCGA-EI-6883-01A-31R-1928-07 | 0.800327 |
| TCGA-DC-6155-01A-11R-1660-07 | 0.803168 |
| TCGA-AG-3593-01A-01R-0821-07 | 0.815532 |
| TCGA-AG-3601-01A-01R-0826-07 | 0.931394 |
| TCGA-A6-6141-01A-11R-1774-07 | 0.778772 |
| TCGA-DM-A280-01A-12R-A16W-07 | 0.868581 |
| TCGA-AA-3939-01A-01R-1022-07 | 0.788972 |
| TCGA-A6-2679-01A-02R-1410-07 | 0.755304 |
| TCGA-AA-3502-01A-01R-1410-07 | 0.571593 |
| TCGA-D5-6533-01A-11R-1723-07 | 0.92359 |
| TCGA-AA-3693-01A-01R-0905-07 | 0.727283 |
| TCGA-AD-6548-01A-11R-1839-07 | 0.907276 |
| TCGA-CM-6170-01A-11R-1653-07 | 0.851659 |
| TCGA-F4-6459-01A-11R-1774-07 | 1.043078 |
| TCGA-DM-A1D0-01A-11R-A155-07 | 0.651976 |
| TCGA-NH-A8F7-01A-11R-A41B-07 | 0.637287 |
| TCGA-AA-A02W-01A-01R-A00A-07 | 0.737491 |
| TCGA-AA-3524-01A-02R-0821-07 | 0.640911 |
| TCGA-AA-3841-01A-01R-0905-07 | 0.908293 |
| TCGA-A6-5659-01A-01R-A278-07 | 0.831825 |
| TCGA-A6-2674-01A-02R-A278-07 | 1.063871 |
| TCGA-A6-6650-01A-11R-A278-07 | 0.620816 |
| TCGA-A6-3810-01A-01R-A278-07 | 1.012477 |
| TCGA-A6-5656-01A-21R-A278-07 | 0.751414 |
| TCGA-A6-6780-01A-11R-A278-07 | 0.59737 |
| TCGA-A6-2684-01A-01R-A278-07 | 0.852856 |
| TCGA-A6-3809-01A-01R-A278-07 | 0.635204 |
| TCGA-A6-6781-01A-22R-A278-07 | 1.116751 |

Table S3 ssGSEA of immune cell infiltration.

| ID | Activated B cell | Activated CD4 T cell | Activated CD8 T cell | Activated dendritic cell | CD56bright natural killer cell | CD56dim natural killer cell | Central memory CD4 T cell | Central memory CD8 T cell | Effector memeory CD4 T cell | Effector memeory CD8 T cell | Eosinophil | Gamma delta T cell | Immature B cell | Immature dendritic cell | Macrophage | Mast cell | MDSC | Memory B cell | Monocyte | Natural killer cell | Natural killer T cell | Neutrophil | Plasmacytoid dendritic cell | Regulatory T cell | T follicular helper cell | Type 1 T helper cell | Type 17 T helper cell | Type 2 T helper cell |
| --- | --- | --- | --- | --- | --- | --- | --- | --- | --- | --- | --- | --- | --- | --- | --- | --- | --- | --- | --- | --- | --- | --- | --- | --- | --- | --- | --- | --- |
| TCGA-AH-6643-11A-01R-1830-07 | 0.523366 | 0.454622 | 0.548951 | 0.400536 | 0.437208 | 0.555061 | 0.679106 | 0.535002 | 0.436655 | 0.411431 | 0.237603 | 0.505406 | 0.508907 | 0.475941 | 0.304621 | 0.370672 | 0.571072 | 0.398964 | 0.581411 | 0.505575 | 0.263257 | 0.213835 | 0.567188 | 0.463206 | 0.386448 | 0.410597 | 0.27124 | 0.347395 |
| TCGA-AA-3655-11A-01R-1723-07 | 0.318852 | 0.39244 | 0.540176 | 0.403853 | 0.447972 | 0.543006 | 0.654402 | 0.519455 | 0.407902 | 0.405724 | 0.146555 | 0.51181 | 0.302397 | 0.486742 | 0.301632 | 0.348669 | 0.526696 | 0.306236 | 0.582363 | 0.496991 | 0.26165 | 0.11563 | 0.531421 | 0.42228 | 0.379907 | 0.378684 | 0.263743 | 0.317903 |
| TCGA-AA-3489-11A-01R-1839-07 | 0.240185 | 0.427629 | 0.557683 | 0.431951 | 0.438202 | 0.515872 | 0.659008 | 0.542087 | 0.463111 | 0.455377 | 0.17833 | 0.550477 | 0.300642 | 0.534501 | 0.364157 | 0.408848 | 0.620794 | 0.239604 | 0.575566 | 0.528462 | 0.289033 | 0.25025 | 0.55137 | 0.508921 | 0.382872 | 0.39916 | 0.270427 | 0.374102 |
| TCGA-A6-2685-11A-01R-A32Z-07 | 0.246917 | 0.432082 | 0.520907 | 0.412243 | 0.417874 | 0.549192 | 0.647215 | 0.510124 | 0.367306 | 0.413145 | 0.199829 | 0.484296 | 0.267074 | 0.471717 | 0.245012 | 0.303687 | 0.493272 | 0.311574 | 0.56857 | 0.463982 | 0.25761 | 0.124531 | 0.528923 | 0.378738 | 0.335588 | 0.369904 | 0.284483 | 0.335542 |
| TCGA-A6-2683-11A-01R-A32Z-07 | 0.332934 | 0.392401 | 0.47707 | 0.371675 | 0.443244 | 0.572491 | 0.655304 | 0.544928 | 0.348645 | 0.360305 | 0.19095 | 0.543759 | 0.311827 | 0.486914 | 0.296857 | 0.335453 | 0.528125 | 0.347284 | 0.573226 | 0.459448 | 0.257011 | 0.2127 | 0.552114 | 0.433909 | 0.361136 | 0.369266 | 0.294541 | 0.304708 |
| TCGA-AA-3531-11A-01R-A32Z-07 | 0.380258 | 0.377708 | 0.492623 | 0.385785 | 0.442959 | 0.589168 | 0.642023 | 0.512112 | 0.366245 | 0.38623 | 0.161515 | 0.507674 | 0.371904 | 0.458322 | 0.272991 | 0.329762 | 0.502021 | 0.341576 | 0.584526 | 0.438923 | 0.263271 | 0.230356 | 0.542629 | 0.40506 | 0.36328 | 0.372731 | 0.286335 | 0.283309 |
| TCGA-AA-3660-11A-01R-1723-07 | 0.313473 | 0.392737 | 0.548893 | 0.382138 | 0.447712 | 0.567073 | 0.652519 | 0.507314 | 0.30817 | 0.388187 | 0.132519 | 0.512756 | 0.30197 | 0.472588 | 0.292162 | 0.319507 | 0.534741 | 0.338009 | 0.587996 | 0.459481 | 0.254648 | 0.097337 | 0.529672 | 0.407346 | 0.358202 | 0.378766 | 0.256657 | 0.300321 |
| TCGA-AA-3663-11A-01R-1723-07 | 0.320342 | 0.374048 | 0.46523 | 0.386253 | 0.447481 | 0.560609 | 0.676437 | 0.537404 | 0.423734 | 0.405936 | 0.204138 | 0.525153 | 0.304722 | 0.499377 | 0.331483 | 0.408412 | 0.580848 | 0.316551 | 0.572124 | 0.499231 | 0.285349 | 0.206873 | 0.561568 | 0.468963 | 0.380413 | 0.376619 | 0.238351 | 0.319605 |
| TCGA-A6-2675-11A-01R-1723-07 | 0.129857 | 0.287914 | 0.37812 | 0.34154 | 0.41369 | 0.553547 | 0.653927 | 0.516492 | 0.424005 | 0.338318 | 0.169537 | 0.467505 | 0.180934 | 0.479388 | 0.237766 | 0.325505 | 0.430584 | 0.258077 | 0.548438 | 0.504058 | 0.255241 | 0.153005 | 0.547516 | 0.384219 | 0.327945 | 0.352283 | 0.226581 | 0.312805 |
| TCGA-AA-3525-11A-01R-A32Z-07 | 0.515971 | 0.474389 | 0.598275 | 0.433277 | 0.457305 | 0.590742 | 0.685126 | 0.530517 | 0.389736 | 0.477679 | 0.17517 | 0.533748 | 0.472529 | 0.484926 | 0.323461 | 0.340993 | 0.599056 | 0.36384 | 0.599414 | 0.462873 | 0.281474 | 0.172608 | 0.560395 | 0.473692 | 0.396946 | 0.400332 | 0.333424 | 0.293901 |
| TCGA-AA-3496-11A-01R-1839-07 | 0.250335 | 0.370431 | 0.457543 | 0.374976 | 0.435264 | 0.53847 | 0.676052 | 0.526639 | 0.434973 | 0.404807 | 0.156616 | 0.534356 | 0.25354 | 0.510265 | 0.324739 | 0.365384 | 0.564739 | 0.324468 | 0.574324 | 0.518721 | 0.296815 | 0.187377 | 0.560882 | 0.498943 | 0.371814 | 0.385884 | 0.239641 | 0.342578 |
| TCGA-AG-3731-11A-01R-1736-07 | 0.365021 | 0.382454 | 0.495105 | 0.39366 | 0.442057 | 0.530766 | 0.672722 | 0.550928 | 0.442082 | 0.427371 | 0.256776 | 0.526673 | 0.345728 | 0.52031 | 0.37777 | 0.4142 | 0.580503 | 0.30293 | 0.598075 | 0.508344 | 0.264705 | 0.145174 | 0.567577 | 0.478969 | 0.386316 | 0.389183 | 0.250746 | 0.314369 |
| TCGA-AG-3725-11A-01R-1736-07 | 0.286835 | 0.407622 | 0.525477 | 0.387888 | 0.446938 | 0.544201 | 0.643047 | 0.516187 | 0.39311 | 0.387421 | 0.15369 | 0.554078 | 0.273707 | 0.498616 | 0.31961 | 0.350808 | 0.545155 | 0.276545 | 0.585929 | 0.466583 | 0.266062 | 0.122486 | 0.532848 | 0.437935 | 0.397039 | 0.382902 | 0.255924 | 0.329031 |
| TCGA-AG-3742-11A-01R-1660-07 | 0.257045 | 0.380928 | 0.467517 | 0.366488 | 0.433424 | 0.4949 | 0.656623 | 0.538457 | 0.421091 | 0.39048 | 0.188498 | 0.532987 | 0.273236 | 0.511702 | 0.332565 | 0.386426 | 0.545459 | 0.309122 | 0.558381 | 0.505046 | 0.258788 | 0.18193 | 0.555232 | 0.457095 | 0.387214 | 0.372707 | 0.221864 | 0.350474 |
| TCGA-AF-2691-11A-01R-A32Z-07 | 0.386498 | 0.441295 | 0.471237 | 0.390967 | 0.433315 | 0.55144 | 0.665282 | 0.546933 | 0.414141 | 0.374342 | 0.247715 | 0.503752 | 0.356923 | 0.495603 | 0.320231 | 0.361133 | 0.538712 | 0.355092 | 0.575627 | 0.499423 | 0.282369 | 0.176144 | 0.557316 | 0.438767 | 0.379562 | 0.394758 | 0.290888 | 0.339513 |
| TCGA-AZ-6598-11A-01R-1774-07 | 0.313319 | 0.374441 | 0.49216 | 0.367986 | 0.428535 | 0.588994 | 0.659292 | 0.484738 | 0.400958 | 0.373926 | 0.205715 | 0.479981 | 0.28497 | 0.442037 | 0.236269 | 0.278585 | 0.471707 | 0.309384 | 0.554313 | 0.484176 | 0.258371 | 0.085308 | 0.526642 | 0.347594 | 0.339029 | 0.354441 | 0.242014 | 0.314892 |
| TCGA-A6-2678-11A-01R-A32Z-07 | 0.310079 | 0.355154 | 0.43911 | 0.354421 | 0.426011 | 0.569223 | 0.655123 | 0.531606 | 0.365239 | 0.399487 | 0.226392 | 0.52008 | 0.291809 | 0.475739 | 0.287993 | 0.364674 | 0.531162 | 0.332582 | 0.570919 | 0.477339 | 0.283171 | 0.141369 | 0.545518 | 0.427371 | 0.349061 | 0.364246 | 0.258419 | 0.300759 |
| TCGA-A6-2679-11A-01R-A32Z-07 | 0.370009 | 0.439468 | 0.60855 | 0.43388 | 0.443042 | 0.591943 | 0.68332 | 0.541035 | 0.369336 | 0.493247 | 0.207461 | 0.548817 | 0.364893 | 0.487241 | 0.306899 | 0.383157 | 0.613711 | 0.335536 | 0.587321 | 0.50277 | 0.305988 | 0.207155 | 0.546444 | 0.487109 | 0.403203 | 0.409543 | 0.287663 | 0.321904 |
| TCGA-AA-3527-11A-01R-A32Z-07 | 0.29431 | 0.37659 | 0.496307 | 0.393703 | 0.436133 | 0.580867 | 0.657683 | 0.546473 | 0.374651 | 0.409928 | 0.169521 | 0.523044 | 0.298755 | 0.497432 | 0.300821 | 0.332929 | 0.563333 | 0.311751 | 0.580134 | 0.498446 | 0.266069 | 0.185177 | 0.54475 | 0.4623 | 0.372162 | 0.371541 | 0.297046 | 0.308242 |
| TCGA-AZ-6600-11A-01R-1774-07 | 0.341897 | 0.368772 | 0.450241 | 0.369324 | 0.447588 | 0.572531 | 0.645415 | 0.505209 | 0.364328 | 0.34625 | 0.230429 | 0.493905 | 0.302768 | 0.476119 | 0.251979 | 0.331653 | 0.457103 | 0.295988 | 0.564595 | 0.469539 | 0.276862 | 0.08629 | 0.52355 | 0.326704 | 0.331994 | 0.373464 | 0.271327 | 0.312854 |
| TCGA-AA-3517-11A-01R-A32Z-07 | 0.519127 | 0.416881 | 0.56621 | 0.399789 | 0.451563 | 0.592736 | 0.664343 | 0.512937 | 0.397046 | 0.431815 | 0.18466 | 0.522425 | 0.46914 | 0.473704 | 0.297886 | 0.334402 | 0.564513 | 0.351721 | 0.585652 | 0.476687 | 0.274746 | 0.16282 | 0.534091 | 0.455224 | 0.394594 | 0.401331 | 0.274833 | 0.290418 |
| TCGA-A6-5659-11A-01R-1653-07 | 0.342506 | 0.384735 | 0.447704 | 0.3641 | 0.431799 | 0.570719 | 0.63712 | 0.52069 | 0.395339 | 0.36321 | 0.269811 | 0.499363 | 0.347986 | 0.474907 | 0.271881 | 0.337088 | 0.528039 | 0.323239 | 0.555873 | 0.4577 | 0.258773 | 0.249682 | 0.515035 | 0.400641 | 0.329459 | 0.36164 | 0.257502 | 0.29943 |
| TCGA-AZ-6605-11A-01R-1839-07 | 0.036662 | 0.179442 | 0.3423 | 0.310734 | 0.412388 | 0.495188 | 0.652453 | 0.467878 | 0.477431 | 0.372878 | 0.07047 | 0.438252 | 0.141245 | 0.490754 | 0.277702 | 0.253985 | 0.420872 | 0.234525 | 0.525722 | 0.495659 | 0.261916 | 0.022552 | 0.541146 | 0.394178 | 0.311316 | 0.326884 | 0.147317 | 0.302563 |
| TCGA-A6-2680-11A-01R-A32Z-07 | 0.138646 | 0.283158 | 0.44945 | 0.344512 | 0.419927 | 0.531028 | 0.652169 | 0.498922 | 0.377048 | 0.364762 | 0.188726 | 0.498636 | 0.186185 | 0.478151 | 0.24866 | 0.329416 | 0.449306 | 0.297181 | 0.609676 | 0.471892 | 0.250635 | 0.250763 | 0.525856 | 0.333396 | 0.341015 | 0.375254 | 0.218898 | 0.273299 |
| TCGA-AA-3516-11A-01R-A32Z-07 | 0.298436 | 0.327135 | 0.565725 | 0.397761 | 0.421737 | 0.578938 | 0.654019 | 0.512536 | 0.366447 | 0.428595 | 0.170384 | 0.513569 | 0.31444 | 0.462887 | 0.281664 | 0.292776 | 0.540842 | 0.287292 | 0.589751 | 0.484346 | 0.281763 | 0.196501 | 0.533126 | 0.410142 | 0.366736 | 0.384188 | 0.28944 | 0.312071 |
| TCGA-A6-5667-11A-01R-1723-07 | 0.348989 | 0.396682 | 0.448128 | 0.363705 | 0.428928 | 0.56787 | 0.644411 | 0.523331 | 0.335885 | 0.367404 | 0.259018 | 0.488475 | 0.330364 | 0.472423 | 0.272486 | 0.339494 | 0.489377 | 0.330556 | 0.570641 | 0.462858 | 0.245418 | 0.102597 | 0.53756 | 0.38267 | 0.35124 | 0.357565 | 0.287254 | 0.290834 |
| TCGA-AZ-6603-11A-02R-1839-07 | 0.179013 | 0.391302 | 0.515235 | 0.401734 | 0.444967 | 0.535783 | 0.682772 | 0.565127 | 0.434134 | 0.459931 | 0.132654 | 0.567385 | 0.250043 | 0.527087 | 0.377291 | 0.383914 | 0.638473 | 0.252671 | 0.567498 | 0.525153 | 0.307015 | 0.200838 | 0.586886 | 0.540536 | 0.40431 | 0.383068 | 0.23767 | 0.369464 |
| TCGA-AA-3713-11A-01R-1723-07 | 0.264152 | 0.408871 | 0.551826 | 0.37636 | 0.432295 | 0.551685 | 0.638429 | 0.52877 | 0.394275 | 0.375737 | 0.230863 | 0.548636 | 0.268632 | 0.517911 | 0.306364 | 0.372297 | 0.582686 | 0.278962 | 0.570646 | 0.449515 | 0.306537 | 0.224983 | 0.519859 | 0.444193 | 0.344564 | 0.389703 | 0.250412 | 0.311443 |
| TCGA-AF-5654-11A-11R-1660-07 | 0.220877 | 0.377625 | 0.406335 | 0.325842 | 0.435881 | 0.563875 | 0.619091 | 0.492407 | 0.362873 | 0.279642 | 0.146603 | 0.479343 | 0.215347 | 0.459333 | 0.223273 | 0.231114 | 0.410949 | 0.314688 | 0.56897 | 0.416941 | 0.217809 | 0.149258 | 0.507178 | 0.334319 | 0.301733 | 0.335423 | 0.241114 | 0.316587 |
| TCGA-AF-2689-11A-01R-A32Z-07 | 0.434613 | 0.425393 | 0.520921 | 0.36398 | 0.440684 | 0.559456 | 0.676799 | 0.517591 | 0.405022 | 0.417708 | 0.230253 | 0.500617 | 0.388571 | 0.473474 | 0.289202 | 0.370799 | 0.525672 | 0.358496 | 0.583543 | 0.505385 | 0.265171 | 0.135181 | 0.553558 | 0.452206 | 0.38942 | 0.398278 | 0.261333 | 0.348291 |
| TCGA-A6-5662-11A-01R-1653-07 | 0.310267 | 0.404509 | 0.485969 | 0.358582 | 0.431793 | 0.54762 | 0.673257 | 0.530664 | 0.448085 | 0.389166 | 0.251008 | 0.512459 | 0.317529 | 0.490021 | 0.309634 | 0.384495 | 0.543523 | 0.339686 | 0.555543 | 0.502616 | 0.278637 | 0.290517 | 0.567132 | 0.440812 | 0.354191 | 0.397841 | 0.247303 | 0.361244 |
| TCGA-AA-3511-11A-01R-1839-07 | 0.555016 | 0.452 | 0.521298 | 0.386838 | 0.445987 | 0.561291 | 0.667191 | 0.504344 | 0.417063 | 0.387958 | 0.185046 | 0.501373 | 0.497543 | 0.463323 | 0.289005 | 0.285108 | 0.52689 | 0.399848 | 0.603266 | 0.453281 | 0.235557 | 0.104493 | 0.543466 | 0.421972 | 0.386621 | 0.37782 | 0.2695 | 0.306609 |
| TCGA-AA-3662-11A-01R-1723-07 | 0.285436 | 0.327899 | 0.498466 | 0.362037 | 0.462293 | 0.54642 | 0.649713 | 0.524751 | 0.372137 | 0.40432 | 0.132349 | 0.5166 | 0.305584 | 0.488344 | 0.307514 | 0.360481 | 0.522894 | 0.28161 | 0.608756 | 0.472399 | 0.245145 | 0.093444 | 0.552234 | 0.434237 | 0.381965 | 0.358778 | 0.246064 | 0.27924 |
| TCGA-AA-3520-11A-01R-A32Z-07 | 0.360139 | 0.380521 | 0.508881 | 0.389641 | 0.437649 | 0.577017 | 0.651287 | 0.518597 | 0.364482 | 0.399319 | 0.207429 | 0.536368 | 0.347423 | 0.482708 | 0.288934 | 0.333673 | 0.514374 | 0.337931 | 0.587389 | 0.456626 | 0.269788 | 0.130582 | 0.530965 | 0.450613 | 0.371263 | 0.382561 | 0.284934 | 0.288731 |
| TCGA-A6-2682-11A-01R-A32Z-07 | 0.429293 | 0.391497 | 0.473014 | 0.404989 | 0.446865 | 0.581227 | 0.656517 | 0.545384 | 0.365934 | 0.376834 | 0.258151 | 0.52528 | 0.389372 | 0.494262 | 0.31159 | 0.346958 | 0.562429 | 0.343218 | 0.590218 | 0.443965 | 0.259592 | 0.193764 | 0.54806 | 0.4256 | 0.372653 | 0.368325 | 0.281562 | 0.29095 |
| TCGA-AA-3522-11A-01R-A32Z-07 | 0.329961 | 0.372904 | 0.471067 | 0.367796 | 0.431169 | 0.5771 | 0.648698 | 0.516358 | 0.373454 | 0.379163 | 0.159921 | 0.526466 | 0.321726 | 0.472378 | 0.27171 | 0.319596 | 0.51336 | 0.338451 | 0.570673 | 0.45825 | 0.269837 | 0.134954 | 0.535637 | 0.419394 | 0.361635 | 0.364089 | 0.255581 | 0.281001 |
| TCGA-AZ-6601-11A-01R-1774-07 | 0.356444 | 0.390919 | 0.465487 | 0.341777 | 0.418587 | 0.567933 | 0.660616 | 0.508582 | 0.385333 | 0.358749 | 0.160492 | 0.501079 | 0.313326 | 0.460298 | 0.239785 | 0.263847 | 0.503138 | 0.317179 | 0.563773 | 0.491811 | 0.262931 | 0.090725 | 0.546734 | 0.376004 | 0.340301 | 0.368507 | 0.264297 | 0.312622 |
| TCGA-A6-5665-11A-01R-1653-07 | 0.36374 | 0.392391 | 0.470786 | 0.342471 | 0.419389 | 0.588879 | 0.640343 | 0.526211 | 0.374214 | 0.388809 | 0.205495 | 0.51757 | 0.350908 | 0.461928 | 0.269939 | 0.327344 | 0.53115 | 0.310462 | 0.557405 | 0.444356 | 0.254629 | 0.246641 | 0.526184 | 0.421409 | 0.353221 | 0.356563 | 0.250984 | 0.283594 |
| TCGA-AA-3514-11A-01R-A32Z-07 | 0.304473 | 0.471443 | 0.572941 | 0.421381 | 0.44374 | 0.573786 | 0.653782 | 0.535814 | 0.382138 | 0.445762 | 0.15542 | 0.516603 | 0.337173 | 0.481267 | 0.303584 | 0.348643 | 0.584916 | 0.32527 | 0.571492 | 0.49006 | 0.273805 | 0.161753 | 0.522629 | 0.44355 | 0.360892 | 0.380227 | 0.275584 | 0.322752 |
| TCGA-AF-2692-11A-01R-A32Z-07 | 0.233999 | 0.422991 | 0.439302 | 0.391262 | 0.417354 | 0.534198 | 0.670745 | 0.550945 | 0.418066 | 0.39083 | 0.267898 | 0.494622 | 0.280465 | 0.509224 | 0.304086 | 0.395369 | 0.539536 | 0.294961 | 0.551545 | 0.52281 | 0.282578 | 0.210508 | 0.571876 | 0.477408 | 0.381875 | 0.383954 | 0.266506 | 0.381733 |
| TCGA-AG-3732-11A-01R-1660-07 | 0.214423 | 0.293725 | 0.388135 | 0.303661 | 0.427864 | 0.493329 | 0.661468 | 0.521038 | 0.438911 | 0.379751 | 0.12567 | 0.489167 | 0.258775 | 0.489654 | 0.285093 | 0.349561 | 0.433051 | 0.304612 | 0.528963 | 0.516014 | 0.263989 | 0.039693 | 0.556427 | 0.454638 | 0.373395 | 0.36501 | 0.190787 | 0.347938 |
| TCGA-AA-3534-11A-01R-A32Z-07 | 0.45068 | 0.399797 | 0.524015 | 0.398518 | 0.447172 | 0.598739 | 0.671311 | 0.535074 | 0.355483 | 0.407204 | 0.173861 | 0.532369 | 0.409751 | 0.494772 | 0.314979 | 0.317433 | 0.554206 | 0.345908 | 0.583201 | 0.444115 | 0.275512 | 0.124925 | 0.547948 | 0.456004 | 0.380992 | 0.38512 | 0.272255 | 0.280901 |
| TCGA-AF-3400-11A-01R-A32Z-07 | 0.192047 | 0.347433 | 0.42718 | 0.33046 | 0.426615 | 0.567118 | 0.610276 | 0.503338 | 0.350866 | 0.308297 | 0.109226 | 0.456633 | 0.200903 | 0.44351 | 0.201312 | 0.246896 | 0.418996 | 0.292327 | 0.55922 | 0.419594 | 0.235326 | 0.084116 | 0.510883 | 0.29752 | 0.303995 | 0.330462 | 0.254934 | 0.290205 |
| TCGA-A6-2684-11A-01R-A32Z-07 | 0.327568 | 0.372752 | 0.507633 | 0.403056 | 0.44041 | 0.592928 | 0.646482 | 0.524735 | 0.316408 | 0.393598 | 0.197321 | 0.527608 | 0.314167 | 0.479071 | 0.282319 | 0.331847 | 0.533844 | 0.29584 | 0.586139 | 0.442739 | 0.259672 | 0.183852 | 0.534702 | 0.41424 | 0.360701 | 0.357968 | 0.290654 | 0.271767 |
| TCGA-A6-2686-11A-01R-A32Z-07 | 0.237995 | 0.358158 | 0.491214 | 0.386812 | 0.436956 | 0.573645 | 0.638707 | 0.547585 | 0.354481 | 0.354744 | 0.162743 | 0.518401 | 0.263208 | 0.475962 | 0.2816 | 0.324638 | 0.52465 | 0.312 | 0.560166 | 0.457256 | 0.257497 | 0.216428 | 0.540995 | 0.418994 | 0.358669 | 0.36098 | 0.266968 | 0.279024 |
| TCGA-F4-6704-11A-01R-1839-07 | 0.203485 | 0.405154 | 0.401524 | 0.380715 | 0.416512 | 0.528059 | 0.644695 | 0.542664 | 0.435655 | 0.340131 | 0.247808 | 0.50536 | 0.224644 | 0.473024 | 0.262475 | 0.36044 | 0.484699 | 0.297389 | 0.545863 | 0.47422 | 0.269364 | 0.216447 | 0.545892 | 0.376197 | 0.325468 | 0.355761 | 0.239403 | 0.344191 |
| TCGA-AA-3518-11A-01R-1672-07 | 0.288669 | 0.315518 | 0.503644 | 0.33382 | 0.444063 | 0.584931 | 0.655332 | 0.52909 | 0.370192 | 0.38256 | 0.149432 | 0.51733 | 0.280161 | 0.48533 | 0.283156 | 0.314114 | 0.511297 | 0.291694 | 0.580629 | 0.472517 | 0.257691 | 0.181207 | 0.544425 | 0.403589 | 0.356206 | 0.375285 | 0.266798 | 0.272035 |
| TCGA-A6-2671-11A-01R-A32Z-07 | 0.24887 | 0.320821 | 0.487316 | 0.366927 | 0.426119 | 0.576753 | 0.650521 | 0.510454 | 0.388682 | 0.379263 | 0.171668 | 0.513696 | 0.246345 | 0.460054 | 0.240969 | 0.28164 | 0.488567 | 0.278435 | 0.565036 | 0.478768 | 0.255204 | 0.161338 | 0.535744 | 0.385966 | 0.34665 | 0.370551 | 0.270146 | 0.294764 |
| TCGA-AZ-6599-11A-01R-1774-07 | 0.177201 | 0.33836 | 0.422752 | 0.343215 | 0.434049 | 0.582433 | 0.659595 | 0.547571 | 0.375213 | 0.346847 | 0.181281 | 0.509846 | 0.172973 | 0.482511 | 0.291153 | 0.314696 | 0.527535 | 0.291681 | 0.546158 | 0.484294 | 0.270939 | 0.148304 | 0.550839 | 0.434825 | 0.341524 | 0.3526 | 0.26878 | 0.300324 |
| TCGA-AA-3712-11A-01R-1723-07 | 0.297317 | 0.33951 | 0.455693 | 0.365437 | 0.43317 | 0.579255 | 0.661145 | 0.515134 | 0.365166 | 0.383495 | 0.240162 | 0.525394 | 0.299305 | 0.480888 | 0.314226 | 0.315623 | 0.542579 | 0.310895 | 0.567579 | 0.44681 | 0.249999 | 0.146187 | 0.542856 | 0.427517 | 0.356622 | 0.348381 | 0.255071 | 0.283684 |
| TCGA-AA-3697-11A-01R-1723-07 | 0.367159 | 0.466857 | 0.511766 | 0.434687 | 0.45234 | 0.577037 | 0.669727 | 0.549545 | 0.426809 | 0.433162 | 0.215127 | 0.538877 | 0.346288 | 0.520878 | 0.337771 | 0.395476 | 0.613547 | 0.307702 | 0.576955 | 0.503071 | 0.310339 | 0.233831 | 0.574844 | 0.512666 | 0.394894 | 0.399236 | 0.310209 | 0.354128 |
| TCGA-DC-4745-01A-01R-A32Z-07 | -0.0046 | 0.466711 | 0.455618 | 0.352393 | 0.466238 | 0.553766 | 0.606703 | 0.489582 | 0.319257 | 0.276021 | 0.141062 | 0.487905 | 0.11134 | 0.469303 | 0.212748 | 0.190147 | 0.416757 | 0.282256 | 0.534792 | 0.427757 | 0.245259 | 0.023384 | 0.49356 | 0.35793 | 0.303018 | 0.32543 | 0.247187 | 0.315712 |
| TCGA-DY-A1DC-01A-31R-A155-07 | 0.031769 | 0.43863 | 0.43944 | 0.360022 | 0.492084 | 0.525025 | 0.617873 | 0.468672 | 0.320122 | 0.282683 | 0.143138 | 0.472858 | 0.151204 | 0.464889 | 0.186997 | 0.127164 | 0.396753 | 0.282356 | 0.532796 | 0.444576 | 0.2714 | -0.0889 | 0.471034 | 0.321317 | 0.298422 | 0.324954 | 0.273709 | 0.280675 |
| TCGA-AG-3732-01A-11R-1660-07 | 0.381757 | 0.499272 | 0.534129 | 0.345166 | 0.438316 | 0.581228 | 0.65415 | 0.493728 | 0.320396 | 0.362787 | 0.166978 | 0.426429 | 0.366308 | 0.449625 | 0.22663 | 0.254159 | 0.48002 | 0.348701 | 0.530087 | 0.417828 | 0.22331 | 0.090951 | 0.548032 | 0.328385 | 0.337639 | 0.359526 | 0.268316 | 0.328438 |
| TCGA-AF-3911-01A-01R-1736-07 | -0.00082 | 0.424643 | 0.423257 | 0.361689 | 0.466262 | 0.532104 | 0.631302 | 0.484303 | 0.329205 | 0.306216 | 0.169179 | 0.510157 | 0.105044 | 0.502892 | 0.202458 | 0.218864 | 0.438221 | 0.279365 | 0.534595 | 0.449314 | 0.284424 | 0.073116 | 0.536727 | 0.368001 | 0.315476 | 0.326332 | 0.213989 | 0.273099 |
| TCGA-AH-6549-01A-11R-1830-07 | 0.011424 | 0.424272 | 0.366422 | 0.375709 | 0.433887 | 0.517556 | 0.639187 | 0.510431 | 0.339687 | 0.306574 | 0.146077 | 0.47828 | 0.067418 | 0.493832 | 0.207753 | 0.193336 | 0.444281 | 0.269901 | 0.503952 | 0.459992 | 0.269043 | 0.082721 | 0.543124 | 0.414041 | 0.318468 | 0.303055 | 0.235417 | 0.339552 |
| TCGA-AG-3887-01A-01R-1119-07 | 0.04408 | 0.451272 | 0.52143 | 0.341548 | 0.458201 | 0.546285 | 0.642857 | 0.482547 | 0.274449 | 0.31904 | 0.085979 | 0.446604 | 0.096683 | 0.504772 | 0.228349 | 0.132724 | 0.45912 | 0.222962 | 0.5495 | 0.429154 | 0.250611 | 0.042528 | 0.488881 | 0.309229 | 0.280287 | 0.354661 | 0.317574 | 0.328626 |
| TCGA-DY-A1DD-01A-21R-A155-07 | -0.09651 | 0.273052 | 0.390983 | 0.264011 | 0.437861 | 0.545499 | 0.63476 | 0.421684 | 0.287996 | 0.188766 | 0.027446 | 0.424649 | -0.03132 | 0.447834 | 0.147225 | 0.050665 | 0.278651 | 0.285015 | 0.530617 | 0.400075 | 0.221353 | -0.02872 | 0.509984 | 0.223507 | 0.229665 | 0.242968 | 0.187002 | 0.236664 |
| TCGA-EI-6881-01A-11R-A32Z-07 | 0.018856 | 0.464917 | 0.42479 | 0.377566 | 0.45915 | 0.52453 | 0.656081 | 0.465533 | 0.31718 | 0.292959 | 0.1549 | 0.479162 | 0.142974 | 0.498809 | 0.184349 | 0.211736 | 0.413092 | 0.270792 | 0.570975 | 0.426078 | 0.251956 | 0.184634 | 0.510396 | 0.346097 | 0.279555 | 0.330132 | 0.280296 | 0.346421 |
| TCGA-AF-5654-01A-01R-1660-07 | -0.10035 | 0.416643 | 0.395756 | 0.327321 | 0.448506 | 0.515218 | 0.59527 | 0.458542 | 0.312909 | 0.19213 | 0.11835 | 0.432734 | 0.023675 | 0.441561 | 0.135984 | 0.157172 | 0.325678 | 0.273968 | 0.51778 | 0.378353 | 0.202153 | 0.023015 | 0.516411 | 0.307166 | 0.234115 | 0.296911 | 0.282469 | 0.299139 |
| TCGA-AH-6544-01A-11R-1830-07 | 0.065075 | 0.389399 | 0.335814 | 0.254717 | 0.452993 | 0.518482 | 0.571262 | 0.460801 | 0.28724 | 0.209694 | -0.00475 | 0.430825 | 0.115842 | 0.458166 | 0.082923 | 0.021265 | 0.279026 | 0.266953 | 0.545424 | 0.356312 | 0.194241 | 0.045222 | 0.465239 | 0.147433 | 0.203273 | 0.272404 | 0.221749 | 0.316773 |
| TCGA-DY-A1H8-01A-21R-A155-07 | -0.16306 | 0.389424 | 0.403308 | 0.276981 | 0.446971 | 0.563033 | 0.59494 | 0.405715 | 0.232487 | 0.196842 | 0.060036 | 0.401026 | -0.04165 | 0.404898 | 0.068406 | 0.037901 | 0.324148 | 0.201646 | 0.544783 | 0.325013 | 0.183545 | -0.05942 | 0.462543 | 0.198144 | 0.204368 | 0.218126 | 0.209728 | 0.243816 |
| TCGA-EI-6514-01A-11R-1736-07 | -0.02725 | 0.312219 | 0.39676 | 0.344505 | 0.474507 | 0.535706 | 0.646012 | 0.50301 | 0.340913 | 0.27216 | 0.072319 | 0.509808 | 0.107522 | 0.444652 | 0.197853 | 0.184865 | 0.408793 | 0.306662 | 0.547829 | 0.469319 | 0.243741 | 0.043169 | 0.536609 | 0.37696 | 0.30695 | 0.314413 | 0.225997 | 0.246909 |
| TCGA-AG-4001-01A-02R-1119-07 | 0.009178 | 0.36828 | 0.440916 | 0.398044 | 0.464305 | 0.600803 | 0.662289 | 0.538983 | 0.337338 | 0.342162 | 0.110455 | 0.494407 | 0.129279 | 0.469846 | 0.248763 | 0.201462 | 0.519088 | 0.306582 | 0.558973 | 0.474534 | 0.273752 | 0.003083 | 0.564698 | 0.440511 | 0.338638 | 0.349419 | 0.288166 | 0.221411 |
| TCGA-EI-6917-01A-11R-1928-07 | 0.13665 | 0.534061 | 0.516921 | 0.449415 | 0.454729 | 0.566274 | 0.676123 | 0.57505 | 0.368791 | 0.451278 | 0.123226 | 0.556096 | 0.249263 | 0.490079 | 0.315822 | 0.261347 | 0.643535 | 0.28254 | 0.544799 | 0.487958 | 0.32667 | 0.049957 | 0.528462 | 0.584365 | 0.384652 | 0.392886 | 0.217954 | 0.372076 |
| TCGA-AG-3608-01A-01R-0826-07 | 0.079807 | 0.446117 | 0.527011 | 0.395878 | 0.454938 | 0.581926 | 0.626121 | 0.50309 | 0.290687 | 0.348313 | 0.122147 | 0.515685 | 0.179029 | 0.467473 | 0.237627 | 0.26737 | 0.487641 | 0.244524 | 0.564937 | 0.435284 | 0.248512 | 0.142186 | 0.521632 | 0.392088 | 0.348115 | 0.343663 | 0.299494 | 0.269568 |
| TCGA-DC-6160-01A-11R-1660-07 | 0.097847 | 0.509721 | 0.463761 | 0.392923 | 0.44781 | 0.512323 | 0.638752 | 0.499597 | 0.34524 | 0.345623 | 0.143956 | 0.47331 | 0.204283 | 0.484948 | 0.208156 | 0.198261 | 0.457765 | 0.305342 | 0.549198 | 0.415985 | 0.239465 | 0.143056 | 0.50768 | 0.363128 | 0.30454 | 0.351497 | 0.296122 | 0.325228 |
| TCGA-EI-7002-01A-11R-1928-07 | 0.007156 | 0.331547 | 0.391264 | 0.36677 | 0.469406 | 0.550866 | 0.648266 | 0.502687 | 0.323548 | 0.316207 | 0.094216 | 0.471362 | 0.109936 | 0.4468 | 0.195399 | 0.177846 | 0.443633 | 0.268429 | 0.522879 | 0.465923 | 0.224197 | -0.01066 | 0.533105 | 0.397685 | 0.293528 | 0.333074 | 0.21952 | 0.259974 |
| TCGA-AG-3999-01A-01R-1119-07 | -0.05929 | 0.350952 | 0.343789 | 0.336774 | 0.462193 | 0.585792 | 0.648428 | 0.474147 | 0.28995 | 0.266499 | 0.086515 | 0.475053 | 0.103782 | 0.493613 | 0.235161 | 0.189748 | 0.416039 | 0.232247 | 0.534132 | 0.411888 | 0.252579 | 0.076395 | 0.512649 | 0.372107 | 0.323636 | 0.319303 | 0.250552 | 0.260623 |
| TCGA-AG-A002-01A-01R-A002-07 | -0.20584 | 0.347055 | 0.325144 | 0.263079 | 0.442964 | 0.529612 | 0.607247 | 0.481751 | 0.301839 | 0.212724 | 0.009384 | 0.421051 | 0.043346 | 0.530842 | 0.128013 | 0.122291 | 0.358505 | 0.202458 | 0.553992 | 0.3671 | 0.199736 | -0.01648 | 0.478209 | 0.294036 | 0.2178 | 0.251785 | 0.249684 | 0.403514 |
| TCGA-BM-6198-01A-11R-1736-07 | 0.064934 | 0.52465 | 0.532021 | 0.418294 | 0.467852 | 0.523935 | 0.672946 | 0.541748 | 0.344423 | 0.382668 | 0.196808 | 0.520479 | 0.187977 | 0.500751 | 0.294784 | 0.272029 | 0.566842 | 0.220184 | 0.538175 | 0.455693 | 0.316894 | 0.178261 | 0.541025 | 0.401733 | 0.331126 | 0.375675 | 0.290238 | 0.334399 |
| TCGA-F5-6864-01A-11R-1928-07 | 0.03052 | 0.370355 | 0.444667 | 0.400995 | 0.443893 | 0.545559 | 0.665487 | 0.50536 | 0.354746 | 0.366182 | 0.106251 | 0.491004 | 0.118834 | 0.49414 | 0.272604 | 0.207817 | 0.509003 | 0.308159 | 0.528028 | 0.501391 | 0.273611 | 0.009549 | 0.533885 | 0.411935 | 0.329646 | 0.336145 | 0.255158 | 0.271437 |
| TCGA-AG-3583-01A-01R-0821-07 | 0.073609 | 0.439567 | 0.481984 | 0.354209 | 0.449696 | 0.566963 | 0.634121 | 0.482876 | 0.328658 | 0.288886 | 0.167981 | 0.502349 | 0.151712 | 0.488968 | 0.189212 | 0.195246 | 0.48219 | 0.286921 | 0.581631 | 0.398207 | 0.213287 | 0.189502 | 0.495293 | 0.330793 | 0.289879 | 0.347072 | 0.320058 | 0.294746 |
| TCGA-CK-4950-01A-01R-1723-07 | 0.214852 | 0.501882 | 0.517415 | 0.41781 | 0.457272 | 0.591096 | 0.669705 | 0.541694 | 0.32983 | 0.378934 | 0.197602 | 0.527755 | 0.271915 | 0.494254 | 0.277231 | 0.321168 | 0.526718 | 0.295917 | 0.570922 | 0.454825 | 0.286364 | 0.203638 | 0.530334 | 0.396888 | 0.35726 | 0.371675 | 0.316441 | 0.342711 |
| TCGA-G4-6294-01A-11R-1774-07 | -0.13711 | 0.381187 | 0.40326 | 0.344316 | 0.481642 | 0.532664 | 0.631084 | 0.422736 | 0.273731 | 0.279202 | 0.119522 | 0.417997 | 0.05382 | 0.481834 | 0.149714 | 0.027223 | 0.366804 | 0.293155 | 0.547167 | 0.420486 | 0.223971 | -0.07628 | 0.499204 | 0.289654 | 0.267293 | 0.26587 | 0.223691 | 0.296306 |
| TCGA-A6-3807-01A-01R-1022-07 | 0.054259 | 0.441718 | 0.455066 | 0.416008 | 0.444564 | 0.541272 | 0.6413 | 0.521732 | 0.301591 | 0.363761 | 0.160566 | 0.530638 | 0.167307 | 0.523611 | 0.257204 | 0.215732 | 0.502754 | 0.242756 | 0.517535 | 0.444527 | 0.27849 | 0.193718 | 0.535232 | 0.415082 | 0.31963 | 0.341941 | 0.286901 | 0.249823 |
| TCGA-AA-3846-01A-01R-1022-07 | 0.05763 | 0.48787 | 0.465613 | 0.378331 | 0.465416 | 0.51588 | 0.650998 | 0.499185 | 0.294328 | 0.339718 | 0.190907 | 0.51122 | 0.187878 | 0.501687 | 0.223605 | 0.219042 | 0.470165 | 0.299293 | 0.563494 | 0.428892 | 0.252659 | 0.085127 | 0.5123 | 0.390312 | 0.295797 | 0.329508 | 0.291951 | 0.293703 |
| TCGA-F4-6703-01A-11R-1839-07 | 0.20253 | 0.514389 | 0.587422 | 0.466719 | 0.524106 | 0.549247 | 0.72735 | 0.635613 | 0.426171 | 0.507903 | 0.147688 | 0.589025 | 0.308202 | 0.588247 | 0.438457 | 0.335022 | 0.717273 | 0.29493 | 0.57682 | 0.539841 | 0.393608 | 0.248277 | 0.576815 | 0.627051 | 0.481914 | 0.459821 | 0.238517 | 0.40073 |
| TCGA-AZ-4313-01A-01R-1410-07 | -0.18953 | 0.369735 | 0.431501 | 0.322696 | 0.427887 | 0.599527 | 0.62707 | 0.463141 | 0.271119 | 0.290544 | 0.047025 | 0.454272 | 0.004232 | 0.456633 | 0.152976 | 0.094894 | 0.414986 | 0.201088 | 0.543564 | 0.39389 | 0.202963 | 0.000403 | 0.514311 | 0.233759 | 0.265418 | 0.279206 | 0.300781 | 0.318471 |
| TCGA-F4-6461-01A-11R-1774-07 | 0.379956 | 0.408423 | 0.450888 | 0.335368 | 0.452124 | 0.549016 | 0.678386 | 0.495063 | 0.295919 | 0.354163 | 0.155107 | 0.484469 | 0.350428 | 0.448255 | 0.230575 | 0.202389 | 0.506063 | 0.318872 | 0.564467 | 0.454721 | 0.238747 | -0.0138 | 0.504891 | 0.340124 | 0.306416 | 0.342992 | 0.266975 | 0.330831 |
| TCGA-AA-3811-01A-01R-1022-07 | 0.110305 | 0.444565 | 0.486265 | 0.344453 | 0.398136 | 0.548925 | 0.646175 | 0.502452 | 0.300547 | 0.351028 | 0.171471 | 0.523102 | 0.212651 | 0.471208 | 0.240796 | 0.265401 | 0.510142 | 0.255541 | 0.528049 | 0.441317 | 0.255076 | 0.101971 | 0.482426 | 0.373049 | 0.327785 | 0.312981 | 0.268944 | 0.343649 |
| TCGA-G4-6303-01A-11R-1774-07 | 0.017732 | 0.405224 | 0.413279 | 0.356288 | 0.451833 | 0.533097 | 0.637522 | 0.526927 | 0.338032 | 0.320489 | 0.125227 | 0.478602 | 0.133301 | 0.457601 | 0.211896 | 0.193839 | 0.440142 | 0.31412 | 0.52657 | 0.456476 | 0.267936 | 0.078145 | 0.518581 | 0.370125 | 0.314544 | 0.33537 | 0.230859 | 0.276305 |
| TCGA-QG-A5Z2-01A-11R-A28H-07 | 0.322014 | 0.487171 | 0.521313 | 0.372722 | 0.44375 | 0.524384 | 0.664212 | 0.463907 | 0.30501 | 0.390064 | 0.184348 | 0.45445 | 0.342663 | 0.431888 | 0.187247 | 0.069268 | 0.44872 | 0.313952 | 0.544503 | 0.399753 | 0.23606 | -0.04371 | 0.471266 | 0.269787 | 0.313752 | 0.319098 | 0.296159 | 0.343711 |
| TCGA-CA-5256-01A-01R-1410-07 | 0.059629 | 0.464286 | 0.545428 | 0.352921 | 0.476992 | 0.540691 | 0.599826 | 0.44094 | 0.264363 | 0.293268 | 0.139703 | 0.47779 | 0.116644 | 0.422447 | 0.207946 | 0.163817 | 0.416028 | 0.289038 | 0.50977 | 0.422766 | 0.185597 | 0.00526 | 0.464828 | 0.330887 | 0.306789 | 0.351104 | 0.242919 | 0.301975 |
| TCGA-G4-6317-01A-11R-1723-07 | -0.15906 | 0.342792 | 0.42357 | 0.221066 | 0.436814 | 0.538444 | 0.549683 | 0.412379 | 0.218711 | 0.142072 | 0.008444 | 0.388791 | -0.07877 | 0.433902 | 0.017939 | 0.078042 | 0.179703 | 0.234859 | 0.539515 | 0.335997 | 0.135135 | -0.01989 | 0.489716 | 0.171799 | 0.202221 | 0.252339 | 0.234376 | 0.209339 |
| TCGA-AA-3488-01A-01R-1410-07 | 0.015979 | 0.364776 | 0.416671 | 0.338291 | 0.472865 | 0.581005 | 0.61722 | 0.498872 | 0.275365 | 0.259527 | 0.05818 | 0.481889 | 0.089447 | 0.475272 | 0.218119 | 0.214989 | 0.438971 | 0.205761 | 0.557036 | 0.377442 | 0.231805 | 0.137509 | 0.501585 | 0.33671 | 0.330329 | 0.323047 | 0.29873 | 0.299466 |
| TCGA-CM-5341-01A-01R-1410-07 | 0.023661 | 0.476145 | 0.560425 | 0.426686 | 0.473386 | 0.514076 | 0.659969 | 0.526314 | 0.376586 | 0.378971 | 0.125722 | 0.578436 | 0.193418 | 0.512153 | 0.329197 | 0.272105 | 0.588327 | 0.233411 | 0.548629 | 0.46352 | 0.291553 | 0.104409 | 0.5113 | 0.499169 | 0.370792 | 0.398956 | 0.262129 | 0.33393 |
| TCGA-F4-6854-01A-11R-1928-07 | 0.057616 | 0.402555 | 0.452612 | 0.367771 | 0.464219 | 0.570773 | 0.641032 | 0.507552 | 0.28885 | 0.313313 | 0.104092 | 0.497075 | 0.126675 | 0.45983 | 0.181607 | 0.179491 | 0.419792 | 0.298034 | 0.564956 | 0.468291 | 0.257637 | 0.048402 | 0.544149 | 0.383117 | 0.310732 | 0.338894 | 0.2347 | 0.307668 |
| TCGA-AA-3947-01A-01R-1022-07 | -0.00332 | 0.571439 | 0.473687 | 0.375356 | 0.45938 | 0.425421 | 0.6275 | 0.528123 | 0.323669 | 0.34072 | 0.23168 | 0.511299 | 0.207872 | 0.50816 | 0.271541 | 0.236435 | 0.532455 | 0.273098 | 0.453255 | 0.450707 | 0.272924 | 0.221475 | 0.507923 | 0.433171 | 0.321504 | 0.329202 | 0.263652 | 0.435613 |
| TCGA-CM-6162-01A-11R-1653-07 | 0.155429 | 0.517296 | 0.498721 | 0.436062 | 0.449558 | 0.557075 | 0.704835 | 0.638519 | 0.384199 | 0.453907 | 0.269711 | 0.560749 | 0.266493 | 0.581306 | 0.385799 | 0.382761 | 0.652123 | 0.318393 | 0.569393 | 0.535097 | 0.348193 | 0.234055 | 0.582325 | 0.563533 | 0.405568 | 0.431284 | 0.309597 | 0.412506 |
| TCGA-AA-3662-01A-01R-1723-07 | 0.078284 | 0.429358 | 0.438466 | 0.402593 | 0.468375 | 0.558863 | 0.660948 | 0.509795 | 0.299757 | 0.374961 | 0.11933 | 0.513031 | 0.191602 | 0.513911 | 0.256045 | 0.254853 | 0.504909 | 0.290364 | 0.541762 | 0.440699 | 0.25669 | 0.178092 | 0.541952 | 0.41672 | 0.347655 | 0.332862 | 0.263535 | 0.294999 |
| TCGA-A6-2678-01A-01R-0821-07 | 0.022347 | 0.478585 | 0.490319 | 0.377912 | 0.471773 | 0.529468 | 0.642747 | 0.520758 | 0.294291 | 0.359355 | 0.12567 | 0.516674 | 0.151237 | 0.515154 | 0.210888 | 0.123278 | 0.488888 | 0.275438 | 0.543735 | 0.448938 | 0.278443 | 0.005564 | 0.518075 | 0.395936 | 0.315203 | 0.359045 | 0.305338 | 0.307236 |
| TCGA-CM-6675-01A-11R-1839-07 | -0.13756 | 0.415716 | 0.403595 | 0.351914 | 0.443574 | 0.578447 | 0.618675 | 0.508044 | 0.293129 | 0.355169 | 0.010288 | 0.456683 | 0.071646 | 0.470031 | 0.156267 | 0.176829 | 0.406674 | 0.284585 | 0.504614 | 0.444362 | 0.267778 | -0.07343 | 0.4704 | 0.304851 | 0.268676 | 0.292829 | 0.232837 | 0.355563 |
| TCGA-AY-4071-01A-01R-1113-07 | 0.185822 | 0.404904 | 0.542443 | 0.404468 | 0.453095 | 0.609211 | 0.67755 | 0.487589 | 0.295066 | 0.385898 | 0.211182 | 0.49733 | 0.25324 | 0.467578 | 0.261534 | 0.200928 | 0.520754 | 0.294702 | 0.573442 | 0.468597 | 0.287917 | 0.13466 | 0.537312 | 0.410974 | 0.356802 | 0.379992 | 0.319735 | 0.257987 |
| TCGA-AA-3976-01A-01R-1022-07 | 0.028187 | 0.424519 | 0.455178 | 0.319902 | 0.474902 | 0.526544 | 0.634777 | 0.470188 | 0.305727 | 0.256092 | 0.084449 | 0.472027 | 0.122473 | 0.470452 | 0.184082 | 0.193114 | 0.425273 | 0.271617 | 0.534828 | 0.429535 | 0.228835 | 0.016022 | 0.514536 | 0.372478 | 0.281552 | 0.340413 | 0.321726 | 0.312442 |
| TCGA-CA-5797-01A-01R-1653-07 | 0.02393 | 0.391784 | 0.435886 | 0.350855 | 0.491552 | 0.547053 | 0.640128 | 0.491502 | 0.331752 | 0.30687 | 0.083726 | 0.498195 | 0.122108 | 0.488371 | 0.208217 | 0.26219 | 0.412514 | 0.269666 | 0.558774 | 0.441562 | 0.278435 | 0.027107 | 0.536041 | 0.368658 | 0.315789 | 0.32791 | 0.238604 | 0.295926 |
| TCGA-AA-A01R-01A-21R-A083-07 | 0.063792 | 0.461106 | 0.609603 | 0.400078 | 0.450243 | 0.608789 | 0.654057 | 0.496124 | 0.26349 | 0.47105 | 0.035103 | 0.512596 | 0.215154 | 0.453143 | 0.247708 | 0.156822 | 0.564793 | 0.224746 | 0.521825 | 0.454247 | 0.308159 | 0.042713 | 0.477226 | 0.430235 | 0.365753 | 0.366577 | 0.269958 | 0.294384 |
| TCGA-A6-5664-01A-21R-1839-07 | 0.162908 | 0.500914 | 0.502811 | 0.418124 | 0.455489 | 0.548085 | 0.673762 | 0.559629 | 0.411022 | 0.418515 | 0.219014 | 0.5252 | 0.23701 | 0.527405 | 0.321286 | 0.365755 | 0.573647 | 0.301762 | 0.573672 | 0.486373 | 0.318916 | 0.136943 | 0.582094 | 0.48955 | 0.376424 | 0.396849 | 0.334417 | 0.38969 |
| TCGA-AA-A02H-01A-01R-A089-07 | -0.07302 | 0.370996 | 0.415429 | 0.279322 | 0.449624 | 0.562529 | 0.640836 | 0.461734 | 0.244673 | 0.257009 | 0.050987 | 0.438675 | 0.084456 | 0.454757 | 0.123243 | 0.128679 | 0.380519 | 0.252922 | 0.52405 | 0.422662 | 0.198455 | -0.00583 | 0.478214 | 0.311262 | 0.242369 | 0.263126 | 0.185111 | 0.216828 |
| TCGA-AA-3862-01A-01R-1022-07 | 0.112044 | 0.507167 | 0.530471 | 0.399381 | 0.484096 | 0.552916 | 0.632692 | 0.525173 | 0.33988 | 0.365381 | 0.133068 | 0.525399 | 0.209483 | 0.502906 | 0.26154 | 0.298631 | 0.518257 | 0.272835 | 0.550946 | 0.430756 | 0.284325 | 0.177383 | 0.523642 | 0.372263 | 0.338395 | 0.374391 | 0.324184 | 0.341534 |
| TCGA-D5-7000-01A-11R-A32Z-07 | 0.057236 | 0.453102 | 0.466009 | 0.410855 | 0.446688 | 0.57806 | 0.656629 | 0.559694 | 0.329782 | 0.362959 | 0.14881 | 0.506686 | 0.168767 | 0.472516 | 0.235781 | 0.257441 | 0.495058 | 0.318178 | 0.552 | 0.470951 | 0.280373 | 0.144264 | 0.534791 | 0.405436 | 0.322651 | 0.340579 | 0.302631 | 0.333054 |
| TCGA-CA-6716-01A-11R-1839-07 | -0.08525 | 0.301954 | 0.348955 | 0.305743 | 0.471022 | 0.524696 | 0.632312 | 0.471582 | 0.321962 | 0.242941 | 0.073029 | 0.384873 | 0.032184 | 0.424116 | 0.143497 | 0.137111 | 0.375609 | 0.234037 | 0.520715 | 0.42706 | 0.174012 | -0.0491 | 0.476315 | 0.274301 | 0.231006 | 0.291479 | 0.248167 | 0.235234 |
| TCGA-AA-A01G-01A-01R-A002-07 | -0.00492 | 0.409799 | 0.423805 | 0.307549 | 0.473048 | 0.551526 | 0.62287 | 0.478958 | 0.29342 | 0.26924 | 0.136998 | 0.464465 | 0.046317 | 0.448403 | 0.14868 | 0.162799 | 0.357604 | 0.265698 | 0.511448 | 0.40484 | 0.202952 | -0.00263 | 0.463143 | 0.288315 | 0.247085 | 0.288597 | 0.265488 | 0.293834 |
| TCGA-D5-6535-01A-11R-1723-07 | 0.066294 | 0.498541 | 0.468629 | 0.412025 | 0.45692 | 0.539366 | 0.668231 | 0.546375 | 0.303899 | 0.399296 | 0.157236 | 0.494471 | 0.204311 | 0.459502 | 0.214872 | 0.213477 | 0.491296 | 0.300203 | 0.528444 | 0.422459 | 0.249319 | 0.114674 | 0.51599 | 0.377656 | 0.334737 | 0.366794 | 0.285203 | 0.329787 |
| TCGA-CK-6748-01A-11R-1839-07 | -0.03554 | 0.353198 | 0.421098 | 0.397256 | 0.461021 | 0.556137 | 0.664399 | 0.555869 | 0.359323 | 0.31189 | 0.190704 | 0.506314 | 0.115566 | 0.496627 | 0.298156 | 0.258438 | 0.527773 | 0.276385 | 0.539015 | 0.497206 | 0.283788 | 0.085465 | 0.556546 | 0.474398 | 0.310451 | 0.345772 | 0.249087 | 0.288437 |
| TCGA-D5-5539-01A-01R-1653-07 | 0.085492 | 0.42907 | 0.406337 | 0.373376 | 0.469302 | 0.552899 | 0.670663 | 0.538956 | 0.366446 | 0.346043 | 0.178955 | 0.509583 | 0.203324 | 0.514018 | 0.281872 | 0.248781 | 0.537526 | 0.300234 | 0.545153 | 0.45362 | 0.279287 | 0.126868 | 0.557011 | 0.437167 | 0.330287 | 0.33662 | 0.249796 | 0.350359 |
| TCGA-A6-4105-01A-02R-1774-07 | 0.079438 | 0.53007 | 0.542107 | 0.422574 | 0.476393 | 0.562891 | 0.659481 | 0.554137 | 0.363838 | 0.388665 | 0.20659 | 0.51627 | 0.166926 | 0.473928 | 0.273775 | 0.276232 | 0.563292 | 0.3139 | 0.550495 | 0.479252 | 0.303217 | 0.127813 | 0.54673 | 0.503409 | 0.363727 | 0.363113 | 0.303742 | 0.331405 |
| TCGA-G4-6307-01A-11R-1723-07 | 0.078262 | 0.321893 | 0.384666 | 0.307486 | 0.47364 | 0.541933 | 0.590781 | 0.376064 | 0.252261 | 0.185777 | 0.011863 | 0.402831 | 0.118153 | 0.411654 | 0.091501 | 0.116551 | 0.25357 | 0.281592 | 0.546748 | 0.346415 | 0.204456 | -0.07523 | 0.499457 | 0.179492 | 0.22609 | 0.25084 | 0.239504 | 0.225426 |
| TCGA-EI-6512-01A-11R-1736-07 | 0.013943 | 0.439415 | 0.428242 | 0.414381 | 0.451012 | 0.57894 | 0.644582 | 0.511758 | 0.334104 | 0.347209 | 0.119946 | 0.504252 | 0.102626 | 0.466513 | 0.232344 | 0.241565 | 0.464388 | 0.301655 | 0.566443 | 0.44216 | 0.251555 | 0.185081 | 0.536918 | 0.373081 | 0.324236 | 0.309706 | 0.286069 | 0.337315 |
| TCGA-AH-6897-01A-11R-1928-07 | -0.05323 | 0.434953 | 0.385203 | 0.323846 | 0.422307 | 0.511921 | 0.582115 | 0.43896 | 0.303894 | 0.241056 | 0.115409 | 0.445168 | 0.047069 | 0.448471 | 0.066188 | 0.121367 | 0.338821 | 0.311311 | 0.534935 | 0.381961 | 0.202256 | 0.009427 | 0.510566 | 0.296606 | 0.243802 | 0.290006 | 0.259154 | 0.283215 |
| TCGA-F5-6813-01A-11R-1830-07 | 0.15118 | 0.457139 | 0.494958 | 0.374405 | 0.484109 | 0.553554 | 0.642139 | 0.518488 | 0.36469 | 0.312182 | 0.107987 | 0.502915 | 0.177187 | 0.472023 | 0.236763 | 0.189726 | 0.489073 | 0.307319 | 0.531546 | 0.46215 | 0.28722 | 0.012544 | 0.473293 | 0.383947 | 0.324345 | 0.353967 | 0.199027 | 0.316934 |
| TCGA-AG-3591-01A-01R-1736-07 | 0.023374 | 0.44607 | 0.457208 | 0.368361 | 0.477626 | 0.569886 | 0.636378 | 0.478025 | 0.291841 | 0.322841 | 0.142679 | 0.47654 | 0.141797 | 0.500009 | 0.250177 | 0.193932 | 0.442467 | 0.268668 | 0.532349 | 0.404195 | 0.233023 | 0.072837 | 0.536459 | 0.317787 | 0.305744 | 0.324792 | 0.260504 | 0.398142 |
| TCGA-AG-3605-01A-01R-0826-07 | 0.061657 | 0.424121 | 0.484234 | 0.39903 | 0.457106 | 0.544563 | 0.612011 | 0.481299 | 0.261522 | 0.275904 | 0.056988 | 0.498602 | 0.113325 | 0.476937 | 0.212205 | 0.206661 | 0.460975 | 0.260699 | 0.564847 | 0.388026 | 0.203301 | 0.161648 | 0.531178 | 0.332752 | 0.302432 | 0.307998 | 0.329157 | 0.302373 |
| TCGA-A6-6137-01A-11R-1774-07 | 0.136737 | 0.438368 | 0.451713 | 0.395414 | 0.453847 | 0.549596 | 0.656512 | 0.507837 | 0.321733 | 0.34992 | 0.156725 | 0.478513 | 0.200705 | 0.473517 | 0.224574 | 0.246478 | 0.4609 | 0.333949 | 0.566983 | 0.426618 | 0.235858 | 0.08654 | 0.545154 | 0.359586 | 0.341136 | 0.354846 | 0.301844 | 0.282997 |
| TCGA-AA-3860-01A-02R-0905-07 | 0.182022 | 0.488082 | 0.526763 | 0.412996 | 0.47155 | 0.613429 | 0.644211 | 0.500142 | 0.313999 | 0.352024 | 0.132474 | 0.500855 | 0.250009 | 0.474955 | 0.272551 | 0.247755 | 0.528738 | 0.26012 | 0.545581 | 0.471137 | 0.287511 | 0.10495 | 0.523872 | 0.410821 | 0.32237 | 0.366827 | 0.284597 | 0.304218 |
| TCGA-AA-3986-01A-02R-1022-07 | 0.253214 | 0.492059 | 0.546372 | 0.422336 | 0.476849 | 0.5701 | 0.687016 | 0.528864 | 0.337357 | 0.39014 | 0.167495 | 0.50869 | 0.297699 | 0.530447 | 0.291076 | 0.238957 | 0.570313 | 0.31472 | 0.580413 | 0.45455 | 0.221295 | 0.190433 | 0.550263 | 0.434957 | 0.366073 | 0.387444 | 0.327004 | 0.311077 |
| TCGA-CM-6165-01A-11R-1653-07 | 0.078383 | 0.394818 | 0.421586 | 0.380237 | 0.467747 | 0.54006 | 0.645091 | 0.514409 | 0.385688 | 0.325791 | 0.135232 | 0.480861 | 0.174506 | 0.48065 | 0.261796 | 0.23618 | 0.475434 | 0.294836 | 0.567388 | 0.475419 | 0.263562 | 0.104239 | 0.541564 | 0.425273 | 0.334682 | 0.351811 | 0.230091 | 0.298382 |
| TCGA-CM-4750-01A-01R-1410-07 | 0.102761 | 0.404308 | 0.479965 | 0.36679 | 0.452018 | 0.579295 | 0.612029 | 0.460162 | 0.279915 | 0.276376 | 0.067739 | 0.469663 | 0.122037 | 0.446349 | 0.137504 | 0.140549 | 0.411803 | 0.273353 | 0.549975 | 0.426927 | 0.235153 | -0.04108 | 0.493772 | 0.333424 | 0.291696 | 0.326111 | 0.234781 | 0.221963 |
| TCGA-AA-3520-01A-01R-0821-07 | 0.105733 | 0.430709 | 0.429024 | 0.387751 | 0.473233 | 0.540988 | 0.655193 | 0.511387 | 0.327664 | 0.345509 | 0.139069 | 0.516432 | 0.184354 | 0.514158 | 0.265487 | 0.23125 | 0.515739 | 0.276261 | 0.556983 | 0.457694 | 0.262176 | 0.111574 | 0.534286 | 0.434883 | 0.346755 | 0.353419 | 0.3255 | 0.306813 |
| TCGA-G4-6625-01A-21R-1774-07 | 0.269269 | 0.507454 | 0.565112 | 0.412707 | 0.467545 | 0.567131 | 0.684716 | 0.538838 | 0.34978 | 0.429838 | 0.184334 | 0.521147 | 0.319614 | 0.478312 | 0.291212 | 0.278469 | 0.579546 | 0.315205 | 0.577322 | 0.483177 | 0.301668 | 0.041919 | 0.556173 | 0.496882 | 0.365601 | 0.386336 | 0.280998 | 0.31113 |
| TCGA-DM-A28M-01A-12R-A16W-07 | -0.25115 | 0.391211 | 0.393874 | 0.282935 | 0.460914 | 0.526798 | 0.577184 | 0.449011 | 0.273302 | 0.229082 | 0.0894 | 0.456358 | -0.07128 | 0.448038 | 0.097259 | 0.126397 | 0.33086 | 0.240698 | 0.532132 | 0.345766 | 0.185623 | 0.044624 | 0.486551 | 0.220764 | 0.224709 | 0.27351 | 0.296697 | 0.311779 |
| TCGA-D5-6920-01A-11R-1928-07 | 0.107071 | 0.471847 | 0.477324 | 0.402107 | 0.451039 | 0.560896 | 0.651725 | 0.521375 | 0.316924 | 0.340231 | 0.206331 | 0.50993 | 0.195187 | 0.510494 | 0.246088 | 0.289148 | 0.500016 | 0.295113 | 0.577206 | 0.448857 | 0.265221 | 0.25149 | 0.524126 | 0.390417 | 0.320623 | 0.353395 | 0.345799 | 0.311066 |
| TCGA-DM-A288-01A-11R-A16W-07 | -0.18608 | 0.33236 | 0.264442 | 0.266886 | 0.431256 | 0.529989 | 0.621837 | 0.428892 | 0.269461 | 0.203345 | 0.108033 | 0.413328 | 0.015944 | 0.392924 | 0.095282 | 0.075507 | 0.323379 | 0.184884 | 0.510315 | 0.351299 | 0.182322 | -0.0064 | 0.485123 | 0.24577 | 0.236729 | 0.235743 | 0.19824 | 0.270762 |
| TCGA-AA-A02O-01A-21R-A16W-07 | 0.00212 | 0.352585 | 0.464434 | 0.345533 | 0.453257 | 0.619718 | 0.643999 | 0.471498 | 0.275006 | 0.304285 | 0.104904 | 0.461204 | 0.021696 | 0.443843 | 0.170439 | 0.116676 | 0.423828 | 0.253201 | 0.565248 | 0.377827 | 0.201567 | 0.06656 | 0.502243 | 0.28751 | 0.279722 | 0.288177 | 0.347985 | 0.272091 |
| TCGA-AA-A02E-01A-01R-A00A-07 | -0.08459 | 0.385863 | 0.432308 | 0.311885 | 0.439102 | 0.544224 | 0.642791 | 0.437597 | 0.26397 | 0.308367 | 0.140596 | 0.429498 | 0.048282 | 0.457989 | 0.169417 | 0.107861 | 0.370264 | 0.213545 | 0.538822 | 0.39708 | 0.198902 | 0.08096 | 0.498612 | 0.272395 | 0.301586 | 0.300322 | 0.29318 | 0.3044 |
| TCGA-AA-3710-01A-01R-1022-07 | 0.153158 | 0.5618 | 0.613854 | 0.456293 | 0.447021 | 0.560361 | 0.683556 | 0.572919 | 0.328292 | 0.50901 | 0.170375 | 0.562471 | 0.284753 | 0.57318 | 0.341574 | 0.218624 | 0.641507 | 0.304657 | 0.565861 | 0.488476 | 0.338408 | 0.328259 | 0.562566 | 0.481041 | 0.426161 | 0.405605 | 0.320361 | 0.402909 |
| TCGA-AZ-4614-01A-01R-1410-07 | -0.09305 | 0.367444 | 0.465915 | 0.325378 | 0.442268 | 0.593124 | 0.598376 | 0.464151 | 0.269241 | 0.299113 | 0.03316 | 0.429503 | -0.00919 | 0.449822 | 0.142939 | 0.073039 | 0.324323 | 0.234245 | 0.563741 | 0.397309 | 0.23582 | 0.023167 | 0.474211 | 0.229251 | 0.246294 | 0.272924 | 0.263999 | 0.322756 |
| TCGA-G4-6320-01A-11R-1723-07 | 0.123096 | 0.40934 | 0.439013 | 0.276996 | 0.456087 | 0.554136 | 0.665285 | 0.451505 | 0.277451 | 0.284827 | 0.107592 | 0.416745 | 0.150372 | 0.415219 | 0.155586 | 0.044879 | 0.384484 | 0.260428 | 0.545361 | 0.397542 | 0.16959 | -0.10338 | 0.46507 | 0.198222 | 0.275329 | 0.273587 | 0.250381 | 0.31621 |
| TCGA-AA-3856-01A-01R-0905-07 | 0.201991 | 0.446905 | 0.516732 | 0.399448 | 0.472412 | 0.560905 | 0.663844 | 0.50818 | 0.329126 | 0.370561 | 0.18054 | 0.528993 | 0.257375 | 0.488743 | 0.256406 | 0.282963 | 0.503408 | 0.310999 | 0.589742 | 0.444286 | 0.272285 | 0.067967 | 0.525967 | 0.428618 | 0.406027 | 0.363178 | 0.289041 | 0.284944 |
| TCGA-A6-6138-01A-11R-1774-07 | 0.177182 | 0.505162 | 0.561245 | 0.425538 | 0.4961 | 0.554271 | 0.667825 | 0.558602 | 0.392792 | 0.423658 | 0.174248 | 0.539596 | 0.26772 | 0.488355 | 0.305026 | 0.278938 | 0.588326 | 0.298672 | 0.569452 | 0.517847 | 0.330287 | 0.100103 | 0.541989 | 0.504648 | 0.392342 | 0.402129 | 0.305437 | 0.331057 |
| TCGA-A6-2685-01A-01R-1410-07 | 0.179047 | 0.35047 | 0.482642 | 0.413715 | 0.451545 | 0.58849 | 0.690358 | 0.580048 | 0.354856 | 0.402349 | 0.222894 | 0.558694 | 0.249989 | 0.499208 | 0.343352 | 0.385645 | 0.589785 | 0.270512 | 0.593757 | 0.495545 | 0.33043 | 0.15935 | 0.575241 | 0.493135 | 0.392303 | 0.390537 | 0.311783 | 0.289918 |
| TCGA-QG-A5YX-01A-11R-A28H-07 | -0.02171 | 0.433423 | 0.38411 | 0.352029 | 0.432124 | 0.55777 | 0.589435 | 0.454311 | 0.300568 | 0.273095 | 0.090995 | 0.454635 | 0.059194 | 0.455591 | 0.158147 | 0.119466 | 0.306553 | 0.31742 | 0.547957 | 0.357054 | 0.208174 | 0.032934 | 0.474202 | 0.247067 | 0.238562 | 0.274119 | 0.27289 | 0.278237 |
| TCGA-DM-A1D8-01A-11R-A155-07 | -0.11315 | 0.350518 | 0.423789 | 0.285589 | 0.45343 | 0.50022 | 0.605647 | 0.412339 | 0.232856 | 0.225241 | 0.008159 | 0.375705 | -0.02095 | 0.425912 | 0.071062 | 0.043138 | 0.258125 | 0.273699 | 0.520241 | 0.364758 | 0.193029 | -0.04515 | 0.501333 | 0.191673 | 0.236884 | 0.25945 | 0.222795 | 0.279895 |
| TCGA-AM-5821-01A-01R-1653-07 | 0.163466 | 0.591006 | 0.565479 | 0.422791 | 0.444886 | 0.529235 | 0.680299 | 0.533119 | 0.368131 | 0.448314 | 0.10571 | 0.542614 | 0.271053 | 0.521495 | 0.276708 | 0.255873 | 0.560454 | 0.223995 | 0.561405 | 0.465396 | 0.341309 | 0.20029 | 0.519757 | 0.441261 | 0.343864 | 0.382307 | 0.292341 | 0.397983 |
| TCGA-CK-5916-01A-11R-1653-07 | 0.150717 | 0.528647 | 0.571138 | 0.418897 | 0.450212 | 0.539063 | 0.663386 | 0.585833 | 0.356622 | 0.441725 | 0.108826 | 0.50052 | 0.276017 | 0.501308 | 0.287785 | 0.218055 | 0.592096 | 0.303997 | 0.558264 | 0.505954 | 0.32565 | 0.079681 | 0.512526 | 0.472564 | 0.390906 | 0.409595 | 0.243004 | 0.376992 |
| TCGA-NH-A50T-01A-11R-A28H-07 | -0.14263 | 0.400997 | 0.333976 | 0.328119 | 0.43829 | 0.513323 | 0.571154 | 0.43293 | 0.288743 | 0.234493 | 0.069242 | 0.450973 | -0.00236 | 0.444551 | 0.130704 | 0.086996 | 0.305743 | 0.256357 | 0.504181 | 0.373363 | 0.205632 | 0.065016 | 0.457644 | 0.251866 | 0.224097 | 0.280121 | 0.168572 | 0.249316 |
| TCGA-WS-AB45-01A-11R-A41B-07 | 0.091632 | 0.515544 | 0.495493 | 0.475706 | 0.429602 | 0.552595 | 0.716805 | 0.628496 | 0.44043 | 0.49814 | 0.269559 | 0.560674 | 0.258231 | 0.566609 | 0.450491 | 0.409811 | 0.706502 | 0.328669 | 0.57323 | 0.560651 | 0.388485 | 0.449541 | 0.624863 | 0.648482 | 0.427722 | 0.439998 | 0.259071 | 0.394572 |
| TCGA-AA-A00Z-01A-01R-A002-07 | 0.021618 | 0.41357 | 0.43176 | 0.345698 | 0.451239 | 0.53877 | 0.591957 | 0.443136 | 0.307761 | 0.28082 | 0.059223 | 0.473875 | 0.075324 | 0.458766 | 0.16929 | 0.116687 | 0.379822 | 0.309461 | 0.533787 | 0.426283 | 0.20878 | 0.025909 | 0.523214 | 0.318985 | 0.266105 | 0.284687 | 0.301708 | 0.245581 |
| TCGA-CM-6674-01A-11R-1839-07 | 0.04312 | 0.44819 | 0.498337 | 0.381451 | 0.453191 | 0.563464 | 0.672986 | 0.533875 | 0.345468 | 0.384269 | 0.089763 | 0.497571 | 0.171546 | 0.528196 | 0.280756 | 0.181439 | 0.530264 | 0.28856 | 0.543243 | 0.467576 | 0.321803 | 0.049975 | 0.528111 | 0.388195 | 0.326275 | 0.360671 | 0.289257 | 0.390089 |
| TCGA-AA-3833-01A-01R-0905-07 | 0.132264 | 0.469413 | 0.503577 | 0.453522 | 0.455733 | 0.615674 | 0.672109 | 0.566545 | 0.316805 | 0.39585 | 0.153923 | 0.545074 | 0.213934 | 0.520444 | 0.31463 | 0.366716 | 0.570911 | 0.287451 | 0.557819 | 0.476367 | 0.324876 | 0.195532 | 0.542412 | 0.440155 | 0.368867 | 0.379947 | 0.316433 | 0.334927 |
| TCGA-A6-2680-01A-01R-1410-07 | 0.078954 | 0.385041 | 0.418266 | 0.355079 | 0.450886 | 0.574413 | 0.648153 | 0.513539 | 0.287136 | 0.331037 | 0.115517 | 0.475743 | 0.159235 | 0.468923 | 0.227934 | 0.200531 | 0.476108 | 0.277311 | 0.595254 | 0.445316 | 0.254369 | 0.116704 | 0.512273 | 0.377791 | 0.325391 | 0.330902 | 0.284426 | 0.252466 |
| TCGA-CM-5862-01A-01R-1653-07 | -0.13532 | 0.332252 | 0.376619 | 0.304808 | 0.462124 | 0.539495 | 0.622704 | 0.456092 | 0.304391 | 0.184005 | 0.072706 | 0.462451 | -0.02001 | 0.493303 | 0.166898 | 0.152318 | 0.332337 | 0.263923 | 0.519838 | 0.417928 | 0.236649 | 0.025012 | 0.493858 | 0.272234 | 0.281721 | 0.290469 | 0.195007 | 0.25646 |
| TCGA-AA-3555-01A-01R-0821-07 | -0.06328 | 0.442084 | 0.397783 | 0.40075 | 0.440046 | 0.551882 | 0.659342 | 0.573485 | 0.380388 | 0.321997 | 0.121978 | 0.504712 | 0.078165 | 0.530823 | 0.23538 | 0.174782 | 0.525284 | 0.2461 | 0.540416 | 0.442062 | 0.266101 | 0.213295 | 0.537597 | 0.420997 | 0.272682 | 0.33387 | 0.280079 | 0.358479 |
| TCGA-DM-A28G-01A-11R-A16W-07 | -0.0214 | 0.361767 | 0.430993 | 0.299856 | 0.486182 | 0.525593 | 0.628989 | 0.429419 | 0.293024 | 0.258286 | 0.084965 | 0.434908 | 0.053629 | 0.447514 | 0.14934 | 0.068049 | 0.371852 | 0.283596 | 0.524905 | 0.409428 | 0.167414 | -0.1037 | 0.512474 | 0.253299 | 0.262322 | 0.304749 | 0.282375 | 0.295162 |
| TCGA-5M-AATE-01A-11R-A41B-07 | -0.00955 | 0.398935 | 0.383712 | 0.301365 | 0.473655 | 0.522997 | 0.629562 | 0.454238 | 0.291211 | 0.237341 | 0.123537 | 0.413049 | 0.081211 | 0.452153 | 0.116821 | 0.13892 | 0.34034 | 0.295775 | 0.530089 | 0.404945 | 0.212797 | -0.05086 | 0.482752 | 0.287412 | 0.243694 | 0.275642 | 0.207166 | 0.258815 |
| TCGA-DM-A28H-01A-11R-A16W-07 | -0.01064 | 0.421055 | 0.362079 | 0.319503 | 0.413453 | 0.518266 | 0.563872 | 0.429834 | 0.27912 | 0.1785 | 0.020177 | 0.448293 | 0.044371 | 0.45791 | 0.137113 | 0.165212 | 0.296711 | 0.302556 | 0.530316 | 0.351895 | 0.186372 | -0.04879 | 0.520903 | 0.246229 | 0.244248 | 0.235209 | 0.24617 | 0.252815 |
| TCGA-AA-A024-01A-02R-A00A-07 | -0.12848 | 0.301126 | 0.436533 | 0.319809 | 0.470914 | 0.576473 | 0.625625 | 0.454222 | 0.280887 | 0.269131 | 0.092904 | 0.439007 | 0.070049 | 0.452237 | 0.128467 | 0.107682 | 0.407892 | 0.25247 | 0.524259 | 0.388487 | 0.187552 | 0.106783 | 0.484694 | 0.265406 | 0.280536 | 0.28894 | 0.33332 | 0.276071 |
| TCGA-CM-4747-01A-01R-1410-07 | 0.036746 | 0.284818 | 0.434068 | 0.330787 | 0.458765 | 0.604508 | 0.652991 | 0.477903 | 0.277257 | 0.302786 | 0.028768 | 0.466615 | 0.082072 | 0.439568 | 0.211416 | 0.117996 | 0.476274 | 0.185102 | 0.554631 | 0.438163 | 0.226225 | -0.05499 | 0.461005 | 0.347207 | 0.292783 | 0.333391 | 0.326674 | 0.220448 |
| TCGA-G4-6314-01A-11R-1723-07 | 0.034755 | 0.409822 | 0.426573 | 0.347164 | 0.442765 | 0.519872 | 0.650151 | 0.504015 | 0.359918 | 0.341796 | 0.177204 | 0.478032 | 0.141135 | 0.451032 | 0.252742 | 0.256396 | 0.46271 | 0.296482 | 0.5196 | 0.475711 | 0.297445 | -0.07589 | 0.528647 | 0.40451 | 0.312638 | 0.357557 | 0.223811 | 0.312093 |
| TCGA-AA-3930-01A-01R-1022-07 | 0.078586 | 0.520419 | 0.483446 | 0.41023 | 0.460415 | 0.531833 | 0.65165 | 0.544269 | 0.361994 | 0.372579 | 0.128901 | 0.516289 | 0.217276 | 0.515933 | 0.267376 | 0.335057 | 0.536118 | 0.272673 | 0.537391 | 0.440324 | 0.293849 | 0.254525 | 0.51767 | 0.404722 | 0.347329 | 0.348997 | 0.262922 | 0.340665 |
| TCGA-CA-6718-01A-11R-1839-07 | 0.169111 | 0.60266 | 0.590326 | 0.453395 | 0.455991 | 0.542446 | 0.679579 | 0.555544 | 0.373918 | 0.490332 | 0.114604 | 0.504782 | 0.283773 | 0.521016 | 0.243133 | 0.149122 | 0.590795 | 0.26304 | 0.535193 | 0.500262 | 0.358016 | 0.05541 | 0.515464 | 0.456837 | 0.354614 | 0.400658 | 0.25195 | 0.419337 |
| TCGA-AD-6963-01A-11R-1928-07 | 0.027673 | 0.511635 | 0.513803 | 0.41091 | 0.458047 | 0.543909 | 0.634202 | 0.49997 | 0.328248 | 0.37042 | 0.122987 | 0.500144 | 0.175791 | 0.468964 | 0.188283 | 0.172103 | 0.469662 | 0.310224 | 0.572139 | 0.426072 | 0.260019 | 0.202318 | 0.536332 | 0.3747 | 0.316942 | 0.339522 | 0.227023 | 0.282848 |
| TCGA-AA-3982-01A-02R-1022-07 | 0.118822 | 0.465682 | 0.554349 | 0.399364 | 0.457678 | 0.560483 | 0.668268 | 0.507513 | 0.326619 | 0.360079 | 0.123376 | 0.544845 | 0.204495 | 0.49392 | 0.2587 | 0.257284 | 0.530211 | 0.331498 | 0.575139 | 0.451647 | 0.281942 | 0.05424 | 0.520893 | 0.431548 | 0.380337 | 0.364988 | 0.291633 | 0.2718 |
| TCGA-5M-AATA-01A-31R-A41B-07 | -0.0476 | 0.439941 | 0.477726 | 0.355685 | 0.447653 | 0.546442 | 0.639869 | 0.46186 | 0.345198 | 0.290666 | 0.142584 | 0.466509 | 0.081483 | 0.474683 | 0.180265 | 0.163101 | 0.412881 | 0.264793 | 0.525536 | 0.429022 | 0.2154 | 0.029294 | 0.528213 | 0.346685 | 0.302505 | 0.330197 | 0.240582 | 0.343461 |
| TCGA-CM-6680-01A-11R-1839-07 | 0.075511 | 0.444099 | 0.484666 | 0.40551 | 0.46692 | 0.56253 | 0.685894 | 0.505081 | 0.315466 | 0.400229 | 0.141236 | 0.489792 | 0.154775 | 0.485087 | 0.258233 | 0.205916 | 0.539286 | 0.269603 | 0.551113 | 0.471496 | 0.265745 | -0.01533 | 0.531753 | 0.419247 | 0.319811 | 0.351978 | 0.345057 | 0.324143 |
| TCGA-DM-A1D6-01A-21R-A155-07 | -0.21193 | 0.332061 | 0.330105 | 0.267558 | 0.413069 | 0.563206 | 0.622376 | 0.401014 | 0.259533 | 0.200372 | 0.005039 | 0.394725 | -0.01788 | 0.444519 | 0.133494 | 0.030285 | 0.271416 | 0.256072 | 0.483158 | 0.372083 | 0.175654 | -0.0019 | 0.479121 | 0.116392 | 0.181342 | 0.222234 | 0.198894 | 0.355778 |
| TCGA-AZ-6603-01A-11R-1839-07 | 0.056498 | 0.406011 | 0.454138 | 0.376349 | 0.450419 | 0.532968 | 0.641679 | 0.502665 | 0.333315 | 0.348086 | 0.10513 | 0.49221 | 0.15626 | 0.453504 | 0.21984 | 0.203547 | 0.500191 | 0.301881 | 0.536919 | 0.466416 | 0.25682 | 0.075554 | 0.562095 | 0.400878 | 0.311044 | 0.329492 | 0.241095 | 0.267418 |
| TCGA-A6-6142-01A-11R-1774-07 | -0.02631 | 0.353991 | 0.38574 | 0.395559 | 0.4722 | 0.547716 | 0.677401 | 0.549324 | 0.32239 | 0.311052 | 0.161334 | 0.513619 | 0.104404 | 0.520013 | 0.273939 | 0.252782 | 0.502005 | 0.262696 | 0.546368 | 0.458368 | 0.288219 | 0.123891 | 0.568241 | 0.442046 | 0.34144 | 0.34857 | 0.262943 | 0.258764 |
| TCGA-AZ-4315-01A-01R-1410-07 | 0.077539 | 0.474578 | 0.495149 | 0.392081 | 0.475066 | 0.566263 | 0.633929 | 0.487699 | 0.327015 | 0.353951 | 0.095244 | 0.494498 | 0.134337 | 0.487297 | 0.240556 | 0.197938 | 0.482569 | 0.231579 | 0.546917 | 0.423686 | 0.292283 | 0.017466 | 0.45935 | 0.386588 | 0.326333 | 0.334278 | 0.311368 | 0.300028 |
| TCGA-AD-6895-01A-11R-1928-07 | 0.052262 | 0.494995 | 0.551899 | 0.39744 | 0.442357 | 0.577419 | 0.697793 | 0.51554 | 0.361328 | 0.435439 | 0.13694 | 0.483162 | 0.184715 | 0.4607 | 0.231252 | 0.224609 | 0.497415 | 0.260998 | 0.526525 | 0.487547 | 0.311532 | 0.127375 | 0.533818 | 0.350498 | 0.341084 | 0.370383 | 0.292584 | 0.391872 |
| TCGA-CA-5796-01A-01R-1653-07 | 0.08644 | 0.408489 | 0.485536 | 0.385389 | 0.46396 | 0.54521 | 0.648292 | 0.488821 | 0.259396 | 0.331525 | 0.112177 | 0.461284 | 0.223434 | 0.455918 | 0.211398 | 0.147472 | 0.41924 | 0.281481 | 0.554539 | 0.410688 | 0.242276 | 0.054074 | 0.507094 | 0.310121 | 0.309833 | 0.318884 | 0.34472 | 0.377973 |
| TCGA-AA-3510-01A-01R-1410-07 | 0.141667 | 0.450192 | 0.492659 | 0.384587 | 0.452495 | 0.570349 | 0.641943 | 0.543521 | 0.329314 | 0.353978 | 0.163169 | 0.523347 | 0.206704 | 0.509808 | 0.271729 | 0.308496 | 0.559241 | 0.269897 | 0.570771 | 0.435294 | 0.270946 | 0.11062 | 0.505514 | 0.420158 | 0.355156 | 0.351966 | 0.294538 | 0.265561 |
| TCGA-AA-3527-01A-01R-0821-07 | 0.073552 | 0.297407 | 0.488283 | 0.388602 | 0.464017 | 0.645489 | 0.637404 | 0.522656 | 0.245417 | 0.363976 | 0.086923 | 0.486933 | 0.113182 | 0.457662 | 0.272948 | 0.243973 | 0.540869 | 0.200599 | 0.623256 | 0.458046 | 0.262429 | 0.082218 | 0.479478 | 0.409758 | 0.391692 | 0.377442 | 0.284348 | 0.269545 |
| TCGA-F4-6807-01A-11R-1839-07 | 0.208329 | 0.498369 | 0.553641 | 0.4228 | 0.454178 | 0.536889 | 0.676255 | 0.584984 | 0.397628 | 0.442169 | 0.217926 | 0.516454 | 0.282947 | 0.523589 | 0.310915 | 0.324309 | 0.574947 | 0.328522 | 0.553164 | 0.511578 | 0.315169 | 0.06638 | 0.556533 | 0.469631 | 0.359312 | 0.398505 | 0.273517 | 0.355045 |
| TCGA-G4-6310-01A-11R-1723-07 | -0.16202 | 0.250694 | 0.279199 | 0.245546 | 0.459573 | 0.482266 | 0.604706 | 0.448562 | 0.363174 | 0.207034 | 0.132757 | 0.428129 | 0.00116 | 0.439559 | 0.096983 | 0.126358 | 0.230989 | 0.269275 | 0.515253 | 0.44811 | 0.216378 | -0.14818 | 0.499812 | 0.203569 | 0.22053 | 0.254117 | 0.118876 | 0.277443 |
| TCGA-AA-A00K-01A-02R-A002-07 | 0.0355 | 0.424754 | 0.444691 | 0.340606 | 0.432941 | 0.597289 | 0.642664 | 0.470829 | 0.285089 | 0.299452 | 0.071376 | 0.4929 | 0.115609 | 0.478876 | 0.217024 | 0.203575 | 0.421845 | 0.262585 | 0.556386 | 0.426177 | 0.257916 | 0.068584 | 0.525297 | 0.35188 | 0.278755 | 0.332808 | 0.32083 | 0.302157 |
| TCGA-D5-6529-01A-11R-1774-07 | 0.219663 | 0.493886 | 0.526349 | 0.449274 | 0.451367 | 0.565397 | 0.696821 | 0.577303 | 0.358195 | 0.435417 | 0.186202 | 0.540496 | 0.291496 | 0.514372 | 0.31395 | 0.291033 | 0.589374 | 0.316773 | 0.561043 | 0.481833 | 0.289788 | 0.163226 | 0.562016 | 0.496574 | 0.379829 | 0.414415 | 0.2805 | 0.316367 |
| TCGA-AA-A01I-01A-02R-A089-07 | -0.03688 | 0.457524 | 0.447686 | 0.343516 | 0.487477 | 0.535683 | 0.634278 | 0.483111 | 0.333738 | 0.311364 | 0.100554 | 0.480732 | 0.104279 | 0.477678 | 0.166291 | 0.159367 | 0.363471 | 0.264286 | 0.530293 | 0.421077 | 0.21265 | 0.087021 | 0.512811 | 0.310983 | 0.28726 | 0.311811 | 0.294884 | 0.33027 |
| TCGA-AA-A01Z-01A-11R-A083-07 | -0.14528 | 0.328351 | 0.375501 | 0.316868 | 0.440754 | 0.557217 | 0.606197 | 0.456415 | 0.229149 | 0.221552 | 0.086838 | 0.421959 | 0.010367 | 0.450053 | 0.073413 | 0.095129 | 0.300376 | 0.284193 | 0.552858 | 0.383614 | 0.229995 | 0.013953 | 0.469117 | 0.228879 | 0.243282 | 0.268154 | 0.292059 | 0.203616 |
| TCGA-G4-6588-01A-11R-1774-07 | -0.04108 | 0.500387 | 0.450795 | 0.389021 | 0.436012 | 0.547773 | 0.660077 | 0.517996 | 0.309602 | 0.383043 | 0.098172 | 0.489301 | 0.093827 | 0.48767 | 0.227431 | 0.146149 | 0.513297 | 0.254835 | 0.525557 | 0.457551 | 0.308516 | 0.161168 | 0.535492 | 0.391011 | 0.298206 | 0.329148 | 0.259065 | 0.369144 |
| TCGA-CK-5912-01A-11R-1653-07 | -0.01608 | 0.388586 | 0.379348 | 0.315937 | 0.46313 | 0.490305 | 0.634486 | 0.455327 | 0.3399 | 0.260272 | 0.075009 | 0.445042 | 0.073534 | 0.51337 | 0.173931 | 0.154238 | 0.375332 | 0.308982 | 0.535728 | 0.412611 | 0.213499 | -0.01968 | 0.518734 | 0.30102 | 0.276493 | 0.298102 | 0.243467 | 0.281927 |
| TCGA-CM-6679-01A-11R-1839-07 | 0.149656 | 0.363629 | 0.441321 | 0.357251 | 0.459867 | 0.535857 | 0.675945 | 0.497561 | 0.352949 | 0.347008 | 0.1541 | 0.475722 | 0.195215 | 0.459676 | 0.236783 | 0.197362 | 0.475871 | 0.310391 | 0.540156 | 0.486409 | 0.249112 | 0.049957 | 0.53651 | 0.377875 | 0.295112 | 0.346099 | 0.24635 | 0.261174 |
| TCGA-AA-3519-01A-02R-0821-07 | 0.034422 | 0.441287 | 0.444114 | 0.334769 | 0.44589 | 0.566616 | 0.632965 | 0.485515 | 0.341739 | 0.299448 | 0.159378 | 0.481098 | 0.120598 | 0.460298 | 0.19971 | 0.22566 | 0.45047 | 0.270286 | 0.550382 | 0.420897 | 0.22396 | 0.144916 | 0.50982 | 0.343916 | 0.2963 | 0.322776 | 0.312525 | 0.282185 |
| TCGA-AZ-6607-01A-11R-1839-07 | 0.063879 | 0.436428 | 0.510706 | 0.403318 | 0.44507 | 0.57511 | 0.672693 | 0.587964 | 0.371374 | 0.458577 | 0.178826 | 0.527679 | 0.21824 | 0.511731 | 0.297503 | 0.334424 | 0.560741 | 0.310862 | 0.555907 | 0.516164 | 0.354084 | 0.124316 | 0.567704 | 0.51316 | 0.402381 | 0.402627 | 0.292832 | 0.386746 |
| TCGA-CM-5344-01A-21R-1723-07 | -0.05508 | 0.354093 | 0.407103 | 0.349724 | 0.45747 | 0.560697 | 0.639111 | 0.48972 | 0.316577 | 0.276573 | 0.099151 | 0.48313 | 0.091394 | 0.452648 | 0.191226 | 0.165726 | 0.385626 | 0.267566 | 0.508518 | 0.472172 | 0.255672 | -0.06129 | 0.487008 | 0.34201 | 0.282887 | 0.327513 | 0.269949 | 0.29131 |
| TCGA-AA-3831-01A-01R-0905-07 | 0.094065 | 0.470437 | 0.469755 | 0.386904 | 0.442109 | 0.563427 | 0.631072 | 0.475751 | 0.300366 | 0.306824 | 0.168152 | 0.48223 | 0.188911 | 0.464066 | 0.215847 | 0.169388 | 0.479088 | 0.27567 | 0.559063 | 0.432015 | 0.250661 | 0.119069 | 0.506766 | 0.374081 | 0.342707 | 0.366731 | 0.289388 | 0.300451 |
| TCGA-A6-2672-01A-01R-0826-07 | 0.090459 | 0.521423 | 0.600212 | 0.484623 | 0.439763 | 0.59378 | 0.691653 | 0.563932 | 0.298686 | 0.480031 | 0.190808 | 0.51558 | 0.277738 | 0.502952 | 0.311253 | 0.237844 | 0.628626 | 0.251728 | 0.562299 | 0.496077 | 0.335814 | 0.188392 | 0.535538 | 0.507897 | 0.397053 | 0.383629 | 0.252094 | 0.321914 |
| TCGA-DM-A0X9-01A-11R-A155-07 | 0.034667 | 0.458342 | 0.495303 | 0.341579 | 0.445008 | 0.540305 | 0.632627 | 0.46408 | 0.280089 | 0.318507 | 0.087234 | 0.452605 | 0.075226 | 0.465768 | 0.175546 | 0.12025 | 0.398719 | 0.276099 | 0.532895 | 0.394231 | 0.217302 | -0.03387 | 0.507906 | 0.280286 | 0.273124 | 0.304097 | 0.226873 | 0.315464 |
| TCGA-D5-6541-01A-11R-1723-07 | 0.104268 | 0.395759 | 0.495286 | 0.381447 | 0.491777 | 0.566471 | 0.672191 | 0.537282 | 0.368943 | 0.42708 | 0.133069 | 0.513488 | 0.238755 | 0.52596 | 0.289357 | 0.181817 | 0.583928 | 0.288733 | 0.565888 | 0.482405 | 0.299271 | 0.095959 | 0.549149 | 0.462581 | 0.366546 | 0.378749 | 0.258148 | 0.254662 |
| TCGA-A6-4107-01A-02R-1410-07 | 0.183788 | 0.413297 | 0.460557 | 0.389706 | 0.462075 | 0.57673 | 0.675758 | 0.492749 | 0.313212 | 0.333078 | 0.112948 | 0.47992 | 0.210463 | 0.47876 | 0.259078 | 0.221557 | 0.491022 | 0.259219 | 0.577516 | 0.427521 | 0.264233 | 0.05896 | 0.520439 | 0.390355 | 0.355931 | 0.354768 | 0.321015 | 0.314602 |
| TCGA-AA-3875-01A-01R-0905-07 | 0.13732 | 0.481233 | 0.48117 | 0.393908 | 0.469271 | 0.547758 | 0.651389 | 0.54222 | 0.301569 | 0.329383 | 0.169762 | 0.528197 | 0.212749 | 0.498248 | 0.266566 | 0.28691 | 0.524546 | 0.28826 | 0.545509 | 0.433508 | 0.233484 | 0.228835 | 0.537869 | 0.414915 | 0.334366 | 0.367342 | 0.267396 | 0.309714 |
| TCGA-G4-6295-01A-11R-1723-07 | 0.219097 | 0.536821 | 0.563175 | 0.403069 | 0.462929 | 0.543978 | 0.654094 | 0.490074 | 0.308101 | 0.378503 | 0.224761 | 0.483216 | 0.287428 | 0.446275 | 0.246007 | 0.155278 | 0.507297 | 0.318877 | 0.548713 | 0.45776 | 0.275754 | 0.027161 | 0.518548 | 0.408313 | 0.35403 | 0.353325 | 0.308583 | 0.317486 |
| TCGA-DM-A28F-01A-11R-A32Y-07 | 0.039541 | 0.407638 | 0.442317 | 0.307096 | 0.438403 | 0.547754 | 0.627005 | 0.410977 | 0.28801 | 0.280222 | 0.066002 | 0.451601 | 0.133542 | 0.427179 | 0.10884 | 0.048618 | 0.329513 | 0.278752 | 0.507119 | 0.398608 | 0.208302 | -0.03167 | 0.49061 | 0.197548 | 0.255252 | 0.294485 | 0.252942 | 0.213014 |
| TCGA-CM-4743-01A-01R-1723-07 | -0.06768 | 0.49668 | 0.494357 | 0.4028 | 0.442738 | 0.526425 | 0.647785 | 0.472313 | 0.299863 | 0.441309 | 0.155002 | 0.496486 | 0.143248 | 0.464065 | 0.164083 | 0.125629 | 0.42168 | 0.234071 | 0.546662 | 0.440198 | 0.290254 | 0.217368 | 0.507288 | 0.269605 | 0.301653 | 0.331243 | 0.311529 | 0.393832 |
| TCGA-F4-6808-01A-11R-1839-07 | -0.1341 | 0.334989 | 0.370605 | 0.325498 | 0.453379 | 0.582956 | 0.611366 | 0.443324 | 0.263541 | 0.179131 | 0.041194 | 0.446121 | -0.01383 | 0.435805 | 0.109541 | 0.055831 | 0.31222 | 0.250322 | 0.572385 | 0.382898 | 0.216712 | 0.108646 | 0.536235 | 0.249588 | 0.267713 | 0.273392 | 0.231559 | 0.236565 |
| TCGA-AA-A00U-01A-01R-A002-07 | -0.03718 | 0.442619 | 0.451427 | 0.341905 | 0.466536 | 0.548671 | 0.627733 | 0.472437 | 0.287167 | 0.285231 | 0.105146 | 0.456649 | 0.056134 | 0.474311 | 0.120811 | 0.169546 | 0.398446 | 0.253139 | 0.530933 | 0.414842 | 0.220278 | 0.003528 | 0.485682 | 0.303366 | 0.279759 | 0.320204 | 0.321175 | 0.329136 |
| TCGA-AA-A017-01A-01R-A00A-07 | 0.002557 | 0.335163 | 0.420979 | 0.334302 | 0.44575 | 0.561979 | 0.649199 | 0.497358 | 0.312431 | 0.27338 | 0.094559 | 0.476611 | 0.081245 | 0.472862 | 0.173213 | 0.12483 | 0.426731 | 0.275224 | 0.546367 | 0.434953 | 0.225718 | 0.137909 | 0.545634 | 0.332722 | 0.293049 | 0.301777 | 0.249266 | 0.201868 |
| TCGA-AA-A01X-01A-21R-A083-07 | 0.084145 | 0.290927 | 0.394864 | 0.323675 | 0.467456 | 0.554579 | 0.653063 | 0.459152 | 0.262345 | 0.273401 | 0.050796 | 0.428607 | 0.13219 | 0.480508 | 0.201758 | 0.092302 | 0.405677 | 0.232018 | 0.57471 | 0.403361 | 0.206373 | 0.008745 | 0.5022 | 0.302371 | 0.308044 | 0.299251 | 0.263407 | 0.267123 |
| TCGA-CM-5861-01A-01R-1653-07 | -0.0809 | 0.409816 | 0.435766 | 0.361956 | 0.443366 | 0.540392 | 0.656844 | 0.511948 | 0.317079 | 0.336144 | 0.018784 | 0.439916 | 0.044462 | 0.476829 | 0.182619 | 0.101303 | 0.414538 | 0.258995 | 0.536826 | 0.434523 | 0.275421 | 0.091592 | 0.456116 | 0.289311 | 0.275126 | 0.316049 | 0.284486 | 0.390812 |
| TCGA-D5-5537-01A-21R-1928-07 | -0.00195 | 0.367309 | 0.406612 | 0.336128 | 0.460354 | 0.556383 | 0.646262 | 0.437394 | 0.290078 | 0.285511 | 0.123104 | 0.447656 | 0.083659 | 0.462823 | 0.177792 | 0.116722 | 0.386444 | 0.225587 | 0.543893 | 0.416944 | 0.204045 | -0.02062 | 0.496928 | 0.289494 | 0.257435 | 0.307176 | 0.254877 | 0.267261 |
| TCGA-AA-3968-01A-01R-1022-07 | 0.104157 | 0.460788 | 0.448175 | 0.371976 | 0.448081 | 0.552135 | 0.639478 | 0.500358 | 0.323176 | 0.344421 | 0.124617 | 0.49784 | 0.200122 | 0.48145 | 0.254842 | 0.226393 | 0.476754 | 0.317129 | 0.541348 | 0.454612 | 0.258325 | 0.081707 | 0.5176 | 0.379298 | 0.332439 | 0.339832 | 0.232258 | 0.288032 |
| TCGA-D5-6530-01A-11R-1723-07 | 0.147999 | 0.495424 | 0.5346 | 0.389624 | 0.435987 | 0.571251 | 0.683594 | 0.508395 | 0.313488 | 0.435095 | 0.15756 | 0.477312 | 0.240724 | 0.493726 | 0.293537 | 0.131639 | 0.577598 | 0.267885 | 0.543514 | 0.4793 | 0.281771 | 0.168805 | 0.533277 | 0.387457 | 0.351094 | 0.35429 | 0.286744 | 0.336245 |
| TCGA-AA-3818-01A-01R-0905-07 | 0.035046 | 0.416352 | 0.460932 | 0.363346 | 0.4651 | 0.57663 | 0.633031 | 0.520447 | 0.299615 | 0.256288 | 0.148682 | 0.497068 | 0.123186 | 0.437176 | 0.19776 | 0.137636 | 0.416221 | 0.302464 | 0.565128 | 0.428675 | 0.242293 | 0.073103 | 0.505671 | 0.350591 | 0.3119 | 0.331878 | 0.28364 | 0.297961 |
| TCGA-G4-6315-01A-11R-1723-07 | 0.093317 | 0.37369 | 0.407151 | 0.276005 | 0.448945 | 0.534532 | 0.594549 | 0.449093 | 0.303921 | 0.223883 | 0.079688 | 0.432433 | 0.123237 | 0.405394 | 0.115766 | 0.073187 | 0.315306 | 0.306282 | 0.536081 | 0.378254 | 0.168576 | 0.014727 | 0.482388 | 0.250587 | 0.245364 | 0.285848 | 0.24664 | 0.283406 |
| TCGA-AA-3562-01A-02R-0821-07 | 0.118643 | 0.390401 | 0.453469 | 0.351056 | 0.471726 | 0.568694 | 0.641333 | 0.482224 | 0.315178 | 0.317781 | 0.097981 | 0.51165 | 0.163871 | 0.499686 | 0.243824 | 0.256997 | 0.47302 | 0.284929 | 0.556982 | 0.434271 | 0.280335 | 0.05975 | 0.546398 | 0.376929 | 0.326103 | 0.325291 | 0.277367 | 0.243605 |
| TCGA-AA-3872-01A-01R-1022-07 | 0.101548 | 0.392692 | 0.451682 | 0.40871 | 0.442324 | 0.581765 | 0.697899 | 0.552767 | 0.381653 | 0.352768 | 0.170124 | 0.529816 | 0.19605 | 0.510041 | 0.313223 | 0.344228 | 0.549171 | 0.265306 | 0.582007 | 0.479413 | 0.328677 | 0.0712 | 0.540101 | 0.45307 | 0.390194 | 0.352997 | 0.257782 | 0.316309 |
| TCGA-AA-3845-01A-01R-1022-07 | 0.146488 | 0.517348 | 0.534115 | 0.374458 | 0.419699 | 0.522928 | 0.686945 | 0.507483 | 0.371271 | 0.415526 | 0.123395 | 0.550435 | 0.250912 | 0.551095 | 0.234072 | 0.228548 | 0.532415 | 0.206343 | 0.546833 | 0.474171 | 0.319311 | 0.172747 | 0.538759 | 0.410176 | 0.325056 | 0.360232 | 0.321796 | 0.36263 |
| TCGA-AA-A010-01A-01R-A089-07 | 0.045794 | 0.437842 | 0.490488 | 0.39481 | 0.463814 | 0.54793 | 0.645223 | 0.487996 | 0.287965 | 0.34459 | 0.032938 | 0.4798 | 0.152931 | 0.467196 | 0.212688 | 0.094414 | 0.459775 | 0.182191 | 0.543326 | 0.440061 | 0.235134 | 0.069039 | 0.502355 | 0.332078 | 0.32154 | 0.316963 | 0.301719 | 0.356276 |
| TCGA-AA-3529-01A-02R-0821-07 | -0.02424 | 0.363221 | 0.391448 | 0.333776 | 0.461363 | 0.565555 | 0.645037 | 0.489768 | 0.30949 | 0.295958 | 0.120902 | 0.520609 | 0.087948 | 0.524974 | 0.222308 | 0.302012 | 0.414231 | 0.298205 | 0.542526 | 0.386379 | 0.238767 | 0.114837 | 0.538976 | 0.382228 | 0.322386 | 0.323903 | 0.256858 | 0.287652 |
| TCGA-A6-2675-01A-02R-1723-07 | 0.098623 | 0.358644 | 0.452068 | 0.409877 | 0.460446 | 0.574344 | 0.682914 | 0.56855 | 0.359371 | 0.362502 | 0.151284 | 0.541828 | 0.22526 | 0.496932 | 0.294447 | 0.295239 | 0.542143 | 0.311134 | 0.553003 | 0.498252 | 0.293842 | 0.148275 | 0.581742 | 0.487837 | 0.349965 | 0.374625 | 0.272204 | 0.252683 |
| TCGA-AG-A023-01A-01R-A00A-07 | 0.1728 | 0.415665 | 0.487183 | 0.334278 | 0.445779 | 0.579197 | 0.638314 | 0.465789 | 0.274069 | 0.320056 | 0.134743 | 0.468621 | 0.181806 | 0.45568 | 0.205044 | 0.157848 | 0.42903 | 0.254683 | 0.584089 | 0.417928 | 0.239932 | -0.00184 | 0.498979 | 0.335541 | 0.307673 | 0.361328 | 0.275595 | 0.294831 |
| TCGA-DY-A1DF-01A-11R-A155-07 | -0.17636 | 0.257595 | 0.312476 | 0.278564 | 0.42633 | 0.55016 | 0.642324 | 0.406417 | 0.277454 | 0.211488 | 0.062107 | 0.432909 | -0.03526 | 0.420956 | 0.11429 | 0.112867 | 0.294093 | 0.248462 | 0.545084 | 0.413406 | 0.231616 | 0.009616 | 0.487501 | 0.197346 | 0.259006 | 0.249896 | 0.172756 | 0.259879 |
| TCGA-AA-3952-01A-01R-1022-07 | -0.07862 | 0.363474 | 0.41367 | 0.350909 | 0.428752 | 0.558171 | 0.629143 | 0.493909 | 0.311586 | 0.2472 | 0.095198 | 0.492687 | 0.052453 | 0.484536 | 0.229852 | 0.196272 | 0.502099 | 0.238098 | 0.504909 | 0.425954 | 0.269527 | 0.091738 | 0.501781 | 0.344433 | 0.285461 | 0.33256 | 0.225829 | 0.265384 |
| TCGA-AA-3532-01A-01R-0821-07 | 0.195058 | 0.458109 | 0.513533 | 0.432383 | 0.473279 | 0.554804 | 0.676338 | 0.536325 | 0.345496 | 0.392779 | 0.142878 | 0.539859 | 0.282128 | 0.516297 | 0.299724 | 0.254271 | 0.567794 | 0.314751 | 0.569732 | 0.471816 | 0.317044 | 0.118612 | 0.514586 | 0.473081 | 0.378363 | 0.385091 | 0.31195 | 0.299027 |
| TCGA-AA-3989-01A-01R-1022-07 | 0.17834 | 0.404751 | 0.512733 | 0.375109 | 0.451534 | 0.578746 | 0.663261 | 0.514352 | 0.323303 | 0.342559 | 0.122549 | 0.511371 | 0.211043 | 0.450848 | 0.253172 | 0.170113 | 0.550637 | 0.268393 | 0.541835 | 0.45929 | 0.252652 | 0.106355 | 0.540713 | 0.410897 | 0.337315 | 0.362709 | 0.31231 | 0.265685 |
| TCGA-AA-3530-01A-01R-1022-07 | 0.178573 | 0.450196 | 0.459168 | 0.334388 | 0.447457 | 0.505249 | 0.637252 | 0.460867 | 0.318036 | 0.343293 | 0.159926 | 0.465434 | 0.240556 | 0.484194 | 0.205785 | 0.17897 | 0.436544 | 0.325259 | 0.534281 | 0.408061 | 0.221104 | -0.00236 | 0.518289 | 0.345106 | 0.292623 | 0.320417 | 0.256433 | 0.301497 |
| TCGA-NH-A5IV-01A-42R-A37K-07 | -4.09E-06 | 0.46259 | 0.564537 | 0.395189 | 0.425334 | 0.551225 | 0.680059 | 0.514069 | 0.287328 | 0.389621 | 0.158736 | 0.509753 | 0.182662 | 0.488417 | 0.254864 | 0.175127 | 0.564075 | 0.268811 | 0.532955 | 0.459811 | 0.299568 | -0.04416 | 0.508098 | 0.428915 | 0.339746 | 0.331629 | 0.174743 | 0.279908 |
| TCGA-AA-3521-01A-01R-0821-07 | 0.033295 | 0.370186 | 0.399622 | 0.382084 | 0.468043 | 0.544697 | 0.644879 | 0.516929 | 0.304056 | 0.306978 | 0.109453 | 0.49873 | 0.120141 | 0.482427 | 0.228926 | 0.136389 | 0.46327 | 0.25286 | 0.54992 | 0.451051 | 0.243604 | 0.138067 | 0.507692 | 0.387151 | 0.293577 | 0.334328 | 0.264669 | 0.262249 |
| TCGA-AA-3554-01A-01R-0826-07 | 0.163031 | 0.476815 | 0.495628 | 0.478964 | 0.440644 | 0.606214 | 0.683378 | 0.596658 | 0.322331 | 0.44197 | 0.206632 | 0.565552 | 0.280277 | 0.542446 | 0.378216 | 0.325902 | 0.637122 | 0.247059 | 0.592153 | 0.493368 | 0.339887 | 0.261431 | 0.569248 | 0.508155 | 0.406012 | 0.402517 | 0.286 | 0.342357 |
| TCGA-D5-5538-01A-01R-1653-07 | 0.126908 | 0.483136 | 0.484982 | 0.462987 | 0.455526 | 0.560029 | 0.658559 | 0.573137 | 0.333455 | 0.396552 | 0.155458 | 0.559274 | 0.228198 | 0.517939 | 0.309345 | 0.244423 | 0.598854 | 0.302753 | 0.567237 | 0.483974 | 0.285471 | 0.128351 | 0.584198 | 0.511264 | 0.372001 | 0.402595 | 0.31024 | 0.313931 |
| TCGA-AG-A02X-01A-01R-A00A-07 | -0.01367 | 0.401869 | 0.413019 | 0.290461 | 0.415587 | 0.557329 | 0.621425 | 0.437842 | 0.26983 | 0.263569 | 0.105944 | 0.4189 | 0.03189 | 0.42183 | 0.116924 | 0.088482 | 0.366589 | 0.239461 | 0.491864 | 0.353362 | 0.181182 | 0.072019 | 0.473308 | 0.201793 | 0.24621 | 0.249704 | 0.259962 | 0.268798 |
| TCGA-AF-2692-01A-01R-0821-07 | -0.00247 | 0.45039 | 0.437641 | 0.347665 | 0.448602 | 0.546435 | 0.665196 | 0.488985 | 0.286295 | 0.293192 | 0.238827 | 0.446293 | 0.11475 | 0.475328 | 0.186819 | 0.242504 | 0.443118 | 0.271237 | 0.52362 | 0.443652 | 0.271355 | 0.115262 | 0.524849 | 0.347624 | 0.276426 | 0.336007 | 0.263543 | 0.293188 |
| TCGA-AG-3599-01A-02R-0826-07 | 0.077828 | 0.440187 | 0.504951 | 0.37305 | 0.460605 | 0.561042 | 0.655409 | 0.478751 | 0.315217 | 0.354964 | 0.11677 | 0.498256 | 0.175738 | 0.494041 | 0.251842 | 0.164122 | 0.472699 | 0.240148 | 0.55062 | 0.407305 | 0.275328 | 0.109216 | 0.485972 | 0.394188 | 0.348349 | 0.343489 | 0.332145 | 0.308811 |
| TCGA-AG-A016-01A-01R-A002-07 | -0.13006 | 0.318946 | 0.341477 | 0.241762 | 0.430853 | 0.559741 | 0.603956 | 0.429399 | 0.25168 | 0.211532 | 0.077727 | 0.433459 | -0.05137 | 0.441748 | 0.113776 | 0.083658 | 0.296717 | 0.255979 | 0.51239 | 0.357353 | 0.184513 | -0.04692 | 0.490726 | 0.250092 | 0.278136 | 0.244395 | 0.242741 | 0.228641 |
| TCGA-AF-A56L-01A-31R-A39D-07 | -0.00676 | 0.376688 | 0.399704 | 0.338019 | 0.428647 | 0.537502 | 0.631769 | 0.484581 | 0.294518 | 0.288422 | 0.064105 | 0.471787 | 0.093242 | 0.432055 | 0.179041 | 0.15268 | 0.37848 | 0.289341 | 0.506622 | 0.437113 | 0.22006 | -0.06704 | 0.521675 | 0.326788 | 0.270358 | 0.302575 | 0.228636 | 0.241966 |
| TCGA-AG-3578-01A-01R-0821-07 | -0.03525 | 0.409313 | 0.433046 | 0.364162 | 0.42513 | 0.612129 | 0.67577 | 0.521857 | 0.317535 | 0.326197 | 0.093839 | 0.497232 | 0.121608 | 0.544938 | 0.272937 | 0.214838 | 0.528809 | 0.27766 | 0.549344 | 0.410163 | 0.23546 | 0.215378 | 0.545022 | 0.379777 | 0.304148 | 0.302341 | 0.253946 | 0.366273 |
| TCGA-AG-3574-01A-01R-0821-07 | -0.01849 | 0.350061 | 0.44686 | 0.347878 | 0.459576 | 0.566588 | 0.603634 | 0.483791 | 0.299777 | 0.292445 | 0.094092 | 0.492987 | 0.087107 | 0.49114 | 0.240412 | 0.176898 | 0.471673 | 0.273126 | 0.564445 | 0.439018 | 0.222852 | 0.115201 | 0.524198 | 0.358797 | 0.311627 | 0.320633 | 0.259714 | 0.263288 |
| TCGA-F5-6465-01A-11R-1736-07 | 0.159152 | 0.437228 | 0.512687 | 0.403304 | 0.460657 | 0.525967 | 0.671567 | 0.535772 | 0.349718 | 0.397625 | 0.172212 | 0.506457 | 0.245568 | 0.482605 | 0.297078 | 0.292996 | 0.554081 | 0.319722 | 0.571111 | 0.500752 | 0.264445 | 0.143414 | 0.552315 | 0.470076 | 0.384634 | 0.400849 | 0.308702 | 0.302524 |
| TCGA-AG-3892-01A-01R-1119-07 | 0.206958 | 0.522776 | 0.577157 | 0.432345 | 0.466066 | 0.551725 | 0.648046 | 0.50417 | 0.271684 | 0.391353 | 0.167672 | 0.533335 | 0.259714 | 0.504344 | 0.291277 | 0.236447 | 0.551157 | 0.284811 | 0.584613 | 0.433458 | 0.282344 | 0.229747 | 0.534051 | 0.396755 | 0.352739 | 0.389716 | 0.350503 | 0.333526 |
| TCGA-AG-3587-01A-01R-0821-07 | -0.00341 | 0.401188 | 0.438499 | 0.339716 | 0.463775 | 0.586066 | 0.610171 | 0.483638 | 0.278996 | 0.31372 | 0.093168 | 0.465437 | 0.088055 | 0.4878 | 0.198178 | 0.169852 | 0.426583 | 0.243117 | 0.530032 | 0.41877 | 0.240461 | 0.03723 | 0.53844 | 0.381623 | 0.31841 | 0.331941 | 0.262466 | 0.242691 |
| TCGA-AG-A00Y-01A-02R-A002-07 | 0.065855 | 0.415656 | 0.463994 | 0.352435 | 0.481508 | 0.548598 | 0.635518 | 0.467804 | 0.311292 | 0.304013 | 0.092826 | 0.437776 | 0.162252 | 0.451626 | 0.194883 | 0.138511 | 0.431639 | 0.285188 | 0.554866 | 0.407858 | 0.207336 | 0.076848 | 0.46903 | 0.279273 | 0.311457 | 0.303099 | 0.279145 | 0.281474 |
| TCGA-AG-3896-01A-01R-1119-07 | 0.101575 | 0.393134 | 0.431488 | 0.354026 | 0.46984 | 0.569445 | 0.644212 | 0.494574 | 0.310245 | 0.315604 | 0.133928 | 0.48883 | 0.147025 | 0.489335 | 0.252132 | 0.161715 | 0.465643 | 0.278692 | 0.566645 | 0.421059 | 0.276464 | 0.094092 | 0.524308 | 0.366808 | 0.313203 | 0.330647 | 0.282746 | 0.285207 |
| TCGA-AG-3594-01A-02R-0821-07 | 0.086975 | 0.516914 | 0.607462 | 0.418779 | 0.481155 | 0.622981 | 0.672626 | 0.534947 | 0.317923 | 0.465139 | 0.140315 | 0.520621 | 0.191067 | 0.54363 | 0.310013 | 0.216506 | 0.580981 | 0.252488 | 0.553453 | 0.454462 | 0.281002 | 0.270262 | 0.553742 | 0.40523 | 0.346344 | 0.359395 | 0.35846 | 0.386358 |
| TCGA-EI-7004-01A-11R-1928-07 | 0.160397 | 0.413298 | 0.411917 | 0.393447 | 0.443811 | 0.590567 | 0.697497 | 0.568802 | 0.423542 | 0.429801 | 0.166574 | 0.541005 | 0.23436 | 0.534119 | 0.360571 | 0.332399 | 0.602233 | 0.27112 | 0.557425 | 0.530395 | 0.343253 | 0.205323 | 0.563808 | 0.526384 | 0.386027 | 0.38835 | 0.24456 | 0.374586 |
| TCGA-F5-6863-01A-11R-1928-07 | -0.09727 | 0.303028 | 0.330899 | 0.276733 | 0.478314 | 0.536258 | 0.62609 | 0.423803 | 0.315035 | 0.226212 | 0.059399 | 0.413248 | 0.003915 | 0.433067 | 0.131302 | 0.030439 | 0.303557 | 0.261814 | 0.520243 | 0.459757 | 0.202286 | -0.12309 | 0.530476 | 0.237963 | 0.275694 | 0.277408 | 0.219146 | 0.284628 |
| TCGA-F5-6814-01A-31R-1928-07 | 0.039747 | 0.465819 | 0.518767 | 0.369315 | 0.465647 | 0.5101 | 0.644918 | 0.526098 | 0.343816 | 0.361119 | 0.084088 | 0.484776 | 0.172991 | 0.475174 | 0.207239 | 0.173733 | 0.508794 | 0.266374 | 0.534642 | 0.446644 | 0.292157 | 0.022723 | 0.517534 | 0.385498 | 0.353962 | 0.34195 | 0.218236 | 0.350044 |
| TCGA-D5-6532-01A-11R-1723-07 | -0.00066 | 0.362777 | 0.394582 | 0.297968 | 0.45178 | 0.531865 | 0.606115 | 0.467513 | 0.320229 | 0.226722 | 0.048477 | 0.440091 | 0.056877 | 0.459083 | 0.126766 | 0.120418 | 0.339598 | 0.291626 | 0.566448 | 0.410392 | 0.189385 | 0.036148 | 0.508773 | 0.254051 | 0.245423 | 0.272952 | 0.237604 | 0.263015 |
| TCGA-DM-A0XD-01A-12R-A155-07 | -0.11245 | 0.403745 | 0.388588 | 0.311603 | 0.460956 | 0.577199 | 0.608421 | 0.490319 | 0.319069 | 0.298654 | 0.040139 | 0.505766 | 0.045275 | 0.439358 | 0.185452 | 0.136868 | 0.364851 | 0.274501 | 0.551215 | 0.399046 | 0.241223 | 0.070921 | 0.504456 | 0.296152 | 0.277353 | 0.278731 | 0.227095 | 0.278724 |
| TCGA-AA-3492-01A-01R-1410-07 | 0.033558 | 0.439767 | 0.514699 | 0.388542 | 0.447446 | 0.584911 | 0.642896 | 0.511225 | 0.288356 | 0.390201 | 0.101695 | 0.50373 | 0.170779 | 0.4785 | 0.192866 | 0.140804 | 0.493907 | 0.233768 | 0.615339 | 0.450364 | 0.294855 | 0.192108 | 0.511514 | 0.391734 | 0.347522 | 0.339919 | 0.286085 | 0.285074 |
| TCGA-AA-A022-01A-21R-A16W-07 | 0.064112 | 0.449931 | 0.613535 | 0.408557 | 0.434583 | 0.643843 | 0.673093 | 0.511296 | 0.260575 | 0.453644 | 0.086511 | 0.489277 | 0.230651 | 0.430767 | 0.255298 | 0.209392 | 0.541425 | 0.232545 | 0.558953 | 0.487329 | 0.312846 | 0.049116 | 0.492946 | 0.362671 | 0.387528 | 0.335679 | 0.34288 | 0.309633 |
| TCGA-AD-6899-01A-11R-1928-07 | 0.148136 | 0.45715 | 0.514087 | 0.414093 | 0.459977 | 0.575947 | 0.68284 | 0.528225 | 0.357802 | 0.413525 | 0.137922 | 0.512782 | 0.245827 | 0.508594 | 0.259998 | 0.252214 | 0.574376 | 0.302872 | 0.521734 | 0.480933 | 0.303151 | 0.040126 | 0.556876 | 0.480506 | 0.365643 | 0.386505 | 0.258763 | 0.29854 |
| TCGA-AA-3844-01A-01R-1022-07 | -0.02556 | 0.489516 | 0.482983 | 0.385515 | 0.456715 | 0.569254 | 0.629118 | 0.472943 | 0.276006 | 0.336944 | 0.146216 | 0.46572 | 0.103362 | 0.503874 | 0.200782 | 0.134344 | 0.440615 | 0.231627 | 0.557638 | 0.399082 | 0.231023 | 0.11965 | 0.522602 | 0.316394 | 0.288757 | 0.33081 | 0.365582 | 0.365881 |
| TCGA-AD-6901-01A-11R-1928-07 | 0.008894 | 0.344028 | 0.482011 | 0.393253 | 0.47533 | 0.559807 | 0.685359 | 0.544242 | 0.352325 | 0.38034 | 0.143386 | 0.536898 | 0.153273 | 0.495796 | 0.295623 | 0.285063 | 0.54283 | 0.269921 | 0.540613 | 0.478487 | 0.293987 | -0.03309 | 0.521106 | 0.435286 | 0.347827 | 0.363911 | 0.228689 | 0.241636 |
| TCGA-AA-A01P-01A-21R-A083-07 | 0.246155 | 0.535087 | 0.613249 | 0.487684 | 0.457561 | 0.572353 | 0.71422 | 0.577674 | 0.344215 | 0.511957 | 0.102116 | 0.529351 | 0.331363 | 0.504256 | 0.333524 | 0.173391 | 0.614866 | 0.304236 | 0.549204 | 0.535419 | 0.319784 | 0.20029 | 0.521731 | 0.444806 | 0.39978 | 0.424596 | 0.322867 | 0.414227 |
| TCGA-G4-6293-01A-11R-1723-07 | 0.160881 | 0.429758 | 0.519891 | 0.401851 | 0.467466 | 0.566592 | 0.671982 | 0.48264 | 0.315739 | 0.364955 | 0.149899 | 0.492249 | 0.214412 | 0.482362 | 0.205707 | 0.199197 | 0.50189 | 0.259789 | 0.555659 | 0.437611 | 0.225283 | 0.163281 | 0.520636 | 0.372139 | 0.335091 | 0.357174 | 0.332616 | 0.293122 |
| TCGA-AA-3715-01A-01R-0905-07 | 0.1374 | 0.46678 | 0.560862 | 0.455447 | 0.485594 | 0.645853 | 0.704155 | 0.56591 | 0.298513 | 0.494327 | 0.107806 | 0.559883 | 0.242493 | 0.513982 | 0.334895 | 0.300665 | 0.628581 | 0.233 | 0.602205 | 0.496146 | 0.388509 | 0.250308 | 0.555003 | 0.543331 | 0.424452 | 0.412908 | 0.264591 | 0.375179 |
| TCGA-AY-A71X-01A-12R-A37K-07 | -0.1494 | 0.271266 | 0.335123 | 0.261617 | 0.451786 | 0.541707 | 0.623297 | 0.41795 | 0.23009 | 0.21065 | 0.047977 | 0.401865 | -0.03138 | 0.435956 | 0.094772 | 0.047978 | 0.302897 | 0.232999 | 0.521507 | 0.310612 | 0.164405 | -0.01795 | 0.467912 | 0.155117 | 0.208567 | 0.226499 | 0.2692 | 0.291216 |
| TCGA-DM-A28C-01A-11R-A32Y-07 | -0.05937 | 0.335485 | 0.325684 | 0.256326 | 0.474633 | 0.51105 | 0.610139 | 0.411405 | 0.270614 | 0.223388 | 0.117974 | 0.394614 | 0.029416 | 0.475202 | 0.139026 | 0.048471 | 0.328692 | 0.238737 | 0.532104 | 0.364416 | 0.126943 | -0.03353 | 0.455215 | 0.180345 | 0.216508 | 0.252057 | 0.236749 | 0.258724 |
| TCGA-AA-3560-01A-01R-0821-07 | 0.086447 | 0.400318 | 0.497522 | 0.398966 | 0.458977 | 0.580784 | 0.657185 | 0.492942 | 0.327708 | 0.328066 | 0.10734 | 0.495688 | 0.176923 | 0.525236 | 0.283184 | 0.201786 | 0.495799 | 0.274194 | 0.546345 | 0.443357 | 0.280696 | 0.168708 | 0.532621 | 0.357411 | 0.349981 | 0.332354 | 0.320934 | 0.306759 |
| TCGA-AA-3552-01A-01R-0821-07 | 0.182252 | 0.422763 | 0.511601 | 0.383937 | 0.475636 | 0.582345 | 0.664076 | 0.501354 | 0.315 | 0.364243 | 0.094372 | 0.50726 | 0.248718 | 0.531516 | 0.25022 | 0.256487 | 0.539111 | 0.252837 | 0.58241 | 0.440078 | 0.273694 | 0.037598 | 0.538995 | 0.401804 | 0.365036 | 0.377765 | 0.300503 | 0.249085 |
| TCGA-CM-6166-01A-11R-1653-07 | -0.16525 | 0.383713 | 0.37751 | 0.28101 | 0.474558 | 0.515777 | 0.61904 | 0.454885 | 0.300678 | 0.209267 | 0.046025 | 0.441725 | -0.02946 | 0.423211 | 0.133811 | 0.102983 | 0.268776 | 0.284864 | 0.507172 | 0.413632 | 0.186337 | -0.12152 | 0.497146 | 0.282298 | 0.242527 | 0.283215 | 0.237085 | 0.30041 |
| TCGA-AZ-5403-01A-01R-1653-07 | -0.04574 | 0.369732 | 0.401289 | 0.372507 | 0.457678 | 0.541284 | 0.639228 | 0.499189 | 0.362363 | 0.247978 | 0.123067 | 0.52267 | 0.094385 | 0.51628 | 0.251883 | 0.252318 | 0.431471 | 0.27818 | 0.561195 | 0.450116 | 0.278221 | 0.070199 | 0.531634 | 0.355329 | 0.306071 | 0.354506 | 0.272389 | 0.289611 |
| TCGA-G4-6299-01A-11R-1774-07 | 0.084504 | 0.491027 | 0.553984 | 0.389806 | 0.462982 | 0.559951 | 0.706939 | 0.501486 | 0.37519 | 0.430426 | 0.076415 | 0.514775 | 0.215244 | 0.510052 | 0.26499 | 0.177614 | 0.546618 | 0.285417 | 0.531827 | 0.489476 | 0.305135 | -0.05343 | 0.497653 | 0.477754 | 0.371701 | 0.362793 | 0.26171 | 0.379062 |
| TCGA-AA-3496-01A-21R-1839-07 | 0.145028 | 0.427631 | 0.502435 | 0.391012 | 0.442356 | 0.551376 | 0.6771 | 0.542817 | 0.35544 | 0.407536 | 0.141524 | 0.498941 | 0.202366 | 0.521926 | 0.290089 | 0.257522 | 0.587346 | 0.299675 | 0.533072 | 0.492195 | 0.270067 | 0.118202 | 0.558846 | 0.451824 | 0.352489 | 0.355851 | 0.247343 | 0.275763 |
| TCGA-A6-5661-01A-01R-1653-07 | 0.082176 | 0.483526 | 0.459485 | 0.388985 | 0.420119 | 0.509324 | 0.624673 | 0.522986 | 0.301575 | 0.382743 | 0.128078 | 0.47244 | 0.184744 | 0.47636 | 0.200498 | 0.144394 | 0.448729 | 0.261524 | 0.532503 | 0.431539 | 0.265085 | 0.152163 | 0.480173 | 0.330776 | 0.32685 | 0.345365 | 0.282966 | 0.367437 |
| TCGA-G4-6304-01A-11R-1928-07 | -0.11278 | 0.393818 | 0.445541 | 0.382447 | 0.45866 | 0.529448 | 0.615326 | 0.47037 | 0.250831 | 0.333057 | 0.012549 | 0.469664 | 0.042048 | 0.454269 | 0.175924 | 0.045272 | 0.418063 | 0.218851 | 0.535157 | 0.398663 | 0.188077 | -0.01679 | 0.484272 | 0.285382 | 0.287502 | 0.276438 | 0.271052 | 0.247305 |
| TCGA-CM-5864-01A-01R-1653-07 | 0.048065 | 0.435812 | 0.442309 | 0.330948 | 0.471635 | 0.518005 | 0.622661 | 0.458023 | 0.309478 | 0.253744 | 0.066058 | 0.450703 | 0.106302 | 0.483487 | 0.172668 | 0.116168 | 0.393498 | 0.284168 | 0.562579 | 0.399457 | 0.207228 | 0.045205 | 0.524824 | 0.309991 | 0.268565 | 0.29503 | 0.28302 | 0.337404 |
| TCGA-AY-A8YK-01A-11R-A41B-07 | 0.037634 | 0.376081 | 0.386177 | 0.340766 | 0.436887 | 0.557883 | 0.633422 | 0.438255 | 0.292228 | 0.232707 | 0.097797 | 0.461615 | 0.095554 | 0.452761 | 0.154614 | 0.094024 | 0.350101 | 0.312421 | 0.54123 | 0.40652 | 0.217473 | 0.073467 | 0.536841 | 0.257428 | 0.254897 | 0.294369 | 0.271982 | 0.243 |
| TCGA-DM-A28K-01A-21R-A32Y-07 | -0.20327 | 0.357796 | 0.383849 | 0.319838 | 0.413864 | 0.576175 | 0.639784 | 0.412123 | 0.327903 | 0.270729 | 0.084165 | 0.468559 | 0.003788 | 0.483649 | 0.160223 | 0.05933 | 0.399533 | 0.191974 | 0.494936 | 0.34292 | 0.213795 | 0.025797 | 0.509425 | 0.23071 | 0.223898 | 0.222842 | 0.242699 | 0.360109 |
| TCGA-CK-5913-01A-11R-1653-07 | 0.006636 | 0.498647 | 0.496668 | 0.4413 | 0.423808 | 0.532297 | 0.654643 | 0.566341 | 0.361473 | 0.419822 | 0.126936 | 0.492853 | 0.166397 | 0.505941 | 0.280837 | 0.208006 | 0.49383 | 0.312184 | 0.5602 | 0.471643 | 0.284176 | 0.212028 | 0.51913 | 0.393193 | 0.35988 | 0.36657 | 0.302743 | 0.386217 |
| TCGA-AZ-6605-01A-11R-1839-07 | 0.180352 | 0.441626 | 0.514576 | 0.406525 | 0.475955 | 0.558376 | 0.69097 | 0.546336 | 0.398429 | 0.403393 | 0.136378 | 0.526423 | 0.268694 | 0.500443 | 0.294941 | 0.277809 | 0.563355 | 0.30929 | 0.554105 | 0.492273 | 0.298938 | 0.102787 | 0.572902 | 0.475218 | 0.354819 | 0.391722 | 0.286153 | 0.321428 |
| TCGA-CM-4751-01A-02R-1839-07 | 0.179687 | 0.48607 | 0.526765 | 0.384319 | 0.456778 | 0.572563 | 0.676017 | 0.511226 | 0.360876 | 0.3983 | 0.174349 | 0.525078 | 0.23902 | 0.479305 | 0.239495 | 0.220781 | 0.509563 | 0.286966 | 0.552058 | 0.442033 | 0.282379 | 0.081891 | 0.519658 | 0.369891 | 0.354351 | 0.35673 | 0.245667 | 0.340807 |
| TCGA-CM-6677-01A-11R-1839-07 | 0.08182 | 0.46093 | 0.469701 | 0.374582 | 0.435999 | 0.542092 | 0.642379 | 0.47717 | 0.338105 | 0.323526 | 0.075422 | 0.505463 | 0.179261 | 0.45715 | 0.226446 | 0.203639 | 0.46071 | 0.290687 | 0.546456 | 0.452536 | 0.269537 | 0.051217 | 0.53523 | 0.380152 | 0.314874 | 0.339828 | 0.225303 | 0.291357 |
| TCGA-G4-6627-01A-11R-1774-07 | 0.245802 | 0.510516 | 0.542204 | 0.404719 | 0.458605 | 0.553712 | 0.664423 | 0.552972 | 0.410249 | 0.404867 | 0.257577 | 0.538192 | 0.289544 | 0.490865 | 0.283333 | 0.345388 | 0.525091 | 0.318902 | 0.550953 | 0.501693 | 0.312792 | 0.17302 | 0.567028 | 0.414843 | 0.362125 | 0.411537 | 0.273958 | 0.3528 |
| TCGA-CI-6622-01A-11R-1830-07 | -0.01333 | 0.36842 | 0.360329 | 0.325487 | 0.466956 | 0.545178 | 0.610036 | 0.476646 | 0.31746 | 0.224763 | 0.163508 | 0.469045 | 0.085783 | 0.46914 | 0.18875 | 0.244016 | 0.362262 | 0.284906 | 0.57502 | 0.412321 | 0.235504 | 0.122686 | 0.509096 | 0.299521 | 0.296216 | 0.318369 | 0.255145 | 0.278033 |
| TCGA-F5-6861-01A-11R-1928-07 | -0.03419 | 0.393594 | 0.419273 | 0.33057 | 0.468205 | 0.525138 | 0.631831 | 0.46676 | 0.29558 | 0.251963 | 0.112295 | 0.465851 | 0.05078 | 0.442889 | 0.16633 | 0.121854 | 0.396942 | 0.272431 | 0.526408 | 0.445009 | 0.221833 | 0.018688 | 0.505873 | 0.353613 | 0.286768 | 0.317859 | 0.276139 | 0.323785 |
| TCGA-AG-A036-01A-12R-A083-07 | 0.002044 | 0.427491 | 0.453304 | 0.334868 | 0.464354 | 0.52993 | 0.626863 | 0.475967 | 0.332391 | 0.278476 | 0.072797 | 0.473791 | 0.094257 | 0.486136 | 0.148066 | 0.088742 | 0.390193 | 0.23453 | 0.55784 | 0.418504 | 0.272138 | -0.03044 | 0.485917 | 0.322127 | 0.278576 | 0.313301 | 0.259472 | 0.277989 |
| TCGA-AG-3898-01A-01R-1119-07 | -0.01657 | 0.480972 | 0.445873 | 0.377431 | 0.448528 | 0.554037 | 0.623726 | 0.517955 | 0.336699 | 0.347661 | 0.065683 | 0.50278 | 0.132565 | 0.491801 | 0.228597 | 0.168449 | 0.497332 | 0.250627 | 0.546202 | 0.447148 | 0.269476 | 0.19953 | 0.518098 | 0.427641 | 0.308429 | 0.335666 | 0.239343 | 0.2679 |
| TCGA-AG-A011-01A-01R-A002-07 | 0.118511 | 0.350509 | 0.438787 | 0.306151 | 0.460523 | 0.593356 | 0.621544 | 0.47311 | 0.298134 | 0.278123 | 0.055481 | 0.446464 | 0.145947 | 0.461556 | 0.185052 | 0.177292 | 0.410461 | 0.259175 | 0.550109 | 0.409403 | 0.202605 | 0.019742 | 0.517074 | 0.303376 | 0.311842 | 0.292791 | 0.281114 | 0.260591 |
| TCGA-AG-3575-01A-01R-0821-07 | 0.009943 | 0.491698 | 0.494436 | 0.400662 | 0.446204 | 0.510521 | 0.65693 | 0.516887 | 0.331449 | 0.421496 | 0.15096 | 0.552209 | 0.181348 | 0.544984 | 0.289004 | 0.22397 | 0.522074 | 0.246436 | 0.538038 | 0.477033 | 0.317392 | 0.109665 | 0.5165 | 0.47278 | 0.332555 | 0.354063 | 0.264224 | 0.376212 |
| TCGA-DC-6683-01A-11R-1830-07 | 0.038533 | 0.38413 | 0.400464 | 0.357743 | 0.465297 | 0.563666 | 0.634421 | 0.519463 | 0.34446 | 0.277518 | 0.102669 | 0.478622 | 0.183213 | 0.46576 | 0.19625 | 0.204382 | 0.41927 | 0.327012 | 0.530824 | 0.460187 | 0.253245 | -0.05468 | 0.513675 | 0.370849 | 0.295785 | 0.333861 | 0.249028 | 0.295543 |
| TCGA-EI-6882-01A-11R-1928-07 | 0.072245 | 0.517276 | 0.45074 | 0.374681 | 0.441455 | 0.536296 | 0.665411 | 0.528317 | 0.342216 | 0.37148 | 0.165973 | 0.500666 | 0.19006 | 0.503931 | 0.230604 | 0.255808 | 0.476732 | 0.306482 | 0.537278 | 0.423616 | 0.290611 | 0.151073 | 0.530735 | 0.372198 | 0.299371 | 0.315944 | 0.27838 | 0.398301 |
| TCGA-DT-5265-01A-21R-1830-07 | -0.11791 | 0.337273 | 0.407981 | 0.399533 | 0.456869 | 0.56934 | 0.69401 | 0.553004 | 0.32529 | 0.375583 | 0.120039 | 0.530139 | 0.109015 | 0.506093 | 0.290549 | 0.173808 | 0.521213 | 0.249995 | 0.54914 | 0.475943 | 0.286183 | 0.052207 | 0.55646 | 0.453814 | 0.347426 | 0.331811 | 0.214112 | 0.402825 |
| TCGA-DY-A0XA-01A-11R-A155-07 | -0.05436 | 0.417548 | 0.427302 | 0.309109 | 0.472512 | 0.514263 | 0.616434 | 0.48081 | 0.268855 | 0.243195 | 0.109379 | 0.442373 | 0.07372 | 0.428803 | 0.138038 | 0.154572 | 0.366469 | 0.261631 | 0.501593 | 0.406221 | 0.178533 | -0.00823 | 0.497616 | 0.248828 | 0.247614 | 0.279414 | 0.209424 | 0.210769 |
| TCGA-AG-3600-01A-01R-0826-07 | 0.059958 | 0.467375 | 0.491494 | 0.37091 | 0.457299 | 0.584314 | 0.63125 | 0.530918 | 0.293655 | 0.337453 | 0.127466 | 0.510551 | 0.17542 | 0.491597 | 0.271474 | 0.250385 | 0.50681 | 0.25861 | 0.555305 | 0.450885 | 0.301241 | 0.064104 | 0.491828 | 0.388623 | 0.36383 | 0.346031 | 0.252782 | 0.373309 |
| TCGA-DC-5869-01A-01R-1660-07 | 0.014883 | 0.460448 | 0.441432 | 0.345574 | 0.442735 | 0.496497 | 0.616569 | 0.456349 | 0.33146 | 0.287655 | 0.160209 | 0.462763 | 0.109845 | 0.461729 | 0.168752 | 0.18043 | 0.37413 | 0.306289 | 0.518078 | 0.420884 | 0.254406 | -0.07612 | 0.537613 | 0.34706 | 0.288839 | 0.335975 | 0.234352 | 0.29499 |
| TCGA-CL-5918-01A-11R-1660-07 | -0.14088 | 0.391076 | 0.457844 | 0.333351 | 0.476292 | 0.536246 | 0.612562 | 0.445705 | 0.268249 | 0.23848 | 0.096434 | 0.446572 | 0.019655 | 0.443687 | 0.105266 | 0.124175 | 0.337804 | 0.27525 | 0.555133 | 0.385592 | 0.22935 | 0.099612 | 0.514398 | 0.264401 | 0.238941 | 0.288964 | 0.235169 | 0.259564 |
| TCGA-DY-A1DG-01A-11R-A32Y-07 | -0.15549 | 0.372674 | 0.386322 | 0.299619 | 0.44835 | 0.527154 | 0.591914 | 0.367427 | 0.265922 | 0.196725 | 0.029649 | 0.43714 | -0.04264 | 0.422445 | 0.06384 | 0.094235 | 0.269456 | 0.28656 | 0.51657 | 0.355255 | 0.198431 | 0.015541 | 0.484713 | 0.191002 | 0.228394 | 0.224679 | 0.184022 | 0.214539 |
| TCGA-CL-4957-01A-01R-1736-07 | -0.00752 | 0.397315 | 0.392297 | 0.308218 | 0.4465 | 0.546396 | 0.607184 | 0.464225 | 0.319763 | 0.256208 | 0.075377 | 0.45951 | 0.061836 | 0.472095 | 0.159249 | 0.085095 | 0.362928 | 0.291672 | 0.534619 | 0.417165 | 0.207518 | 0.051263 | 0.512178 | 0.310636 | 0.280082 | 0.307513 | 0.258361 | 0.318886 |
| TCGA-AG-3586-01A-02R-0821-07 | -0.00031 | 0.39625 | 0.482925 | 0.338292 | 0.456589 | 0.579015 | 0.624613 | 0.486782 | 0.296733 | 0.280993 | 0.088825 | 0.490969 | 0.107589 | 0.490765 | 0.215458 | 0.178497 | 0.439481 | 0.248397 | 0.563096 | 0.412948 | 0.253816 | 0.077371 | 0.530492 | 0.353915 | 0.34408 | 0.360811 | 0.28148 | 0.278328 |
| TCGA-DC-4749-01A-01R-1736-07 | -0.03498 | 0.424757 | 0.41984 | 0.338188 | 0.447689 | 0.517752 | 0.623219 | 0.450776 | 0.286817 | 0.262454 | 0.105089 | 0.454902 | 0.080046 | 0.451665 | 0.14593 | 0.073933 | 0.356499 | 0.254059 | 0.549307 | 0.38916 | 0.23475 | 0.068896 | 0.473755 | 0.285155 | 0.259411 | 0.305922 | 0.294459 | 0.260776 |
| TCGA-G5-6235-01A-11R-1736-07 | 0.068146 | 0.401313 | 0.415292 | 0.304735 | 0.451407 | 0.532367 | 0.60198 | 0.437353 | 0.314675 | 0.238852 | 0.079677 | 0.425988 | 0.147 | 0.481344 | 0.095596 | 0.099631 | 0.315788 | 0.309226 | 0.565727 | 0.362121 | 0.165546 | 0.063088 | 0.497428 | 0.20046 | 0.216404 | 0.279344 | 0.280845 | 0.294717 |
| TCGA-AG-3612-01A-01R-0826-07 | 0.08486 | 0.424498 | 0.455506 | 0.373409 | 0.468997 | 0.580347 | 0.645623 | 0.492689 | 0.300748 | 0.297392 | 0.11173 | 0.500896 | 0.179232 | 0.511916 | 0.23114 | 0.259555 | 0.495908 | 0.301885 | 0.556639 | 0.432581 | 0.294983 | 0.075014 | 0.532848 | 0.402498 | 0.355802 | 0.357901 | 0.243442 | 0.250764 |
| TCGA-AG-3725-01A-11R-1736-07 | 0.04313 | 0.405415 | 0.460025 | 0.362698 | 0.446409 | 0.547195 | 0.632473 | 0.459376 | 0.305675 | 0.303834 | 0.108613 | 0.45744 | 0.112019 | 0.432457 | 0.1489 | 0.13589 | 0.389215 | 0.219264 | 0.546292 | 0.404096 | 0.247723 | 0.065492 | 0.494806 | 0.285255 | 0.291177 | 0.304848 | 0.29704 | 0.27924 |
| TCGA-EI-6509-01A-11R-1736-07 | -0.20497 | 0.312865 | 0.304736 | 0.328471 | 0.441809 | 0.562261 | 0.633437 | 0.473044 | 0.294528 | 0.233241 | 0.046938 | 0.460691 | -0.02403 | 0.45353 | 0.198385 | 0.074247 | 0.389624 | 0.234267 | 0.524396 | 0.421483 | 0.204983 | -0.01274 | 0.515097 | 0.313096 | 0.284951 | 0.26943 | 0.212935 | 0.261522 |
| TCGA-AH-6547-01A-11R-1830-07 | 0.406032 | 0.454868 | 0.541799 | 0.435269 | 0.446333 | 0.573518 | 0.700948 | 0.579562 | 0.351759 | 0.426863 | 0.244007 | 0.557627 | 0.399407 | 0.514112 | 0.350782 | 0.317809 | 0.615626 | 0.324426 | 0.583964 | 0.495682 | 0.315706 | 0.17963 | 0.58111 | 0.522852 | 0.383953 | 0.414046 | 0.297925 | 0.323063 |
| TCGA-AF-6672-01A-11R-1830-07 | 0.110855 | 0.349543 | 0.423825 | 0.338112 | 0.440199 | 0.557991 | 0.656396 | 0.438464 | 0.334907 | 0.289025 | 0.124796 | 0.430319 | 0.167594 | 0.486429 | 0.212917 | 0.164514 | 0.402791 | 0.278119 | 0.508631 | 0.396767 | 0.188002 | 0.006334 | 0.540567 | 0.240845 | 0.274282 | 0.298815 | 0.273736 | 0.308639 |
| TCGA-AG-A01N-01A-01R-A00A-07 | -0.07512 | 0.323532 | 0.351962 | 0.308349 | 0.417952 | 0.554876 | 0.604708 | 0.45624 | 0.231971 | 0.183213 | 0.032211 | 0.435246 | -0.01112 | 0.436777 | 0.144557 | 0.098509 | 0.321295 | 0.25669 | 0.55326 | 0.380302 | 0.1875 | 0.100595 | 0.504469 | 0.26214 | 0.261235 | 0.289145 | 0.275309 | 0.220169 |
| TCGA-AF-2691-01A-01R-0821-07 | 0.114224 | 0.427943 | 0.528666 | 0.373963 | 0.456239 | 0.565421 | 0.661978 | 0.52059 | 0.31997 | 0.331793 | 0.215882 | 0.486295 | 0.197411 | 0.514425 | 0.275932 | 0.273456 | 0.538694 | 0.276109 | 0.555947 | 0.449117 | 0.273982 | 0.07388 | 0.54578 | 0.396391 | 0.356176 | 0.375142 | 0.300786 | 0.331111 |
| TCGA-AG-3883-01A-02R-0905-07 | -0.00068 | 0.377949 | 0.496117 | 0.422423 | 0.467831 | 0.618497 | 0.645485 | 0.52924 | 0.300442 | 0.347202 | 0.162339 | 0.532829 | 0.130055 | 0.497112 | 0.329463 | 0.29244 | 0.565837 | 0.267146 | 0.562702 | 0.44385 | 0.312304 | 0.152881 | 0.528635 | 0.491029 | 0.373102 | 0.386198 | 0.304815 | 0.25075 |
| TCGA-DC-5337-01A-01R-1660-07 | 0.027166 | 0.492745 | 0.506303 | 0.39018 | 0.45036 | 0.535745 | 0.62132 | 0.456393 | 0.333066 | 0.335802 | 0.091347 | 0.49961 | 0.090135 | 0.461752 | 0.197241 | 0.123114 | 0.456379 | 0.28732 | 0.545691 | 0.418192 | 0.22669 | 0.104927 | 0.514067 | 0.350366 | 0.298538 | 0.366401 | 0.300184 | 0.327824 |
| TCGA-AG-3592-01A-02R-1736-07 | 0.057712 | 0.450883 | 0.458519 | 0.387338 | 0.44285 | 0.544476 | 0.618988 | 0.501285 | 0.344436 | 0.304056 | 0.092758 | 0.499107 | 0.130263 | 0.470236 | 0.191806 | 0.162402 | 0.456399 | 0.285627 | 0.547443 | 0.443876 | 0.259011 | 0.07237 | 0.495618 | 0.348684 | 0.283224 | 0.320175 | 0.270594 | 0.292197 |
| TCGA-AF-2690-01A-02R-1736-07 | 0.245533 | 0.513455 | 0.590002 | 0.430449 | 0.495985 | 0.547353 | 0.702803 | 0.609544 | 0.397999 | 0.467927 | 0.247441 | 0.586266 | 0.312551 | 0.523679 | 0.384056 | 0.451051 | 0.67404 | 0.331658 | 0.583182 | 0.530387 | 0.350307 | 0.176769 | 0.575744 | 0.583748 | 0.436307 | 0.4346 | 0.248716 | 0.333626 |
| TCGA-F5-6811-01A-11R-1830-07 | 0.045342 | 0.391522 | 0.449287 | 0.387097 | 0.446707 | 0.537971 | 0.665942 | 0.538568 | 0.330052 | 0.334822 | 0.069954 | 0.516575 | 0.188758 | 0.471826 | 0.243996 | 0.182099 | 0.470272 | 0.301891 | 0.528213 | 0.484765 | 0.291693 | 0.092314 | 0.544655 | 0.411507 | 0.334614 | 0.361121 | 0.234375 | 0.303161 |
| TCGA-AF-3400-01A-01R-0821-07 | 0.065952 | 0.523453 | 0.511774 | 0.515372 | 0.471772 | 0.599002 | 0.700603 | 0.622653 | 0.401277 | 0.429937 | 0.288137 | 0.585615 | 0.227571 | 0.586537 | 0.440671 | 0.397509 | 0.683569 | 0.285665 | 0.579683 | 0.518837 | 0.394812 | 0.441365 | 0.598448 | 0.609677 | 0.447105 | 0.419446 | 0.341303 | 0.362039 |
| TCGA-AG-A008-01A-01R-A002-07 | -0.17812 | 0.384482 | 0.430587 | 0.340213 | 0.443162 | 0.544793 | 0.620938 | 0.437277 | 0.249417 | 0.250195 | 0.068672 | 0.425462 | -0.0557 | 0.470155 | 0.176047 | 0.118151 | 0.398143 | 0.228332 | 0.535968 | 0.350889 | 0.170874 | 0.037042 | 0.502547 | 0.254387 | 0.252985 | 0.268197 | 0.333998 | 0.320738 |
| TCGA-AA-3544-01A-01R-1873-07 | 0.196594 | 0.448151 | 0.593052 | 0.395938 | 0.490547 | 0.574116 | 0.663754 | 0.530527 | 0.329177 | 0.376201 | 0.13571 | 0.549702 | 0.229515 | 0.520197 | 0.336707 | 0.339935 | 0.586388 | 0.249871 | 0.563131 | 0.457649 | 0.365306 | 0.109014 | 0.522592 | 0.469447 | 0.399252 | 0.385686 | 0.298785 | 0.30427 |
| TCGA-A6-A567-01A-31R-A28H-07 | -0.00851 | 0.339562 | 0.333775 | 0.290444 | 0.416995 | 0.513607 | 0.633488 | 0.434063 | 0.314695 | 0.207457 | 0.052329 | 0.428829 | 0.076931 | 0.511224 | 0.15415 | 0.115609 | 0.321399 | 0.308569 | 0.525346 | 0.425003 | 0.226897 | -0.01015 | 0.523749 | 0.310439 | 0.250169 | 0.29948 | 0.199814 | 0.240628 |
| TCGA-CM-6676-01A-11R-1839-07 | -0.11989 | 0.359213 | 0.393519 | 0.317196 | 0.456504 | 0.545275 | 0.607003 | 0.463031 | 0.323417 | 0.240948 | 0.122684 | 0.456346 | 0.019845 | 0.481598 | 0.148765 | 0.179763 | 0.332274 | 0.298494 | 0.53187 | 0.44418 | 0.216688 | -0.00576 | 0.518274 | 0.29925 | 0.285249 | 0.296249 | 0.252994 | 0.292302 |
| TCGA-AA-3867-01A-01R-1022-07 | 0.128575 | 0.355685 | 0.41037 | 0.320678 | 0.481012 | 0.558726 | 0.635838 | 0.525608 | 0.341883 | 0.34593 | 0.108851 | 0.523682 | 0.195386 | 0.477658 | 0.246228 | 0.218515 | 0.456442 | 0.311156 | 0.552265 | 0.449798 | 0.277427 | -0.03227 | 0.532835 | 0.400258 | 0.359531 | 0.354778 | 0.244162 | 0.240948 |
| TCGA-CA-6719-01A-11R-1839-07 | 0.078323 | 0.405091 | 0.457848 | 0.38773 | 0.472922 | 0.556053 | 0.666262 | 0.53592 | 0.353133 | 0.340801 | 0.140838 | 0.509332 | 0.192073 | 0.482635 | 0.238973 | 0.214325 | 0.515105 | 0.264736 | 0.554852 | 0.480197 | 0.296905 | 0.013464 | 0.521675 | 0.443909 | 0.355254 | 0.372814 | 0.221569 | 0.27661 |
| TCGA-NH-A50V-01A-11R-A28H-07 | 0.069167 | 0.399749 | 0.448361 | 0.40834 | 0.447468 | 0.509719 | 0.670894 | 0.510086 | 0.318476 | 0.374897 | 0.149296 | 0.439694 | 0.178025 | 0.511006 | 0.218087 | 0.148458 | 0.523378 | 0.247761 | 0.519695 | 0.475854 | 0.271804 | 0.031699 | 0.521796 | 0.364396 | 0.284782 | 0.351472 | 0.321656 | 0.316194 |
| TCGA-AA-A01C-01A-01R-A00A-07 | -0.07347 | 0.377739 | 0.417797 | 0.302125 | 0.450607 | 0.579566 | 0.634121 | 0.472591 | 0.287066 | 0.284587 | 0.080963 | 0.478348 | 0.048124 | 0.436329 | 0.192594 | 0.128932 | 0.416103 | 0.234526 | 0.530966 | 0.442021 | 0.235458 | -0.09451 | 0.528544 | 0.338206 | 0.276609 | 0.29754 | 0.241872 | 0.227029 |
| TCGA-AA-A02F-01A-01R-A089-07 | -0.22994 | 0.385774 | 0.317992 | 0.289091 | 0.437901 | 0.522309 | 0.619731 | 0.438684 | 0.257147 | 0.198513 | 0.110957 | 0.418891 | -0.08244 | 0.472926 | 0.128003 | 0.07846 | 0.317567 | 0.221753 | 0.524229 | 0.395906 | 0.218161 | -0.02426 | 0.500003 | 0.226511 | 0.258841 | 0.273183 | 0.196509 | 0.277724 |
| TCGA-AA-A03F-01A-11R-A16W-07 | 0.087865 | 0.300163 | 0.415393 | 0.330425 | 0.438517 | 0.63292 | 0.637738 | 0.447053 | 0.171934 | 0.321679 | 0.131358 | 0.44618 | 0.117628 | 0.39839 | 0.106965 | 0.041367 | 0.446405 | 0.183612 | 0.553112 | 0.379954 | 0.185404 | 0.075486 | 0.486659 | 0.287822 | 0.283801 | 0.3005 | 0.318992 | 0.253618 |
| TCGA-AA-A00O-01A-02R-A089-07 | -0.06588 | 0.269052 | 0.382858 | 0.32676 | 0.481131 | 0.531116 | 0.658253 | 0.471811 | 0.309586 | 0.318927 | 0.049385 | 0.451269 | 0.053201 | 0.500668 | 0.154665 | 0.085901 | 0.455544 | 0.25167 | 0.571882 | 0.463092 | 0.234195 | -0.00442 | 0.539312 | 0.355275 | 0.350626 | 0.307067 | 0.248991 | 0.260441 |
| TCGA-AZ-4615-01A-01R-1410-07 | 0.052909 | 0.543852 | 0.61886 | 0.418912 | 0.461834 | 0.589885 | 0.684705 | 0.560507 | 0.345465 | 0.434042 | 0.120686 | 0.510921 | 0.211981 | 0.463929 | 0.255292 | 0.247044 | 0.569987 | 0.291326 | 0.547935 | 0.48059 | 0.299884 | 0.023459 | 0.460462 | 0.444022 | 0.333542 | 0.374373 | 0.263031 | 0.408001 |
| TCGA-AA-3854-01A-01R-0905-07 | 0.016025 | 0.374718 | 0.418024 | 0.370919 | 0.447467 | 0.579736 | 0.646462 | 0.518484 | 0.277986 | 0.29077 | 0.097261 | 0.495676 | 0.129778 | 0.508889 | 0.232125 | 0.210775 | 0.474951 | 0.272597 | 0.546832 | 0.403098 | 0.233706 | 0.110666 | 0.527043 | 0.340284 | 0.331068 | 0.303533 | 0.346293 | 0.332907 |
| TCGA-AA-A01V-01A-23R-A083-07 | -0.05017 | 0.372794 | 0.484639 | 0.365675 | 0.479025 | 0.580198 | 0.630443 | 0.451204 | 0.260107 | 0.318058 | 0.093461 | 0.461969 | 0.055547 | 0.46287 | 0.164376 | 0.133132 | 0.4135 | 0.242539 | 0.561724 | 0.388563 | 0.188473 | 0.043887 | 0.510137 | 0.286476 | 0.301875 | 0.312785 | 0.411446 | 0.303977 |
| TCGA-AA-A00D-01A-01R-A002-07 | 0.195234 | 0.501013 | 0.583472 | 0.422313 | 0.457773 | 0.58034 | 0.68613 | 0.531355 | 0.379115 | 0.438015 | 0.153846 | 0.52597 | 0.282681 | 0.513453 | 0.299004 | 0.241019 | 0.596164 | 0.264535 | 0.604741 | 0.479253 | 0.314677 | 0.112123 | 0.534371 | 0.462287 | 0.3847 | 0.383697 | 0.346565 | 0.340871 |
| TCGA-A6-5657-01A-01R-A32Z-07 | 0.340215 | 0.475812 | 0.507777 | 0.389243 | 0.434335 | 0.532116 | 0.657859 | 0.505182 | 0.380267 | 0.362843 | 0.162881 | 0.504884 | 0.330563 | 0.469796 | 0.241206 | 0.259962 | 0.479723 | 0.359682 | 0.553947 | 0.477567 | 0.300285 | 0.009988 | 0.521566 | 0.37157 | 0.330394 | 0.380625 | 0.246482 | 0.337942 |
| TCGA-AA-3522-01A-01R-0821-07 | 0.172232 | 0.44398 | 0.463313 | 0.350536 | 0.469755 | 0.547021 | 0.635774 | 0.494701 | 0.345542 | 0.341679 | 0.133988 | 0.484191 | 0.225546 | 0.473669 | 0.226352 | 0.203938 | 0.45528 | 0.280785 | 0.558708 | 0.420817 | 0.213952 | 0.071819 | 0.487883 | 0.331902 | 0.311896 | 0.347637 | 0.357181 | 0.33395 |
| TCGA-AA-3877-01A-01R-1022-07 | 0.056994 | 0.50188 | 0.48865 | 0.420076 | 0.442168 | 0.550404 | 0.676238 | 0.577214 | 0.343289 | 0.411235 | 0.116289 | 0.504976 | 0.201647 | 0.510336 | 0.28266 | 0.25465 | 0.584449 | 0.285181 | 0.564576 | 0.462415 | 0.292051 | 0.289767 | 0.542503 | 0.446202 | 0.36498 | 0.369567 | 0.265448 | 0.377007 |
| TCGA-D5-6923-01A-11R-A32Z-07 | -0.03569 | 0.419892 | 0.386861 | 0.390609 | 0.449211 | 0.529387 | 0.637352 | 0.519156 | 0.355118 | 0.309359 | 0.124389 | 0.513257 | 0.11196 | 0.491011 | 0.206093 | 0.229294 | 0.446848 | 0.304756 | 0.528179 | 0.461014 | 0.28167 | 0.105839 | 0.547915 | 0.426367 | 0.323645 | 0.338673 | 0.273565 | 0.282777 |
| TCGA-AZ-6599-01A-11R-1774-07 | 0.027409 | 0.362051 | 0.428446 | 0.247192 | 0.46494 | 0.538273 | 0.629459 | 0.406177 | 0.296883 | 0.265137 | 0.121169 | 0.413388 | 0.088139 | 0.386928 | 0.10185 | 0.080553 | 0.342438 | 0.272079 | 0.514406 | 0.366142 | 0.145022 | -0.09633 | 0.49028 | 0.113342 | 0.192283 | 0.261657 | 0.284035 | 0.341715 |
| TCGA-D5-6922-01A-11R-1928-07 | 0.011488 | 0.36301 | 0.428778 | 0.379969 | 0.448166 | 0.535538 | 0.646677 | 0.519104 | 0.358533 | 0.336522 | 0.061784 | 0.502606 | 0.141512 | 0.494508 | 0.2201 | 0.254039 | 0.466126 | 0.30025 | 0.540134 | 0.47476 | 0.259914 | 0.023558 | 0.526107 | 0.417395 | 0.359412 | 0.325577 | 0.203586 | 0.27014 |
| TCGA-AA-3692-01A-01R-0905-07 | 0.062577 | 0.4557 | 0.469692 | 0.404704 | 0.475403 | 0.558185 | 0.652445 | 0.528755 | 0.297032 | 0.337314 | 0.21069 | 0.503227 | 0.145111 | 0.52168 | 0.273872 | 0.267491 | 0.526337 | 0.263853 | 0.537061 | 0.433353 | 0.255082 | 0.108128 | 0.539113 | 0.417948 | 0.335567 | 0.35446 | 0.32327 | 0.311867 |
| TCGA-4T-AA8H-01A-11R-A41B-07 | -0.04813 | 0.373354 | 0.35688 | 0.248063 | 0.428936 | 0.49379 | 0.622389 | 0.409966 | 0.216321 | 0.239491 | 0.138262 | 0.435414 | 0.061205 | 0.436287 | 0.089545 | 0.05163 | 0.266411 | 0.243606 | 0.541168 | 0.354117 | 0.15637 | -0.00959 | 0.488814 | 0.17885 | 0.212693 | 0.261597 | 0.204353 | 0.288505 |
| TCGA-AA-A01Q-01A-01R-A002-07 | -0.03281 | 0.464153 | 0.513219 | 0.398656 | 0.444651 | 0.535737 | 0.64503 | 0.473995 | 0.280013 | 0.381171 | 0.120294 | 0.446214 | 0.105437 | 0.450171 | 0.201332 | 0.10959 | 0.470922 | 0.279565 | 0.532669 | 0.425402 | 0.238413 | 0.077945 | 0.507495 | 0.277184 | 0.299873 | 0.295409 | 0.292284 | 0.245975 |
| TCGA-AA-3979-01A-01R-1022-07 | -0.12728 | 0.427486 | 0.374953 | 0.32576 | 0.450935 | 0.531974 | 0.580724 | 0.488238 | 0.302756 | 0.207758 | 0.072371 | 0.472851 | 0.002348 | 0.500939 | 0.17385 | 0.1369 | 0.347622 | 0.234406 | 0.527557 | 0.352671 | 0.223955 | 0.148467 | 0.490232 | 0.300216 | 0.305784 | 0.305751 | 0.300403 | 0.278324 |
| TCGA-A6-A565-01A-31R-A28H-07 | 0.389928 | 0.511305 | 0.553374 | 0.410525 | 0.431006 | 0.585158 | 0.715566 | 0.514132 | 0.350476 | 0.446706 | 0.243637 | 0.525484 | 0.390083 | 0.53418 | 0.293623 | 0.300279 | 0.602806 | 0.322741 | 0.56124 | 0.4709 | 0.300516 | 0.202617 | 0.543718 | 0.438486 | 0.352495 | 0.385316 | 0.287664 | 0.349268 |
| TCGA-A6-5667-01A-21R-1723-07 | -0.02959 | 0.401941 | 0.39786 | 0.363865 | 0.455024 | 0.551757 | 0.610989 | 0.504163 | 0.342173 | 0.248358 | 0.064758 | 0.481936 | 0.095778 | 0.456515 | 0.178732 | 0.222978 | 0.394265 | 0.328665 | 0.536113 | 0.465784 | 0.259283 | 0.054621 | 0.52663 | 0.358344 | 0.294248 | 0.333448 | 0.195357 | 0.267202 |
| TCGA-AA-3842-01A-01R-1022-07 | -0.08183 | 0.430302 | 0.462766 | 0.387018 | 0.437616 | 0.571782 | 0.6584 | 0.482518 | 0.319156 | 0.293695 | 0.025266 | 0.519379 | 0.084898 | 0.501812 | 0.24935 | 0.15405 | 0.46171 | 0.244116 | 0.552213 | 0.457565 | 0.253709 | 0.045309 | 0.50572 | 0.409396 | 0.339026 | 0.33096 | 0.284521 | 0.304734 |
| TCGA-A6-A5ZU-01A-11R-A28H-07 | 0.143167 | 0.347474 | 0.452631 | 0.336251 | 0.439176 | 0.562662 | 0.674329 | 0.483421 | 0.334694 | 0.350447 | 0.133254 | 0.486428 | 0.245315 | 0.474476 | 0.288503 | 0.168935 | 0.524987 | 0.286392 | 0.558421 | 0.474609 | 0.277624 | -0.01117 | 0.531391 | 0.40595 | 0.32889 | 0.368966 | 0.208586 | 0.221412 |
| TCGA-AA-3870-01A-01R-1022-07 | 0.14839 | 0.442639 | 0.49405 | 0.404625 | 0.448616 | 0.630686 | 0.694517 | 0.572426 | 0.325536 | 0.400208 | 0.091026 | 0.549384 | 0.272347 | 0.510015 | 0.304123 | 0.281241 | 0.576046 | 0.28616 | 0.580764 | 0.499057 | 0.332542 | 0.06389 | 0.52554 | 0.526111 | 0.402483 | 0.385286 | 0.247615 | 0.344139 |
| TCGA-NH-A6GC-01A-12R-A41B-07 | -0.0161 | 0.268198 | 0.390346 | 0.291753 | 0.441689 | 0.607613 | 0.650199 | 0.459686 | 0.247929 | 0.271957 | 0.105937 | 0.376617 | 0.054421 | 0.431471 | 0.161692 | 0.019926 | 0.397257 | 0.231759 | 0.510023 | 0.435899 | 0.235046 | -0.02799 | 0.517254 | 0.22743 | 0.261269 | 0.236706 | 0.272901 | 0.242214 |
| TCGA-AA-3511-01A-21R-1839-07 | 0.022992 | 0.351706 | 0.403079 | 0.349581 | 0.462122 | 0.548251 | 0.628781 | 0.509609 | 0.344756 | 0.285725 | 0.135838 | 0.485755 | 0.134021 | 0.469821 | 0.21838 | 0.207679 | 0.448046 | 0.273128 | 0.568046 | 0.455058 | 0.243748 | 0.119436 | 0.542411 | 0.361123 | 0.299704 | 0.322541 | 0.290377 | 0.287539 |
| TCGA-CM-6167-01A-11R-1653-07 | 0.193547 | 0.389173 | 0.467595 | 0.357127 | 0.452685 | 0.501449 | 0.701685 | 0.554368 | 0.418192 | 0.415978 | 0.177116 | 0.516763 | 0.277441 | 0.521801 | 0.314392 | 0.358273 | 0.537534 | 0.293116 | 0.541523 | 0.519997 | 0.329163 | 0.032055 | 0.565535 | 0.414146 | 0.376079 | 0.385284 | 0.188079 | 0.383953 |
| TCGA-CM-5348-01A-21R-1723-07 | -0.05658 | 0.370261 | 0.388631 | 0.423242 | 0.447615 | 0.592507 | 0.698301 | 0.579373 | 0.331733 | 0.385204 | 0.12854 | 0.539092 | 0.203857 | 0.570149 | 0.32597 | 0.313834 | 0.57538 | 0.288566 | 0.563668 | 0.502664 | 0.300645 | 0.163564 | 0.520109 | 0.529837 | 0.401162 | 0.375043 | 0.268197 | 0.327867 |
| TCGA-AA-3949-01A-01R-1022-07 | 0.235215 | 0.557003 | 0.629445 | 0.462591 | 0.465668 | 0.568933 | 0.690205 | 0.555278 | 0.372018 | 0.464633 | 0.144373 | 0.544038 | 0.309239 | 0.531201 | 0.402069 | 0.317688 | 0.65193 | 0.281354 | 0.578412 | 0.516698 | 0.374756 | 0.19188 | 0.546971 | 0.492198 | 0.401267 | 0.419718 | 0.309308 | 0.400794 |
| TCGA-AY-A54L-01A-11R-A28H-07 | -0.12685 | 0.391102 | 0.390411 | 0.288468 | 0.435999 | 0.482987 | 0.582607 | 0.412595 | 0.256818 | 0.196196 | 0.133511 | 0.419202 | -0.01009 | 0.438924 | 0.052805 | 0.154601 | 0.280969 | 0.238547 | 0.587403 | 0.3385 | 0.163915 | 0.044639 | 0.4924 | 0.184355 | 0.210853 | 0.250186 | 0.264612 | 0.304224 |
| TCGA-A6-2671-01A-01R-1410-07 | -0.03226 | 0.377027 | 0.42684 | 0.37746 | 0.465943 | 0.570197 | 0.649146 | 0.542218 | 0.337778 | 0.335963 | 0.157937 | 0.503892 | 0.137774 | 0.476452 | 0.266154 | 0.256375 | 0.51887 | 0.269003 | 0.534665 | 0.462085 | 0.294785 | 0.077905 | 0.499023 | 0.435899 | 0.340364 | 0.332545 | 0.234687 | 0.253933 |
| TCGA-NH-A8F8-01A-72R-A41B-07 | 0.028515 | 0.369847 | 0.401243 | 0.358277 | 0.472821 | 0.546287 | 0.658889 | 0.502877 | 0.32794 | 0.309069 | 0.1093 | 0.47247 | 0.142065 | 0.440924 | 0.196937 | 0.20038 | 0.444889 | 0.242254 | 0.514968 | 0.465193 | 0.258181 | 0.011093 | 0.519872 | 0.349499 | 0.28295 | 0.318165 | 0.225163 | 0.224518 |
| TCGA-AA-3516-01A-02R-0826-07 | 0.045973 | 0.489878 | 0.512027 | 0.420272 | 0.457267 | 0.541006 | 0.651811 | 0.524949 | 0.328601 | 0.434567 | 0.088267 | 0.546647 | 0.200219 | 0.478283 | 0.264464 | 0.210354 | 0.535201 | 0.255462 | 0.559962 | 0.448052 | 0.301223 | 0.196203 | 0.506848 | 0.39943 | 0.369216 | 0.383724 | 0.316052 | 0.397048 |
| TCGA-G4-6628-01A-11R-1839-07 | 0.168964 | 0.557691 | 0.641881 | 0.424963 | 0.458095 | 0.496344 | 0.680744 | 0.56395 | 0.389767 | 0.507174 | 0.197195 | 0.552732 | 0.299862 | 0.508642 | 0.315962 | 0.271217 | 0.627899 | 0.298758 | 0.556365 | 0.478116 | 0.366096 | 0.087328 | 0.499085 | 0.473102 | 0.396533 | 0.412412 | 0.21317 | 0.39695 |
| TCGA-AD-6964-01A-11R-1928-07 | 0.187504 | 0.486866 | 0.618739 | 0.447297 | 0.49464 | 0.560513 | 0.708971 | 0.548086 | 0.346276 | 0.491181 | 0.112626 | 0.514753 | 0.259806 | 0.515256 | 0.35189 | 0.274909 | 0.642032 | 0.245227 | 0.546245 | 0.516509 | 0.296904 | 0.038256 | 0.562725 | 0.492557 | 0.394534 | 0.413228 | 0.310223 | 0.34554 |
| TCGA-AD-6888-01A-11R-1928-07 | -0.10376 | 0.319927 | 0.376078 | 0.308183 | 0.459928 | 0.532606 | 0.58779 | 0.407214 | 0.271813 | 0.22849 | 0.036864 | 0.42569 | -0.00142 | 0.431699 | 0.11438 | 0.069536 | 0.300441 | 0.235034 | 0.572875 | 0.324495 | 0.159394 | 0.096441 | 0.503603 | 0.237078 | 0.239047 | 0.253649 | 0.261272 | 0.261372 |
| TCGA-AA-3973-01A-01R-1022-07 | 0.008199 | 0.296145 | 0.400993 | 0.34966 | 0.466129 | 0.535171 | 0.623832 | 0.455467 | 0.305229 | 0.276064 | 0.048297 | 0.438458 | 0.082088 | 0.467764 | 0.181031 | 0.164015 | 0.391055 | 0.297111 | 0.516421 | 0.431333 | 0.239021 | -0.05098 | 0.534108 | 0.334397 | 0.263231 | 0.295103 | 0.200281 | 0.207789 |
| TCGA-AA-3680-01A-01R-0905-07 | 0.192027 | 0.3784 | 0.470198 | 0.342193 | 0.464362 | 0.575151 | 0.656289 | 0.501975 | 0.291491 | 0.3414 | 0.180259 | 0.4865 | 0.203411 | 0.478058 | 0.19907 | 0.209299 | 0.450079 | 0.274768 | 0.556929 | 0.419963 | 0.206477 | 0.00918 | 0.51572 | 0.333034 | 0.319492 | 0.344166 | 0.296841 | 0.291533 |
| TCGA-AA-3514-01A-02R-0821-07 | 0.008535 | 0.312169 | 0.426428 | 0.378515 | 0.457392 | 0.622752 | 0.630889 | 0.494583 | 0.288163 | 0.331554 | 0.081466 | 0.457193 | 0.079144 | 0.472488 | 0.254034 | 0.223077 | 0.49546 | 0.250846 | 0.624365 | 0.454756 | 0.235965 | 0.003295 | 0.465594 | 0.39631 | 0.359856 | 0.343797 | 0.270629 | 0.248785 |
| TCGA-QG-A5YW-01A-11R-A28H-07 | 0.105316 | 0.444931 | 0.46725 | 0.373801 | 0.448333 | 0.581542 | 0.641601 | 0.478257 | 0.326661 | 0.378574 | 0.147814 | 0.489077 | 0.191241 | 0.475538 | 0.220918 | 0.215359 | 0.505708 | 0.266764 | 0.519919 | 0.440085 | 0.257721 | 0.051329 | 0.50895 | 0.363081 | 0.302988 | 0.31791 | 0.306529 | 0.287187 |
| TCGA-AY-A69D-01A-11R-A37K-07 | 0.101025 | 0.471036 | 0.396375 | 0.349697 | 0.427519 | 0.536403 | 0.634274 | 0.448152 | 0.293118 | 0.273622 | 0.173759 | 0.44194 | 0.189785 | 0.449872 | 0.19228 | 0.143143 | 0.409618 | 0.282464 | 0.52309 | 0.416094 | 0.200469 | 0.030323 | 0.473176 | 0.307284 | 0.262328 | 0.313029 | 0.276019 | 0.291319 |
| TCGA-5M-AAT5-01A-21R-A41B-07 | -0.06172 | 0.410564 | 0.38151 | 0.293804 | 0.446638 | 0.48773 | 0.593989 | 0.419454 | 0.25864 | 0.232369 | 0.143414 | 0.398244 | 0.029729 | 0.444873 | 0.116518 | 0.125254 | 0.327681 | 0.21876 | 0.503123 | 0.375138 | 0.182495 | -0.00047 | 0.477367 | 0.229879 | 0.221977 | 0.292915 | 0.244275 | 0.280316 |
| TCGA-AA-3673-01A-01R-0905-07 | 0.107009 | 0.402497 | 0.440617 | 0.336599 | 0.470229 | 0.537439 | 0.66092 | 0.470679 | 0.312181 | 0.294321 | 0.122321 | 0.477488 | 0.176715 | 0.468914 | 0.225238 | 0.210573 | 0.417045 | 0.314562 | 0.545986 | 0.431884 | 0.233961 | 0.034402 | 0.511446 | 0.367155 | 0.312179 | 0.323463 | 0.311081 | 0.284526 |
| TCGA-AA-3855-01A-01R-1022-07 | 0.112893 | 0.438664 | 0.486739 | 0.337668 | 0.464507 | 0.566559 | 0.653138 | 0.484326 | 0.342333 | 0.327732 | 0.143489 | 0.494592 | 0.181337 | 0.48007 | 0.258521 | 0.236411 | 0.484251 | 0.273789 | 0.573618 | 0.43079 | 0.222507 | 0.192789 | 0.487686 | 0.357276 | 0.341492 | 0.333347 | 0.286488 | 0.317804 |
| TCGA-CM-6172-01A-11R-1653-07 | 0.013825 | 0.39242 | 0.388002 | 0.323065 | 0.443716 | 0.514484 | 0.646895 | 0.478076 | 0.342431 | 0.297259 | 0.1166 | 0.458885 | 0.10562 | 0.509843 | 0.211618 | 0.191795 | 0.428288 | 0.288499 | 0.536597 | 0.438869 | 0.227402 | 0.044387 | 0.540786 | 0.332166 | 0.274319 | 0.311786 | 0.289755 | 0.307332 |
| TCGA-AZ-6600-01A-11R-1774-07 | 0.209233 | 0.467723 | 0.444786 | 0.413302 | 0.438303 | 0.565668 | 0.683695 | 0.556523 | 0.337295 | 0.35333 | 0.183163 | 0.518764 | 0.243826 | 0.49047 | 0.277065 | 0.270757 | 0.513495 | 0.280692 | 0.555887 | 0.465188 | 0.294325 | 0.097449 | 0.556937 | 0.406622 | 0.360553 | 0.367088 | 0.247097 | 0.342698 |
| TCGA-F4-6463-01A-11R-1723-07 | 0.047727 | 0.385239 | 0.471508 | 0.307043 | 0.486178 | 0.537899 | 0.657627 | 0.494204 | 0.358288 | 0.299418 | 0.143166 | 0.482995 | 0.135861 | 0.479954 | 0.242847 | 0.205067 | 0.455894 | 0.290548 | 0.514611 | 0.481811 | 0.250353 | -0.0633 | 0.545099 | 0.373808 | 0.318972 | 0.343918 | 0.237382 | 0.32668 |
| TCGA-AA-3971-01A-01R-1022-07 | 0.182018 | 0.423355 | 0.508721 | 0.384755 | 0.464641 | 0.55717 | 0.664026 | 0.505096 | 0.312046 | 0.334152 | 0.154946 | 0.515276 | 0.245975 | 0.492286 | 0.243239 | 0.251242 | 0.516078 | 0.296887 | 0.582421 | 0.440536 | 0.262355 | 0.095105 | 0.53574 | 0.394128 | 0.340394 | 0.358065 | 0.282467 | 0.270477 |
| TCGA-AA-3666-01A-02R-0905-07 | 0.042313 | 0.487565 | 0.443894 | 0.38532 | 0.451117 | 0.559394 | 0.641509 | 0.491999 | 0.30045 | 0.335927 | 0.158705 | 0.500488 | 0.120199 | 0.530828 | 0.245664 | 0.191461 | 0.489152 | 0.241889 | 0.575954 | 0.425454 | 0.240667 | 0.191718 | 0.500785 | 0.405805 | 0.327255 | 0.347183 | 0.314067 | 0.375535 |
| TCGA-D5-6898-01A-11R-1928-07 | 0.071761 | 0.411953 | 0.426213 | 0.414748 | 0.474179 | 0.554697 | 0.647603 | 0.53336 | 0.342395 | 0.302524 | 0.089618 | 0.524659 | 0.184461 | 0.502885 | 0.246471 | 0.258497 | 0.508739 | 0.310804 | 0.55458 | 0.481659 | 0.286934 | 0.085315 | 0.533585 | 0.472394 | 0.360743 | 0.371267 | 0.238848 | 0.306025 |
| TCGA-D5-6537-01A-11R-1723-07 | -0.10433 | 0.474411 | 0.442704 | 0.373152 | 0.437503 | 0.548433 | 0.612638 | 0.503058 | 0.331138 | 0.28509 | 0.095269 | 0.482511 | 0.051334 | 0.472535 | 0.168557 | 0.211649 | 0.389295 | 0.285288 | 0.515369 | 0.406847 | 0.255569 | 0.176847 | 0.512872 | 0.375427 | 0.252098 | 0.318101 | 0.272261 | 0.299757 |
| TCGA-SS-A7HO-01A-21R-A37K-07 | -0.19013 | 0.366455 | 0.371136 | 0.269815 | 0.4389 | 0.499118 | 0.586075 | 0.427721 | 0.266295 | 0.199712 | 0.121136 | 0.406218 | -0.02446 | 0.464727 | 0.122532 | 0.105344 | 0.363405 | 0.254174 | 0.52939 | 0.364709 | 0.168151 | -0.00214 | 0.542823 | 0.27193 | 0.223842 | 0.238501 | 0.211675 | 0.265094 |
| TCGA-CK-5915-01A-11R-1653-07 | -0.1732 | 0.416753 | 0.320186 | 0.302353 | 0.451359 | 0.525414 | 0.596402 | 0.428139 | 0.305517 | 0.220551 | 0.07007 | 0.436979 | 0.018982 | 0.434127 | 0.143452 | 0.104151 | 0.274456 | 0.344782 | 0.52767 | 0.405603 | 0.158032 | -0.0452 | 0.477902 | 0.287352 | 0.230439 | 0.285135 | 0.205619 | 0.259474 |
| TCGA-AA-A00L-01A-01R-A002-07 | -0.09749 | 0.375708 | 0.396757 | 0.334414 | 0.462978 | 0.570029 | 0.584588 | 0.454146 | 0.263444 | 0.222867 | 0.103719 | 0.459242 | 0.046332 | 0.447108 | 0.117669 | 0.121434 | 0.332166 | 0.272787 | 0.525391 | 0.405351 | 0.199575 | -0.01167 | 0.504815 | 0.300232 | 0.241726 | 0.283204 | 0.263706 | 0.234462 |
| TCGA-G4-6321-01A-11R-1723-07 | 0.248727 | 0.396686 | 0.524182 | 0.370818 | 0.448405 | 0.559941 | 0.629294 | 0.508456 | 0.28584 | 0.397612 | 0.187877 | 0.463172 | 0.294453 | 0.432278 | 0.214758 | 0.164143 | 0.44018 | 0.261414 | 0.583201 | 0.411312 | 0.238326 | 0.15795 | 0.522171 | 0.310168 | 0.332825 | 0.340583 | 0.320768 | 0.336265 |
| TCGA-AA-3489-01A-21R-1839-07 | 0.417235 | 0.49656 | 0.569337 | 0.43822 | 0.493686 | 0.541764 | 0.693973 | 0.621501 | 0.423248 | 0.437213 | 0.197492 | 0.565868 | 0.456561 | 0.507305 | 0.34969 | 0.391459 | 0.657541 | 0.327517 | 0.562664 | 0.493643 | 0.353349 | 0.139521 | 0.566274 | 0.552287 | 0.399513 | 0.431665 | 0.184567 | 0.30319 |
| TCGA-AA-A01S-01A-21R-A083-07 | -0.10909 | 0.299833 | 0.327979 | 0.245317 | 0.423168 | 0.570893 | 0.5843 | 0.4034 | 0.213637 | 0.17611 | -0.03116 | 0.421152 | -0.04838 | 0.400015 | 0.081936 | 0.030847 | 0.290717 | 0.222268 | 0.512639 | 0.353784 | 0.137718 | 0.004432 | 0.461896 | 0.203527 | 0.25193 | 0.258518 | 0.221965 | 0.185399 |
| TCGA-DM-A1HB-01A-21R-A180-07 | -0.10058 | 0.394189 | 0.359348 | 0.37282 | 0.435954 | 0.539973 | 0.643339 | 0.503164 | 0.349894 | 0.266745 | 0.057959 | 0.460236 | 0.028005 | 0.440394 | 0.178295 | 0.219186 | 0.445208 | 0.261521 | 0.535726 | 0.410726 | 0.250362 | 0.164044 | 0.50991 | 0.294111 | 0.275158 | 0.284341 | 0.257312 | 0.374043 |
| TCGA-D5-6931-01A-11R-1928-07 | 0.173826 | 0.481197 | 0.485925 | 0.402176 | 0.437821 | 0.570659 | 0.696474 | 0.52508 | 0.344418 | 0.404995 | 0.117851 | 0.53984 | 0.247745 | 0.508397 | 0.238119 | 0.256613 | 0.516881 | 0.321408 | 0.538865 | 0.473408 | 0.301697 | 0.104027 | 0.517459 | 0.439858 | 0.341005 | 0.367983 | 0.299199 | 0.350812 |
| TCGA-T9-A92H-01A-11R-A37K-07 | -0.06384 | 0.414761 | 0.407431 | 0.315865 | 0.44915 | 0.528827 | 0.605043 | 0.432246 | 0.271375 | 0.289009 | 0.095809 | 0.43206 | 0.064332 | 0.461836 | 0.132074 | 0.160228 | 0.303237 | 0.272254 | 0.520997 | 0.385019 | 0.233957 | 0.001626 | 0.473399 | 0.261002 | 0.22672 | 0.292771 | 0.230774 | 0.252512 |
| TCGA-AD-6965-01A-11R-1928-07 | 0.04168 | 0.402503 | 0.437872 | 0.301677 | 0.446318 | 0.552124 | 0.638397 | 0.453859 | 0.313271 | 0.275336 | 0.079932 | 0.462059 | 0.122428 | 0.450145 | 0.114251 | 0.125581 | 0.378466 | 0.284353 | 0.524061 | 0.417039 | 0.21794 | -0.03481 | 0.47432 | 0.308418 | 0.273485 | 0.304614 | 0.210777 | 0.271559 |
| TCGA-DM-A1D7-01A-11R-A155-07 | -0.03493 | 0.400981 | 0.437064 | 0.342822 | 0.455124 | 0.562627 | 0.616597 | 0.43019 | 0.292887 | 0.257791 | 0.127626 | 0.434074 | 0.052247 | 0.46433 | 0.175832 | 0.099942 | 0.367004 | 0.268916 | 0.565107 | 0.376158 | 0.214542 | -0.09097 | 0.484478 | 0.257202 | 0.269533 | 0.291186 | 0.292849 | 0.315694 |
| TCGA-A6-6140-01A-11R-1774-07 | 0.001235 | 0.438807 | 0.477214 | 0.331472 | 0.440118 | 0.547211 | 0.602247 | 0.463618 | 0.30606 | 0.269047 | 0.136451 | 0.465206 | 0.064903 | 0.454631 | 0.161561 | 0.204095 | 0.394605 | 0.299684 | 0.549949 | 0.420232 | 0.240107 | 0.124049 | 0.519116 | 0.31183 | 0.275149 | 0.31148 | 0.297455 | 0.258842 |
| TCGA-AA-3672-01A-01R-0905-07 | 0.094774 | 0.417229 | 0.56771 | 0.435913 | 0.469418 | 0.631704 | 0.672753 | 0.57534 | 0.263769 | 0.490096 | 0.112012 | 0.521455 | 0.180052 | 0.50672 | 0.301904 | 0.180616 | 0.608947 | 0.239262 | 0.601703 | 0.498673 | 0.346435 | 0.223246 | 0.525425 | 0.441105 | 0.39896 | 0.361771 | 0.313075 | 0.323464 |
| TCGA-AA-3553-01A-01R-0821-07 | 0.111065 | 0.450073 | 0.511645 | 0.424066 | 0.470127 | 0.55078 | 0.660749 | 0.513723 | 0.332093 | 0.368597 | 0.070214 | 0.522665 | 0.203887 | 0.504597 | 0.238018 | 0.19911 | 0.517772 | 0.275591 | 0.566309 | 0.456101 | 0.267204 | 0.16463 | 0.541518 | 0.431607 | 0.355689 | 0.380541 | 0.313989 | 0.304102 |
| TCGA-A6-A56B-01A-31R-A28H-07 | -0.15394 | 0.316449 | 0.309582 | 0.327227 | 0.426636 | 0.528302 | 0.615216 | 0.472206 | 0.319007 | 0.275727 | 0.151156 | 0.442957 | -0.00285 | 0.40823 | 0.147476 | 0.192494 | 0.365347 | 0.260631 | 0.504378 | 0.426474 | 0.231827 | -0.12402 | 0.484639 | 0.351807 | 0.268695 | 0.280761 | 0.212082 | 0.237717 |
| TCGA-D5-6927-01A-21R-1928-07 | 0.06158 | 0.507079 | 0.514002 | 0.428261 | 0.434345 | 0.566551 | 0.689916 | 0.572849 | 0.365619 | 0.449566 | 0.174613 | 0.517184 | 0.21118 | 0.511211 | 0.269583 | 0.258753 | 0.60758 | 0.260992 | 0.550926 | 0.489214 | 0.342083 | 0.193354 | 0.518787 | 0.488805 | 0.393011 | 0.391038 | 0.303419 | 0.33295 |
| TCGA-AA-A00E-01A-01R-A002-07 | 0.186278 | 0.463356 | 0.512754 | 0.405162 | 0.449282 | 0.586373 | 0.674144 | 0.538646 | 0.328402 | 0.40219 | 0.162757 | 0.534816 | 0.299303 | 0.455844 | 0.269398 | 0.179179 | 0.591016 | 0.177045 | 0.522005 | 0.446179 | 0.29474 | 0.042163 | 0.50613 | 0.433415 | 0.377068 | 0.365714 | 0.305686 | 0.366737 |
| TCGA-AA-3814-01A-01R-0905-07 | 0.120684 | 0.440525 | 0.49983 | 0.425214 | 0.478002 | 0.571308 | 0.656839 | 0.564281 | 0.314359 | 0.386561 | 0.196593 | 0.541175 | 0.222467 | 0.502649 | 0.288864 | 0.304515 | 0.549928 | 0.314657 | 0.556482 | 0.477445 | 0.286811 | 0.164636 | 0.538765 | 0.48488 | 0.367471 | 0.385089 | 0.309577 | 0.288145 |
| TCGA-A6-2676-01A-01R-0826-07 | 0.023237 | 0.548973 | 0.570577 | 0.442148 | 0.429347 | 0.544029 | 0.657389 | 0.552953 | 0.299835 | 0.453534 | 0.111559 | 0.550889 | 0.225937 | 0.514206 | 0.312445 | 0.218925 | 0.612619 | 0.217668 | 0.538858 | 0.495168 | 0.350985 | 0.172942 | 0.509998 | 0.497051 | 0.437079 | 0.380661 | 0.238622 | 0.351665 |
| TCGA-AA-A02K-01A-03R-A32Y-07 | -0.13386 | 0.34709 | 0.348495 | 0.302923 | 0.417365 | 0.580235 | 0.626757 | 0.480349 | 0.240551 | 0.227358 | -0.00755 | 0.433905 | 0.019883 | 0.42439 | 0.138957 | 0.104476 | 0.375136 | 0.223732 | 0.513733 | 0.379919 | 0.203304 | 0.09539 | 0.506562 | 0.244548 | 0.270917 | 0.282899 | 0.179648 | 0.212769 |
| TCGA-F4-6570-01A-11R-1774-07 | 0.155469 | 0.47319 | 0.572787 | 0.424369 | 0.444294 | 0.608949 | 0.701886 | 0.602848 | 0.331216 | 0.476139 | 0.063143 | 0.517503 | 0.261401 | 0.491388 | 0.30606 | 0.243692 | 0.638416 | 0.296469 | 0.55176 | 0.508653 | 0.349119 | 0.075026 | 0.52203 | 0.544115 | 0.424736 | 0.407636 | 0.292372 | 0.379155 |
| TCGA-DM-A1D9-01A-11R-A155-07 | -0.1333 | 0.387875 | 0.371083 | 0.314873 | 0.470315 | 0.528768 | 0.626228 | 0.435196 | 0.262937 | 0.219346 | 0.108977 | 0.44863 | 0.024142 | 0.467971 | 0.133073 | 0.09628 | 0.340825 | 0.259445 | 0.519226 | 0.37301 | 0.216785 | -0.0645 | 0.485016 | 0.267205 | 0.242421 | 0.252454 | 0.280031 | 0.299684 |
| TCGA-CK-4951-01A-01R-1410-07 | 0.002893 | 0.504614 | 0.538256 | 0.437794 | 0.480386 | 0.530576 | 0.683062 | 0.563144 | 0.328074 | 0.445651 | 0.131195 | 0.526394 | 0.196122 | 0.503169 | 0.272103 | 0.203735 | 0.578381 | 0.298377 | 0.565842 | 0.471624 | 0.29082 | 0.149827 | 0.524056 | 0.443487 | 0.382699 | 0.390678 | 0.308369 | 0.363502 |
| TCGA-AA-3956-01A-02R-1022-07 | 0.109539 | 0.400873 | 0.459255 | 0.376281 | 0.481833 | 0.564304 | 0.642834 | 0.511394 | 0.319662 | 0.310984 | 0.114327 | 0.466179 | 0.168414 | 0.508752 | 0.214163 | 0.229387 | 0.450884 | 0.315138 | 0.553148 | 0.443931 | 0.241323 | 0.121567 | 0.551558 | 0.357419 | 0.29729 | 0.341143 | 0.291127 | 0.282309 |
| TCGA-A6-2683-01A-01R-0821-07 | -0.1347 | 0.261411 | 0.336553 | 0.310686 | 0.483655 | 0.620333 | 0.625195 | 0.485366 | 0.217235 | 0.221347 | -0.00154 | 0.443207 | 0.034006 | 0.449538 | 0.153336 | 0.120291 | 0.419052 | 0.240131 | 0.570995 | 0.396029 | 0.174282 | -0.05447 | 0.470139 | 0.281133 | 0.265129 | 0.29734 | 0.253541 | 0.210568 |
| TCGA-AA-3713-01A-21R-1723-07 | 0.178538 | 0.50283 | 0.506614 | 0.393993 | 0.446136 | 0.542504 | 0.652039 | 0.532619 | 0.335077 | 0.394359 | 0.194978 | 0.520206 | 0.272589 | 0.489216 | 0.237502 | 0.260851 | 0.54132 | 0.284932 | 0.548585 | 0.444401 | 0.305941 | 0.153211 | 0.519625 | 0.401246 | 0.32812 | 0.344816 | 0.262058 | 0.387789 |
| TCGA-AA-3955-01A-02R-1022-07 | -0.03149 | 0.425391 | 0.426477 | 0.334076 | 0.468568 | 0.510611 | 0.594264 | 0.449713 | 0.348328 | 0.222407 | 0.119892 | 0.475018 | 0.100317 | 0.487791 | 0.171464 | 0.236216 | 0.384513 | 0.25112 | 0.550736 | 0.40428 | 0.222552 | 0.097673 | 0.496285 | 0.341455 | 0.291278 | 0.299972 | 0.267957 | 0.283819 |
| TCGA-AZ-6598-01A-11R-1774-07 | -0.01579 | 0.463282 | 0.533998 | 0.362644 | 0.46467 | 0.608721 | 0.658105 | 0.52475 | 0.301269 | 0.374505 | 0.104652 | 0.483393 | 0.123939 | 0.477087 | 0.28034 | 0.138482 | 0.549814 | 0.23908 | 0.531598 | 0.445899 | 0.267169 | 0.147905 | 0.523599 | 0.354442 | 0.301425 | 0.321993 | 0.238082 | 0.373471 |
| TCGA-F4-6704-01A-11R-1839-07 | 0.073311 | 0.401117 | 0.455213 | 0.371228 | 0.458933 | 0.5967 | 0.685985 | 0.541295 | 0.380208 | 0.376039 | 0.096058 | 0.482536 | 0.167021 | 0.504894 | 0.286291 | 0.224872 | 0.565639 | 0.241334 | 0.546282 | 0.496165 | 0.301033 | 0.044308 | 0.531344 | 0.437675 | 0.345792 | 0.346841 | 0.215507 | 0.325113 |
| TCGA-CM-6163-01A-11R-1653-07 | 0.157182 | 0.475185 | 0.487744 | 0.389619 | 0.460331 | 0.503 | 0.660573 | 0.50922 | 0.351513 | 0.34018 | 0.139438 | 0.485389 | 0.239795 | 0.522047 | 0.262227 | 0.211428 | 0.519981 | 0.307097 | 0.560544 | 0.471185 | 0.247277 | 0.135929 | 0.522024 | 0.394771 | 0.333835 | 0.353829 | 0.245112 | 0.278635 |
| TCGA-AA-3869-01A-01R-1022-07 | 0.007391 | 0.459673 | 0.483968 | 0.42271 | 0.475596 | 0.552114 | 0.660761 | 0.537854 | 0.327867 | 0.341891 | 0.142436 | 0.530339 | 0.16467 | 0.531234 | 0.272915 | 0.255092 | 0.539632 | 0.29811 | 0.593575 | 0.438572 | 0.244763 | 0.248918 | 0.523268 | 0.430751 | 0.371102 | 0.367813 | 0.294857 | 0.330522 |
| TCGA-AA-3538-01A-01R-0821-07 | 0.075733 | 0.380094 | 0.388365 | 0.346267 | 0.452179 | 0.567338 | 0.634707 | 0.509945 | 0.290723 | 0.284208 | 0.096 | 0.508507 | 0.151444 | 0.497476 | 0.228035 | 0.200388 | 0.428652 | 0.325459 | 0.587322 | 0.436732 | 0.241487 | 0.17021 | 0.541077 | 0.393611 | 0.335076 | 0.331216 | 0.246465 | 0.256969 |
| TCGA-DM-A1DA-01A-11R-A155-07 | -0.08119 | 0.430326 | 0.405251 | 0.324144 | 0.419677 | 0.55945 | 0.61157 | 0.468577 | 0.260885 | 0.240544 | 0.054222 | 0.467394 | 0.035454 | 0.46603 | 0.137763 | 0.127583 | 0.337493 | 0.278379 | 0.518341 | 0.382341 | 0.251578 | -0.01628 | 0.463047 | 0.260231 | 0.242828 | 0.261887 | 0.215419 | 0.348138 |
| TCGA-AA-3494-01A-01R-1410-07 | -0.00365 | 0.381888 | 0.442312 | 0.349044 | 0.481547 | 0.563091 | 0.620096 | 0.490299 | 0.315874 | 0.270491 | 0.074634 | 0.479818 | 0.0704 | 0.526435 | 0.161297 | 0.231328 | 0.40672 | 0.250313 | 0.541999 | 0.404131 | 0.24882 | 0.09967 | 0.545446 | 0.370816 | 0.299842 | 0.319618 | 0.338385 | 0.323377 |
| TCGA-A6-2677-01A-01R-0821-07 | -0.02269 | 0.419571 | 0.409476 | 0.301624 | 0.442952 | 0.523439 | 0.5953 | 0.46883 | 0.294969 | 0.216777 | 0.016372 | 0.457936 | 0.038592 | 0.458064 | 0.109566 | 0.183191 | 0.318231 | 0.283742 | 0.515221 | 0.375148 | 0.2027 | -0.0363 | 0.471421 | 0.272445 | 0.255621 | 0.312019 | 0.196541 | 0.282348 |
| TCGA-CM-6168-01A-11R-1653-07 | 0.166746 | 0.499089 | 0.45805 | 0.439694 | 0.456099 | 0.529495 | 0.677219 | 0.595786 | 0.391459 | 0.410313 | 0.193811 | 0.554817 | 0.28733 | 0.53021 | 0.313553 | 0.333778 | 0.581725 | 0.311615 | 0.553671 | 0.503908 | 0.32381 | 0.148992 | 0.554259 | 0.517636 | 0.38669 | 0.400508 | 0.279393 | 0.356706 |
| TCGA-AA-3852-01A-01R-0905-07 | 0.097458 | 0.428384 | 0.472888 | 0.386488 | 0.448929 | 0.611953 | 0.674681 | 0.53446 | 0.333268 | 0.349244 | 0.154836 | 0.508687 | 0.166431 | 0.51235 | 0.258581 | 0.216424 | 0.554872 | 0.237352 | 0.556891 | 0.451278 | 0.265731 | 0.139623 | 0.529808 | 0.411267 | 0.305966 | 0.342844 | 0.356772 | 0.337067 |
| TCGA-AA-3851-01A-01R-1022-07 | 0.148126 | 0.465475 | 0.495443 | 0.376081 | 0.476703 | 0.583746 | 0.675808 | 0.508425 | 0.328441 | 0.376103 | 0.126106 | 0.487411 | 0.230066 | 0.517109 | 0.263966 | 0.249187 | 0.513683 | 0.270068 | 0.549293 | 0.423161 | 0.256755 | 0.122397 | 0.522617 | 0.387436 | 0.336531 | 0.359862 | 0.307319 | 0.317102 |
| TCGA-A6-6654-01A-21R-1839-07 | 0.161285 | 0.494679 | 0.536468 | 0.444514 | 0.496158 | 0.556038 | 0.701962 | 0.609979 | 0.398577 | 0.478816 | 0.234281 | 0.567524 | 0.304838 | 0.522399 | 0.382556 | 0.390345 | 0.677828 | 0.305042 | 0.576761 | 0.53194 | 0.338516 | 0.106174 | 0.578314 | 0.5941 | 0.419304 | 0.436044 | 0.21956 | 0.331961 |
| TCGA-DM-A28E-01A-11R-A32Y-07 | -0.13028 | 0.342842 | 0.356267 | 0.272178 | 0.455363 | 0.509021 | 0.589611 | 0.399693 | 0.258887 | 0.190534 | 0.083211 | 0.377724 | -0.00012 | 0.418075 | 0.087653 | 0.101117 | 0.284127 | 0.279314 | 0.530599 | 0.350988 | 0.147422 | -0.07018 | 0.44865 | 0.209929 | 0.206027 | 0.254047 | 0.237419 | 0.258292 |
| TCGA-AA-3509-01A-01R-1410-07 | 0.012959 | 0.449657 | 0.45193 | 0.367678 | 0.461243 | 0.521261 | 0.632913 | 0.526616 | 0.341877 | 0.306526 | 0.144676 | 0.490772 | 0.124729 | 0.469671 | 0.239798 | 0.210304 | 0.487357 | 0.266306 | 0.568924 | 0.434578 | 0.243539 | 0.158205 | 0.511171 | 0.370353 | 0.302257 | 0.35005 | 0.263651 | 0.304754 |
| TCGA-AA-3970-01A-01R-1022-07 | 0.130956 | 0.463781 | 0.49143 | 0.361402 | 0.470962 | 0.55216 | 0.63395 | 0.473329 | 0.330348 | 0.324616 | 0.173657 | 0.503028 | 0.194346 | 0.501863 | 0.215793 | 0.209525 | 0.462969 | 0.256005 | 0.544747 | 0.428161 | 0.241062 | 0.101912 | 0.51862 | 0.376778 | 0.320932 | 0.342216 | 0.338441 | 0.29745 |
| TCGA-CK-5914-01A-11R-1653-07 | -0.07471 | 0.468894 | 0.485359 | 0.350296 | 0.464921 | 0.511178 | 0.62454 | 0.48263 | 0.323523 | 0.325971 | 0.121396 | 0.478869 | 0.051452 | 0.440067 | 0.142011 | 0.1306 | 0.396474 | 0.26269 | 0.496173 | 0.451771 | 0.272213 | 0.00242 | 0.478017 | 0.340593 | 0.271935 | 0.318835 | 0.177587 | 0.25969 |
| TCGA-A6-5666-01A-01R-1653-07 | -0.14593 | 0.401097 | 0.383043 | 0.343307 | 0.457299 | 0.468571 | 0.579986 | 0.495191 | 0.314958 | 0.225559 | 0.104736 | 0.49229 | -0.00206 | 0.501038 | 0.152058 | 0.149836 | 0.387485 | 0.253263 | 0.525565 | 0.381193 | 0.177941 | 0.062702 | 0.489958 | 0.272333 | 0.229179 | 0.275295 | 0.180857 | 0.248084 |
| TCGA-4N-A93T-01A-11R-A37K-07 | 0.062859 | 0.308952 | 0.38335 | 0.271064 | 0.448305 | 0.552644 | 0.594532 | 0.407759 | 0.267618 | 0.232002 | 0.097648 | 0.4056 | 0.126195 | 0.450788 | 0.080633 | 0.021389 | 0.301146 | 0.23769 | 0.538411 | 0.3569 | 0.163758 | -0.10195 | 0.455338 | 0.146597 | 0.234051 | 0.281904 | 0.208381 | 0.175681 |
| TCGA-CM-6171-01A-11R-1653-07 | 0.012939 | 0.46767 | 0.420027 | 0.37638 | 0.421316 | 0.543951 | 0.673559 | 0.547998 | 0.335337 | 0.326254 | 0.13558 | 0.485556 | 0.140524 | 0.481792 | 0.216169 | 0.113827 | 0.52141 | 0.247067 | 0.548674 | 0.41761 | 0.240986 | 0.201741 | 0.539603 | 0.402451 | 0.325738 | 0.295273 | 0.277212 | 0.389196 |
| TCGA-G4-6309-01A-21R-1839-07 | 0.006919 | 0.469829 | 0.443404 | 0.340049 | 0.435186 | 0.565273 | 0.620153 | 0.482969 | 0.313393 | 0.360297 | 0.089497 | 0.466896 | 0.088902 | 0.512771 | 0.177898 | 0.111446 | 0.399398 | 0.219534 | 0.533176 | 0.397911 | 0.249919 | 0.06695 | 0.51111 | 0.297896 | 0.299211 | 0.307084 | 0.270144 | 0.351264 |
| TCGA-AA-3697-01A-01R-1723-07 | 0.085247 | 0.45022 | 0.449289 | 0.371484 | 0.453914 | 0.582457 | 0.642315 | 0.496344 | 0.307895 | 0.332804 | 0.143298 | 0.503701 | 0.177003 | 0.460129 | 0.198574 | 0.272551 | 0.457225 | 0.30871 | 0.545464 | 0.434059 | 0.274626 | 0.133695 | 0.525634 | 0.38028 | 0.32002 | 0.330634 | 0.258816 | 0.28317 |
| TCGA-G4-6586-01A-11R-1774-07 | -0.0038 | 0.464312 | 0.566876 | 0.399933 | 0.443146 | 0.55941 | 0.66505 | 0.482529 | 0.241442 | 0.332391 | 0.091008 | 0.460246 | 0.117037 | 0.446655 | 0.185234 | 0.022559 | 0.456014 | 0.220576 | 0.556016 | 0.410516 | 0.238141 | 0.068911 | 0.492625 | 0.287491 | 0.289158 | 0.32541 | 0.290575 | 0.338089 |
| TCGA-AZ-4616-01A-21R-1839-07 | -0.02641 | 0.484251 | 0.52195 | 0.389435 | 0.46578 | 0.568796 | 0.654508 | 0.513676 | 0.303779 | 0.367769 | 0.107816 | 0.492748 | 0.119775 | 0.482654 | 0.180279 | 0.170817 | 0.464732 | 0.202497 | 0.533121 | 0.439698 | 0.274489 | 0.084056 | 0.523956 | 0.319415 | 0.295597 | 0.330821 | 0.263762 | 0.315454 |
| TCGA-D5-6928-01A-11R-1928-07 | 0.417187 | 0.605047 | 0.685569 | 0.459111 | 0.446478 | 0.592747 | 0.745268 | 0.615571 | 0.41101 | 0.583799 | 0.201012 | 0.546936 | 0.470524 | 0.540208 | 0.389331 | 0.317513 | 0.72791 | 0.34939 | 0.583442 | 0.560555 | 0.373461 | 0.258087 | 0.586534 | 0.566243 | 0.446854 | 0.455952 | 0.291709 | 0.426663 |
| TCGA-AA-A02J-01A-01R-A00A-07 | -0.16337 | 0.367418 | 0.338582 | 0.261994 | 0.423247 | 0.530294 | 0.565017 | 0.437819 | 0.251316 | 0.143058 | 0.083315 | 0.434093 | -0.04954 | 0.415734 | 0.064891 | 0.10471 | 0.276864 | 0.251933 | 0.54504 | 0.337712 | 0.150151 | 0.005248 | 0.480458 | 0.225953 | 0.223123 | 0.257281 | 0.277475 | 0.227916 |
| TCGA-D5-6538-01A-11R-1723-07 | -0.1565 | 0.293462 | 0.305292 | 0.250213 | 0.431973 | 0.55467 | 0.593922 | 0.472354 | 0.278905 | 0.184852 | 0.049694 | 0.42025 | -0.06793 | 0.465795 | 0.131558 | 0.121506 | 0.25567 | 0.285537 | 0.568097 | 0.386326 | 0.178938 | -0.02696 | 0.50094 | 0.232261 | 0.243175 | 0.266239 | 0.198432 | 0.314989 |
| TCGA-NH-A6GB-01A-11R-A37K-07 | 0.009367 | 0.459187 | 0.473997 | 0.340396 | 0.448836 | 0.552905 | 0.641751 | 0.477997 | 0.295185 | 0.367073 | 0.175438 | 0.48467 | 0.140942 | 0.440674 | 0.209608 | 0.137687 | 0.464584 | 0.301078 | 0.526527 | 0.410185 | 0.239169 | 0.194379 | 0.527787 | 0.329401 | 0.290859 | 0.328496 | 0.323101 | 0.321888 |
| TCGA-A6-2682-01A-01R-1410-07 | -0.01155 | 0.400787 | 0.461832 | 0.394496 | 0.454519 | 0.592887 | 0.645594 | 0.532208 | 0.327774 | 0.276302 | 0.105592 | 0.512218 | 0.035516 | 0.515322 | 0.224527 | 0.25646 | 0.495089 | 0.193035 | 0.585292 | 0.455625 | 0.26835 | 0.200109 | 0.552306 | 0.437658 | 0.321601 | 0.381922 | 0.298962 | 0.282136 |
| TCGA-AA-A02Y-01A-43R-A32Y-07 | -0.0391 | 0.43682 | 0.491394 | 0.36935 | 0.471555 | 0.590319 | 0.606457 | 0.466015 | 0.277494 | 0.344141 | 0.09556 | 0.446096 | 0.098899 | 0.433354 | 0.16741 | 0.128184 | 0.435588 | 0.240336 | 0.586207 | 0.356048 | 0.219055 | 0.073597 | 0.493447 | 0.235024 | 0.28947 | 0.296764 | 0.323229 | 0.354069 |
| TCGA-AA-3850-01A-01R-1022-07 | 0.214322 | 0.46331 | 0.489399 | 0.395205 | 0.438068 | 0.570097 | 0.671209 | 0.489344 | 0.320519 | 0.380479 | 0.199709 | 0.524534 | 0.238149 | 0.502078 | 0.269059 | 0.182395 | 0.534669 | 0.295947 | 0.576671 | 0.435788 | 0.254222 | 0.225901 | 0.538719 | 0.417105 | 0.351135 | 0.362509 | 0.352338 | 0.31357 |
| TCGA-CK-6747-01A-11R-1839-07 | 0.041829 | 0.452845 | 0.465102 | 0.36544 | 0.44196 | 0.553719 | 0.639879 | 0.479328 | 0.310718 | 0.330933 | 0.094896 | 0.462182 | 0.129012 | 0.45382 | 0.198102 | 0.164907 | 0.436653 | 0.28949 | 0.55992 | 0.460616 | 0.242453 | 0.066228 | 0.495911 | 0.326845 | 0.342744 | 0.339942 | 0.295828 | 0.331641 |
| TCGA-A6-6782-01A-11R-1839-07 | 0.103092 | 0.444352 | 0.510107 | 0.374994 | 0.47714 | 0.561436 | 0.666773 | 0.529854 | 0.365231 | 0.371135 | 0.20406 | 0.521866 | 0.195974 | 0.497771 | 0.268747 | 0.288734 | 0.544011 | 0.311347 | 0.544022 | 0.476159 | 0.31643 | 0.112392 | 0.553835 | 0.443886 | 0.33698 | 0.357342 | 0.209883 | 0.278394 |
| TCGA-CM-5860-01A-01R-1653-07 | -0.02564 | 0.462677 | 0.441749 | 0.382078 | 0.4676 | 0.565233 | 0.653354 | 0.528965 | 0.363074 | 0.356346 | 0.159052 | 0.522164 | 0.153602 | 0.492924 | 0.276913 | 0.274917 | 0.497298 | 0.280045 | 0.536447 | 0.486822 | 0.320259 | 0.071472 | 0.531852 | 0.43635 | 0.366188 | 0.365867 | 0.268097 | 0.352413 |
| TCGA-AA-A00R-01A-01R-A002-07 | 0.216852 | 0.576944 | 0.661655 | 0.443843 | 0.445089 | 0.505831 | 0.679596 | 0.499053 | 0.317848 | 0.530651 | 0.110343 | 0.513898 | 0.302525 | 0.457184 | 0.316317 | 0.159734 | 0.611918 | 0.291182 | 0.572552 | 0.49844 | 0.33507 | 0.093732 | 0.502717 | 0.445978 | 0.391403 | 0.418141 | 0.283789 | 0.367382 |
| TCGA-A6-6653-01A-11R-1774-07 | -0.0239 | 0.472735 | 0.43749 | 0.426279 | 0.462884 | 0.584513 | 0.642944 | 0.509243 | 0.336059 | 0.378989 | 0.110606 | 0.497311 | 0.141544 | 0.454203 | 0.22524 | 0.114296 | 0.488357 | 0.275302 | 0.572762 | 0.457698 | 0.305745 | 0.183599 | 0.534297 | 0.365228 | 0.339812 | 0.31741 | 0.270338 | 0.331758 |
| TCGA-AD-6890-01A-11R-1928-07 | 0.078158 | 0.444263 | 0.457264 | 0.347026 | 0.451709 | 0.563939 | 0.635806 | 0.474178 | 0.309878 | 0.287376 | 0.107118 | 0.470014 | 0.103401 | 0.440895 | 0.176046 | 0.200559 | 0.412159 | 0.273296 | 0.548252 | 0.434379 | 0.243264 | 0.062192 | 0.498738 | 0.316934 | 0.285407 | 0.324697 | 0.274911 | 0.273683 |
| TCGA-D5-6539-01A-11R-1723-07 | 0.129116 | 0.416302 | 0.441604 | 0.378867 | 0.432444 | 0.555183 | 0.649456 | 0.483359 | 0.314683 | 0.341608 | 0.117252 | 0.515729 | 0.190573 | 0.497069 | 0.22194 | 0.194208 | 0.436274 | 0.304112 | 0.573056 | 0.444865 | 0.240907 | 0.17166 | 0.506107 | 0.344375 | 0.329006 | 0.335834 | 0.262503 | 0.321293 |
| TCGA-AD-A5EK-01A-11R-A28H-07 | -0.10937 | 0.383078 | 0.407171 | 0.330397 | 0.437643 | 0.51435 | 0.585124 | 0.472132 | 0.263222 | 0.232982 | 0.060654 | 0.397958 | -0.03329 | 0.395989 | 0.12781 | 0.158571 | 0.319956 | 0.282077 | 0.516133 | 0.419021 | 0.194682 | -0.06614 | 0.503251 | 0.278746 | 0.253281 | 0.26344 | 0.278615 | 0.317811 |
| TCGA-A6-3808-01A-01R-1022-07 | 0.099826 | 0.475275 | 0.494238 | 0.400791 | 0.475754 | 0.548075 | 0.683503 | 0.57438 | 0.394897 | 0.38607 | 0.235893 | 0.549027 | 0.228502 | 0.52045 | 0.307146 | 0.366074 | 0.576848 | 0.271056 | 0.566174 | 0.482931 | 0.30299 | 0.139959 | 0.54242 | 0.493458 | 0.385984 | 0.394315 | 0.311022 | 0.350811 |
| TCGA-AA-3506-01A-01R-1410-07 | 0.140874 | 0.385479 | 0.498202 | 0.384081 | 0.459978 | 0.611669 | 0.654168 | 0.522175 | 0.319797 | 0.368885 | 0.129471 | 0.510188 | 0.182297 | 0.501053 | 0.285331 | 0.242405 | 0.52402 | 0.235951 | 0.577946 | 0.452056 | 0.258837 | 0.174558 | 0.510155 | 0.41404 | 0.347198 | 0.358027 | 0.338313 | 0.313492 |
| TCGA-G4-6298-01A-11R-1723-07 | -0.08038 | 0.320246 | 0.310537 | 0.288186 | 0.464321 | 0.539463 | 0.626559 | 0.458358 | 0.298893 | 0.224308 | 0.095875 | 0.465573 | -0.02017 | 0.448747 | 0.131142 | 0.147956 | 0.317329 | 0.240322 | 0.519414 | 0.404091 | 0.233237 | -0.04197 | 0.511315 | 0.308794 | 0.246318 | 0.285983 | 0.228219 | 0.251376 |
| TCGA-AZ-6608-01A-11R-1839-07 | -0.17408 | 0.381371 | 0.367337 | 0.275216 | 0.452482 | 0.504099 | 0.584748 | 0.42421 | 0.239345 | 0.199835 | 0.051146 | 0.423735 | -0.03746 | 0.416112 | 0.091786 | 0.024586 | 0.284703 | 0.248025 | 0.514018 | 0.340486 | 0.165836 | -0.01778 | 0.454233 | 0.212884 | 0.227385 | 0.261047 | 0.239053 | 0.274795 |
| TCGA-AA-3678-01A-01R-0905-07 | 0.044355 | 0.435034 | 0.463688 | 0.398349 | 0.468275 | 0.555847 | 0.64602 | 0.492727 | 0.309842 | 0.355927 | 0.162298 | 0.496759 | 0.151991 | 0.501835 | 0.253235 | 0.200958 | 0.486635 | 0.290956 | 0.574559 | 0.430137 | 0.225406 | 0.185651 | 0.524452 | 0.380088 | 0.315907 | 0.348062 | 0.359155 | 0.311496 |
| TCGA-A6-5665-01A-01R-1653-07 | 0.109691 | 0.525598 | 0.534404 | 0.373501 | 0.418681 | 0.538698 | 0.663966 | 0.512655 | 0.309971 | 0.375808 | 0.13047 | 0.487602 | 0.203125 | 0.479968 | 0.164041 | 0.130453 | 0.474665 | 0.257411 | 0.538514 | 0.418041 | 0.290084 | 0.129996 | 0.476663 | 0.302357 | 0.311441 | 0.318781 | 0.29162 | 0.386566 |
| TCGA-G4-6306-01A-11R-1774-07 | -0.0179 | 0.39265 | 0.480089 | 0.335258 | 0.457603 | 0.574922 | 0.653001 | 0.450453 | 0.263398 | 0.297669 | 0.057294 | 0.421335 | 0.101895 | 0.430461 | 0.108381 | 0.109648 | 0.315727 | 0.211443 | 0.567636 | 0.336003 | 0.199603 | 0.058205 | 0.473924 | 0.20386 | 0.254336 | 0.286302 | 0.301614 | 0.255966 |
| TCGA-F4-6806-01A-11R-1839-07 | 0.149216 | 0.399021 | 0.44833 | 0.387145 | 0.470103 | 0.531542 | 0.644083 | 0.479405 | 0.301165 | 0.343299 | 0.154672 | 0.465342 | 0.199455 | 0.480103 | 0.196522 | 0.164627 | 0.428467 | 0.282121 | 0.55192 | 0.448448 | 0.239618 | 0.0696 | 0.54761 | 0.301611 | 0.297746 | 0.336621 | 0.33355 | 0.306951 |
| TCGA-AA-A01T-01A-21R-A16W-07 | -0.19975 | 0.285243 | 0.407753 | 0.317721 | 0.454106 | 0.612287 | 0.605993 | 0.382187 | 0.175719 | 0.265722 | -0.05983 | 0.428485 | -0.07254 | 0.420199 | 0.105223 | 0.026038 | 0.345571 | 0.185969 | 0.554981 | 0.353983 | 0.247298 | 0.071628 | 0.443888 | 0.229163 | 0.266308 | 0.252286 | 0.356683 | 0.172411 |
| TCGA-A6-6651-01A-21R-1839-07 | 0.282354 | 0.461301 | 0.496325 | 0.407982 | 0.46013 | 0.53499 | 0.683372 | 0.575612 | 0.429964 | 0.419737 | 0.196798 | 0.538944 | 0.312312 | 0.514464 | 0.36552 | 0.4054 | 0.63576 | 0.341833 | 0.560147 | 0.526905 | 0.347297 | 0.073183 | 0.578258 | 0.577184 | 0.41572 | 0.414208 | 0.180806 | 0.329435 |
| TCGA-AA-3812-01A-01R-0905-07 | 0.03172 | 0.262089 | 0.420502 | 0.40083 | 0.480792 | 0.649954 | 0.671069 | 0.555324 | 0.28749 | 0.381182 | 0.107681 | 0.505791 | 0.138304 | 0.508844 | 0.305491 | 0.309016 | 0.542719 | 0.210082 | 0.605199 | 0.472576 | 0.299569 | 0.141341 | 0.536368 | 0.502761 | 0.362268 | 0.375 | 0.292568 | 0.242065 |
| TCGA-F4-6809-01A-11R-1839-07 | 0.218875 | 0.439217 | 0.495659 | 0.377386 | 0.473219 | 0.551795 | 0.676571 | 0.515813 | 0.361404 | 0.374274 | 0.173367 | 0.508151 | 0.24828 | 0.462322 | 0.270923 | 0.265139 | 0.499144 | 0.312365 | 0.555416 | 0.483174 | 0.289594 | 0.020617 | 0.531445 | 0.387963 | 0.353028 | 0.372469 | 0.264191 | 0.306885 |
| TCGA-AD-5900-01A-11R-1653-07 | 0.196385 | 0.48157 | 0.557435 | 0.392463 | 0.433384 | 0.548219 | 0.682214 | 0.562588 | 0.371798 | 0.433445 | 0.087951 | 0.493663 | 0.290209 | 0.490763 | 0.303066 | 0.227246 | 0.564788 | 0.280486 | 0.563895 | 0.466634 | 0.334914 | 0.043936 | 0.488213 | 0.430692 | 0.362265 | 0.360338 | 0.227234 | 0.414357 |
| TCGA-D5-6930-01A-11R-1928-07 | 0.304293 | 0.505111 | 0.555836 | 0.416288 | 0.471037 | 0.568015 | 0.681464 | 0.528232 | 0.365733 | 0.445965 | 0.185407 | 0.495752 | 0.295152 | 0.513731 | 0.277867 | 0.25405 | 0.563111 | 0.30521 | 0.559791 | 0.480913 | 0.315072 | 0.062486 | 0.533056 | 0.381896 | 0.351484 | 0.377186 | 0.307457 | 0.383793 |
| TCGA-AA-A03J-01A-21R-A16W-07 | 0.050015 | 0.342754 | 0.510897 | 0.37407 | 0.459031 | 0.621928 | 0.644646 | 0.488155 | 0.266551 | 0.375247 | 0.09459 | 0.505886 | 0.152757 | 0.430338 | 0.232659 | 0.171118 | 0.481554 | 0.252691 | 0.584649 | 0.440575 | 0.262526 | 0.061686 | 0.508099 | 0.400834 | 0.341776 | 0.32961 | 0.31283 | 0.257709 |
| TCGA-AA-3815-01A-01R-1022-07 | 0.179969 | 0.51285 | 0.603545 | 0.460346 | 0.461924 | 0.578664 | 0.674621 | 0.542116 | 0.308874 | 0.452652 | 0.114533 | 0.504084 | 0.272183 | 0.477591 | 0.281645 | 0.246236 | 0.596064 | 0.25108 | 0.578114 | 0.471253 | 0.280822 | 0.159256 | 0.502628 | 0.411649 | 0.396099 | 0.393974 | 0.30464 | 0.33419 |
| TCGA-CM-6169-01A-11R-1653-07 | 0.121014 | 0.473617 | 0.520295 | 0.429864 | 0.462136 | 0.541482 | 0.682341 | 0.573743 | 0.392085 | 0.42429 | 0.214426 | 0.541049 | 0.245138 | 0.499099 | 0.318677 | 0.358054 | 0.60796 | 0.314225 | 0.561855 | 0.520765 | 0.345383 | 0.146328 | 0.580963 | 0.52459 | 0.412033 | 0.4013 | 0.287943 | 0.320024 |
| TCGA-F4-6855-01A-11R-1928-07 | -0.02168 | 0.378305 | 0.394477 | 0.369342 | 0.475795 | 0.550214 | 0.667514 | 0.567643 | 0.33904 | 0.361231 | 0.180743 | 0.504717 | 0.127561 | 0.48648 | 0.269972 | 0.240347 | 0.512439 | 0.261647 | 0.535088 | 0.494189 | 0.267338 | -0.05463 | 0.5485 | 0.439914 | 0.351512 | 0.353127 | 0.22357 | 0.301102 |
| TCGA-AG-3894-01A-01R-1119-07 | -0.05225 | 0.368881 | 0.426618 | 0.298946 | 0.464296 | 0.522013 | 0.654737 | 0.457957 | 0.321786 | 0.267845 | 0.091227 | 0.500746 | 0.070108 | 0.470032 | 0.187816 | 0.169571 | 0.441098 | 0.261825 | 0.545634 | 0.416894 | 0.220382 | 0.037343 | 0.518237 | 0.331723 | 0.299507 | 0.318186 | 0.291405 | 0.294886 |
| TCGA-AG-3885-01A-01R-0905-07 | 0.091588 | 0.325164 | 0.471715 | 0.351376 | 0.453772 | 0.637135 | 0.638648 | 0.507951 | 0.272949 | 0.353057 | 0.048753 | 0.460447 | 0.111264 | 0.457079 | 0.228198 | 0.155605 | 0.490279 | 0.223471 | 0.574758 | 0.414613 | 0.23947 | 0.163161 | 0.493506 | 0.342155 | 0.327153 | 0.344352 | 0.311136 | 0.282984 |
| TCGA-AG-3882-01A-01R-0905-07 | 0.196423 | 0.378139 | 0.588769 | 0.409185 | 0.458099 | 0.625833 | 0.668593 | 0.499282 | 0.251151 | 0.443206 | 0.118039 | 0.483614 | 0.236328 | 0.491281 | 0.271166 | 0.23002 | 0.571499 | 0.257835 | 0.589665 | 0.457495 | 0.266044 | 0.16762 | 0.523058 | 0.456763 | 0.360979 | 0.370367 | 0.311495 | 0.234791 |
| TCGA-AG-3726-01A-02R-0905-07 | -0.03986 | 0.278029 | 0.419185 | 0.365747 | 0.472158 | 0.633799 | 0.610434 | 0.508164 | 0.252832 | 0.320693 | 0.063198 | 0.442956 | 0.047246 | 0.434334 | 0.197575 | 0.159726 | 0.448415 | 0.222331 | 0.616246 | 0.380941 | 0.237664 | 0.083538 | 0.495613 | 0.341012 | 0.312061 | 0.339735 | 0.337056 | 0.224991 |
| TCGA-AF-4110-01A-02R-1736-07 | 0.213939 | 0.43063 | 0.50353 | 0.397864 | 0.470226 | 0.544335 | 0.679985 | 0.531085 | 0.363646 | 0.376297 | 0.182328 | 0.526669 | 0.23086 | 0.494966 | 0.309499 | 0.299168 | 0.534393 | 0.265863 | 0.570124 | 0.486504 | 0.30728 | 0.19567 | 0.555943 | 0.43334 | 0.356672 | 0.373865 | 0.283957 | 0.336355 |
| TCGA-AH-6644-01A-21R-1830-07 | 0.069644 | 0.410771 | 0.44833 | 0.37991 | 0.443735 | 0.493379 | 0.663208 | 0.546944 | 0.365297 | 0.303426 | 0.186294 | 0.506711 | 0.163339 | 0.486959 | 0.274409 | 0.348652 | 0.512888 | 0.278966 | 0.560918 | 0.471891 | 0.281011 | 0.111899 | 0.524413 | 0.406724 | 0.338505 | 0.354245 | 0.288574 | 0.315866 |
| TCGA-AG-A02N-01A-11R-A083-07 | 0.028907 | 0.474709 | 0.508999 | 0.359172 | 0.436281 | 0.554996 | 0.635698 | 0.476018 | 0.326505 | 0.347558 | 0.159715 | 0.453945 | 0.137889 | 0.489601 | 0.154085 | 0.175418 | 0.449411 | 0.249897 | 0.536969 | 0.410576 | 0.279005 | -0.00697 | 0.500524 | 0.289071 | 0.293807 | 0.308096 | 0.304021 | 0.308753 |
| TCGA-AG-3609-01A-02R-0826-07 | 0.040755 | 0.427597 | 0.48341 | 0.389997 | 0.467665 | 0.60346 | 0.642542 | 0.522125 | 0.317342 | 0.369081 | 0.116698 | 0.512838 | 0.131996 | 0.490932 | 0.269525 | 0.230684 | 0.505404 | 0.250489 | 0.560594 | 0.471656 | 0.274709 | 0.095888 | 0.540102 | 0.418313 | 0.342173 | 0.369207 | 0.2792 | 0.302996 |
| TCGA-AG-A01J-01A-01R-A00A-07 | 0.006418 | 0.323105 | 0.453019 | 0.306794 | 0.424599 | 0.58704 | 0.614388 | 0.452937 | 0.247988 | 0.256214 | 0.030866 | 0.481951 | 0.09575 | 0.444326 | 0.160992 | 0.147084 | 0.375508 | 0.284965 | 0.58637 | 0.36502 | 0.192832 | 0.068862 | 0.518327 | 0.263991 | 0.290843 | 0.301923 | 0.315816 | 0.215921 |
| TCGA-AG-4022-01A-01R-1736-07 | 0.125408 | 0.418717 | 0.448304 | 0.404284 | 0.452588 | 0.544282 | 0.669618 | 0.530359 | 0.359006 | 0.362863 | 0.232787 | 0.509557 | 0.220619 | 0.490551 | 0.271531 | 0.319909 | 0.506517 | 0.300362 | 0.55736 | 0.473464 | 0.295389 | 0.115424 | 0.561965 | 0.432024 | 0.338591 | 0.368415 | 0.268248 | 0.297014 |
| TCGA-AG-4021-01A-01R-1736-07 | -0.07689 | 0.408389 | 0.353267 | 0.387323 | 0.458929 | 0.570065 | 0.656869 | 0.511065 | 0.32871 | 0.332408 | 0.070416 | 0.517788 | 0.055318 | 0.494346 | 0.205477 | 0.227305 | 0.496106 | 0.288705 | 0.539361 | 0.443802 | 0.276068 | 0.10142 | 0.529882 | 0.437511 | 0.320803 | 0.34393 | 0.216843 | 0.34603 |
| TCGA-G5-6233-01A-11R-1736-07 | -0.02707 | 0.430831 | 0.383313 | 0.357102 | 0.449723 | 0.577877 | 0.661377 | 0.486721 | 0.326798 | 0.30316 | 0.096391 | 0.477141 | 0.099528 | 0.506858 | 0.218432 | 0.186753 | 0.418045 | 0.275456 | 0.522706 | 0.431406 | 0.277969 | 0.072627 | 0.528441 | 0.35694 | 0.299015 | 0.30906 | 0.284504 | 0.342199 |
| TCGA-AF-6655-01A-11R-1830-07 | -0.0416 | 0.428965 | 0.38796 | 0.372931 | 0.450465 | 0.526317 | 0.644078 | 0.539914 | 0.354224 | 0.291778 | 0.183271 | 0.475154 | 0.083713 | 0.479448 | 0.219568 | 0.19881 | 0.444313 | 0.264351 | 0.540735 | 0.44533 | 0.264314 | 0.098952 | 0.547805 | 0.37475 | 0.282489 | 0.328466 | 0.302616 | 0.300147 |
| TCGA-A6-A566-01A-11R-A28H-07 | 0.038764 | 0.483815 | 0.495304 | 0.410717 | 0.480654 | 0.596834 | 0.703142 | 0.574328 | 0.385946 | 0.448497 | 0.158597 | 0.578595 | 0.207779 | 0.549767 | 0.367859 | 0.314142 | 0.648986 | 0.311466 | 0.551059 | 0.533564 | 0.350028 | 0.15795 | 0.565753 | 0.602517 | 0.406508 | 0.422715 | 0.270728 | 0.361112 |
| TCGA-CA-6715-01A-21R-1839-07 | -0.13673 | 0.377507 | 0.356719 | 0.310013 | 0.448611 | 0.509422 | 0.575142 | 0.469268 | 0.296971 | 0.17449 | 0.083843 | 0.43059 | -0.0254 | 0.462929 | 0.119832 | 0.090883 | 0.316278 | 0.318518 | 0.536578 | 0.401604 | 0.17353 | 0.032529 | 0.511845 | 0.2667 | 0.212491 | 0.277789 | 0.233438 | 0.254236 |
| TCGA-NH-A6GA-01A-11R-A37K-07 | -0.00769 | 0.410873 | 0.411748 | 0.331606 | 0.442012 | 0.55694 | 0.659661 | 0.481239 | 0.314014 | 0.279769 | 0.066822 | 0.459762 | 0.094035 | 0.472947 | 0.180733 | 0.10796 | 0.40425 | 0.293413 | 0.536089 | 0.406702 | 0.240224 | 0.089124 | 0.488192 | 0.293781 | 0.29283 | 0.309098 | 0.213764 | 0.301703 |
| TCGA-AA-A004-01A-01R-A00A-07 | 0.171006 | 0.321982 | 0.508464 | 0.362024 | 0.46616 | 0.657119 | 0.643294 | 0.478525 | 0.182347 | 0.346787 | 0.072858 | 0.453287 | 0.136654 | 0.437299 | 0.232816 | 0.108362 | 0.489979 | 0.204262 | 0.56281 | 0.416248 | 0.26144 | 0.043807 | 0.501518 | 0.304525 | 0.349711 | 0.323238 | 0.372558 | 0.282289 |
| TCGA-AD-6889-01A-11R-1928-07 | -0.11102 | 0.442131 | 0.429614 | 0.388454 | 0.466107 | 0.56299 | 0.62485 | 0.509178 | 0.295092 | 0.33288 | 0.10832 | 0.475163 | 0.054458 | 0.432919 | 0.170861 | 0.098141 | 0.442011 | 0.269698 | 0.509928 | 0.42301 | 0.237017 | 0.084432 | 0.486536 | 0.319892 | 0.287525 | 0.293918 | 0.290072 | 0.370343 |
| TCGA-D5-6536-01A-11R-1723-07 | -0.11087 | 0.359409 | 0.315959 | 0.418169 | 0.449267 | 0.570066 | 0.678984 | 0.551384 | 0.385403 | 0.303627 | 0.097756 | 0.505906 | 0.033672 | 0.533757 | 0.273165 | 0.1832 | 0.535338 | 0.186686 | 0.551313 | 0.455945 | 0.268492 | 0.18277 | 0.574235 | 0.443637 | 0.298268 | 0.324667 | 0.242008 | 0.283336 |
| TCGA-G4-6322-01A-11R-1723-07 | -0.04154 | 0.433854 | 0.449159 | 0.343975 | 0.462671 | 0.595121 | 0.656397 | 0.452068 | 0.321454 | 0.29866 | 0.143163 | 0.462518 | 0.109913 | 0.429958 | 0.177034 | 0.10545 | 0.441804 | 0.269109 | 0.528899 | 0.421528 | 0.248434 | 0.04607 | 0.495822 | 0.295946 | 0.247546 | 0.300668 | 0.336002 | 0.362856 |
| TCGA-AA-A029-01A-01R-A00A-07 | -0.14112 | 0.36931 | 0.425058 | 0.286934 | 0.417955 | 0.5947 | 0.617445 | 0.472491 | 0.258381 | 0.296472 | 0.058689 | 0.443609 | 0.035872 | 0.422009 | 0.08211 | 0.152079 | 0.334675 | 0.2017 | 0.552849 | 0.368655 | 0.184156 | 0.083933 | 0.482683 | 0.301812 | 0.274978 | 0.274744 | 0.263446 | 0.295319 |
| TCGA-AZ-5407-01A-01R-1723-07 | 0.12365 | 0.457909 | 0.500191 | 0.36816 | 0.457584 | 0.536069 | 0.641904 | 0.473702 | 0.295964 | 0.342317 | 0.179681 | 0.482794 | 0.18524 | 0.484117 | 0.213645 | 0.231633 | 0.43907 | 0.285125 | 0.55398 | 0.399648 | 0.220637 | 0.056615 | 0.494809 | 0.310583 | 0.284199 | 0.337156 | 0.301531 | 0.377628 |
| TCGA-AA-A00N-01A-02R-A00A-07 | -0.04622 | 0.385308 | 0.4198 | 0.361854 | 0.464818 | 0.586597 | 0.665011 | 0.573116 | 0.33664 | 0.348446 | 0.087623 | 0.528548 | 0.121427 | 0.515535 | 0.290729 | 0.176432 | 0.545292 | 0.266319 | 0.544119 | 0.464725 | 0.279892 | 0.175705 | 0.539857 | 0.441879 | 0.326909 | 0.379992 | 0.287242 | 0.305335 |
| TCGA-D5-6540-01A-11R-1723-07 | -0.04069 | 0.466673 | 0.480912 | 0.397127 | 0.411026 | 0.544801 | 0.677191 | 0.532439 | 0.325928 | 0.382903 | 0.071818 | 0.476424 | 0.097986 | 0.481317 | 0.231331 | 0.176024 | 0.481649 | 0.282057 | 0.557302 | 0.452974 | 0.307121 | 0.206288 | 0.50755 | 0.387443 | 0.317528 | 0.350375 | 0.270966 | 0.428753 |
| TCGA-AA-3548-01A-01R-1873-07 | 0.172038 | 0.380419 | 0.516816 | 0.319666 | 0.482357 | 0.585063 | 0.661659 | 0.496665 | 0.251984 | 0.337869 | 0.088866 | 0.49892 | 0.208828 | 0.493173 | 0.231785 | 0.16659 | 0.487012 | 0.261381 | 0.556859 | 0.425015 | 0.267883 | -0.01434 | 0.519688 | 0.378361 | 0.33222 | 0.353709 | 0.274729 | 0.272219 |
| TCGA-A6-6649-01A-11R-1774-07 | 0.139105 | 0.444211 | 0.436469 | 0.387839 | 0.440306 | 0.598892 | 0.692658 | 0.545053 | 0.366413 | 0.357716 | 0.140352 | 0.480466 | 0.224303 | 0.514047 | 0.218708 | 0.287548 | 0.506463 | 0.319267 | 0.563908 | 0.463369 | 0.288987 | 0.166452 | 0.518845 | 0.380297 | 0.333353 | 0.345081 | 0.249004 | 0.397893 |
| TCGA-DM-A282-01A-12R-A16W-07 | -0.11371 | 0.330102 | 0.350201 | 0.300593 | 0.441854 | 0.523826 | 0.607404 | 0.432415 | 0.265714 | 0.216902 | 0.062291 | 0.421085 | -0.04553 | 0.40596 | 0.153236 | 0.109853 | 0.314616 | 0.254908 | 0.522949 | 0.377724 | 0.170424 | -0.08815 | 0.515935 | 0.239528 | 0.214606 | 0.252932 | 0.223304 | 0.270696 |
| TCGA-AA-3655-01A-02R-1723-07 | -0.02726 | 0.46191 | 0.429084 | 0.387148 | 0.470618 | 0.551768 | 0.642518 | 0.501715 | 0.32016 | 0.345451 | 0.106193 | 0.48779 | 0.101302 | 0.54344 | 0.202801 | 0.189783 | 0.426677 | 0.288642 | 0.577859 | 0.437462 | 0.239743 | 0.048421 | 0.521907 | 0.373308 | 0.319161 | 0.321481 | 0.280857 | 0.348623 |
| TCGA-G4-6311-01A-11R-1723-07 | 0.239458 | 0.449349 | 0.394163 | 0.397316 | 0.461704 | 0.554221 | 0.66255 | 0.535824 | 0.34604 | 0.319341 | 0.194688 | 0.51338 | 0.306345 | 0.493161 | 0.250621 | 0.21314 | 0.487046 | 0.341382 | 0.536713 | 0.463885 | 0.279347 | 0.068817 | 0.53873 | 0.435062 | 0.33541 | 0.351366 | 0.234864 | 0.265859 |
| TCGA-G4-6297-01A-11R-1723-07 | 0.113017 | 0.413394 | 0.427409 | 0.40353 | 0.441668 | 0.563115 | 0.667757 | 0.544113 | 0.347595 | 0.380171 | 0.179322 | 0.512915 | 0.221753 | 0.480613 | 0.273459 | 0.306084 | 0.521544 | 0.298858 | 0.547475 | 0.488378 | 0.293283 | 0.100158 | 0.555889 | 0.447791 | 0.337694 | 0.374475 | 0.24285 | 0.285266 |
| TCGA-5M-AAT6-01A-11R-A41B-07 | 0.1496 | 0.525812 | 0.570196 | 0.41563 | 0.465056 | 0.52353 | 0.690879 | 0.499088 | 0.352622 | 0.485755 | 0.125864 | 0.510522 | 0.266204 | 0.563159 | 0.310481 | 0.168531 | 0.594628 | 0.288095 | 0.538648 | 0.493234 | 0.329364 | 0.031708 | 0.522872 | 0.49099 | 0.400527 | 0.390141 | 0.211296 | 0.353945 |
| TCGA-D5-6534-01A-21R-1928-07 | 0.235745 | 0.414017 | 0.528842 | 0.465132 | 0.465376 | 0.59251 | 0.70937 | 0.593215 | 0.406098 | 0.504488 | 0.200675 | 0.585247 | 0.337472 | 0.538296 | 0.387541 | 0.378942 | 0.69393 | 0.299972 | 0.588615 | 0.554915 | 0.354438 | 0.12602 | 0.562445 | 0.62106 | 0.467702 | 0.440357 | 0.292187 | 0.334521 |
| TCGA-DM-A1DB-01A-11R-A155-07 | -0.07121 | 0.43764 | 0.480744 | 0.323765 | 0.426733 | 0.508617 | 0.592479 | 0.431045 | 0.270165 | 0.25571 | 0.091018 | 0.446364 | 0.046665 | 0.411087 | 0.11632 | 0.144355 | 0.317591 | 0.282524 | 0.55978 | 0.3602 | 0.190459 | -0.05269 | 0.496975 | 0.240035 | 0.226926 | 0.254481 | 0.187521 | 0.238384 |
| TCGA-CM-6678-01A-11R-1839-07 | -0.07801 | 0.335363 | 0.374015 | 0.317678 | 0.468513 | 0.549324 | 0.628028 | 0.477377 | 0.296189 | 0.23281 | 0.097765 | 0.444475 | -0.00018 | 0.473578 | 0.182267 | 0.123017 | 0.362179 | 0.267228 | 0.544146 | 0.406258 | 0.2043 | 0.05373 | 0.526681 | 0.2771 | 0.277552 | 0.302058 | 0.27336 | 0.320962 |
| TCGA-AA-3660-01A-01R-1723-07 | 0.041923 | 0.404391 | 0.406949 | 0.369391 | 0.491405 | 0.555478 | 0.636432 | 0.488435 | 0.264732 | 0.292008 | 0.118529 | 0.50795 | 0.13895 | 0.483135 | 0.219808 | 0.219956 | 0.430114 | 0.302226 | 0.551556 | 0.426638 | 0.238248 | 0.084339 | 0.542083 | 0.35768 | 0.317442 | 0.322234 | 0.272924 | 0.281996 |
| TCGA-DM-A0XF-01A-11R-A155-07 | -0.13063 | 0.40473 | 0.443759 | 0.355935 | 0.458075 | 0.51857 | 0.631571 | 0.457938 | 0.299726 | 0.303883 | 0.089308 | 0.468767 | 0.043402 | 0.468689 | 0.150741 | 0.092955 | 0.400916 | 0.251405 | 0.566358 | 0.413782 | 0.253146 | 0.014034 | 0.493462 | 0.302038 | 0.319435 | 0.312769 | 0.279334 | 0.281085 |
| TCGA-QG-A5Z1-01A-11R-A28H-07 | -0.07541 | 0.349621 | 0.330578 | 0.334732 | 0.453095 | 0.520475 | 0.625682 | 0.466924 | 0.340556 | 0.293923 | 0.128249 | 0.45414 | 0.067546 | 0.449066 | 0.177828 | 0.139468 | 0.387965 | 0.307001 | 0.514878 | 0.475259 | 0.231294 | -0.15583 | 0.527877 | 0.333792 | 0.301731 | 0.282449 | 0.223187 | 0.236858 |
| TCGA-AG-4015-01A-01R-1119-07 | 0.05701 | 0.379762 | 0.434658 | 0.340473 | 0.445507 | 0.547517 | 0.628273 | 0.475455 | 0.250315 | 0.285536 | 0.079049 | 0.493093 | 0.132982 | 0.464305 | 0.168178 | 0.128359 | 0.418889 | 0.291704 | 0.56379 | 0.413883 | 0.253223 | 0.024122 | 0.501252 | 0.343484 | 0.320669 | 0.305502 | 0.286278 | 0.233923 |
| TCGA-AG-3731-01A-11R-1736-07 | 0.205052 | 0.520978 | 0.522663 | 0.423537 | 0.445187 | 0.53196 | 0.658325 | 0.589087 | 0.426713 | 0.418777 | 0.233231 | 0.536809 | 0.284049 | 0.498366 | 0.317508 | 0.328633 | 0.58126 | 0.31482 | 0.556297 | 0.495555 | 0.321618 | 0.124011 | 0.563758 | 0.501811 | 0.367551 | 0.400571 | 0.304996 | 0.346354 |
| TCGA-AG-3611-01A-01R-0826-07 | 0.036721 | 0.352461 | 0.451926 | 0.36784 | 0.454309 | 0.602885 | 0.670593 | 0.457091 | 0.28641 | 0.331364 | 0.116283 | 0.461421 | 0.103366 | 0.511646 | 0.291218 | 0.099573 | 0.467025 | 0.219207 | 0.555118 | 0.383993 | 0.196878 | 0.172217 | 0.522637 | 0.299929 | 0.283987 | 0.285993 | 0.289267 | 0.325232 |
| TCGA-EI-6885-01A-11R-1928-07 | 0.034601 | 0.453146 | 0.457826 | 0.391468 | 0.503285 | 0.572441 | 0.656993 | 0.535024 | 0.328991 | 0.323764 | 0.192103 | 0.514415 | 0.139961 | 0.50241 | 0.268024 | 0.371231 | 0.494343 | 0.315506 | 0.541429 | 0.483093 | 0.293218 | 0.080433 | 0.559965 | 0.432861 | 0.354404 | 0.369555 | 0.268083 | 0.3363 |
| TCGA-AG-4007-01A-01R-1119-07 | 0.118726 | 0.394718 | 0.505719 | 0.354551 | 0.458247 | 0.601868 | 0.663457 | 0.517938 | 0.29091 | 0.371834 | 0.165132 | 0.51046 | 0.174 | 0.482347 | 0.231329 | 0.25951 | 0.52486 | 0.280338 | 0.533697 | 0.455767 | 0.282486 | 0.137157 | 0.53896 | 0.417965 | 0.333563 | 0.360885 | 0.309604 | 0.247352 |
| TCGA-AG-3581-01A-01R-0821-07 | 0.117817 | 0.370703 | 0.442651 | 0.375206 | 0.464507 | 0.590237 | 0.636494 | 0.523897 | 0.330081 | 0.325206 | 0.084221 | 0.508653 | 0.178396 | 0.483243 | 0.254036 | 0.222156 | 0.477776 | 0.264534 | 0.57871 | 0.427124 | 0.248568 | 0.186803 | 0.53793 | 0.400486 | 0.342158 | 0.344244 | 0.328643 | 0.294389 |
| TCGA-AA-3526-01A-02R-A32Z-07 | 0.076888 | 0.5092 | 0.517986 | 0.424451 | 0.450769 | 0.532631 | 0.650505 | 0.498439 | 0.324178 | 0.377343 | 0.15558 | 0.527507 | 0.164666 | 0.490129 | 0.245412 | 0.208986 | 0.494345 | 0.254292 | 0.53371 | 0.446917 | 0.269658 | 0.100977 | 0.507857 | 0.400154 | 0.340151 | 0.356413 | 0.259986 | 0.333602 |
| TCGA-CA-5255-01A-11R-1839-07 | -0.0947 | 0.401663 | 0.404141 | 0.322835 | 0.462829 | 0.539054 | 0.614162 | 0.472796 | 0.271485 | 0.278196 | 0.043455 | 0.437663 | 0.079614 | 0.463713 | 0.150809 | 0.172526 | 0.31957 | 0.251384 | 0.576379 | 0.37612 | 0.220124 | 0.123788 | 0.482643 | 0.234946 | 0.271956 | 0.293119 | 0.289624 | 0.370117 |
| TCGA-AA-3977-01A-01R-1022-07 | -0.01027 | 0.499996 | 0.530084 | 0.393563 | 0.479335 | 0.524732 | 0.657022 | 0.496461 | 0.358091 | 0.362378 | 0.085515 | 0.52826 | 0.145962 | 0.515908 | 0.269935 | 0.230092 | 0.51615 | 0.239159 | 0.561783 | 0.442175 | 0.283438 | 0.084051 | 0.515643 | 0.418853 | 0.342982 | 0.363212 | 0.285671 | 0.34115 |
| TCGA-AA-A01F-01A-01R-A002-07 | -0.23253 | 0.315368 | 0.407028 | 0.29525 | 0.422301 | 0.54184 | 0.621522 | 0.450433 | 0.245716 | 0.187058 | 0.039352 | 0.416719 | -0.03478 | 0.409393 | 0.070344 | 0.04991 | 0.335736 | 0.238703 | 0.584834 | 0.344248 | 0.173843 | 0.12223 | 0.479706 | 0.203472 | 0.224871 | 0.279153 | 0.265054 | 0.295475 |
| TCGA-D5-5540-01A-01R-1653-07 | -0.12589 | 0.508117 | 0.502296 | 0.35353 | 0.471181 | 0.494628 | 0.610068 | 0.426375 | 0.335109 | 0.241425 | 0.105153 | 0.47462 | 0.042311 | 0.477027 | 0.191246 | 0.12409 | 0.421985 | 0.245202 | 0.487646 | 0.416732 | 0.26861 | -0.03189 | 0.478935 | 0.344777 | 0.284779 | 0.312369 | 0.189748 | 0.307766 |
| TCGA-5M-AAT4-01A-11R-A41B-07 | -0.14 | 0.39168 | 0.369019 | 0.331441 | 0.445128 | 0.481949 | 0.583214 | 0.467478 | 0.261567 | 0.210224 | 0.053371 | 0.439933 | -0.00226 | 0.43858 | 0.130612 | 0.067355 | 0.336762 | 0.249746 | 0.517622 | 0.381749 | 0.206194 | 0.016164 | 0.512396 | 0.257519 | 0.251937 | 0.275372 | 0.194279 | 0.263258 |
| TCGA-AA-3864-01A-01R-1022-07 | 0.046916 | 0.487205 | 0.446911 | 0.362575 | 0.450211 | 0.544587 | 0.649432 | 0.509727 | 0.336509 | 0.32024 | 0.146998 | 0.509168 | 0.147302 | 0.47536 | 0.230386 | 0.19171 | 0.485855 | 0.24573 | 0.535935 | 0.440049 | 0.273418 | 0.123868 | 0.508065 | 0.367874 | 0.314197 | 0.339295 | 0.228492 | 0.344232 |
| TCGA-CA-6717-01A-11R-1839-07 | 0.281677 | 0.507059 | 0.529639 | 0.422276 | 0.467224 | 0.542709 | 0.674628 | 0.568728 | 0.433819 | 0.425713 | 0.182085 | 0.525562 | 0.330249 | 0.530293 | 0.321857 | 0.338272 | 0.610914 | 0.309392 | 0.567669 | 0.503619 | 0.345551 | 0.05959 | 0.536168 | 0.520532 | 0.403612 | 0.40069 | 0.213979 | 0.374074 |
| TCGA-CM-6161-01A-11R-1653-07 | 0.04742 | 0.47208 | 0.488099 | 0.376565 | 0.452366 | 0.476582 | 0.653726 | 0.49186 | 0.327969 | 0.349491 | 0.169202 | 0.512607 | 0.180858 | 0.465165 | 0.233652 | 0.220621 | 0.483978 | 0.308253 | 0.518405 | 0.478972 | 0.214513 | -0.00656 | 0.516887 | 0.441652 | 0.320401 | 0.359461 | 0.247405 | 0.357964 |
| TCGA-AA-3819-01A-01R-0905-07 | 0.080366 | 0.380867 | 0.398357 | 0.367652 | 0.4461 | 0.546679 | 0.635185 | 0.46715 | 0.292786 | 0.269716 | 0.138925 | 0.481618 | 0.127063 | 0.46375 | 0.193374 | 0.200869 | 0.417724 | 0.27567 | 0.55406 | 0.405766 | 0.206663 | 0.101414 | 0.505578 | 0.347791 | 0.297669 | 0.324048 | 0.267721 | 0.299653 |
| TCGA-AA-3861-01A-01R-1022-07 | 0.218874 | 0.481177 | 0.513257 | 0.35976 | 0.489163 | 0.568174 | 0.63442 | 0.491969 | 0.29511 | 0.345814 | 0.130738 | 0.493539 | 0.23695 | 0.467877 | 0.210908 | 0.206734 | 0.475891 | 0.28293 | 0.568569 | 0.395345 | 0.221955 | 0.05147 | 0.490302 | 0.327946 | 0.311539 | 0.339946 | 0.335979 | 0.370277 |
| TCGA-D5-6926-01A-11R-1928-07 | 0.069348 | 0.407427 | 0.431449 | 0.414891 | 0.456943 | 0.564412 | 0.64438 | 0.537962 | 0.356164 | 0.35651 | 0.121199 | 0.516308 | 0.169851 | 0.507029 | 0.251733 | 0.193484 | 0.533761 | 0.295328 | 0.534768 | 0.49499 | 0.287479 | 0.108636 | 0.575612 | 0.457857 | 0.338579 | 0.341723 | 0.234788 | 0.254796 |
| TCGA-AA-3531-01A-01R-0821-07 | -0.09401 | 0.322566 | 0.397254 | 0.326365 | 0.444191 | 0.576424 | 0.604043 | 0.441721 | 0.239011 | 0.260087 | 0.051401 | 0.454131 | -0.01087 | 0.444151 | 0.127683 | 0.100197 | 0.38945 | 0.282854 | 0.547765 | 0.376045 | 0.221935 | -0.01039 | 0.496349 | 0.338745 | 0.262079 | 0.272821 | 0.220886 | 0.197634 |
| TCGA-AA-3685-01A-02R-A32Z-07 | 0.155926 | 0.481156 | 0.55104 | 0.407567 | 0.477401 | 0.55612 | 0.63909 | 0.497453 | 0.335266 | 0.367237 | 0.137923 | 0.537879 | 0.22377 | 0.481084 | 0.280129 | 0.263302 | 0.530406 | 0.272684 | 0.562477 | 0.443311 | 0.279602 | 0.128985 | 0.50569 | 0.430572 | 0.349547 | 0.378788 | 0.30155 | 0.31787 |
| TCGA-AA-3712-01A-21R-1723-07 | -0.04617 | 0.460311 | 0.435204 | 0.379486 | 0.463374 | 0.542505 | 0.641966 | 0.502826 | 0.337932 | 0.334782 | 0.108196 | 0.507765 | 0.092472 | 0.482674 | 0.191693 | 0.16979 | 0.449898 | 0.242322 | 0.51671 | 0.449919 | 0.271098 | 0.077218 | 0.496018 | 0.398087 | 0.301257 | 0.329314 | 0.245116 | 0.289487 |
| TCGA-AA-3837-01A-01R-0905-07 | 0.103226 | 0.398327 | 0.433466 | 0.317966 | 0.441604 | 0.603265 | 0.647349 | 0.483135 | 0.285974 | 0.297128 | 0.073587 | 0.473232 | 0.167897 | 0.432799 | 0.193007 | 0.222318 | 0.390949 | 0.221815 | 0.553194 | 0.424984 | 0.205476 | -0.02632 | 0.506425 | 0.342903 | 0.260934 | 0.31881 | 0.290432 | 0.256445 |
| TCGA-AA-3664-01A-01R-0905-07 | -0.06546 | 0.468652 | 0.438603 | 0.38445 | 0.452163 | 0.537635 | 0.650529 | 0.483921 | 0.311207 | 0.339586 | 0.152564 | 0.454239 | 0.125451 | 0.482095 | 0.180844 | 0.114468 | 0.4445 | 0.300187 | 0.51788 | 0.416005 | 0.224648 | 0.09999 | 0.487251 | 0.332064 | 0.259978 | 0.318398 | 0.264746 | 0.359032 |
| TCGA-D5-6929-01A-31R-1928-07 | 0.164503 | 0.418566 | 0.47652 | 0.33839 | 0.468738 | 0.565581 | 0.657512 | 0.51357 | 0.31249 | 0.346723 | 0.109075 | 0.512218 | 0.188455 | 0.462364 | 0.215163 | 0.171765 | 0.49098 | 0.305379 | 0.520112 | 0.455122 | 0.242861 | 0.018414 | 0.551553 | 0.376437 | 0.30789 | 0.351565 | 0.222191 | 0.265804 |
| TCGA-A6-6648-01A-11R-1774-07 | 0.036295 | 0.446173 | 0.402179 | 0.34137 | 0.447157 | 0.499737 | 0.589832 | 0.466887 | 0.32633 | 0.266624 | 0.131123 | 0.454699 | 0.123602 | 0.467188 | 0.15879 | 0.217208 | 0.361423 | 0.339269 | 0.533179 | 0.42806 | 0.233543 | 0.042276 | 0.473981 | 0.307314 | 0.281141 | 0.304611 | 0.243784 | 0.306565 |
| TCGA-F4-6569-01A-11R-1774-07 | 0.147961 | 0.424295 | 0.491683 | 0.405189 | 0.452398 | 0.570594 | 0.681622 | 0.5717 | 0.39915 | 0.399631 | 0.147743 | 0.558094 | 0.234105 | 0.501693 | 0.328392 | 0.353084 | 0.568216 | 0.291046 | 0.575137 | 0.511418 | 0.327062 | 0.099446 | 0.538058 | 0.505932 | 0.384768 | 0.408145 | 0.226083 | 0.315558 |
| TCGA-AA-3966-01A-01R-1113-07 | 0.104392 | 0.5197 | 0.578546 | 0.483281 | 0.447733 | 0.655168 | 0.719407 | 0.58252 | 0.373144 | 0.439101 | 0.221753 | 0.547671 | 0.23725 | 0.546105 | 0.371909 | 0.321914 | 0.661999 | 0.323365 | 0.566853 | 0.499926 | 0.328189 | 0.274168 | 0.596605 | 0.523419 | 0.396998 | 0.40015 | 0.332586 | 0.39066 |
| TCGA-AZ-4684-01A-01R-1410-07 | 0.13254 | 0.447932 | 0.509626 | 0.427467 | 0.456895 | 0.572457 | 0.654682 | 0.504243 | 0.330161 | 0.320423 | 0.205812 | 0.502315 | 0.196206 | 0.530471 | 0.244605 | 0.260361 | 0.506279 | 0.293608 | 0.586064 | 0.453483 | 0.290359 | 0.166283 | 0.540758 | 0.401389 | 0.363051 | 0.383024 | 0.313667 | 0.309544 |
| TCGA-3L-AA1B-01A-11R-A37K-07 | 0.146371 | 0.346038 | 0.432198 | 0.316884 | 0.459401 | 0.55314 | 0.66692 | 0.476058 | 0.306897 | 0.322302 | 0.131937 | 0.448531 | 0.20151 | 0.444417 | 0.213212 | 0.17312 | 0.411708 | 0.278147 | 0.53803 | 0.442553 | 0.228767 | 0.017079 | 0.534567 | 0.343047 | 0.286063 | 0.317687 | 0.258197 | 0.301789 |
| TCGA-AA-A00A-01A-01R-A002-07 | 0.05014 | 0.432853 | 0.514291 | 0.400507 | 0.466678 | 0.558514 | 0.661264 | 0.504521 | 0.309176 | 0.41345 | 0.114937 | 0.50262 | 0.171382 | 0.442692 | 0.211162 | 0.149287 | 0.482499 | 0.26938 | 0.552722 | 0.442289 | 0.317234 | 0.05296 | 0.512486 | 0.356945 | 0.363907 | 0.363682 | 0.344208 | 0.272247 |
| TCGA-AA-3950-01A-02R-1022-07 | 0.010196 | 0.512749 | 0.575866 | 0.470935 | 0.470624 | 0.550299 | 0.67528 | 0.595354 | 0.341979 | 0.411846 | 0.134622 | 0.563474 | 0.188782 | 0.512776 | 0.355828 | 0.306634 | 0.651834 | 0.264985 | 0.576719 | 0.496631 | 0.317373 | 0.30375 | 0.592317 | 0.553827 | 0.408269 | 0.403776 | 0.24793 | 0.410991 |
| TCGA-DM-A285-01A-11R-A16W-07 | -0.16454 | 0.345179 | 0.308526 | 0.307925 | 0.432994 | 0.537398 | 0.618962 | 0.464367 | 0.287165 | 0.199413 | 0.068538 | 0.453499 | -0.03134 | 0.464144 | 0.140626 | 0.106216 | 0.327497 | 0.265495 | 0.486897 | 0.408569 | 0.212302 | -0.13258 | 0.492782 | 0.28287 | 0.240869 | 0.275563 | 0.218145 | 0.250368 |
| TCGA-CM-5349-01A-21R-1723-07 | 0.121553 | 0.433615 | 0.393525 | 0.399285 | 0.466144 | 0.555849 | 0.660145 | 0.511945 | 0.362383 | 0.313883 | 0.088745 | 0.49903 | 0.232109 | 0.495254 | 0.275769 | 0.201988 | 0.495816 | 0.279728 | 0.554104 | 0.460712 | 0.25133 | -0.00332 | 0.549566 | 0.444446 | 0.309183 | 0.339828 | 0.259223 | 0.291242 |
| TCGA-AA-A00J-01A-02R-A002-07 | -0.04569 | 0.443403 | 0.428369 | 0.352973 | 0.439869 | 0.555487 | 0.664899 | 0.524279 | 0.375557 | 0.368977 | 0.127991 | 0.46492 | 0.095286 | 0.517738 | 0.213304 | 0.103402 | 0.523216 | 0.258834 | 0.526562 | 0.465899 | 0.261881 | 0.103853 | 0.539047 | 0.359916 | 0.284959 | 0.301568 | 0.265083 | 0.397745 |
| TCGA-AY-6196-01A-11R-1723-07 | 0.397142 | 0.483332 | 0.628535 | 0.458058 | 0.446649 | 0.596341 | 0.748854 | 0.604352 | 0.413079 | 0.538491 | 0.231259 | 0.544469 | 0.43089 | 0.55032 | 0.406907 | 0.378031 | 0.722434 | 0.341988 | 0.598294 | 0.529704 | 0.359002 | 0.096612 | 0.597585 | 0.592896 | 0.467711 | 0.462393 | 0.261847 | 0.404848 |
| TCGA-AA-3534-01A-01R-0821-07 | 0.024467 | 0.422473 | 0.403481 | 0.358041 | 0.448606 | 0.528899 | 0.645311 | 0.478974 | 0.310746 | 0.28082 | 0.088459 | 0.48548 | 0.100817 | 0.4728 | 0.232003 | 0.226179 | 0.410862 | 0.321145 | 0.520734 | 0.426305 | 0.232234 | 0.023973 | 0.497364 | 0.366359 | 0.309551 | 0.327189 | 0.261293 | 0.342395 |
| TCGA-G4-6302-01A-11R-1723-07 | 0.078146 | 0.376742 | 0.443912 | 0.408236 | 0.448591 | 0.581991 | 0.68906 | 0.546102 | 0.411382 | 0.444794 | 0.135369 | 0.502041 | 0.196997 | 0.523912 | 0.340075 | 0.299767 | 0.55128 | 0.287625 | 0.547273 | 0.546853 | 0.342003 | 0.071971 | 0.553813 | 0.483815 | 0.385001 | 0.402039 | 0.203989 | 0.395285 |
| TCGA-AG-3901-01A-01R-1119-07 | 0.119934 | 0.418407 | 0.488047 | 0.421738 | 0.479939 | 0.5824 | 0.699769 | 0.567163 | 0.365513 | 0.382235 | 0.141349 | 0.538756 | 0.217001 | 0.535786 | 0.356027 | 0.320758 | 0.628388 | 0.304893 | 0.586887 | 0.491333 | 0.339457 | 0.151555 | 0.570323 | 0.549813 | 0.378743 | 0.398911 | 0.305132 | 0.332601 |
| TCGA-DC-6158-01A-11R-1660-07 | 0.03925 | 0.465839 | 0.475225 | 0.417383 | 0.461012 | 0.530929 | 0.660265 | 0.545757 | 0.374559 | 0.376171 | 0.172601 | 0.520316 | 0.220474 | 0.499591 | 0.279588 | 0.300769 | 0.55466 | 0.322723 | 0.572182 | 0.490742 | 0.306237 | 0.119637 | 0.57491 | 0.4945 | 0.381775 | 0.368977 | 0.283698 | 0.286614 |
| TCGA-F5-6571-01A-12R-1830-07 | 0.086058 | 0.398418 | 0.503177 | 0.394656 | 0.462649 | 0.556573 | 0.677215 | 0.507138 | 0.346037 | 0.401494 | 0.205011 | 0.48381 | 0.215324 | 0.498233 | 0.282994 | 0.270216 | 0.540435 | 0.301307 | 0.564206 | 0.501142 | 0.293405 | 0.050091 | 0.55481 | 0.470265 | 0.360737 | 0.367051 | 0.250832 | 0.286912 |
| TCGA-AG-3582-01A-01R-0821-07 | 0.03008 | 0.426126 | 0.438102 | 0.371833 | 0.456156 | 0.543439 | 0.627645 | 0.494349 | 0.321494 | 0.299534 | 0.086887 | 0.507585 | 0.140895 | 0.486059 | 0.216213 | 0.169562 | 0.494551 | 0.288562 | 0.541241 | 0.449962 | 0.267231 | 0.096449 | 0.526291 | 0.405902 | 0.319516 | 0.345433 | 0.290088 | 0.240422 |
| TCGA-EI-6508-01A-11R-1736-07 | 0.060304 | 0.423666 | 0.412273 | 0.310408 | 0.428789 | 0.539718 | 0.602876 | 0.437535 | 0.317368 | 0.264901 | 0.07418 | 0.484487 | 0.101462 | 0.4772 | 0.162551 | 0.17886 | 0.357512 | 0.312099 | 0.536318 | 0.41686 | 0.21063 | 0.062118 | 0.516559 | 0.321586 | 0.315046 | 0.307867 | 0.263191 | 0.296162 |
| TCGA-CL-5917-01A-11R-1660-07 | -0.15143 | 0.364683 | 0.332166 | 0.329676 | 0.448866 | 0.512241 | 0.622615 | 0.487097 | 0.292373 | 0.200733 | 0.124344 | 0.446072 | -0.00552 | 0.482722 | 0.183155 | 0.155239 | 0.342031 | 0.268548 | 0.547782 | 0.412832 | 0.220783 | 0.076003 | 0.528131 | 0.335322 | 0.245429 | 0.293017 | 0.200176 | 0.277958 |
| TCGA-AF-2687-01A-02R-1736-07 | 0.080486 | 0.479657 | 0.478464 | 0.431094 | 0.454117 | 0.506687 | 0.68035 | 0.574787 | 0.416336 | 0.412852 | 0.145051 | 0.556859 | 0.209003 | 0.520112 | 0.322513 | 0.372463 | 0.600581 | 0.322155 | 0.51717 | 0.496421 | 0.332345 | 0.117289 | 0.58962 | 0.568433 | 0.384184 | 0.3724 | 0.151969 | 0.298918 |
| TCGA-EF-5831-01A-01R-1660-07 | 0.007504 | 0.43261 | 0.421292 | 0.372761 | 0.465601 | 0.588363 | 0.638781 | 0.502414 | 0.285829 | 0.304329 | 0.064728 | 0.486179 | 0.159213 | 0.475326 | 0.208263 | 0.152757 | 0.449137 | 0.322745 | 0.520967 | 0.453854 | 0.280094 | -0.13176 | 0.516884 | 0.399635 | 0.293386 | 0.323373 | 0.251781 | 0.292843 |
| TCGA-DC-6157-01A-11R-1660-07 | 0.008255 | 0.406194 | 0.409837 | 0.323563 | 0.439447 | 0.526618 | 0.636746 | 0.483497 | 0.333989 | 0.269074 | 0.118853 | 0.455933 | 0.106849 | 0.443676 | 0.165694 | 0.16947 | 0.352028 | 0.311358 | 0.522624 | 0.433387 | 0.22282 | -0.07063 | 0.498836 | 0.32647 | 0.253196 | 0.318277 | 0.263202 | 0.287901 |
| TCGA-AG-A00C-01A-01R-A002-07 | 0.030457 | 0.393673 | 0.452271 | 0.323121 | 0.426611 | 0.535612 | 0.613168 | 0.420384 | 0.25612 | 0.276691 | 0.053819 | 0.41857 | 0.088224 | 0.403324 | 0.103491 | 0.080853 | 0.333421 | 0.248942 | 0.552006 | 0.380542 | 0.169183 | 0.086257 | 0.479838 | 0.225025 | 0.268479 | 0.29374 | 0.256975 | 0.23839 |
| TCGA-AF-A56N-01A-12R-A39D-07 | -0.05311 | 0.420899 | 0.421173 | 0.352345 | 0.43343 | 0.514592 | 0.637694 | 0.465108 | 0.307682 | 0.257485 | 0.116766 | 0.443352 | 0.054912 | 0.435722 | 0.167596 | 0.176025 | 0.396419 | 0.271723 | 0.531244 | 0.438098 | 0.228418 | 0.002163 | 0.528876 | 0.342753 | 0.278346 | 0.318145 | 0.298399 | 0.305757 |
| TCGA-DY-A1DE-01A-11R-A155-07 | -0.00808 | 0.435391 | 0.439392 | 0.375126 | 0.477271 | 0.552518 | 0.6356 | 0.486532 | 0.316144 | 0.326628 | 0.093068 | 0.489008 | 0.097438 | 0.449659 | 0.196464 | 0.155771 | 0.435735 | 0.248626 | 0.503734 | 0.439728 | 0.248374 | -0.07226 | 0.487797 | 0.376998 | 0.29665 | 0.324568 | 0.235779 | 0.275433 |
| TCGA-AG-A01L-01A-01R-A002-07 | -0.023 | 0.340252 | 0.404153 | 0.296549 | 0.456292 | 0.578827 | 0.608719 | 0.440433 | 0.262167 | 0.254616 | 0.048758 | 0.435515 | 0.078977 | 0.408564 | 0.13341 | 0.102959 | 0.367107 | 0.291704 | 0.549991 | 0.388026 | 0.201789 | -0.03572 | 0.460875 | 0.244365 | 0.29159 | 0.289004 | 0.274721 | 0.20092 |
| TCGA-AF-2693-01A-02R-1736-07 | 0.131425 | 0.460834 | 0.464725 | 0.403539 | 0.466714 | 0.554408 | 0.633018 | 0.517475 | 0.326445 | 0.328898 | 0.197326 | 0.491717 | 0.193171 | 0.473977 | 0.252923 | 0.279326 | 0.474839 | 0.295584 | 0.542029 | 0.459226 | 0.273296 | 0.151693 | 0.527059 | 0.366517 | 0.349419 | 0.357213 | 0.301383 | 0.336185 |
| TCGA-DC-6681-01A-11R-A32Z-07 | 0.062947 | 0.388457 | 0.423801 | 0.338563 | 0.448582 | 0.552726 | 0.628931 | 0.496175 | 0.330014 | 0.324138 | 0.095971 | 0.469093 | 0.115294 | 0.471768 | 0.212298 | 0.193444 | 0.415731 | 0.28741 | 0.574815 | 0.464614 | 0.245333 | 0.054674 | 0.509233 | 0.339302 | 0.306586 | 0.361541 | 0.302105 | 0.30984 |
| TCGA-AH-6903-01A-11R-1928-07 | -0.0027 | 0.390018 | 0.381687 | 0.323264 | 0.444782 | 0.498213 | 0.619794 | 0.451505 | 0.325979 | 0.268266 | 0.123122 | 0.447189 | 0.089266 | 0.465556 | 0.156423 | 0.120747 | 0.34253 | 0.305367 | 0.562381 | 0.40866 | 0.191774 | 0.082384 | 0.479978 | 0.277925 | 0.246438 | 0.292142 | 0.308086 | 0.305718 |
| TCGA-AG-A025-01A-01R-A00A-07 | 0.001694 | 0.386725 | 0.436797 | 0.33034 | 0.431599 | 0.57308 | 0.615723 | 0.435352 | 0.270686 | 0.243872 | 0.063029 | 0.450069 | 0.068871 | 0.465291 | 0.166095 | 0.185056 | 0.392841 | 0.227115 | 0.551519 | 0.421568 | 0.220433 | 0.025285 | 0.506827 | 0.285149 | 0.297034 | 0.318948 | 0.284467 | 0.276586 |
| TCGA-AG-3902-01A-01R-A32Z-07 | 0.121981 | 0.496374 | 0.55439 | 0.438366 | 0.458456 | 0.576048 | 0.682311 | 0.52196 | 0.322832 | 0.459106 | 0.189784 | 0.530972 | 0.226184 | 0.50464 | 0.277878 | 0.248569 | 0.537121 | 0.310749 | 0.577265 | 0.440681 | 0.280123 | 0.14669 | 0.520824 | 0.429073 | 0.372824 | 0.367685 | 0.322849 | 0.325895 |
| TCGA-AG-3893-01A-01R-1119-07 | -0.10411 | 0.34061 | 0.414416 | 0.368311 | 0.445708 | 0.581087 | 0.637874 | 0.501576 | 0.307292 | 0.310276 | 0.101943 | 0.499396 | 0.062883 | 0.484103 | 0.218774 | 0.197128 | 0.471519 | 0.304867 | 0.558798 | 0.439637 | 0.283991 | 0.058188 | 0.535547 | 0.373138 | 0.339266 | 0.333431 | 0.24747 | 0.226553 |
| TCGA-F5-6812-01A-11R-1830-07 | 0.169524 | 0.380705 | 0.484677 | 0.358009 | 0.470319 | 0.52789 | 0.668618 | 0.500417 | 0.380994 | 0.385953 | 0.118679 | 0.492279 | 0.262012 | 0.456617 | 0.263266 | 0.201144 | 0.523055 | 0.257183 | 0.540933 | 0.50907 | 0.277938 | -0.0146 | 0.547104 | 0.383688 | 0.362961 | 0.35608 | 0.260551 | 0.293149 |
| TCGA-AG-3881-01A-01R-0905-07 | 0.058696 | 0.388914 | 0.495458 | 0.419348 | 0.459371 | 0.655848 | 0.656656 | 0.564171 | 0.316591 | 0.404073 | 0.129407 | 0.52151 | 0.139714 | 0.530791 | 0.363002 | 0.313901 | 0.62117 | 0.201432 | 0.56012 | 0.472651 | 0.319684 | 0.184636 | 0.551983 | 0.499281 | 0.40903 | 0.380548 | 0.303852 | 0.296471 |
| TCGA-AG-A014-01A-02R-A002-07 | -0.20251 | 0.317465 | 0.322661 | 0.282079 | 0.417765 | 0.55648 | 0.609437 | 0.467166 | 0.237435 | 0.226529 | 0.037191 | 0.43086 | -0.04628 | 0.464493 | 0.123855 | 0.083727 | 0.337394 | 0.273909 | 0.570237 | 0.366828 | 0.221873 | 0.091182 | 0.484468 | 0.289666 | 0.27132 | 0.265408 | 0.22158 | 0.2219 |
| TCGA-AG-3580-01A-01R-0821-07 | 0.066335 | 0.486767 | 0.520415 | 0.413375 | 0.457569 | 0.543339 | 0.633174 | 0.483473 | 0.325801 | 0.342067 | 0.157854 | 0.494504 | 0.144155 | 0.473682 | 0.232348 | 0.16572 | 0.478516 | 0.249784 | 0.548554 | 0.413437 | 0.221787 | 0.188056 | 0.524595 | 0.382738 | 0.30769 | 0.349375 | 0.337804 | 0.324135 |
| TCGA-EI-6884-01A-11R-1928-07 | 0.17431 | 0.447553 | 0.478005 | 0.40715 | 0.475789 | 0.523004 | 0.691987 | 0.508806 | 0.370925 | 0.369072 | 0.196024 | 0.500233 | 0.219343 | 0.520032 | 0.255583 | 0.252236 | 0.516249 | 0.310333 | 0.548414 | 0.484019 | 0.284976 | 0.146833 | 0.534423 | 0.406608 | 0.352092 | 0.364622 | 0.296566 | 0.315856 |
| TCGA-AF-6136-01A-11R-1830-07 | 0.092482 | 0.428078 | 0.412667 | 0.35296 | 0.430881 | 0.531864 | 0.637709 | 0.478045 | 0.354735 | 0.301209 | 0.12429 | 0.432109 | 0.169641 | 0.461708 | 0.192719 | 0.165858 | 0.420314 | 0.289832 | 0.561079 | 0.411841 | 0.189901 | 0.102856 | 0.492867 | 0.278805 | 0.286049 | 0.283321 | 0.242022 | 0.292262 |
| TCGA-AF-3913-01A-02R-1119-07 | -0.07304 | 0.366456 | 0.368129 | 0.368374 | 0.432845 | 0.553858 | 0.624929 | 0.490662 | 0.319176 | 0.233355 | 0.099653 | 0.472025 | 0.041858 | 0.46128 | 0.19252 | 0.147898 | 0.428167 | 0.254984 | 0.568075 | 0.426004 | 0.234737 | 0.064133 | 0.517219 | 0.34685 | 0.292176 | 0.303505 | 0.25847 | 0.231979 |
| TCGA-AA-3663-01A-01R-1723-07 | -0.03578 | 0.488811 | 0.438496 | 0.388483 | 0.455383 | 0.533108 | 0.644945 | 0.521895 | 0.321027 | 0.360295 | 0.156332 | 0.535983 | 0.100782 | 0.459591 | 0.26051 | 0.221425 | 0.5103 | 0.268319 | 0.529256 | 0.418189 | 0.255559 | 0.136921 | 0.518233 | 0.376512 | 0.315599 | 0.319475 | 0.262867 | 0.354602 |
| TCGA-AA-3549-01A-02R-0821-07 | 0.089254 | 0.396595 | 0.472509 | 0.403926 | 0.488984 | 0.575683 | 0.645225 | 0.495077 | 0.310144 | 0.295405 | 0.192583 | 0.48733 | 0.156667 | 0.454229 | 0.195715 | 0.257871 | 0.476127 | 0.291417 | 0.559671 | 0.432457 | 0.278361 | 0.123567 | 0.530956 | 0.392597 | 0.315045 | 0.342835 | 0.313739 | 0.266827 |
| TCGA-AA-3561-01A-01R-0821-07 | -0.0528 | 0.447378 | 0.439711 | 0.342138 | 0.458178 | 0.538512 | 0.611394 | 0.473764 | 0.300398 | 0.245734 | 0.102941 | 0.481528 | 0.053146 | 0.464841 | 0.214088 | 0.127264 | 0.473398 | 0.231728 | 0.545688 | 0.404862 | 0.252111 | 0.144729 | 0.493629 | 0.336022 | 0.293004 | 0.326903 | 0.276856 | 0.317633 |
| TCGA-G4-6626-01A-11R-1774-07 | -0.07449 | 0.431468 | 0.415563 | 0.310961 | 0.421524 | 0.520343 | 0.570813 | 0.436871 | 0.267562 | 0.229984 | 0.040794 | 0.445351 | 0.021221 | 0.404144 | 0.076641 | 0.091485 | 0.305975 | 0.313461 | 0.507894 | 0.374724 | 0.23708 | -0.08439 | 0.468411 | 0.252064 | 0.247524 | 0.270079 | 0.278585 | 0.2184 |
| TCGA-AA-3821-01A-01R-1022-07 | -0.11733 | 0.429654 | 0.459303 | 0.358631 | 0.453951 | 0.58672 | 0.671214 | 0.544368 | 0.31598 | 0.35799 | 0.123747 | 0.502891 | 0.104944 | 0.485972 | 0.229212 | 0.142803 | 0.51583 | 0.242991 | 0.505296 | 0.425965 | 0.271716 | 0.096648 | 0.529584 | 0.410865 | 0.320657 | 0.30562 | 0.21837 | 0.341809 |
| TCGA-CK-4952-01A-01R-1723-07 | -0.0809 | 0.473051 | 0.395161 | 0.38419 | 0.419733 | 0.587483 | 0.627155 | 0.509045 | 0.332388 | 0.341119 | 0.178578 | 0.481775 | 0.06039 | 0.489178 | 0.252884 | 0.153667 | 0.48894 | 0.27471 | 0.530628 | 0.408784 | 0.246039 | 0.201521 | 0.531661 | 0.332389 | 0.265865 | 0.281599 | 0.252031 | 0.42091 |
| TCGA-A6-2681-01A-01R-1410-07 | 0.089815 | 0.360782 | 0.46254 | 0.419087 | 0.480093 | 0.612009 | 0.666346 | 0.558867 | 0.324148 | 0.357772 | 0.144201 | 0.499579 | 0.188268 | 0.510401 | 0.273524 | 0.295323 | 0.548261 | 0.234076 | 0.571164 | 0.461921 | 0.301952 | 0.09183 | 0.520857 | 0.447769 | 0.358497 | 0.358433 | 0.314051 | 0.272927 |
| TCGA-AY-6197-01A-11R-1723-07 | -9.57E-05 | 0.483341 | 0.437097 | 0.379555 | 0.445636 | 0.577635 | 0.64673 | 0.501465 | 0.306617 | 0.360729 | 0.182636 | 0.463989 | 0.112614 | 0.502723 | 0.202937 | 0.285784 | 0.440636 | 0.234865 | 0.564652 | 0.42957 | 0.27344 | 0.190036 | 0.487431 | 0.335725 | 0.289174 | 0.314948 | 0.323939 | 0.340925 |
| TCGA-AY-6386-01A-21R-1723-07 | 0.067968 | 0.46614 | 0.459649 | 0.427761 | 0.454944 | 0.565429 | 0.643687 | 0.509353 | 0.270733 | 0.33339 | 0.10342 | 0.498468 | 0.168161 | 0.471722 | 0.210279 | 0.16554 | 0.459716 | 0.324356 | 0.566218 | 0.422975 | 0.252349 | 0.147789 | 0.529575 | 0.383106 | 0.327219 | 0.334809 | 0.328208 | 0.32904 |
| TCGA-AU-3779-01A-01R-1723-07 | 0.115482 | 0.457258 | 0.481907 | 0.417336 | 0.476851 | 0.576431 | 0.666997 | 0.499862 | 0.30338 | 0.385756 | 0.157755 | 0.481479 | 0.215712 | 0.496443 | 0.242505 | 0.190712 | 0.50606 | 0.292959 | 0.557515 | 0.458813 | 0.259845 | 0.121187 | 0.548589 | 0.414786 | 0.325723 | 0.369848 | 0.267705 | 0.310662 |
| TCGA-CM-4744-01A-01R-A32Z-07 | 0.085307 | 0.490163 | 0.570232 | 0.392475 | 0.447838 | 0.548507 | 0.628639 | 0.464065 | 0.29946 | 0.3909 | 0.120171 | 0.492372 | 0.169125 | 0.449351 | 0.186907 | 0.145759 | 0.443236 | 0.254123 | 0.556212 | 0.411163 | 0.292308 | 0.093244 | 0.471312 | 0.340648 | 0.329357 | 0.360543 | 0.285047 | 0.347439 |
| TCGA-AD-A5EJ-01A-11R-A28H-07 | -0.04499 | 0.445087 | 0.471541 | 0.369177 | 0.425055 | 0.573687 | 0.658349 | 0.53543 | 0.302389 | 0.310769 | 0.096508 | 0.477623 | 0.119797 | 0.456395 | 0.221683 | 0.134714 | 0.465048 | 0.27284 | 0.53285 | 0.442451 | 0.269789 | 0.155358 | 0.515767 | 0.323087 | 0.311745 | 0.325383 | 0.245182 | 0.365602 |
| TCGA-AG-A02G-01A-01R-A00A-07 | -0.00383 | 0.353839 | 0.426574 | 0.31385 | 0.456136 | 0.551007 | 0.634158 | 0.434215 | 0.283468 | 0.273925 | 0.086015 | 0.474856 | 0.085895 | 0.443897 | 0.197803 | 0.201194 | 0.377713 | 0.267995 | 0.557048 | 0.36982 | 0.229206 | -0.0154 | 0.473982 | 0.292937 | 0.27004 | 0.287216 | 0.233433 | 0.254081 |
| TCGA-DC-6154-01A-31R-1928-07 | -0.04775 | 0.318615 | 0.419163 | 0.34802 | 0.460688 | 0.555067 | 0.630186 | 0.464907 | 0.30375 | 0.294125 | 0.077617 | 0.469277 | 0.055498 | 0.46477 | 0.174383 | 0.166625 | 0.415229 | 0.275437 | 0.542085 | 0.441853 | 0.256572 | 0.036806 | 0.545986 | 0.352421 | 0.285279 | 0.305459 | 0.185594 | 0.192863 |
| TCGA-AG-3584-01A-01R-0821-07 | 0.077201 | 0.411512 | 0.472793 | 0.372326 | 0.444807 | 0.615713 | 0.63605 | 0.518048 | 0.30147 | 0.336538 | 0.107884 | 0.50642 | 0.150233 | 0.48357 | 0.208248 | 0.201612 | 0.471689 | 0.282253 | 0.549226 | 0.424605 | 0.269019 | 0.074035 | 0.496718 | 0.372853 | 0.345612 | 0.33725 | 0.28612 | 0.263246 |
| TCGA-AG-A032-01A-01R-A00A-07 | 0.040556 | 0.377181 | 0.42324 | 0.339618 | 0.445264 | 0.578086 | 0.644533 | 0.444292 | 0.275251 | 0.276028 | 0.125738 | 0.422179 | 0.091024 | 0.422135 | 0.161225 | 0.116658 | 0.370399 | 0.287839 | 0.550094 | 0.421335 | 0.220224 | -0.01161 | 0.495057 | 0.261835 | 0.271256 | 0.308842 | 0.28044 | 0.241939 |
| TCGA-G5-6641-01A-11R-A32Z-07 | -0.05733 | 0.295835 | 0.344063 | 0.291725 | 0.453172 | 0.521282 | 0.600242 | 0.416534 | 0.30356 | 0.216707 | 0.123277 | 0.412319 | -0.00491 | 0.469473 | 0.125885 | 0.072641 | 0.287092 | 0.262151 | 0.514464 | 0.334108 | 0.148936 | -0.03633 | 0.477787 | 0.1633 | 0.189302 | 0.253108 | 0.228708 | 0.248428 |
| TCGA-AG-3890-01A-01R-1119-07 | 0.192833 | 0.422695 | 0.517412 | 0.3589 | 0.447419 | 0.595468 | 0.667059 | 0.459254 | 0.27513 | 0.333168 | 0.159972 | 0.452104 | 0.223359 | 0.47901 | 0.198005 | 0.154051 | 0.482262 | 0.30743 | 0.552926 | 0.418396 | 0.213377 | 0.047629 | 0.531557 | 0.363556 | 0.333703 | 0.331553 | 0.303089 | 0.231145 |
| TCGA-AG-3742-01A-11R-1660-07 | -0.07761 | 0.389717 | 0.40936 | 0.32445 | 0.441408 | 0.501788 | 0.633883 | 0.495274 | 0.294601 | 0.213369 | 0.095236 | 0.470992 | 0.035566 | 0.465798 | 0.164014 | 0.172027 | 0.39945 | 0.278948 | 0.512727 | 0.429878 | 0.247305 | 0.088034 | 0.506436 | 0.377129 | 0.279823 | 0.302679 | 0.24571 | 0.277492 |
| TCGA-AG-A026-01A-01R-A00A-07 | -0.16519 | 0.311445 | 0.372854 | 0.279278 | 0.453853 | 0.549379 | 0.621016 | 0.476173 | 0.324277 | 0.223214 | 0.082006 | 0.447077 | -0.0082 | 0.432894 | 0.129029 | 0.095999 | 0.334693 | 0.247205 | 0.512373 | 0.453099 | 0.203769 | 0.010693 | 0.513562 | 0.2795 | 0.254379 | 0.283135 | 0.2197 | 0.222137 |
| TCGA-F5-6702-01A-11R-1830-07 | 0.056237 | 0.390754 | 0.422904 | 0.377546 | 0.467698 | 0.558046 | 0.690137 | 0.570942 | 0.376252 | 0.384952 | 0.165624 | 0.532888 | 0.212743 | 0.502592 | 0.276637 | 0.303934 | 0.537042 | 0.299576 | 0.546095 | 0.509447 | 0.340358 | -0.00131 | 0.582095 | 0.50229 | 0.363075 | 0.365264 | 0.248775 | 0.299586 |
| TCGA-G5-6572-01A-11R-1830-07 | -0.12929 | 0.328183 | 0.371856 | 0.31285 | 0.488041 | 0.538301 | 0.630874 | 0.517228 | 0.359173 | 0.281553 | 0.093196 | 0.438994 | 0.030069 | 0.431898 | 0.237673 | 0.261446 | 0.408516 | 0.311499 | 0.535405 | 0.440807 | 0.214971 | -0.05213 | 0.509461 | 0.26984 | 0.282075 | 0.307317 | 0.211644 | 0.245624 |
| TCGA-F5-6464-01A-11R-1736-07 | 0.121575 | 0.424738 | 0.50968 | 0.441003 | 0.438155 | 0.595158 | 0.700412 | 0.59548 | 0.388221 | 0.428709 | 0.153736 | 0.54258 | 0.266304 | 0.513274 | 0.346521 | 0.331557 | 0.605733 | 0.319688 | 0.576527 | 0.541146 | 0.331078 | 0.070349 | 0.549896 | 0.533459 | 0.428524 | 0.403946 | 0.249479 | 0.368037 |
| TCGA-EF-5830-01A-01R-1660-07 | -0.18536 | 0.361847 | 0.479382 | 0.321625 | 0.485758 | 0.55176 | 0.612431 | 0.442605 | 0.300129 | 0.25442 | 0.079046 | 0.48718 | 0.019327 | 0.464646 | 0.140036 | 0.158416 | 0.389221 | 0.256177 | 0.517803 | 0.394842 | 0.236205 | -0.02039 | 0.511211 | 0.307209 | 0.300682 | 0.312201 | 0.247025 | 0.323083 |
| TCGA-DC-6682-01A-11R-1830-07 | -0.00725 | 0.452903 | 0.429994 | 0.335862 | 0.439852 | 0.518994 | 0.585952 | 0.474934 | 0.34502 | 0.28385 | 0.048368 | 0.457117 | 0.101982 | 0.441018 | 0.12463 | 0.118253 | 0.338978 | 0.377871 | 0.474303 | 0.438767 | 0.204503 | -0.00607 | 0.496304 | 0.3003 | 0.306377 | 0.314764 | 0.230787 | 0.341123 |
| TCGA-AG-3727-01A-01R-0905-07 | -0.04918 | 0.265447 | 0.37659 | 0.332056 | 0.459473 | 0.622007 | 0.626388 | 0.493746 | 0.227676 | 0.282891 | 0.058513 | 0.456646 | 0.074847 | 0.465971 | 0.245544 | 0.158033 | 0.500211 | 0.219841 | 0.558146 | 0.427227 | 0.236414 | 0.106571 | 0.484388 | 0.385753 | 0.332802 | 0.326537 | 0.288053 | 0.260179 |
| TCGA-AG-A01Y-01A-41R-A083-07 | 0.103289 | 0.462356 | 0.451522 | 0.388267 | 0.443298 | 0.515706 | 0.620613 | 0.476475 | 0.290615 | 0.329763 | 0.048537 | 0.514606 | 0.181246 | 0.47537 | 0.238068 | 0.204351 | 0.483916 | 0.28319 | 0.517965 | 0.460978 | 0.274056 | -0.05966 | 0.512461 | 0.432521 | 0.344785 | 0.334487 | 0.243316 | 0.259492 |
| TCGA-AG-3598-01A-01R-0826-07 | 0.069131 | 0.479502 | 0.532137 | 0.388099 | 0.464281 | 0.538307 | 0.621347 | 0.483515 | 0.302085 | 0.332668 | 0.117823 | 0.511828 | 0.152806 | 0.495286 | 0.215927 | 0.127714 | 0.485119 | 0.201809 | 0.53435 | 0.423405 | 0.248675 | 0.111134 | 0.481543 | 0.366579 | 0.328072 | 0.33844 | 0.303431 | 0.291312 |
| TCGA-CM-4748-01A-01R-1410-07 | -0.00655 | 0.31461 | 0.360559 | 0.310117 | 0.437619 | 0.553793 | 0.658088 | 0.511552 | 0.252832 | 0.30386 | 0.054096 | 0.440463 | 0.061095 | 0.448035 | 0.210096 | 0.135654 | 0.506873 | 0.198251 | 0.532664 | 0.423864 | 0.259378 | 0.003074 | 0.511335 | 0.367839 | 0.310821 | 0.289147 | 0.346039 | 0.203189 |
| TCGA-AA-3517-01A-01R-0821-07 | -0.00423 | 0.394066 | 0.424029 | 0.3588 | 0.441464 | 0.59605 | 0.64949 | 0.490043 | 0.309389 | 0.336893 | 0.160302 | 0.469046 | 0.095395 | 0.456834 | 0.228363 | 0.205716 | 0.487271 | 0.239263 | 0.534483 | 0.440491 | 0.235609 | 0.171232 | 0.511958 | 0.38933 | 0.303194 | 0.326205 | 0.280573 | 0.26116 |
| TCGA-D5-5541-01A-01R-1653-07 | 0.042454 | 0.441662 | 0.481751 | 0.401566 | 0.455619 | 0.55305 | 0.640931 | 0.549997 | 0.366835 | 0.345236 | 0.113023 | 0.51668 | 0.180287 | 0.486427 | 0.260129 | 0.242852 | 0.51238 | 0.325834 | 0.541566 | 0.468794 | 0.279279 | 0.031664 | 0.53344 | 0.475306 | 0.347948 | 0.346822 | 0.250514 | 0.275966 |
| TCGA-A6-2686-01A-01R-A32Z-07 | 0.175254 | 0.508979 | 0.634788 | 0.432068 | 0.446169 | 0.607019 | 0.676412 | 0.569627 | 0.338345 | 0.430509 | 0.163682 | 0.549536 | 0.288488 | 0.53309 | 0.346842 | 0.320388 | 0.64056 | 0.266919 | 0.550265 | 0.477248 | 0.348119 | 0.135207 | 0.542308 | 0.509265 | 0.405239 | 0.382918 | 0.231523 | 0.317429 |
| TCGA-NH-A50U-01A-33R-A37K-07 | -0.19176 | 0.405045 | 0.335089 | 0.32489 | 0.44816 | 0.560923 | 0.631668 | 0.453588 | 0.273043 | 0.288825 | 0.047681 | 0.466872 | 0.038252 | 0.461053 | 0.1666 | 0.148896 | 0.387507 | 0.258571 | 0.53389 | 0.415211 | 0.207208 | -0.06535 | 0.51323 | 0.269376 | 0.285088 | 0.259173 | 0.176072 | 0.272491 |
| TCGA-AA-3543-01A-01R-0826-07 | 0.143479 | 0.495191 | 0.583336 | 0.441901 | 0.46563 | 0.602631 | 0.677395 | 0.530933 | 0.31156 | 0.475883 | 0.128058 | 0.50873 | 0.232558 | 0.522198 | 0.294798 | 0.204853 | 0.590312 | 0.26566 | 0.554453 | 0.465845 | 0.326764 | 0.173445 | 0.508215 | 0.415625 | 0.409819 | 0.381902 | 0.311686 | 0.346104 |
| TCGA-AA-3518-01A-02R-0826-07 | 0.097126 | 0.476294 | 0.527494 | 0.388747 | 0.439391 | 0.576404 | 0.66594 | 0.52616 | 0.305284 | 0.430726 | 0.110713 | 0.499721 | 0.163553 | 0.467429 | 0.233178 | 0.117517 | 0.552779 | 0.234487 | 0.554302 | 0.437311 | 0.278555 | 0.16393 | 0.530675 | 0.376269 | 0.324041 | 0.363045 | 0.293589 | 0.353399 |
| TCGA-AA-3984-01A-02R-1022-07 | 0.088172 | 0.561999 | 0.504908 | 0.383319 | 0.445406 | 0.555812 | 0.635282 | 0.500391 | 0.349548 | 0.376975 | 0.159961 | 0.491476 | 0.184328 | 0.487613 | 0.225144 | 0.233692 | 0.486644 | 0.30026 | 0.529893 | 0.462357 | 0.268122 | 0.094723 | 0.513606 | 0.385113 | 0.324962 | 0.375182 | 0.286921 | 0.321296 |
| TCGA-CM-4752-01A-01R-1410-07 | 0.116018 | 0.42369 | 0.531215 | 0.397089 | 0.460771 | 0.562764 | 0.640414 | 0.496435 | 0.338201 | 0.36979 | 0.128971 | 0.493778 | 0.200368 | 0.465048 | 0.262234 | 0.199614 | 0.514323 | 0.242512 | 0.545107 | 0.456105 | 0.258568 | 0.082785 | 0.544893 | 0.368779 | 0.334099 | 0.345298 | 0.320059 | 0.264884 |
| TCGA-AA-3684-01A-02R-0905-07 | 0.125558 | 0.340994 | 0.498215 | 0.411687 | 0.478736 | 0.648964 | 0.68104 | 0.564382 | 0.281266 | 0.405906 | 0.14419 | 0.519835 | 0.191187 | 0.546939 | 0.353059 | 0.253607 | 0.576531 | 0.254227 | 0.615324 | 0.478251 | 0.320258 | 0.205894 | 0.544752 | 0.489998 | 0.426558 | 0.383834 | 0.296517 | 0.302323 |
| TCGA-AA-3941-01A-01R-1022-07 | 0.048229 | 0.421472 | 0.415625 | 0.322257 | 0.467882 | 0.580624 | 0.648715 | 0.472579 | 0.281616 | 0.288193 | 0.119183 | 0.460983 | 0.139154 | 0.479988 | 0.17798 | 0.197711 | 0.402379 | 0.277478 | 0.597305 | 0.406072 | 0.212538 | -0.01226 | 0.5077 | 0.283207 | 0.298806 | 0.303886 | 0.247436 | 0.387012 |
| TCGA-A6-5660-01A-01R-1653-07 | -0.04483 | 0.391744 | 0.403155 | 0.358828 | 0.477339 | 0.505289 | 0.639186 | 0.523122 | 0.339418 | 0.304104 | 0.121436 | 0.490154 | 0.11242 | 0.491216 | 0.229832 | 0.22517 | 0.442072 | 0.320697 | 0.545751 | 0.447143 | 0.273738 | 0.036669 | 0.522725 | 0.357612 | 0.305457 | 0.32095 | 0.212528 | 0.267819 |
| TCGA-A6-6652-01A-11R-1774-07 | -0.20185 | 0.36217 | 0.339157 | 0.283707 | 0.475007 | 0.521861 | 0.635289 | 0.436761 | 0.311102 | 0.258022 | 0.062153 | 0.437102 | -0.01239 | 0.494829 | 0.123816 | 0.045572 | 0.315125 | 0.287493 | 0.526309 | 0.398071 | 0.205157 | -0.00998 | 0.490582 | 0.286313 | 0.233417 | 0.295425 | 0.231189 | 0.235079 |
| TCGA-CM-5868-01A-01R-1653-07 | -0.05258 | 0.403846 | 0.392275 | 0.352224 | 0.460595 | 0.52933 | 0.64006 | 0.490835 | 0.314969 | 0.259171 | 0.081399 | 0.499583 | 0.077214 | 0.47544 | 0.188641 | 0.125191 | 0.385202 | 0.274693 | 0.527816 | 0.438166 | 0.247836 | -0.03875 | 0.498031 | 0.307848 | 0.271354 | 0.316651 | 0.230109 | 0.255316 |
| TCGA-CM-6164-01A-11R-1653-07 | 0.004649 | 0.43172 | 0.449375 | 0.365555 | 0.457521 | 0.537088 | 0.635714 | 0.490863 | 0.315931 | 0.323957 | 0.085918 | 0.479861 | 0.118167 | 0.463274 | 0.188365 | 0.162396 | 0.405674 | 0.300157 | 0.528801 | 0.462596 | 0.244399 | 0.020929 | 0.544602 | 0.321312 | 0.296951 | 0.327862 | 0.193344 | 0.240277 |
| TCGA-AZ-6606-01A-11R-1839-07 | -0.00731 | 0.407941 | 0.38838 | 0.312236 | 0.465172 | 0.521278 | 0.632116 | 0.459253 | 0.304795 | 0.277495 | 0.125629 | 0.448839 | 0.116826 | 0.48837 | 0.145945 | 0.126153 | 0.358138 | 0.279785 | 0.526613 | 0.400652 | 0.22361 | 0.016295 | 0.458439 | 0.245454 | 0.270207 | 0.308449 | 0.325185 | 0.357949 |
| TCGA-DM-A1HA-01A-11R-A155-07 | -0.16362 | 0.334489 | 0.424622 | 0.28027 | 0.40956 | 0.586673 | 0.608159 | 0.446345 | 0.275141 | 0.253153 | -0.07875 | 0.409189 | 0.046888 | 0.41681 | 0.08329 | 0.000222 | 0.288206 | 0.221461 | 0.55268 | 0.406337 | 0.225819 | -0.00584 | 0.451827 | 0.188608 | 0.227449 | 0.239445 | 0.191881 | 0.232956 |
| TCGA-G4-6323-01A-11R-1723-07 | 0.456442 | 0.43632 | 0.510166 | 0.351491 | 0.456332 | 0.535636 | 0.66319 | 0.435419 | 0.336713 | 0.366014 | 0.113084 | 0.440312 | 0.397502 | 0.423519 | 0.195895 | 0.096113 | 0.465832 | 0.336514 | 0.556493 | 0.393459 | 0.185352 | -0.02834 | 0.486634 | 0.257702 | 0.307969 | 0.335271 | 0.339844 | 0.297171 |
| TCGA-AA-3542-01A-02R-1873-07 | -0.08688 | 0.280861 | 0.41046 | 0.346027 | 0.454399 | 0.57639 | 0.58817 | 0.436516 | 0.247577 | 0.231015 | -0.01642 | 0.450395 | 0.002124 | 0.476407 | 0.208213 | 0.201565 | 0.401748 | 0.241162 | 0.564565 | 0.364695 | 0.271688 | 0.048 | 0.51164 | 0.289816 | 0.326788 | 0.293974 | 0.256335 | 0.179246 |
| TCGA-D5-6924-01A-11R-1928-07 | 0.153752 | 0.489975 | 0.479711 | 0.379164 | 0.465565 | 0.54812 | 0.668694 | 0.566826 | 0.37434 | 0.372926 | 0.207331 | 0.529856 | 0.260071 | 0.485552 | 0.274612 | 0.329825 | 0.546709 | 0.312608 | 0.560084 | 0.48413 | 0.287488 | 0.149427 | 0.578612 | 0.49611 | 0.354521 | 0.381048 | 0.220305 | 0.30832 |
| TCGA-F4-6856-01A-11R-1928-07 | 0.074668 | 0.427237 | 0.488405 | 0.345318 | 0.452455 | 0.545714 | 0.661338 | 0.492242 | 0.342095 | 0.338704 | 0.090361 | 0.445579 | 0.121719 | 0.477613 | 0.178131 | 0.119469 | 0.477037 | 0.294202 | 0.550093 | 0.453091 | 0.2382 | 0.019582 | 0.500332 | 0.295738 | 0.298517 | 0.320088 | 0.280504 | 0.358389 |
| TCGA-QG-A5YV-01A-11R-A28H-07 | -0.00286 | 0.448137 | 0.419597 | 0.332175 | 0.451829 | 0.51452 | 0.624163 | 0.439977 | 0.3024 | 0.293683 | 0.087831 | 0.446076 | 0.086238 | 0.412491 | 0.146598 | 0.114813 | 0.40182 | 0.252801 | 0.54036 | 0.413723 | 0.193573 | 0.034246 | 0.465746 | 0.274267 | 0.270571 | 0.307114 | 0.245249 | 0.289994 |
| TCGA-AA-3688-01A-01R-0905-07 | 0.052229 | 0.411302 | 0.436718 | 0.361165 | 0.453866 | 0.554065 | 0.618107 | 0.468117 | 0.285588 | 0.294846 | 0.078513 | 0.479795 | 0.126033 | 0.472138 | 0.191177 | 0.167143 | 0.426791 | 0.280922 | 0.535261 | 0.420253 | 0.222906 | 0.115323 | 0.532418 | 0.329702 | 0.284643 | 0.315243 | 0.263433 | 0.265861 |
| TCGA-AA-3980-01A-02R-1022-07 | 0.057297 | 0.524953 | 0.57372 | 0.411068 | 0.471334 | 0.539179 | 0.634573 | 0.519731 | 0.310686 | 0.360783 | 0.134357 | 0.4893 | 0.198333 | 0.489473 | 0.228697 | 0.173432 | 0.50459 | 0.303364 | 0.554275 | 0.455509 | 0.284626 | 0.153977 | 0.49586 | 0.401271 | 0.372283 | 0.364323 | 0.277161 | 0.361841 |
| TCGA-AA-3525-01A-02R-0826-07 | 0.105335 | 0.432269 | 0.451284 | 0.389553 | 0.445978 | 0.578242 | 0.648439 | 0.546385 | 0.308418 | 0.394652 | 0.116035 | 0.523977 | 0.162988 | 0.501888 | 0.26413 | 0.245865 | 0.49363 | 0.267356 | 0.569039 | 0.399059 | 0.287417 | 0.164373 | 0.522824 | 0.343435 | 0.355312 | 0.338509 | 0.305198 | 0.354434 |
| TCGA-AA-3994-01A-01R-1113-07 | 0.00844 | 0.379037 | 0.436324 | 0.394248 | 0.481595 | 0.5707 | 0.694303 | 0.521728 | 0.343614 | 0.343655 | 0.089859 | 0.511296 | 0.140314 | 0.4893 | 0.25962 | 0.211691 | 0.500132 | 0.208279 | 0.556338 | 0.416334 | 0.254083 | 0.111722 | 0.533061 | 0.370458 | 0.319637 | 0.353582 | 0.233254 | 0.361719 |
| TCGA-AY-4070-01A-01R-1113-07 | 0.146298 | 0.381229 | 0.474089 | 0.343483 | 0.428996 | 0.555167 | 0.66365 | 0.459279 | 0.289073 | 0.302575 | 0.134131 | 0.478546 | 0.172812 | 0.510351 | 0.215419 | 0.167508 | 0.453649 | 0.284162 | 0.556055 | 0.421541 | 0.25774 | 0.013173 | 0.525907 | 0.348367 | 0.335566 | 0.325069 | 0.262149 | 0.299996 |
| TCGA-AA-3495-01A-01R-1410-07 | 0.136768 | 0.440169 | 0.505607 | 0.387002 | 0.459898 | 0.561038 | 0.636797 | 0.496679 | 0.337962 | 0.302868 | 0.129995 | 0.524044 | 0.178654 | 0.485043 | 0.225018 | 0.205004 | 0.476188 | 0.287223 | 0.539651 | 0.434751 | 0.25032 | 0.139392 | 0.51842 | 0.381821 | 0.319248 | 0.341636 | 0.321819 | 0.294348 |
| TCGA-D5-6531-01A-11R-1723-07 | 0.042697 | 0.453247 | 0.498401 | 0.40556 | 0.457998 | 0.589936 | 0.637806 | 0.533663 | 0.345897 | 0.353012 | 0.114582 | 0.477846 | 0.149852 | 0.446202 | 0.223287 | 0.221083 | 0.520212 | 0.268008 | 0.527257 | 0.474294 | 0.278412 | 0.071627 | 0.515443 | 0.412566 | 0.371109 | 0.351466 | 0.26383 | 0.320425 |
| TCGA-CA-5254-01A-21R-1839-07 | -0.00222 | 0.374672 | 0.425493 | 0.353237 | 0.43143 | 0.561935 | 0.640715 | 0.548825 | 0.278412 | 0.349612 | 0.051759 | 0.452014 | 0.13265 | 0.448053 | 0.224623 | 0.157096 | 0.441576 | 0.268795 | 0.524937 | 0.442914 | 0.221066 | 0.026281 | 0.492603 | 0.263578 | 0.282294 | 0.315459 | 0.248334 | 0.331617 |
| TCGA-QL-A97D-01A-12R-A41B-07 | 0.1568 | 0.523337 | 0.514653 | 0.402109 | 0.454615 | 0.540027 | 0.639291 | 0.432552 | 0.263095 | 0.342228 | 0.202953 | 0.438812 | 0.235221 | 0.43867 | 0.17873 | 0.159274 | 0.449044 | 0.300028 | 0.537443 | 0.427972 | 0.237827 | -0.01114 | 0.477541 | 0.30651 | 0.289979 | 0.324648 | 0.240953 | 0.292053 |
| TCGA-AA-A02R-01A-01R-A00A-07 | 0.124138 | 0.509987 | 0.587728 | 0.406977 | 0.443959 | 0.605201 | 0.6749 | 0.545751 | 0.314845 | 0.525955 | 0.044355 | 0.52287 | 0.274538 | 0.494256 | 0.29721 | 0.200249 | 0.626009 | 0.253829 | 0.562407 | 0.489086 | 0.333685 | 0.012636 | 0.493386 | 0.483227 | 0.412515 | 0.393245 | 0.217609 | 0.356554 |
| TCGA-CK-6751-01A-11R-1839-07 | 0.001969 | 0.480034 | 0.466817 | 0.357876 | 0.463859 | 0.556313 | 0.644015 | 0.502293 | 0.323225 | 0.314494 | 0.148451 | 0.49281 | 0.112806 | 0.467405 | 0.205223 | 0.215787 | 0.422282 | 0.256586 | 0.543816 | 0.455001 | 0.260045 | 0.065115 | 0.515113 | 0.344223 | 0.313804 | 0.345705 | 0.306112 | 0.331895 |
| TCGA-F4-6805-01A-11R-1839-07 | 0.144911 | 0.477253 | 0.468778 | 0.449096 | 0.464977 | 0.53843 | 0.67772 | 0.58245 | 0.37999 | 0.395867 | 0.196504 | 0.53925 | 0.227602 | 0.539113 | 0.319932 | 0.319735 | 0.567018 | 0.2857 | 0.570645 | 0.503071 | 0.311962 | 0.110896 | 0.568042 | 0.507877 | 0.377932 | 0.39703 | 0.311023 | 0.367202 |
| TCGA-AA-3696-01A-01R-0905-07 | -0.04369 | 0.361244 | 0.337553 | 0.282288 | 0.447464 | 0.580502 | 0.607584 | 0.467467 | 0.294343 | 0.2436 | 0.13148 | 0.454556 | 0.018567 | 0.463561 | 0.157859 | 0.160385 | 0.366047 | 0.207453 | 0.537152 | 0.407422 | 0.132111 | -0.06823 | 0.510909 | 0.258555 | 0.196076 | 0.255361 | 0.257027 | 0.265823 |
| TCGA-A6-5662-01A-01R-1653-07 | -0.12444 | 0.315616 | 0.333404 | 0.341436 | 0.464475 | 0.51017 | 0.622223 | 0.449718 | 0.349882 | 0.253409 | 0.077898 | 0.464404 | 0.041489 | 0.480225 | 0.17728 | 0.138851 | 0.385853 | 0.256624 | 0.515186 | 0.438476 | 0.221723 | -0.02027 | 0.502295 | 0.302459 | 0.276109 | 0.305259 | 0.246703 | 0.235911 |
| TCGA-AZ-6601-01A-11R-1774-07 | 0.225506 | 0.522756 | 0.549972 | 0.415483 | 0.453496 | 0.560891 | 0.695793 | 0.542091 | 0.357875 | 0.439614 | 0.097595 | 0.539006 | 0.309541 | 0.503942 | 0.318784 | 0.21393 | 0.600329 | 0.306754 | 0.596658 | 0.491347 | 0.333575 | 0.06427 | 0.512038 | 0.486383 | 0.385292 | 0.384388 | 0.261434 | 0.400495 |
| TCGA-AA-3556-01A-01R-0821-07 | 0.099651 | 0.421877 | 0.458342 | 0.401162 | 0.449525 | 0.505518 | 0.652042 | 0.546018 | 0.318445 | 0.351166 | 0.107849 | 0.507927 | 0.214696 | 0.461516 | 0.248412 | 0.188327 | 0.49554 | 0.304482 | 0.556625 | 0.449545 | 0.231156 | 0.191252 | 0.514436 | 0.368167 | 0.325783 | 0.349664 | 0.31505 | 0.341727 |
| TCGA-DM-A1D4-01A-21R-A155-07 | -0.1302 | 0.429004 | 0.459354 | 0.316058 | 0.44625 | 0.553533 | 0.623282 | 0.436407 | 0.281108 | 0.260805 | 0.045646 | 0.411281 | 0.011396 | 0.394189 | 0.123685 | 0.062334 | 0.331967 | 0.256713 | 0.52049 | 0.360477 | 0.203129 | -0.0092 | 0.453318 | 0.189066 | 0.232359 | 0.265638 | 0.295184 | 0.325363 |
| TCGA-RU-A8FL-01A-11R-A37K-07 | -0.15054 | 0.350157 | 0.358469 | 0.257978 | 0.428041 | 0.484269 | 0.565733 | 0.384665 | 0.229018 | 0.152406 | 0.09695 | 0.415187 | -0.03657 | 0.457343 | 0.073673 | 0.040495 | 0.235752 | 0.257994 | 0.495868 | 0.315212 | 0.144968 | -0.03047 | 0.487472 | 0.165103 | 0.223906 | 0.220051 | 0.266499 | 0.217604 |
| TCGA-AA-3975-01A-01R-1022-07 | 0.040928 | 0.361379 | 0.428552 | 0.357801 | 0.459916 | 0.597966 | 0.663315 | 0.49408 | 0.295995 | 0.3028 | 0.167187 | 0.475448 | 0.136568 | 0.467925 | 0.240643 | 0.247435 | 0.462897 | 0.288533 | 0.57073 | 0.416586 | 0.246301 | 0.064118 | 0.545156 | 0.359884 | 0.323522 | 0.319285 | 0.294305 | 0.255819 |
| TCGA-F4-6460-01A-11R-1774-07 | 0.095222 | 0.377839 | 0.440379 | 0.329038 | 0.434413 | 0.533463 | 0.671111 | 0.470053 | 0.340757 | 0.323603 | 0.087533 | 0.473098 | 0.167961 | 0.424481 | 0.214434 | 0.106235 | 0.437589 | 0.270243 | 0.549021 | 0.47776 | 0.264924 | -0.01912 | 0.550161 | 0.3504 | 0.320348 | 0.323534 | 0.239115 | 0.294988 |
| TCGA-AZ-4308-01A-01R-1410-07 | -0.11559 | 0.261479 | 0.456333 | 0.399239 | 0.46459 | 0.622683 | 0.637392 | 0.490241 | 0.25056 | 0.285815 | 0.0518 | 0.482377 | 0.019946 | 0.43126 | 0.246711 | 0.164903 | 0.477843 | 0.128874 | 0.561199 | 0.453014 | 0.257694 | 0.083805 | 0.511195 | 0.366105 | 0.32335 | 0.328265 | 0.29725 | 0.248608 |
| TCGA-AA-3972-01A-01R-1022-07 | -0.0949 | 0.367387 | 0.347253 | 0.336542 | 0.495924 | 0.525591 | 0.607639 | 0.506202 | 0.317016 | 0.255538 | 0.119891 | 0.492977 | 0.049544 | 0.492066 | 0.188375 | 0.184382 | 0.401708 | 0.269122 | 0.54095 | 0.41597 | 0.237858 | 0.114299 | 0.512846 | 0.350099 | 0.261065 | 0.303514 | 0.215293 | 0.285651 |
| TCGA-AA-3858-01A-01R-0905-07 | 0.102187 | 0.420856 | 0.451615 | 0.38119 | 0.476035 | 0.556111 | 0.629599 | 0.492804 | 0.296072 | 0.292518 | 0.101864 | 0.49574 | 0.150089 | 0.46706 | 0.211702 | 0.20501 | 0.45583 | 0.254643 | 0.562779 | 0.436751 | 0.252588 | 0.072273 | 0.473725 | 0.340812 | 0.313924 | 0.335902 | 0.284676 | 0.270911 |
| TCGA-AA-3679-01A-02R-0905-07 | 0.09071 | 0.367781 | 0.443291 | 0.363058 | 0.461994 | 0.563872 | 0.624407 | 0.466958 | 0.294662 | 0.274925 | 0.103609 | 0.493788 | 0.12558 | 0.488084 | 0.224708 | 0.150863 | 0.443432 | 0.24537 | 0.580416 | 0.413191 | 0.28312 | 0.132754 | 0.512347 | 0.337048 | 0.310492 | 0.320862 | 0.272429 | 0.24649 |
| TCGA-AA-A01D-01A-01R-A00A-07 | 0.027444 | 0.365324 | 0.392255 | 0.42253 | 0.440215 | 0.595227 | 0.685558 | 0.526672 | 0.303482 | 0.374832 | 0.152969 | 0.484693 | 0.115428 | 0.53747 | 0.301295 | 0.240795 | 0.524939 | 0.273795 | 0.588711 | 0.468313 | 0.273978 | 0.09784 | 0.5448 | 0.446016 | 0.335299 | 0.332156 | 0.294231 | 0.316508 |
| TCGA-AY-5543-01A-01R-1653-07 | 0.107023 | 0.493578 | 0.494049 | 0.383745 | 0.45877 | 0.544029 | 0.633045 | 0.477092 | 0.284211 | 0.360279 | 0.178835 | 0.468882 | 0.200845 | 0.453962 | 0.164453 | 0.180844 | 0.432093 | 0.269807 | 0.527112 | 0.426574 | 0.258262 | 0.025867 | 0.500058 | 0.322932 | 0.291072 | 0.345248 | 0.324902 | 0.345976 |
| TCGA-AU-6004-01A-11R-1723-07 | 0.183466 | 0.498249 | 0.469889 | 0.485944 | 0.414176 | 0.540798 | 0.681958 | 0.565681 | 0.323991 | 0.428383 | 0.100625 | 0.51532 | 0.271369 | 0.517807 | 0.302579 | 0.219087 | 0.562331 | 0.287933 | 0.567231 | 0.474912 | 0.302619 | 0.167081 | 0.525721 | 0.466366 | 0.371704 | 0.374881 | 0.2798 | 0.351939 |
| TCGA-DM-A28A-01A-21R-A32Y-07 | -0.09066 | 0.398017 | 0.392544 | 0.327426 | 0.462802 | 0.52958 | 0.626207 | 0.487553 | 0.302012 | 0.251883 | 0.086513 | 0.484719 | 0.015079 | 0.415193 | 0.153875 | 0.097583 | 0.399103 | 0.267669 | 0.524108 | 0.426674 | 0.248141 | -0.03899 | 0.493639 | 0.321236 | 0.256905 | 0.284801 | 0.185397 | 0.241753 |
| TCGA-AA-3848-01A-01R-0905-07 | -0.03552 | 0.305237 | 0.42564 | 0.329881 | 0.456579 | 0.622041 | 0.624991 | 0.47006 | 0.212176 | 0.306976 | 0.072051 | 0.427838 | 0.057966 | 0.442874 | 0.198473 | 0.152611 | 0.426684 | 0.270854 | 0.565424 | 0.395502 | 0.263285 | 0.032289 | 0.486267 | 0.336074 | 0.296199 | 0.301979 | 0.228167 | 0.201455 |
| TCGA-AA-3667-01A-01R-0905-07 | 0.022591 | 0.335276 | 0.440883 | 0.370356 | 0.464536 | 0.621102 | 0.63571 | 0.497393 | 0.242034 | 0.323178 | 0.091819 | 0.4757 | 0.087491 | 0.466596 | 0.224506 | 0.165025 | 0.479817 | 0.251457 | 0.581226 | 0.422654 | 0.22519 | 0.181038 | 0.506949 | 0.389525 | 0.313717 | 0.318526 | 0.315358 | 0.218504 |
| TCGA-F5-6810-01A-11R-1830-07 | -0.05473 | 0.416423 | 0.402046 | 0.331653 | 0.446573 | 0.494172 | 0.632046 | 0.486397 | 0.377061 | 0.284402 | 0.124713 | 0.466459 | 0.055182 | 0.482604 | 0.181356 | 0.187008 | 0.416469 | 0.294808 | 0.522311 | 0.465002 | 0.298493 | 0.053691 | 0.543853 | 0.33195 | 0.314967 | 0.323808 | 0.222388 | 0.328337 |
| TCGA-AG-A01W-01A-21R-A083-07 | 0.087587 | 0.477779 | 0.457823 | 0.346891 | 0.430543 | 0.548168 | 0.601099 | 0.461399 | 0.270268 | 0.300322 | 0.068416 | 0.472098 | 0.163415 | 0.446169 | 0.182289 | 0.14411 | 0.390649 | 0.27806 | 0.534304 | 0.40636 | 0.236385 | -0.00118 | 0.495112 | 0.322144 | 0.283921 | 0.303794 | 0.267309 | 0.277068 |
| TCGA-AG-3878-01A-02R-0905-07 | 0.194451 | 0.355755 | 0.528318 | 0.383418 | 0.469716 | 0.63232 | 0.67443 | 0.572377 | 0.283881 | 0.408831 | 0.108579 | 0.517266 | 0.236325 | 0.476017 | 0.315612 | 0.227774 | 0.568132 | 0.267049 | 0.615989 | 0.468708 | 0.286528 | 0.189787 | 0.508732 | 0.431535 | 0.395084 | 0.387698 | 0.363822 | 0.271026 |
| TCGA-EI-6507-01A-11R-1736-07 | 0.011731 | 0.532611 | 0.531884 | 0.431242 | 0.436936 | 0.606931 | 0.696673 | 0.585221 | 0.374799 | 0.414929 | 0.12229 | 0.521235 | 0.178237 | 0.504611 | 0.289617 | 0.25092 | 0.629297 | 0.246144 | 0.516804 | 0.4842 | 0.313924 | 0.17428 | 0.587107 | 0.523775 | 0.36118 | 0.367586 | 0.240548 | 0.380013 |
| TCGA-CI-6621-01A-11R-1830-07 | 0.025387 | 0.464259 | 0.480798 | 0.408424 | 0.46589 | 0.525644 | 0.651147 | 0.534568 | 0.357202 | 0.356574 | 0.141391 | 0.517035 | 0.154753 | 0.489558 | 0.277228 | 0.311618 | 0.531051 | 0.277098 | 0.545389 | 0.456513 | 0.288538 | 0.203234 | 0.56239 | 0.460256 | 0.353105 | 0.360017 | 0.28208 | 0.325344 |
| TCGA-AG-A020-01A-21R-A083-07 | -0.10892 | 0.407392 | 0.418056 | 0.312562 | 0.436013 | 0.577925 | 0.596662 | 0.41665 | 0.253956 | 0.254922 | 0.077323 | 0.43907 | -0.01238 | 0.473922 | 0.109499 | 0.045429 | 0.324723 | 0.225204 | 0.528483 | 0.34462 | 0.199963 | 0.051714 | 0.484021 | 0.249875 | 0.228332 | 0.267958 | 0.293134 | 0.296882 |
| TCGA-AG-4008-01A-01R-1119-07 | 0.011246 | 0.372374 | 0.42644 | 0.381205 | 0.447034 | 0.569903 | 0.645485 | 0.525763 | 0.319987 | 0.311335 | 0.092856 | 0.522352 | 0.146019 | 0.528562 | 0.248841 | 0.245074 | 0.476612 | 0.283233 | 0.542462 | 0.453774 | 0.26959 | 0.113759 | 0.579602 | 0.421808 | 0.341109 | 0.34876 | 0.261623 | 0.258091 |
| TCGA-AG-3602-01A-02R-0826-07 | 0.078343 | 0.463982 | 0.528372 | 0.384398 | 0.459721 | 0.564878 | 0.629394 | 0.468214 | 0.278307 | 0.329867 | 0.12874 | 0.515488 | 0.15553 | 0.515771 | 0.216312 | 0.190254 | 0.481203 | 0.255393 | 0.564724 | 0.41082 | 0.25964 | 0.164714 | 0.517284 | 0.393785 | 0.317139 | 0.354953 | 0.310998 | 0.281828 |
| TCGA-AG-3909-01A-01R-1119-07 | 0.147476 | 0.334406 | 0.439543 | 0.358838 | 0.462471 | 0.600284 | 0.640122 | 0.514349 | 0.285267 | 0.303881 | 0.107937 | 0.503278 | 0.201495 | 0.466842 | 0.240498 | 0.178241 | 0.454 | 0.279172 | 0.575377 | 0.42936 | 0.242757 | 0.057419 | 0.517929 | 0.365124 | 0.327933 | 0.336064 | 0.324342 | 0.233255 |
| TCGA-DC-6156-01A-11R-1660-07 | 0.055176 | 0.439105 | 0.54892 | 0.43145 | 0.468056 | 0.537153 | 0.68863 | 0.554533 | 0.376365 | 0.460318 | 0.134323 | 0.540652 | 0.198873 | 0.549906 | 0.312365 | 0.253436 | 0.606613 | 0.32737 | 0.564427 | 0.528202 | 0.305489 | 0.080815 | 0.557248 | 0.568287 | 0.395718 | 0.394424 | 0.223885 | 0.270922 |
| TCGA-EI-6510-01A-11R-1736-07 | 0.085659 | 0.429257 | 0.456209 | 0.340351 | 0.439317 | 0.520388 | 0.637782 | 0.449646 | 0.275257 | 0.303626 | 0.083404 | 0.439229 | 0.165373 | 0.435372 | 0.153643 | 0.068537 | 0.401445 | 0.306638 | 0.538485 | 0.400099 | 0.177293 | -0.01007 | 0.495397 | 0.246932 | 0.258479 | 0.307544 | 0.319073 | 0.327687 |
| TCGA-AG-3728-01A-01R-0905-07 | 0.248171 | 0.333053 | 0.523224 | 0.411483 | 0.462578 | 0.605702 | 0.657267 | 0.479929 | 0.303141 | 0.405486 | 0.113143 | 0.495546 | 0.267445 | 0.480972 | 0.293694 | 0.264846 | 0.575943 | 0.256316 | 0.603698 | 0.47072 | 0.299406 | 0.100032 | 0.518411 | 0.420649 | 0.387228 | 0.376742 | 0.303742 | 0.249444 |
| TCGA-AG-A015-01A-01R-A002-07 | -0.05415 | 0.368609 | 0.351544 | 0.297051 | 0.461017 | 0.546649 | 0.652332 | 0.443405 | 0.262813 | 0.255952 | 0.102621 | 0.424942 | 0.049876 | 0.440601 | 0.133481 | 0.07765 | 0.391516 | 0.282717 | 0.538359 | 0.368195 | 0.183734 | -0.00879 | 0.476675 | 0.242428 | 0.275244 | 0.283331 | 0.301722 | 0.32174 |
| TCGA-CI-6620-01A-11R-1830-07 | 0.126521 | 0.460635 | 0.435308 | 0.360874 | 0.426851 | 0.525691 | 0.627682 | 0.477658 | 0.34479 | 0.293847 | 0.131909 | 0.481662 | 0.181733 | 0.499982 | 0.21505 | 0.228663 | 0.437532 | 0.29254 | 0.52897 | 0.435031 | 0.229525 | 0.081372 | 0.536684 | 0.344066 | 0.28697 | 0.309292 | 0.249413 | 0.349386 |
| TCGA-AH-6643-01A-11R-1830-07 | -0.14266 | 0.402316 | 0.392165 | 0.358922 | 0.435282 | 0.514819 | 0.658722 | 0.52936 | 0.339849 | 0.272913 | 0.081414 | 0.462387 | 0.032301 | 0.49533 | 0.197883 | 0.147776 | 0.407486 | 0.216602 | 0.54347 | 0.435355 | 0.254454 | 0.152671 | 0.506368 | 0.343315 | 0.265209 | 0.313961 | 0.239239 | 0.332175 |
| TCGA-EI-6513-01A-21R-1736-07 | -0.08301 | 0.415811 | 0.412884 | 0.334684 | 0.457081 | 0.53534 | 0.623542 | 0.480226 | 0.29722 | 0.28359 | 0.094547 | 0.47493 | 0.061283 | 0.497276 | 0.171662 | 0.158243 | 0.420565 | 0.25154 | 0.534291 | 0.437093 | 0.249507 | 0.029031 | 0.536391 | 0.342838 | 0.307612 | 0.310248 | 0.203193 | 0.244003 |
| TCGA-EI-6506-01A-11R-1736-07 | 0.128947 | 0.459023 | 0.464952 | 0.400467 | 0.463175 | 0.552958 | 0.647762 | 0.519193 | 0.326562 | 0.35786 | 0.166731 | 0.509117 | 0.211354 | 0.490477 | 0.270633 | 0.301895 | 0.499812 | 0.294358 | 0.557073 | 0.441582 | 0.263121 | 0.206464 | 0.537322 | 0.399771 | 0.340664 | 0.343741 | 0.31095 | 0.302848 |
| TCGA-AG-4005-01A-01R-1119-07 | 0.001038 | 0.390675 | 0.398374 | 0.381918 | 0.442291 | 0.571128 | 0.633574 | 0.500993 | 0.311184 | 0.261867 | 0.135959 | 0.497352 | 0.112169 | 0.506514 | 0.23539 | 0.176524 | 0.445977 | 0.275523 | 0.527469 | 0.429189 | 0.270112 | 0.093059 | 0.527732 | 0.384017 | 0.308843 | 0.322425 | 0.255876 | 0.273743 |
| TCGA-AF-A56K-01A-32R-A39D-07 | 0.018826 | 0.41413 | 0.446391 | 0.385471 | 0.448874 | 0.551757 | 0.657172 | 0.502971 | 0.359966 | 0.348118 | 0.188201 | 0.477003 | 0.156404 | 0.450839 | 0.231414 | 0.267825 | 0.472056 | 0.29518 | 0.522624 | 0.466318 | 0.27361 | 0.047465 | 0.572049 | 0.407501 | 0.325463 | 0.337084 | 0.210507 | 0.244785 |
| TCGA-AA-A00F-01A-01R-A002-07 | 0.035207 | 0.356605 | 0.409649 | 0.322356 | 0.452229 | 0.574442 | 0.662697 | 0.482283 | 0.297135 | 0.306374 | 0.054552 | 0.491422 | 0.085848 | 0.428583 | 0.184847 | 0.121222 | 0.421367 | 0.257268 | 0.551408 | 0.436917 | 0.281604 | 0.055797 | 0.516681 | 0.323601 | 0.289809 | 0.331825 | 0.277875 | 0.278429 |
| TCGA-AA-3866-01A-01R-1022-07 | 0.141944 | 0.507159 | 0.527894 | 0.448699 | 0.468467 | 0.550534 | 0.671196 | 0.590122 | 0.357154 | 0.424398 | 0.152348 | 0.557438 | 0.258104 | 0.503241 | 0.344519 | 0.277293 | 0.629106 | 0.298517 | 0.573453 | 0.50476 | 0.312058 | 0.119602 | 0.525163 | 0.535199 | 0.3851 | 0.422235 | 0.233917 | 0.312849 |
| TCGA-AA-A01K-01A-01R-A00A-07 | 0.024174 | 0.41546 | 0.478241 | 0.37364 | 0.466857 | 0.577015 | 0.665024 | 0.499248 | 0.290426 | 0.33849 | 0.053235 | 0.491997 | 0.144309 | 0.472906 | 0.228594 | 0.16584 | 0.470595 | 0.248197 | 0.551629 | 0.440931 | 0.282425 | -0.00453 | 0.522481 | 0.392901 | 0.310349 | 0.343756 | 0.225694 | 0.309622 |
| TCGA-CM-5863-01A-21R-1839-07 | 0.056309 | 0.381328 | 0.400235 | 0.378768 | 0.454034 | 0.587117 | 0.677662 | 0.550073 | 0.318904 | 0.365971 | 0.127033 | 0.511844 | 0.128372 | 0.526619 | 0.29443 | 0.23414 | 0.537636 | 0.276802 | 0.557433 | 0.480336 | 0.28061 | 0.107605 | 0.537373 | 0.41423 | 0.342345 | 0.359515 | 0.296359 | 0.342182 |
| TCGA-D5-6932-01A-11R-1928-07 | -0.04942 | 0.381359 | 0.457864 | 0.373002 | 0.471488 | 0.570324 | 0.66004 | 0.519585 | 0.321988 | 0.309919 | 0.140687 | 0.494692 | 0.103851 | 0.45724 | 0.220856 | 0.189399 | 0.477755 | 0.27014 | 0.525231 | 0.46624 | 0.279153 | 0.027352 | 0.522573 | 0.401792 | 0.312583 | 0.331482 | 0.236989 | 0.273736 |
| TCGA-AA-3675-01A-02R-0905-07 | -0.00021 | 0.425884 | 0.410549 | 0.351202 | 0.477354 | 0.510888 | 0.630762 | 0.503172 | 0.333976 | 0.278728 | 0.133185 | 0.486599 | 0.111722 | 0.503961 | 0.213862 | 0.176467 | 0.447892 | 0.225326 | 0.546933 | 0.421188 | 0.2086 | 0.170597 | 0.488326 | 0.326328 | 0.294958 | 0.335147 | 0.295476 | 0.325241 |
| TCGA-AA-3681-01A-01R-0905-07 | 0.156793 | 0.479641 | 0.50269 | 0.413881 | 0.45039 | 0.586587 | 0.657211 | 0.513709 | 0.29109 | 0.374426 | 0.20132 | 0.523747 | 0.236315 | 0.472799 | 0.230037 | 0.21765 | 0.523006 | 0.301729 | 0.550341 | 0.45485 | 0.296883 | 0.181151 | 0.53727 | 0.422244 | 0.344546 | 0.363649 | 0.33931 | 0.339901 |
| TCGA-AA-A00Q-01A-01R-A002-07 | -0.00569 | 0.363417 | 0.456847 | 0.338332 | 0.441204 | 0.55931 | 0.638943 | 0.465781 | 0.279272 | 0.291654 | 0.06566 | 0.470418 | 0.102469 | 0.44524 | 0.178365 | 0.087413 | 0.432829 | 0.260142 | 0.532008 | 0.430733 | 0.218765 | -0.02996 | 0.470291 | 0.30737 | 0.308913 | 0.304908 | 0.275633 | 0.263046 |
| TCGA-AM-5820-01A-01R-1653-07 | -0.07085 | 0.406327 | 0.373112 | 0.372481 | 0.455941 | 0.521078 | 0.590456 | 0.537533 | 0.351201 | 0.26684 | 0.082059 | 0.484062 | 0.058041 | 0.461685 | 0.20444 | 0.124624 | 0.404665 | 0.243821 | 0.53119 | 0.442794 | 0.227597 | 0.06687 | 0.514248 | 0.352721 | 0.324632 | 0.33228 | 0.181634 | 0.317266 |
| TCGA-CK-6746-01A-11R-1839-07 | -0.03075 | 0.518734 | 0.553823 | 0.367722 | 0.435912 | 0.530119 | 0.655579 | 0.493414 | 0.337233 | 0.477227 | 0.061985 | 0.488716 | 0.183825 | 0.453196 | 0.251975 | 0.170033 | 0.52308 | 0.237843 | 0.53639 | 0.477449 | 0.28866 | -0.01448 | 0.508709 | 0.437914 | 0.360071 | 0.337577 | 0.262643 | 0.382513 |
| TCGA-AA-A00W-01A-01R-A002-07 | 0.037029 | 0.422587 | 0.438992 | 0.330941 | 0.467178 | 0.540918 | 0.607351 | 0.429 | 0.252171 | 0.257014 | 0.131448 | 0.440708 | 0.099765 | 0.454115 | 0.159995 | 0.043111 | 0.364674 | 0.275344 | 0.560788 | 0.366691 | 0.198122 | 0.111689 | 0.476425 | 0.247178 | 0.232409 | 0.281011 | 0.325256 | 0.290295 |
| TCGA-AZ-4323-01A-21R-1839-07 | 0.521832 | 0.539482 | 0.634307 | 0.395132 | 0.478723 | 0.567945 | 0.722334 | 0.491753 | 0.426763 | 0.451899 | 0.271683 | 0.489325 | 0.488275 | 0.460555 | 0.245768 | 0.209895 | 0.565674 | 0.327797 | 0.562119 | 0.488442 | 0.28094 | -0.01155 | 0.535279 | 0.406544 | 0.385789 | 0.414649 | 0.157601 | 0.334185 |
| TCGA-CM-4746-01A-01R-1410-07 | -0.11793 | 0.400799 | 0.469042 | 0.344582 | 0.440352 | 0.582601 | 0.624751 | 0.426863 | 0.320105 | 0.299239 | 0.06041 | 0.485745 | -0.01244 | 0.490739 | 0.149524 | 0.113141 | 0.432898 | 0.161516 | 0.55228 | 0.380824 | 0.226098 | 0.085967 | 0.469668 | 0.246665 | 0.230376 | 0.297306 | 0.272123 | 0.3093 |
| TCGA-AG-A00H-01A-01R-A00A-07 | -0.09946 | 0.316546 | 0.3696 | 0.315093 | 0.451008 | 0.566928 | 0.644289 | 0.489767 | 0.299528 | 0.262013 | 0.053941 | 0.50631 | 0.014051 | 0.459085 | 0.173116 | 0.148237 | 0.405822 | 0.249018 | 0.530289 | 0.440737 | 0.240172 | -0.03565 | 0.519171 | 0.351071 | 0.291586 | 0.313155 | 0.268138 | 0.229349 |
| TCGA-EI-6511-01A-11R-1736-07 | 0.208223 | 0.519269 | 0.597166 | 0.429033 | 0.473321 | 0.581231 | 0.66829 | 0.574869 | 0.370807 | 0.420425 | 0.135722 | 0.571935 | 0.283617 | 0.472189 | 0.283237 | 0.278393 | 0.603666 | 0.330155 | 0.598448 | 0.496501 | 0.309007 | 0.208216 | 0.56869 | 0.468742 | 0.380376 | 0.3978 | 0.30481 | 0.331893 |
| TCGA-EI-6883-01A-31R-1928-07 | 0.078055 | 0.394545 | 0.5137 | 0.342697 | 0.47062 | 0.578262 | 0.646256 | 0.470438 | 0.308201 | 0.329451 | 0.135216 | 0.470269 | 0.150912 | 0.43564 | 0.188838 | 0.153806 | 0.412898 | 0.302315 | 0.565956 | 0.44502 | 0.273434 | -0.00725 | 0.506747 | 0.354907 | 0.340495 | 0.326267 | 0.260928 | 0.271475 |
| TCGA-DC-6155-01A-11R-1660-07 | 0.04644 | 0.358296 | 0.416134 | 0.319246 | 0.442639 | 0.582785 | 0.595906 | 0.423473 | 0.267877 | 0.294172 | 0.07761 | 0.416356 | 0.061004 | 0.467097 | 0.26996 | 0.060843 | 0.430699 | 0.20898 | 0.557493 | 0.38381 | 0.226913 | 0.078788 | 0.499949 | 0.273277 | 0.268585 | 0.278498 | 0.257671 | 0.223499 |
| TCGA-AG-3593-01A-01R-0821-07 | 0.072913 | 0.464279 | 0.495177 | 0.37213 | 0.468093 | 0.528752 | 0.650895 | 0.474945 | 0.293971 | 0.335528 | 0.150426 | 0.499862 | 0.174208 | 0.493737 | 0.254858 | 0.125973 | 0.519339 | 0.287307 | 0.532143 | 0.436871 | 0.218079 | 0.066287 | 0.54284 | 0.421 | 0.324365 | 0.341145 | 0.282302 | 0.295223 |
| TCGA-AG-3601-01A-01R-0826-07 | -0.07616 | 0.350534 | 0.392891 | 0.335465 | 0.466946 | 0.564942 | 0.62979 | 0.482651 | 0.306112 | 0.267777 | 0.055124 | 0.476555 | 0.070939 | 0.461326 | 0.184233 | 0.146908 | 0.421265 | 0.247087 | 0.547883 | 0.419105 | 0.231789 | -0.02145 | 0.518698 | 0.331057 | 0.274574 | 0.311972 | 0.220494 | 0.20892 |
| TCGA-A6-6141-01A-11R-1774-07 | 0.202303 | 0.443614 | 0.535786 | 0.379114 | 0.46845 | 0.567983 | 0.689443 | 0.475287 | 0.33575 | 0.381949 | 0.129432 | 0.45736 | 0.23798 | 0.493911 | 0.220599 | 0.138077 | 0.476958 | 0.335996 | 0.557226 | 0.457295 | 0.240437 | -0.02674 | 0.527636 | 0.334903 | 0.324069 | 0.364801 | 0.314434 | 0.359067 |
| TCGA-DM-A280-01A-12R-A16W-07 | -0.18125 | 0.387664 | 0.366744 | 0.410792 | 0.446338 | 0.543777 | 0.645683 | 0.522553 | 0.289144 | 0.251917 | 0.097574 | 0.498167 | 0.031853 | 0.474039 | 0.179564 | 0.13497 | 0.415304 | 0.243989 | 0.59424 | 0.427769 | 0.242434 | 0.18538 | 0.514736 | 0.315323 | 0.302191 | 0.315296 | 0.23418 | 0.321891 |
| TCGA-AA-3939-01A-01R-1022-07 | 0.112821 | 0.489209 | 0.502149 | 0.399557 | 0.470038 | 0.542474 | 0.643345 | 0.505002 | 0.346143 | 0.340775 | 0.232282 | 0.510423 | 0.191322 | 0.508748 | 0.287052 | 0.233945 | 0.484329 | 0.31131 | 0.564257 | 0.418138 | 0.266192 | 0.144833 | 0.515347 | 0.380145 | 0.310053 | 0.373175 | 0.310382 | 0.324611 |
| TCGA-A6-2679-01A-02R-1410-07 | 0.189795 | 0.359486 | 0.546681 | 0.35953 | 0.46937 | 0.618091 | 0.65695 | 0.499746 | 0.23046 | 0.415636 | 0.021015 | 0.483811 | 0.172298 | 0.440948 | 0.232383 | 0.239533 | 0.538895 | 0.1961 | 0.601588 | 0.403921 | 0.250692 | 0.037671 | 0.504144 | 0.330773 | 0.288627 | 0.370447 | 0.344312 | 0.224954 |
| TCGA-AA-3502-01A-01R-1410-07 | 0.099331 | 0.402242 | 0.492394 | 0.366787 | 0.458468 | 0.54167 | 0.659916 | 0.472421 | 0.337616 | 0.365123 | 0.128195 | 0.488756 | 0.184556 | 0.46728 | 0.215467 | 0.147276 | 0.451513 | 0.281856 | 0.561749 | 0.385678 | 0.226393 | -0.10282 | 0.484772 | 0.310595 | 0.304689 | 0.307498 | 0.293198 | 0.346856 |
| TCGA-D5-6533-01A-11R-1723-07 | 0.014265 | 0.460277 | 0.418581 | 0.379867 | 0.443411 | 0.544955 | 0.627962 | 0.491818 | 0.3254 | 0.297661 | 0.138866 | 0.478933 | 0.138828 | 0.458991 | 0.176192 | 0.136445 | 0.445643 | 0.307768 | 0.541158 | 0.441591 | 0.236439 | 0.119879 | 0.529212 | 0.396062 | 0.314808 | 0.330775 | 0.279561 | 0.303499 |
| TCGA-AA-3693-01A-01R-0905-07 | -0.07092 | 0.371636 | 0.417726 | 0.32942 | 0.47774 | 0.532759 | 0.607383 | 0.457211 | 0.317297 | 0.260496 | 0.081158 | 0.488598 | 0.078393 | 0.464889 | 0.161757 | 0.131653 | 0.362244 | 0.317146 | 0.539746 | 0.421206 | 0.249053 | 0.024537 | 0.475216 | 0.335024 | 0.272163 | 0.324378 | 0.234393 | 0.251857 |
| TCGA-AD-6548-01A-11R-1839-07 | 0.107467 | 0.522713 | 0.518511 | 0.434675 | 0.442052 | 0.542498 | 0.64397 | 0.551918 | 0.350755 | 0.383065 | 0.146554 | 0.543243 | 0.217224 | 0.487768 | 0.262159 | 0.289866 | 0.551966 | 0.305675 | 0.546912 | 0.458415 | 0.304651 | 0.138378 | 0.542983 | 0.455484 | 0.364709 | 0.376364 | 0.262054 | 0.337955 |
| TCGA-CM-6170-01A-11R-1653-07 | 0.095358 | 0.399168 | 0.446612 | 0.367337 | 0.460117 | 0.523802 | 0.649088 | 0.506449 | 0.350803 | 0.314653 | 0.139113 | 0.486854 | 0.192789 | 0.478077 | 0.226827 | 0.22748 | 0.497597 | 0.301866 | 0.550947 | 0.464719 | 0.268848 | 0.071182 | 0.543322 | 0.406226 | 0.31756 | 0.33861 | 0.239395 | 0.24759 |
| TCGA-F4-6459-01A-11R-1774-07 | 0.02698 | 0.378444 | 0.407954 | 0.369092 | 0.44682 | 0.560652 | 0.646431 | 0.521949 | 0.32077 | 0.335894 | 0.130275 | 0.488607 | 0.126847 | 0.518727 | 0.239202 | 0.253126 | 0.464704 | 0.288546 | 0.532022 | 0.479116 | 0.278776 | 0.009424 | 0.536234 | 0.401124 | 0.361187 | 0.345076 | 0.189494 | 0.297148 |
| TCGA-DM-A1D0-01A-11R-A155-07 | -0.25031 | 0.303522 | 0.283884 | 0.272891 | 0.467157 | 0.533577 | 0.575158 | 0.427348 | 0.23597 | 0.174961 | 0.015758 | 0.419322 | -0.09065 | 0.451427 | 0.096029 | 0.036042 | 0.248766 | 0.244691 | 0.532493 | 0.326774 | 0.146442 | -0.10314 | 0.481426 | 0.200499 | 0.222075 | 0.235789 | 0.182189 | 0.256525 |
| TCGA-NH-A8F7-01A-11R-A41B-07 | -0.13836 | 0.29458 | 0.302973 | 0.270102 | 0.449416 | 0.517861 | 0.574498 | 0.439745 | 0.258144 | 0.167325 | 0.031719 | 0.426524 | -0.05131 | 0.420606 | 0.112481 | 0.068821 | 0.267797 | 0.283 | 0.517984 | 0.381432 | 0.165917 | 0.117458 | 0.505065 | 0.243038 | 0.23785 | 0.237871 | 0.167498 | 0.19584 |
| TCGA-AA-A02W-01A-01R-A00A-07 | -0.02424 | 0.3676 | 0.427829 | 0.302221 | 0.458404 | 0.522816 | 0.627998 | 0.438829 | 0.302794 | 0.231054 | 0.055228 | 0.432609 | 0.080621 | 0.451241 | 0.13485 | 0.191526 | 0.326365 | 0.31416 | 0.514612 | 0.423238 | 0.215349 | -0.07801 | 0.524868 | 0.278289 | 0.270087 | 0.288437 | 0.250636 | 0.26881 |
| TCGA-AA-3524-01A-02R-0821-07 | -0.02366 | 0.485249 | 0.486311 | 0.344244 | 0.44651 | 0.549806 | 0.60095 | 0.485359 | 0.328818 | 0.283838 | 0.072807 | 0.509683 | 0.137175 | 0.493825 | 0.211669 | 0.193932 | 0.422991 | 0.251442 | 0.585432 | 0.420508 | 0.230484 | 0.164447 | 0.478425 | 0.353806 | 0.320561 | 0.347365 | 0.253567 | 0.273461 |
| TCGA-AA-3841-01A-01R-0905-07 | 0.164212 | 0.354459 | 0.488325 | 0.392447 | 0.471992 | 0.637256 | 0.670402 | 0.517764 | 0.261985 | 0.356429 | 0.108918 | 0.485265 | 0.179591 | 0.500082 | 0.266353 | 0.203011 | 0.51236 | 0.28313 | 0.631884 | 0.44464 | 0.27973 | 0.155104 | 0.503422 | 0.408398 | 0.344007 | 0.36258 | 0.30137 | 0.27459 |
| TCGA-A6-5659-01A-01R-A278-07 | -0.0127 | 0.435566 | 0.373039 | 0.319286 | 0.425979 | 0.496274 | 0.586745 | 0.471626 | 0.365122 | 0.245589 | 0.11414 | 0.443796 | 0.114899 | 0.443453 | 0.18194 | 0.230225 | 0.369991 | 0.343521 | 0.510217 | 0.435426 | 0.198253 | 0.020187 | 0.476743 | 0.394723 | 0.283256 | 0.298844 | 0.213365 | 0.308567 |
| TCGA-A6-2674-01A-02R-A278-07 | 0.086319 | 0.554626 | 0.41099 | 0.500661 | 0.393439 | 0.515392 | 0.699048 | 0.578484 | 0.391187 | 0.393309 | 0.17055 | 0.536899 | 0.248595 | 0.543418 | 0.342017 | 0.31659 | 0.593985 | 0.354618 | 0.517532 | 0.511083 | 0.276279 | 0.401956 | 0.580568 | 0.549325 | 0.37651 | 0.367581 | 0.258433 | 0.365052 |
| TCGA-A6-6650-01A-11R-A278-07 | -0.1011 | 0.433323 | 0.336814 | 0.330454 | 0.407166 | 0.523215 | 0.566613 | 0.412678 | 0.294414 | 0.230607 | 0.117491 | 0.409459 | 0.064865 | 0.418905 | 0.091361 | 0.11 | 0.327687 | 0.310114 | 0.526005 | 0.395719 | 0.160898 | -0.02895 | 0.451823 | 0.301602 | 0.252919 | 0.256186 | 0.228791 | 0.303319 |
| TCGA-A6-3810-01A-01R-A278-07 | -0.03011 | 0.440008 | 0.380572 | 0.425875 | 0.447852 | 0.54127 | 0.667358 | 0.539948 | 0.317957 | 0.345316 | 0.186666 | 0.49551 | 0.156809 | 0.497205 | 0.223881 | 0.246969 | 0.510911 | 0.303889 | 0.51068 | 0.494382 | 0.244848 | 0.124953 | 0.535125 | 0.479268 | 0.308476 | 0.330309 | 0.213381 | 0.282662 |
| TCGA-A6-5656-01A-21R-A278-07 | -0.21188 | 0.366631 | 0.29222 | 0.303565 | 0.369789 | 0.527621 | 0.576179 | 0.401339 | 0.296499 | 0.20858 | 0.112412 | 0.403738 | 0.007753 | 0.418689 | 0.078079 | 0.09992 | 0.267044 | 0.321537 | 0.479587 | 0.39745 | 0.153098 | -0.00924 | 0.475034 | 0.291909 | 0.244798 | 0.258983 | 0.204592 | 0.227689 |
| TCGA-A6-6780-01A-11R-A278-07 | 0.03566 | 0.642985 | 0.526014 | 0.400744 | 0.420275 | 0.501441 | 0.633413 | 0.494316 | 0.352195 | 0.419988 | 0.119093 | 0.523556 | 0.235788 | 0.502243 | 0.26406 | 0.189839 | 0.56214 | 0.26295 | 0.498205 | 0.492042 | 0.276501 | 0.057375 | 0.45998 | 0.487319 | 0.374443 | 0.347345 | 0.265424 | 0.397527 |
| TCGA-A6-2684-01A-01R-A278-07 | 0.021444 | 0.496412 | 0.404735 | 0.410318 | 0.391155 | 0.516386 | 0.673605 | 0.4987 | 0.331939 | 0.377344 | 0.155409 | 0.503429 | 0.173364 | 0.489233 | 0.23725 | 0.250661 | 0.504305 | 0.297979 | 0.532293 | 0.491517 | 0.234065 | -0.01441 | 0.507051 | 0.513224 | 0.345546 | 0.356215 | 0.249036 | 0.317391 |
| TCGA-A6-3809-01A-01R-A278-07 | 0.111435 | 0.656827 | 0.511478 | 0.465637 | 0.406838 | 0.513004 | 0.644712 | 0.532607 | 0.351639 | 0.452656 | 0.153358 | 0.51684 | 0.294649 | 0.530812 | 0.275737 | 0.254195 | 0.562263 | 0.277453 | 0.510565 | 0.490505 | 0.306601 | 0.191529 | 0.494283 | 0.497515 | 0.340966 | 0.352963 | 0.282391 | 0.448736 |
| TCGA-A6-6781-01A-22R-A278-07 | 0.028994 | 0.482946 | 0.462456 | 0.454947 | 0.411925 | 0.551547 | 0.693543 | 0.59214 | 0.380042 | 0.438311 | 0.140532 | 0.501843 | 0.2133 | 0.514063 | 0.329653 | 0.276276 | 0.615899 | 0.303338 | 0.522254 | 0.534269 | 0.293816 | 0.207394 | 0.544382 | 0.594441 | 0.37449 | 0.387297 | 0.259552 | 0.427706 |

Table S4 Hub modules

| Module | Cor(lymphangiogenesis) | Cor(top immnue infiltrion cells) | Gene number | MM > 0.6 and | GS | > 0.4 |
| --- | --- | --- | --- | --- |
| Brown | 0.78 | 0.81 | 888 | 529 |
| Purple | 0.87 | 0.75 | 457 | 332 |
| Greenyellow | 0.6 | 0.94 | 448 | 215 |

Table S5 Biological functions.

| ONTOLOGY | ID | Description | Gene Ratio | BgRatio | P value | p.adjust | Q value | Gene ID | Count |
| --- | --- | --- | --- | --- | --- | --- | --- | --- | --- |
| BP | GO:0030198 | extracellular matrix organization | 127/941 | 393/18862 | 7.30E-69 | 2.53E-65 | 1.81E-65 | A2M/ADAMTS1/ADAMTS16/ADAMTS3/ADAMTSL4/CAV1/CAV2/CCDC80/COL14A1/CRISPLD2/CYP1B1/DCN/DDR2/ECM2/FBLN1/FBLN2/FBLN5/FLRT2/GREM1/HSPG2/ITGA1/ITGA5/ITGB1/ITGB3/JAM3/LAMA2/LAMA4/LAMC1/LOXL4/MFAP5/MMP16/OLFML2A/PDGFRA/PHLDB1/PHLDB2/RECK/SFRP2/TIMP2/TNC/VCAM1/ADAM12/ADAM19/ADAMTS10/ADAMTS12/ADAMTS14/ADAMTS2/ADAMTS4/ADAMTS5/ADAMTS7/AEBP1/ANTXR1/BGN/BMP1/COL10A1/COL11A1/COL12A1/COL13A1/COL15A1/COL16A1/COL18A1/COL1A1/COL1A2/COL22A1/COL24A1/COL3A1/COL4A1/COL4A2/COL5A1/COL5A2/COL5A3/COL6A1/COL6A2/COL6A3/COL7A1/COL8A1/COL8A2/COMP/CTSK/EFEMP2/EGFLAM/ELN/EMILIN1/ENG/FAP/FBN1/FN1/HTRA1/ITGA11/KDR/LAMB2/LOX/LOXL1/LOXL2/LOXL3/LTBP3/LUM/MATN3/MFAP2/MMP14/MMP19/MMP2/NID1/NID2/NTNG2/PDGFB/PDPN/PECAM1/POSTN/PXDN/RAMP2/SERPINE1/SERPINH1/SH3PXD2B/SPARC/SULF1/THBS1/TIE1/VCAN/VWF/ADAM8/CTSL/ICAM1/ITGAM/ITGAX/ITGB2/MMP9/TGFB1 | 127 |
| BP | GO:0043062 | extracellular structure organization | 127/941 | 394/18862 | 1.03E-68 | 2.53E-65 | 1.81E-65 | A2M/ADAMTS1/ADAMTS16/ADAMTS3/ADAMTSL4/CAV1/CAV2/CCDC80/COL14A1/CRISPLD2/CYP1B1/DCN/DDR2/ECM2/FBLN1/FBLN2/FBLN5/FLRT2/GREM1/HSPG2/ITGA1/ITGA5/ITGB1/ITGB3/JAM3/LAMA2/LAMA4/LAMC1/LOXL4/MFAP5/MMP16/OLFML2A/PDGFRA/PHLDB1/PHLDB2/RECK/SFRP2/TIMP2/TNC/VCAM1/ADAM12/ADAM19/ADAMTS10/ADAMTS12/ADAMTS14/ADAMTS2/ADAMTS4/ADAMTS5/ADAMTS7/AEBP1/ANTXR1/BGN/BMP1/COL10A1/COL11A1/COL12A1/COL13A1/COL15A1/COL16A1/COL18A1/COL1A1/COL1A2/COL22A1/COL24A1/COL3A1/COL4A1/COL4A2/COL5A1/COL5A2/COL5A3/COL6A1/COL6A2/COL6A3/COL7A1/COL8A1/COL8A2/COMP/CTSK/EFEMP2/EGFLAM/ELN/EMILIN1/ENG/FAP/FBN1/FN1/HTRA1/ITGA11/KDR/LAMB2/LOX/LOXL1/LOXL2/LOXL3/LTBP3/LUM/MATN3/MFAP2/MMP14/MMP19/MMP2/NID1/NID2/NTNG2/PDGFB/PDPN/PECAM1/POSTN/PXDN/RAMP2/SERPINE1/SERPINH1/SH3PXD2B/SPARC/SULF1/THBS1/TIE1/VCAN/VWF/ADAM8/CTSL/ICAM1/ITGAM/ITGAX/ITGB2/MMP9/TGFB1 | 127 |
| BP | GO:0045229 | external encapsulating structure organization | 127/941 | 396/18862 | 2.04E-68 | 3.34E-65 | 2.40E-65 | A2M/ADAMTS1/ADAMTS16/ADAMTS3/ADAMTSL4/CAV1/CAV2/CCDC80/COL14A1/CRISPLD2/CYP1B1/DCN/DDR2/ECM2/FBLN1/FBLN2/FBLN5/FLRT2/GREM1/HSPG2/ITGA1/ITGA5/ITGB1/ITGB3/JAM3/LAMA2/LAMA4/LAMC1/LOXL4/MFAP5/MMP16/OLFML2A/PDGFRA/PHLDB1/PHLDB2/RECK/SFRP2/TIMP2/TNC/VCAM1/ADAM12/ADAM19/ADAMTS10/ADAMTS12/ADAMTS14/ADAMTS2/ADAMTS4/ADAMTS5/ADAMTS7/AEBP1/ANTXR1/BGN/BMP1/COL10A1/COL11A1/COL12A1/COL13A1/COL15A1/COL16A1/COL18A1/COL1A1/COL1A2/COL22A1/COL24A1/COL3A1/COL4A1/COL4A2/COL5A1/COL5A2/COL5A3/COL6A1/COL6A2/COL6A3/COL7A1/COL8A1/COL8A2/COMP/CTSK/EFEMP2/EGFLAM/ELN/EMILIN1/ENG/FAP/FBN1/FN1/HTRA1/ITGA11/KDR/LAMB2/LOX/LOXL1/LOXL2/LOXL3/LTBP3/LUM/MATN3/MFAP2/MMP14/MMP19/MMP2/NID1/NID2/NTNG2/PDGFB/PDPN/PECAM1/POSTN/PXDN/RAMP2/SERPINE1/SERPINH1/SH3PXD2B/SPARC/SULF1/THBS1/TIE1/VCAN/VWF/ADAM8/CTSL/ICAM1/ITGAM/ITGAX/ITGB2/MMP9/TGFB1 | 127 |
| BP | GO:0031589 | cell-substrate adhesion | 72/941 | 359/18862 | 1.28E-24 | 1.57E-21 | 1.13E-21 | ANGPT1/AXL/BCL6/CCDC80/CCL21/DLC1/ECM2/EDIL3/EPHA3/FBLN1/FBLN2/FBLN5/FERMT2/FLNA/FZD4/GREM1/ITGA1/ITGA5/ITGB1/ITGB3/JAM3/LAMC1/NRP1/PARVA/PHLDB2/SGCE/SNED1/TEK/VCAM1/VCL/ADAMTS12/ANTXR1/CD34/CDH13/COL13A1/COL16A1/COL1A1/COL3A1/COL5A3/COL8A1/EFEMP2/EGFLAM/EMILIN1/FN1/ITGA11/ITGBL1/KDR/LAMB2/LGALS1/MMP14/NID1/NID2/NTNG2/PDGFB/PDPN/PLAU/POSTN/PPM1F/SERPINE1/SPOCK1/THBS1/THSD1/THY1/VWF/CASS4/CSF1/FERMT3/ITGB2/MYO1G/PARVG/PREX1/SIGLEC1 | 72 |
| BP | GO:0030199 | collagen fibril organization | 29/941 | 54/18862 | 5.86E-24 | 5.74E-21 | 4.12E-21 | ADAMTS3/COL14A1/CYP1B1/DDR2/GREM1/LOXL4/SFRP2/ADAMTS14/ADAMTS2/AEBP1/COL11A1/COL12A1/COL13A1/COL1A1/COL1A2/COL3A1/COL5A1/COL5A2/COL5A3/COMP/EFEMP2/EMILIN1/LOX/LOXL1/LOXL2/LOXL3/LUM/PXDN/SERPINH1 | 29 |
| BP | GO:0001501 | skeletal system development | 83/941 | 486/18862 | 2.51E-23 | 2.05E-20 | 1.47E-20 | ANXA6/EFEMP1/EVC/FAT4/FGFR1/FST/GDF6/GJA1/GLI3/GREM1/LRRC17/MAF/MEF2C/MEIS1/MGP/MMP16/NKX3-2/NOTCH2/NPR2/NPR3/PDGFC/PDGFRA/PRELP/RAB23/RARB/ROR2/SFRP2/SNAI2/SOX11/SOX5/TEK/TGFB3/TMEM119/TRPS1/ADAMTS12/ADAMTS4/ADAMTS7/ALPL/BGN/BMP1/BMP8A/CDH11/CHRD/COL10A1/COL11A1/COL13A1/COL1A1/COL1A2/COL3A1/COL5A2/COMP/DCHS1/ENG/FBN1/GLI2/GPR68/LOX/LOXL2/LTBP3/LUM/MATN3/MMP14/MMP2/P3H1/PRRX1/PTHLH/SERPINH1/SFRP4/SH3PXD2B/STC1/SULF1/TRPV4/TWIST1/VCAN/CHST11/CYTL1/FGR/HAPLN3/LILRB1/MMP9/TGFB1/TYROBP/ZNF385A | 83 |
| BP | GO:0001503 | ossification | 73/941 | 401/18862 | 2.64E-22 | 1.85E-19 | 1.33E-19 | ASPN/DDR2/FAT4/FERMT2/FZD1/GLI1/GLI3/GREM1/HGF/IGFBP5/IL6ST/MEF2C/MGP/MMP16/MN1/NPR2/OMD/PDLIM7/PRKD1/ROR2/S1PR1/SFRP2/SLC24A3/SLC8A1/SNAI2/SOX11/TEK/TGFB3/TMEM119/TNC/TWIST2/VEGFC/WWTR1/ALPL/BMP1/BMP8A/CDH11/CHRD/CLEC11A/COL11A1/COL13A1/COL1A1/COL1A2/COL5A2/COL6A1/COMP/CTHRC1/DCHS1/EGR2/FSTL3/GLI2/IGFBP3/ITGA11/LOX/LTBP3/MMP14/MMP2/MRC2/PTHLH/SEMA7A/SPARC/STC1/TWIST1/VCAN/ACP5/CCR1/CLEC5A/CSF1/FGR/MMP9/P2RX7/SRGN/TGFB1 | 73 |
| BP | GO:0045765 | regulation of angiogenesis | 59/941 | 335/18862 | 1.59E-17 | 9.74E-15 | 6.99E-15 | ADAMTS1/AKT3/AQP1/ATP2B4/CYP1B1/DCN/ECSCR/FGF1/GREM1/HGF/HSPG2/ITGA5/ITGB1/PRKD1/PTGIS/PTPRM/RECK/RHOJ/SASH1/SERPINF1/SFRP2/TEK/THBS4/TNFSF12/VEGFC/ADAM12/APLNR/C3/CD34/CDH5/COL4A2/EMILIN1/ENG/FLT1/GPR4/ISM1/KDR/NPR1/PLXND1/RAMP2/SERPINE1/SPARC/SULF1/TBXA2R/THBS1/THBS2/TIE1/TWIST1/VASH1/C3AR1/C5AR1/CXCR4/CYBB/GPNMB/ITGAX/ITGB2/PIK3R6/SPHK1/STAB1 | 59 |
| BP | GO:1901342 | regulation of vasculature development | 59/941 | 341/18862 | 3.77E-17 | 1.86E-14 | 1.33E-14 | ADAMTS1/AKT3/AQP1/ATP2B4/CYP1B1/DCN/ECSCR/FGF1/GREM1/HGF/HSPG2/ITGA5/ITGB1/PRKD1/PTGIS/PTPRM/RECK/RHOJ/SASH1/SERPINF1/SFRP2/TEK/THBS4/TNFSF12/VEGFC/ADAM12/APLNR/C3/CD34/CDH5/COL4A2/EMILIN1/ENG/FLT1/GPR4/ISM1/KDR/NPR1/PLXND1/RAMP2/SERPINE1/SPARC/SULF1/TBXA2R/THBS1/THBS2/TIE1/TWIST1/VASH1/C3AR1/C5AR1/CXCR4/CYBB/GPNMB/ITGAX/ITGB2/PIK3R6/SPHK1/STAB1 | 59 |
| BP | GO:0045785 | positive regulation of cell adhesion | 67/941 | 425/18862 | 3.79E-17 | 1.86E-14 | 1.33E-14 | ANGPT1/BCL6/CAV1/CCDC80/CCL21/CYTH3/ECM2/EDIL3/FBLN2/FERMT2/FLNA/FYN/GLI3/HLX/IL6ST/ITGA5/NRP1/SFRP2/TEK/VCAM1/ADAM19/CDH13/CHRD/COL16A1/COL8A1/EFEMP2/EFNB3/EGFLAM/EMILIN1/FN1/FSTL3/GLI2/KDR/KIF26B/LGALS1/NID1/PDGFB/PDPN/PPM1F/SELE/SELP/SIRPA/THY1/TNFSF4/ADAM8/AIF1/CASS4/CCL2/CD4/CD86/CSF1/FOXP3/HAVCR2/HLA-DPB1/ICAM1/ITGB2/LILRB1/LILRB2/LILRB4/NLRP3/PDCD1LG2/PIK3R6/PREX1/PTAFR/SIRPB1/TNFSF13B/TNFSF14 | 67 |
| BP | GO:0007160 | cell-matrix adhesion | 47/941 | 230/18862 | 8.34E-17 | 3.72E-14 | 2.67E-14 | BCL6/CCL21/DLC1/ECM2/EPHA3/FBLN5/FERMT2/GREM1/ITGA1/ITGB1/ITGB3/JAM3/NRP1/PHLDB2/SGCE/SNED1/TEK/VCAM1/VCL/ADAMTS12/CD34/CDH13/COL13A1/COL16A1/COL3A1/COL5A3/EFEMP2/EMILIN1/FN1/ITGA11/ITGBL1/KDR/MMP14/NID1/NID2/PLAU/POSTN/PPM1F/SERPINE1/THBS1/THSD1/THY1/CSF1/FERMT3/ITGB2/PARVG/SIGLEC1 | 47 |
| BP | GO:0061448 | connective tissue development | 48/941 | 243/18862 | 1.62E-16 | 6.63E-14 | 4.76E-14 | ACTA2/ANXA6/EBF2/EFEMP1/EVC/GDF6/GLI3/GREM1/MAF/MEF2C/MGP/NKX3-2/RARB/ROR2/SFRP2/SNAI2/SOX5/TRPS1/ADAMTS12/ADAMTS7/BGN/BMP1/BMP8A/CD34/COL11A1/COL1A1/COL5A1/COMP/GLI2/GPR4/LOX/LOXL2/LTBP3/LUM/MATN3/PDGFB/PDGFRB/PRRX1/PTHLH/SERPINH1/SH3PXD2B/STC1/SULF1/TRPV4/CHST11/CSF1/CYTL1/TGFB1 | 48 |
| BP | GO:0032963 | collagen metabolic process | 31/941 | 109/18862 | 9.96E-16 | 3.76E-13 | 2.70E-13 | ADAMTS3/ITGB1/MMP16/TGFB3/TNS2/VIM/ADAMTS14/ADAMTS2/COL13A1/COL15A1/COL1A1/COL1A2/COL5A1/CTSK/EMILIN1/ENG/F2R/FAP/MMP14/MMP19/MMP2/MRC2/P3H1/P3H3/PDGFRB/RCN3/SERPINH1/CTSB/CTSL/MMP9/TGFB1 | 31 |
| BP | GO:0060348 | bone development | 41/941 | 193/18862 | 1.93E-15 | 6.76E-13 | 4.85E-13 | ANXA6/FAT4/GJA1/GLI3/GREM1/LRRC17/MEF2C/MEIS1/MMP16/NOTCH2/NPR2/PDGFC/RAB23/RARB/SFRP2/TEK/TGFB3/TMEM119/ALPL/BGN/COL13A1/COL1A1/COMP/DCHS1/ENG/FBN1/GPR68/LOX/LTBP3/MMP14/P3H1/SERPINH1/SFRP4/SH3PXD2B/STC1/SULF1/TRPV4/TWIST1/LILRB1/TYROBP/ZNF385A | 41 |
| BP | GO:0003158 | endothelium development | 33/941 | 137/18862 | 2.41E-14 | 7.89E-12 | 5.66E-12 | CLIC4/FGF1/GJA1/GJA5/MSN/NRP1/RDX/S1PR1/S1PR3/VCL/ZEB1/ADAMTS12/CD34/CDH5/COL15A1/COL18A1/COL22A1/ENG/FSTL1/GJA4/HEG1/KDR/NOTCH4/PDPN/PECAM1/ROBO4/SOX17/STC1/TIE1/CXCR4/ICAM1/ITGAX/S1PR2 | 33 |
| BP | GO:0010810 | regulation of cell-substrate adhesion | 42/941 | 218/18862 | 3.20E-14 | 9.80E-12 | 7.04E-12 | BCL6/CCDC80/CCL21/DLC1/ECM2/EDIL3/EPHA3/FBLN1/FBLN2/FERMT2/FLNA/FZD4/GREM1/ITGA5/NRP1/PHLDB2/TEK/VCL/CDH13/COL16A1/COL1A1/COL8A1/EFEMP2/EGFLAM/EMILIN1/FN1/KDR/LGALS1/MMP14/NID1/PDGFB/PDPN/PLAU/POSTN/PPM1F/SERPINE1/SPOCK1/THBS1/THY1/CASS4/CSF1/PREX1 | 42 |
| BP | GO:0045766 | positive regulation of angiogenesis | 37/941 | 175/18862 | 5.37E-14 | 1.46E-11 | 1.05E-11 | AKT3/AQP1/CYP1B1/FGF1/GREM1/HGF/ITGA5/ITGB1/PRKD1/PTGIS/SASH1/SFRP2/TEK/TNFSF12/VEGFC/ADAM12/APLNR/C3/CD34/CDH5/ENG/FLT1/KDR/RAMP2/SERPINE1/TBXA2R/THBS1/TIE1/TWIST1/C3AR1/C5AR1/CXCR4/CYBB/ITGAX/ITGB2/PIK3R6/SPHK1 | 37 |
| BP | GO:1904018 | positive regulation of vasculature development | 37/941 | 175/18862 | 5.37E-14 | 1.46E-11 | 1.05E-11 | AKT3/AQP1/CYP1B1/FGF1/GREM1/HGF/ITGA5/ITGB1/PRKD1/PTGIS/SASH1/SFRP2/TEK/TNFSF12/VEGFC/ADAM12/APLNR/C3/CD34/CDH5/ENG/FLT1/KDR/RAMP2/SERPINE1/TBXA2R/THBS1/TIE1/TWIST1/C3AR1/C5AR1/CXCR4/CYBB/ITGAX/ITGB2/PIK3R6/SPHK1 | 37 |
| BP | GO:0051216 | cartilage development | 38/941 | 185/18862 | 6.48E-14 | 1.67E-11 | 1.20E-11 | ANXA6/EFEMP1/EVC/GDF6/GLI3/GREM1/MAF/MEF2C/MGP/NKX3-2/RARB/ROR2/SFRP2/SNAI2/SOX5/TRPS1/ADAMTS12/ADAMTS7/BGN/BMP1/BMP8A/COL11A1/COL1A1/COMP/GLI2/LOXL2/LTBP3/LUM/MATN3/PRRX1/PTHLH/SERPINH1/STC1/SULF1/TRPV4/CHST11/CYTL1/TGFB1 | 38 |
| BP | GO:0001667 | ameboidal-type cell migration | 65/941 | 473/18862 | 9.76E-14 | 2.39E-11 | 1.72E-11 | AKAP12/AKT3/AMOTL1/ANGPT1/ANXA6/ATP2B4/CDH2/CYP1B1/DCN/DDR2/FGF1/FGF10/FGF7/FGFR1/GJA1/GREM1/HTR2B/ITGB1/ITGB3/MAP3K3/MCC/MEF2C/MEOX2/NRP1/NRP2/PRKD1/PTPRM/RHOJ/SASH1/SERPINF1/SLC8A1/SLIT2/TEK/TNFSF12/VEGFC/ZEB2/CDH13/CDH5/CLEC14A/FAP/FLT4/FN1/FSTL1/GNA12/KDR/LOXL2/MMRN2/PDGFB/PLXND1/PPM1F/SEMA6B/SEMA7A/SOX17/SPARC/STC1/SYDE1/TBXA2R/THBS1/TWIST1/VASH1/APOE/GLIPR2/ITGB2/MMP9/TGFB1 | 65 |
| BP | GO:0006023 | aminoglycan biosynthetic process | 29/941 | 114/18862 | 2.00E-13 | 4.68E-11 | 3.36E-11 | ANGPT1/CHST15/CHST3/CSGALNACT2/CSPG4/DCN/DSEL/GPC6/HSPG2/OMD/PRELP/SDC2/B3GNT9/BGN/CHST1/CHST14/CHSY3/DSE/HS3ST3A1/LUM/PDGFB/PDGFRB/VCAN/CHST11/CHST2/HS3ST2/HS3ST3B1/ST3GAL6/TGFB1 | 29 |
| BP | GO:0006024 | glycosaminoglycan biosynthetic process | 28/941 | 107/18862 | 2.43E-13 | 5.42E-11 | 3.89E-11 | ANGPT1/CHST15/CHST3/CSGALNACT2/CSPG4/DCN/DSEL/GPC6/HSPG2/OMD/PRELP/SDC2/BGN/CHST1/CHST14/CHSY3/DSE/HS3ST3A1/LUM/PDGFB/PDGFRB/VCAN/CHST11/CHST2/HS3ST2/HS3ST3B1/ST3GAL6/TGFB1 | 28 |
| BP | GO:0001819 | positive regulation of cytokine production | 61/941 | 437/18862 | 2.88E-13 | 6.13E-11 | 4.40E-11 | C1QTNF3/CADM1/CYP1B1/HGF/HTR2B/IL1R1/IL6ST/LURAP1/POLR2H/RORA/C3/CD34/F2R/FLT4/GPRC5B/HEG1/LUM/POSTN/SEMA7A/SERPINE1/SULF1/THBS1/TNFSF4/TWIST1/ADAM8/AIF1/C3AR1/C5AR1/CD14/CD4/CD86/CLEC7A/CSF1R/FCER1G/FCN1/FGR/FOXP3/GPSM3/HAVCR2/HLA-DPB1/LAPTM5/LILRA2/LILRA5/LILRB1/LILRB2/LY96/MNDA/NFAM1/NLRC4/NLRP3/P2RX7/PTAFR/RAB7B/SLC11A1/SPHK1/TGFB1/TLR1/TLR2/TLR8/TREM2/TYROBP | 61 |
| BP | GO:0002683 | negative regulation of immune system process | 57/941 | 403/18862 | 1.02E-12 | 2.08E-10 | 1.50E-10 | A2M/ANGPT1/AXL/BCL6/CCL21/GLI3/GREM1/HLX/LRRC17/SERPING1/SLIT2/SOX11/SYT11/TGFB3/TSC22D3/COL3A1/EMILIN1/FBN1/FSTL3/GPR68/HTRA1/INHBA/LOXL3/LRRC32/THBS1/THY1/TNFSF4/ZBTB46/C1QC/C5AR2/CCL2/CD300A/CD84/CD86/FCGR2B/FGR/FOXP3/GPNMB/HAVCR2/HLA-DOA/LAPTM5/LILRB1/LILRB2/LILRB3/LILRB4/LST1/MAFB/MILR1/MNDA/PDCD1LG2/SAMHD1/SAMSN1/SLAMF8/TGFB1/TNFAIP8L2/TYROBP/VSIG4 | 57 |
| BP | GO:0098742 | cell-cell adhesion via plasma-membrane adhesion molecules | 45/941 | 273/18862 | 1.30E-12 | 2.54E-10 | 1.83E-10 | CADM1/CDH2/FAT4/GPC6/HMCN1/ITGA5/ITGB1/MDGA1/NEXN/PALLD/PCDH18/PCDH7/PCDHB4/PCDHB5/PCDHB7/PCDHGA12/PCDHGA9/PCDHGB7/PCDHGC3/PTPRM/SDK1/SPARCL1/TENM3/VCAM1/CDH11/CDH13/CDH5/CDH6/DCHS1/ESAM/NTNG2/PCDH12/PCDH17/PECAM1/ROBO4/SCARF2/SELE/SELP/TENM4/TRO/CD84/ICAM1/ITGAM/ITGB2/MILR1 | 45 |
| BP | GO:0090287 | regulation of cellular response to growth factor stimulus | 47/941 | 296/18862 | 1.64E-12 | 3.10E-10 | 2.23E-10 | ADAMTS3/ASPN/ATP2B4/CAV1/CAV2/DCN/FGF1/FGF10/FGFR1/FST/FZD4/GREM1/ITGA5/ITGB3/LATS2/LTBP1/NOTCH2/SFRP2/SLIT2/SOX11/TGFB1I1/TGFB3/VEGFC/ADAMTS12/CDH5/CHRD/DKK3/DOK5/EMILIN1/ENG/FBN1/FSTL1/FSTL3/HTRA1/HTRA3/LOX/MMRN2/NREP/SFRP4/SPRED3/SULF1/THBS1/TMEM204/VASN/CHST11/NRROS/TGFB1 | 47 |
| BP | GO:0001655 | urogenital system development | 49/941 | 320/18862 | 2.19E-12 | 3.97E-10 | 2.85E-10 | ACTA2/ADAMTS1/ADAMTS16/ANGPT1/BASP1/BICC1/CYP7B1/DCN/FAT4/FGF1/FGF10/GLI1/GLI3/GREM1/KCNJ8/LRRK2/MEF2C/NOTCH2/PDGFRA/PKD2/PYGO1/RARB/SERPINF1/SLIT2/SOX11/TEK/TNC/TNS2/WWTR1/CD34/COL4A1/DCHS1/FBN1/FSTL3/GLI2/GLIS2/GPR4/HEYL/KIF26B/LAMB2/NID1/PDGFB/PDGFRB/PECAM1/PLXND1/SOX17/SULF1/MMP9/PSAP | 49 |
| BP | GO:0007159 | leukocyte cell-cell adhesion | 53/941 | 366/18862 | 2.57E-12 | 4.50E-10 | 3.23E-10 | BCL6/CAV1/CCL21/FYN/GLI3/HLX/IL6ST/ITGA5/ITGB1/MSN/VCAM1/CERCAM/EFNB3/GLI2/LGALS1/LOXL3/LRRC32/PECAM1/SELE/SELP/SIRPA/THY1/TNFSF4/ADAM8/AIF1/CCL2/CD300A/CD4/CD86/FCGR2B/FERMT3/FOXP3/GPNMB/HAVCR2/HLA-DPB1/ICAM1/ITGB2/LAPTM5/LILRB1/LILRB2/LILRB4/NLRP3/OLR1/PDCD1LG2/PIK3R6/PTAFR/S100A8/SELPLG/SIRPB1/TNFAIP8L2/TNFSF13B/TNFSF14/VSIG4 | 53 |
| BP | GO:0002237 | response to molecule of bacterial origin | 51/941 | 346/18862 | 3.40E-12 | 5.74E-10 | 4.12E-10 | AKAP12/AXL/C4B/DCN/FGF10/GJA1/KCNJ8/LDOC1/MEF2C/PTGFR/SASH1/SSC5D/VCAM1/VIM/ALPL/F2R/GGT5/LOXL1/PTGIR/SELE/SELP/SERPINE1/SIRPA/SPARC/TBXA2R/THBD/TNFSF4/C5AR1/CCL2/CD14/CD86/FCGR2B/FOXP3/HAVCR2/HCK/ICAM1/LILRA2/LILRB1/LILRB2/LY86/LY96/MRC1/NLRP3/PDCD1LG2/PTAFR/S100A8/SLC11A1/TGFB1/TLR1/TLR2/TREM2 | 51 |
| BP | GO:0050673 | epithelial cell proliferation | 58/941 | 428/18862 | 3.92E-12 | 6.41E-10 | 4.60E-10 | AKT3/CAV1/CAV2/CYP7B1/FGF1/FGF10/FGF7/FGFR1/FST/GJA1/GLI1/HGF/HTR2B/IGFBP5/ITGB3/KLF9/LAMC1/MCC/MEF2C/NOTCH2/NRP1/NRP2/PRKD1/PTPRM/SERPINF1/SFRP2/SNAI2/SOX11/TEK/THBS4/TNFSF12/VEGFC/APLNR/CD34/CDH13/COL8A1/COL8A2/FAP/FLT1/FLT4/HTRA1/IGFBP3/KDR/LOXL2/MMP14/PDGFB/SPARC/SULF1/THBS1/TIE1/TWIST1/VASH1/WNT2/APOE/C5AR1/C5AR2/CCL2/TGFB1 | 58 |
| BP | GO:0002062 | chondrocyte differentiation | 26/941 | 103/18862 | 4.28E-12 | 6.77E-10 | 4.86E-10 | ANXA6/EFEMP1/GDF6/GLI3/GREM1/MAF/MEF2C/NKX3-2/RARB/SFRP2/SNAI2/SOX5/TRPS1/ADAMTS12/ADAMTS7/COL11A1/COMP/GLI2/LOXL2/LTBP3/PTHLH/SERPINH1/SULF1/CHST11/CYTL1/TGFB1 | 26 |
| BP | GO:0018212 | peptidyl-tyrosine modification | 53/941 | 372/18862 | 4.88E-12 | 7.48E-10 | 5.37E-10 | ANGPT1/AXL/CAV1/CSPG4/DDR2/EFEMP1/EPHA3/FGF10/FGF7/FGFR1/FYN/GREM1/HGF/IL6ST/ITGA5/ITGB3/NRP1/PDGFC/PDGFRA/ROR2/SFRP2/SH3BP5/TEK/THBS4/TPST1/DLG4/FLT1/FLT4/GPRC5B/KDR/NOX4/PDGFB/PDGFRB/PECAM1/THY1/TIE1/CASS4/CD300A/CD4/CLEC7A/CSF1R/FCGR1A/FES/FGR/HCK/ICAM1/ITGB2/LILRA5/LILRB4/SAMSN1/SOCS3/TGFB1/TREM2 | 53 |
| BP | GO:0007162 | negative regulation of cell adhesion | 46/941 | 295/18862 | 5.46E-12 | 8.11E-10 | 5.82E-10 | ANGPT1/BCL6/CCL21/CYP1B1/DLC1/FBLN1/FZD4/GLI3/HLX/JAM3/PHLDB2/PLXNA4/PRKG1/RDX/SNAI2/TNC/CDH13/COL1A1/LGALS1/LOXL3/LRRC32/MMP14/NOTCH4/PLXND1/POSTN/PPM1F/SERPINE1/SPOCK1/THBS1/TNFSF4/TRPV4/CD300A/CD86/FCGR2B/FOXP3/GPNMB/HAVCR2/LAPTM5/LILRB1/LILRB2/LILRB4/PDCD1LG2/PLXNC1/TGFB1/TNFAIP8L2/VSIG4 | 46 |
| BP | GO:0060485 | mesenchyme development | 45/941 | 287/18862 | 7.67E-12 | 1.11E-09 | 7.94E-10 | ACTA2/ANXA6/BASP1/BNC2/CDH2/EPHA3/FERMT2/FGF10/FGFR1/FLNA/GREM1/HGF/HTR2B/MEF2C/NRP1/NRP2/PHLDB1/PHLDB2/PKD2/SFRP2/SNAI2/SOX11/TGFB1I1/TGFB3/WWTR1/ZFPM2/COL1A1/DCHS1/EDNRA/ENG/FN1/HEYL/LOXL2/LOXL3/NOTCH4/PDGFRB/PDPN/SEMA6B/SEMA7A/SPRED3/TWIST1/VASN/WNT2/GLIPR2/TGFB1 | 45 |
| BP | GO:0072001 | renal system development | 45/941 | 288/18862 | 8.67E-12 | 1.19E-09 | 8.52E-10 | ACTA2/ADAMTS1/ADAMTS16/ANGPT1/BASP1/BICC1/DCN/FAT4/FGF1/FGF10/GLI3/GREM1/KCNJ8/LRRK2/MEF2C/NOTCH2/PDGFRA/PKD2/PYGO1/RARB/SERPINF1/SLIT2/SOX11/TEK/TNS2/WWTR1/CD34/COL4A1/DCHS1/FBN1/FSTL3/GLI2/GLIS2/GPR4/HEYL/KIF26B/LAMB2/NID1/PDGFB/PDGFRB/PECAM1/PLXND1/SOX17/SULF1/MMP9 | 45 |
| BP | GO:0007229 | integrin-mediated signaling pathway | 26/941 | 106/18862 | 8.71E-12 | 1.19E-09 | 8.52E-10 | ADAMTS1/FERMT2/FLNA/ITGA1/ITGA5/ITGB1/ITGB3/NRP1/PRKD1/TLN1/COL16A1/COL3A1/FN1/ITGA11/ITGBL1/LOXL3/SEMA7A/THY1/FERMT3/FGR/HCK/ITGAM/ITGAX/ITGB2/PLEK/PRAM1 | 26 |
| BP | GO:0018108 | peptidyl-tyrosine phosphorylation | 52/941 | 369/18862 | 1.19E-11 | 1.57E-09 | 1.13E-09 | ANGPT1/AXL/CAV1/CSPG4/DDR2/EFEMP1/EPHA3/FGF10/FGF7/FGFR1/FYN/GREM1/HGF/IL6ST/ITGA5/ITGB3/NRP1/PDGFC/PDGFRA/ROR2/SFRP2/SH3BP5/TEK/THBS4/DLG4/FLT1/FLT4/GPRC5B/KDR/NOX4/PDGFB/PDGFRB/PECAM1/THY1/TIE1/CASS4/CD300A/CD4/CLEC7A/CSF1R/FCGR1A/FES/FGR/HCK/ICAM1/ITGB2/LILRA5/LILRB4/SAMSN1/SOCS3/TGFB1/TREM2 | 52 |
| BP | GO:0001822 | kidney development | 44/941 | 280/18862 | 1.22E-11 | 1.57E-09 | 1.13E-09 | ACTA2/ADAMTS1/ADAMTS16/ANGPT1/BASP1/BICC1/DCN/FAT4/FGF1/FGF10/GLI3/GREM1/KCNJ8/LRRK2/MEF2C/NOTCH2/PDGFRA/PKD2/PYGO1/RARB/SERPINF1/SLIT2/SOX11/TEK/TNS2/WWTR1/CD34/DCHS1/FBN1/FSTL3/GLI2/GLIS2/GPR4/HEYL/KIF26B/LAMB2/NID1/PDGFB/PDGFRB/PECAM1/PLXND1/SOX17/SULF1/MMP9 | 44 |
| BP | GO:0050866 | negative regulation of cell activation | 36/941 | 200/18862 | 1.75E-11 | 2.20E-09 | 1.58E-09 | AXL/BCL6/GLI3/HLX/PDGFRA/PRKG1/SOX11/SYT11/EMILIN1/INHBA/LOXL3/LRRC32/PDGFB/THBD/TNFSF4/APOE/CD300A/CD84/CD86/FCGR2B/FGR/FOXP3/GPNMB/HAVCR2/LAPTM5/LILRB1/LILRB2/LILRB4/LST1/MILR1/MNDA/PDCD1LG2/SAMSN1/TNFAIP8L2/TYROBP/VSIG4 | 36 |
| BP | GO:0030336 | negative regulation of cell migration | 48/941 | 330/18862 | 2.40E-11 | 2.94E-09 | 2.11E-09 | ADARB1/ATP2B4/CCL21/CLIC4/CYP1B1/DCN/DLC1/DPYSL3/GJA1/GREM1/IGFBP5/MCC/MEF2C/MEOX2/MITF/NAV3/PHLDB2/PODN/PRKG1/PTPRM/RECK/SERPINF1/SFRP2/SLIT2/SRGAP2B/SRGAP2C/VCL/CHRD/COL3A1/EMILIN1/ENG/IGFBP3/MMRN2/SERPINE1/STC1/SULF1/TBXA2R/THBS1/THY1/TIE1/VASH1/AIF1/APOE/C5AR2/CCL2/CD300A/SLAMF8/TGFB1 | 48 |
| BP | GO:0040013 | negative regulation of locomotion | 52/941 | 377/18862 | 2.68E-11 | 3.21E-09 | 2.30E-09 | ADARB1/ATP2B4/CCL21/CLIC4/CYP1B1/DCN/DLC1/DPYSL3/FBLN1/GJA1/GREM1/IGFBP5/MCC/MEF2C/MEOX2/MITF/NAV3/NRP1/PHLDB2/PODN/PRKG1/PTPRM/RECK/SERPINF1/SFRP2/SLIT2/SRGAP2B/SRGAP2C/VCL/CHRD/COL3A1/EMILIN1/ENG/IGFBP3/MMRN2/SEMA6B/SEMA7A/SERPINE1/STC1/SULF1/TBXA2R/THBS1/THY1/TIE1/VASH1/AIF1/APOE/C5AR2/CCL2/CD300A/SLAMF8/TGFB1 | 52 |
| BP | GO:0022407 | regulation of cell-cell adhesion | 57/941 | 437/18862 | 2.84E-11 | 3.26E-09 | 2.34E-09 | BCL6/CAV1/CCL21/FYN/GLI3/HLX/IL6ST/MDGA1/PRKG1/RDX/TENM3/VCAM1/ADAM19/EFNB3/FSTL3/GLI2/KIF26B/LGALS1/LOXL3/LRRC32/NOTCH4/PDPN/PPM1F/SELE/SELP/SIRPA/THY1/TNFSF4/TRPV4/ADAM8/AIF1/CCL2/CD300A/CD4/CD86/FCGR2B/FERMT3/FOXP3/GPNMB/HAVCR2/HLA-DPB1/ICAM1/ITGB2/LAPTM5/LILRB1/LILRB2/LILRB4/NLRP3/PDCD1LG2/PIK3R6/PTAFR/SIRPB1/TGFB1/TNFAIP8L2/TNFSF13B/TNFSF14/VSIG4 | 57 |
| BP | GO:0034329 | cell junction assembly | 56/941 | 425/18862 | 2.86E-11 | 3.26E-09 | 2.34E-09 | CAV1/CDH2/CNTNAP1/DLC1/EPHA3/FERMT2/FLNA/FLRT2/FZD1/GJA1/GJA5/GJC1/GPC6/GREM1/ITGA5/JAM3/LAMC1/LGI2/MAP1B/MDGA1/MEF2C/NLGN4X/NRP1/PARD6G/PCDHB4/PCDHB5/PHLDB2/SDK1/SNAI2/TEK/TLN1/VCL/CDH11/CDH5/CDH6/COL16A1/DCHS1/ESAM/FN1/GJA4/HEG1/KDR/MMP14/NLGN2/NTNG2/PCDH17/PECAM1/PLXND1/PPM1F/RAMP2/SYNDIG1/THBS1/THSD1/THY1/TRPV4/TLR2 | 56 |
| BP | GO:2000146 | negative regulation of cell motility | 49/941 | 345/18862 | 3.54E-11 | 3.94E-09 | 2.83E-09 | ADARB1/ATP2B4/CCL21/CLIC4/CYP1B1/DCN/DLC1/DPYSL3/FBLN1/GJA1/GREM1/IGFBP5/MCC/MEF2C/MEOX2/MITF/NAV3/PHLDB2/PODN/PRKG1/PTPRM/RECK/SERPINF1/SFRP2/SLIT2/SRGAP2B/SRGAP2C/VCL/CHRD/COL3A1/EMILIN1/ENG/IGFBP3/MMRN2/SERPINE1/STC1/SULF1/TBXA2R/THBS1/THY1/TIE1/VASH1/AIF1/APOE/C5AR2/CCL2/CD300A/SLAMF8/TGFB1 | 49 |
| BP | GO:0007599 | hemostasis | 49/941 | 346/18862 | 3.93E-11 | 4.28E-09 | 3.07E-09 | A2M/AXL/CAV1/EHD2/ENTPD1/F8/FBLN1/FLNA/FYN/GNG2/ITGB3/ITPR1/PDGFRA/PHF21A/PRKG1/SERPING1/TLN1/TRPC6/VCL/ZFPM2/CD34/COL1A1/COL1A2/COL3A1/COMP/EHD3/F2R/F2RL3/FAP/FN1/GNA12/PDGFB/PDPN/PLAT/PLAU/SELP/SERPINE1/TBXA2R/THBD/THBS1/VWF/APOE/FCER1G/FERMT3/LCP2/P2RX7/PIK3R6/PLEK/ZNF385A | 49 |
| BP | GO:0032496 | response to lipopolysaccharide | 47/941 | 326/18862 | 5.30E-11 | 5.65E-09 | 4.05E-09 | AKAP12/AXL/DCN/FGF10/GJA1/KCNJ8/LDOC1/MEF2C/PTGFR/SASH1/VCAM1/VIM/ALPL/F2R/GGT5/LOXL1/PTGIR/SELE/SELP/SERPINE1/SIRPA/SPARC/TBXA2R/THBD/TNFSF4/C5AR1/CCL2/CD14/CD86/FOXP3/HAVCR2/HCK/ICAM1/LILRA2/LILRB1/LILRB2/LY86/LY96/MRC1/NLRP3/PDCD1LG2/PTAFR/S100A8/SLC11A1/TGFB1/TLR2/TREM2 | 47 |
| BP | GO:0097529 | myeloid leukocyte migration | 37/941 | 218/18862 | 5.47E-11 | 5.71E-09 | 4.10E-09 | CCL21/CD99L2/GREM1/IL1R1/ITGA1/JAM3/ROR2/SLIT2/THBS4/VEGFC/EMILIN1/FLT1/MMP14/PDGFB/PECAM1/SERPINE1/SIRPA/THBS1/ADAM8/AIF1/C3AR1/C5AR1/C5AR2/CCL18/CCL2/CCR1/CD300A/CSF1/CSF1R/CSF3R/FCER1G/ITGB2/PLA2G7/PREX1/S100A8/SLAMF8/TREM2 | 37 |
| BP | GO:0008360 | regulation of cell shape | 30/941 | 151/18862 | 6.80E-11 | 6.95E-09 | 4.99E-09 | CDC42EP3/DLC1/FERMT2/FGD5/FYN/MSN/PALMD/PARVA/PLEKHO1/PLXNA4/RDX/RHOJ/RHOQ/ATP10A/FMNL3/FN1/KDR/MYH10/PDPN/PLXND1/CCL2/CSF1R/FES/FGR/FMNL1/HCK/ICAM1/ITGB2/LST1/PLXNC1 | 30 |
| BP | GO:0060326 | cell chemotaxis | 45/941 | 306/18862 | 7.01E-11 | 7.01E-09 | 5.03E-09 | CCL21/CYP7B1/FGF1/FGFR1/GREM1/HGF/ITGA1/JAM3/NRP1/PARVA/PDGFRA/PRKD1/S1PR1/SLIT2/THBS4/VCAM1/VEGFC/FLT1/KDR/LOX/PDGFB/PDGFRB/SERPINE1/THBS1/ADAM8/AIF1/C3AR1/C5AR1/C5AR2/CCL18/CCL2/CCR1/CCR8/CSF1/CSF1R/CSF3R/CXCR4/DOCK4/FCER1G/GPSM3/ITGB2/PLA2G7/PREX1/S100A8/SLAMF8 | 45 |
| BP | GO:0051271 | negative regulation of cellular component movement | 49/941 | 352/18862 | 7.29E-11 | 7.15E-09 | 5.13E-09 | ADARB1/ATP2B4/CCL21/CLIC4/CYP1B1/DCN/DLC1/DPYSL3/FBLN1/GJA1/GREM1/IGFBP5/MCC/MEF2C/MEOX2/MITF/NAV3/PHLDB2/PODN/PRKG1/PTPRM/RECK/SERPINF1/SFRP2/SLIT2/SRGAP2B/SRGAP2C/VCL/CHRD/COL3A1/EMILIN1/ENG/IGFBP3/MMRN2/SERPINE1/STC1/SULF1/TBXA2R/THBS1/THY1/TIE1/VASH1/AIF1/APOE/C5AR2/CCL2/CD300A/SLAMF8/TGFB1 | 49 |
| BP | GO:0070374 | positive regulation of ERK1 and ERK2 cascade | 36/941 | 210/18862 | 7.44E-11 | 7.15E-09 | 5.13E-09 | AKAP12/ANGPT1/CCL21/FERMT2/FGF10/HTR2B/NOTCH2/NRP1/PDGFC/PDGFRA/TEK/F2R/FLT4/GNAI2/KDR/MAP3K12/PDGFB/PDGFRB/RAMP3/SEMA7A/APOE/C5AR1/C5AR2/CCL18/CCL2/CCR1/CD4/CSF1R/GLIPR2/GPNMB/HAVCR2/ICAM1/MARCO/P2RY6/TGFB1/TREM2 | 36 |
| BP | GO:0048762 | mesenchymal cell differentiation | 38/941 | 231/18862 | 7.82E-11 | 7.37E-09 | 5.29E-09 | ANXA6/CDH2/EPHA3/FERMT2/FGF10/FGFR1/FLNA/GREM1/HGF/HTR2B/MEF2C/NRP1/NRP2/PHLDB1/PHLDB2/SFRP2/SNAI2/SOX11/TGFB1I1/TGFB3/WWTR1/COL1A1/EDNRA/ENG/FN1/HEYL/LOXL2/LOXL3/NOTCH4/PDPN/SEMA6B/SEMA7A/SPRED3/TWIST1/VASN/WNT2/GLIPR2/TGFB1 | 38 |
| BP | GO:0090130 | tissue migration | 50/941 | 365/18862 | 8.35E-11 | 7.72E-09 | 5.54E-09 | ACTA2/AKT3/ANGPT1/ATP2B4/CYP1B1/DCN/FGF1/FGF10/FGF7/FGFR1/GREM1/ITGB3/MAP3K3/MCC/MEF2C/MEOX2/NRP1/NRP2/PRKD1/PTPRM/RHOJ/SASH1/SERPINF1/SLIT2/TEK/TNFSF12/VEGFC/ZEB2/CDH13/CDH5/CLEC14A/FAP/FLT4/FSTL1/KDR/LOXL2/MMRN2/PDGFB/PLXND1/PPM1F/SPARC/STC1/TBXA2R/THBS1/VASH1/APOE/GLIPR2/ITGB2/MMP9/TGFB1 | 50 |
| BP | GO:0007596 | blood coagulation | 48/941 | 342/18862 | 8.56E-11 | 7.77E-09 | 5.58E-09 | A2M/AXL/CAV1/EHD2/ENTPD1/F8/FBLN1/FLNA/FYN/GNG2/ITGB3/ITPR1/PDGFRA/PHF21A/PRKG1/SERPING1/TLN1/TRPC6/VCL/ZFPM2/CD34/COL1A1/COL1A2/COL3A1/COMP/EHD3/F2R/F2RL3/FAP/FN1/GNA12/PDGFB/PDPN/PLAT/PLAU/SELP/SERPINE1/TBXA2R/THBD/THBS1/VWF/APOE/FCER1G/FERMT3/LCP2/P2RX7/PIK3R6/PLEK | 48 |
| BP | GO:0010631 | epithelial cell migration | 49/941 | 357/18862 | 1.21E-10 | 1.07E-08 | 7.71E-09 | AKT3/ANGPT1/ATP2B4/CYP1B1/DCN/FGF1/FGF10/FGF7/FGFR1/GREM1/ITGB3/MAP3K3/MCC/MEF2C/MEOX2/NRP1/NRP2/PRKD1/PTPRM/RHOJ/SASH1/SERPINF1/SLIT2/TEK/TNFSF12/VEGFC/ZEB2/CDH13/CDH5/CLEC14A/FAP/FLT4/FSTL1/KDR/LOXL2/MMRN2/PDGFB/PLXND1/PPM1F/SPARC/STC1/TBXA2R/THBS1/VASH1/APOE/GLIPR2/ITGB2/MMP9/TGFB1 | 49 |
| BP | GO:0050920 | regulation of chemotaxis | 37/941 | 224/18862 | 1.23E-10 | 1.08E-08 | 7.72E-09 | CCL21/FGF1/FGF10/FGFR1/GREM1/JAM3/NRP1/PDGFRA/PLXNA4/PRKD1/S1PR1/SLIT2/THBS4/VEGFC/CDH13/KDR/PDGFB/PDGFRB/PPM1F/SEMA6B/SEMA7A/SERPINE1/THBS1/AIF1/C3AR1/C5AR1/C5AR2/CCL2/CCR1/CSF1/CSF1R/CXCR4/GPSM3/PLA2G7/SLAMF8/TGFB1/TREM2 | 37 |
| BP | GO:0043542 | endothelial cell migration | 42/941 | 278/18862 | 1.28E-10 | 1.10E-08 | 7.93E-09 | AKT3/ANGPT1/ATP2B4/CYP1B1/DCN/FGF1/FGFR1/GREM1/ITGB3/MAP3K3/MEF2C/MEOX2/NRP1/NRP2/PRKD1/PTPRM/RHOJ/SASH1/SERPINF1/SLIT2/TEK/TNFSF12/VEGFC/CDH13/CDH5/CLEC14A/FAP/FLT4/FSTL1/KDR/LOXL2/MMRN2/PDGFB/PLXND1/SPARC/STC1/TBXA2R/THBS1/VASH1/APOE/ITGB2/TGFB1 | 42 |
| BP | GO:0045446 | endothelial cell differentiation | 26/941 | 119/18862 | 1.41E-10 | 1.16E-08 | 8.35E-09 | CLIC4/MSN/NRP1/RDX/S1PR1/S1PR3/VCL/ZEB1/CDH5/COL15A1/COL18A1/COL22A1/ENG/FSTL1/HEG1/KDR/NOTCH4/PDPN/PECAM1/ROBO4/SOX17/STC1/TIE1/CXCR4/ICAM1/S1PR2 | 26 |
| BP | GO:0071559 | response to transforming growth factor beta | 40/941 | 257/18862 | 1.42E-10 | 1.16E-08 | 8.35E-09 | ASPN/CAV1/CAV2/FERMT2/FYN/LATS2/LTBP1/MEF2C/NR3C1/SOX11/SOX5/TGFB1I1/TGFB3/CDH5/COL1A1/COL1A2/COL3A1/COL4A2/DKK3/EMILIN1/ENG/FBN1/FNDC4/HTRA1/HTRA3/LOX/LRRC32/LTBP2/LTBP3/MXRA5/NOX4/NREP/POSTN/SPRED3/THBS1/VASN/WNT2/CHST11/NRROS/TGFB1 | 40 |
| BP | GO:0050817 | coagulation | 48/941 | 347/18862 | 1.42E-10 | 1.16E-08 | 8.35E-09 | A2M/AXL/CAV1/EHD2/ENTPD1/F8/FBLN1/FLNA/FYN/GNG2/ITGB3/ITPR1/PDGFRA/PHF21A/PRKG1/SERPING1/TLN1/TRPC6/VCL/ZFPM2/CD34/COL1A1/COL1A2/COL3A1/COMP/EHD3/F2R/F2RL3/FAP/FN1/GNA12/PDGFB/PDPN/PLAT/PLAU/SELP/SERPINE1/TBXA2R/THBD/THBS1/VWF/APOE/FCER1G/FERMT3/LCP2/P2RX7/PIK3R6/PLEK | 48 |
| BP | GO:0035987 | endodermal cell differentiation | 16/941 | 44/18862 | 1.45E-10 | 1.16E-08 | 8.35E-09 | ITGA5/COL11A1/COL12A1/COL4A2/COL5A1/COL5A2/COL6A1/COL7A1/COL8A1/FN1/INHBA/MMP14/MMP2/SOX17/ITGB2/MMP9 | 16 |
| BP | GO:0048771 | tissue remodeling | 32/941 | 175/18862 | 1.53E-10 | 1.21E-08 | 8.66E-09 | AXL/CAV1/CSPG4/DDR2/FGF10/FLNA/GJA1/GREM1/IGFBP5/ITGB3/MEF2C/MITF/NOTCH2/RSPO3/S1PR1/TGFB3/THBS4/TMEM119/BGN/CTHRC1/F2R/FLT4/LTBP3/MMP14/MMP2/TIE1/ACP5/ADAM8/CSF1R/GPNMB/P2RX7/TGFB1 | 32 |
| BP | GO:0090132 | epithelium migration | 49/941 | 360/18862 | 1.62E-10 | 1.26E-08 | 9.06E-09 | AKT3/ANGPT1/ATP2B4/CYP1B1/DCN/FGF1/FGF10/FGF7/FGFR1/GREM1/ITGB3/MAP3K3/MCC/MEF2C/MEOX2/NRP1/NRP2/PRKD1/PTPRM/RHOJ/SASH1/SERPINF1/SLIT2/TEK/TNFSF12/VEGFC/ZEB2/CDH13/CDH5/CLEC14A/FAP/FLT4/FSTL1/KDR/LOXL2/MMRN2/PDGFB/PLXND1/PPM1F/SPARC/STC1/TBXA2R/THBS1/VASH1/APOE/GLIPR2/ITGB2/MMP9/TGFB1 | 49 |
| BP | GO:0030168 | platelet activation | 30/941 | 157/18862 | 1.86E-10 | 1.43E-08 | 1.03E-08 | AXL/FLNA/FYN/GNG2/ITGB3/ITPR1/PDGFRA/PRKG1/TLN1/TRPC6/VCL/COL1A1/COL1A2/COL3A1/COMP/F2R/F2RL3/FN1/GNA12/PDGFB/PDPN/SELP/THBD/VWF/APOE/FCER1G/FERMT3/LCP2/PIK3R6/PLEK | 30 |
| BP | GO:0007156 | homophilic cell adhesion via plasma membrane adhesion molecules | 31/941 | 167/18862 | 1.99E-10 | 1.50E-08 | 1.08E-08 | CADM1/CDH2/FAT4/HMCN1/ITGB1/NEXN/PALLD/PCDH18/PCDH7/PCDHB4/PCDHB5/PCDHB7/PCDHGA12/PCDHGA9/PCDHGB7/PCDHGC3/PTPRM/SDK1/TENM3/CDH11/CDH13/CDH5/CDH6/DCHS1/ESAM/PCDH12/PCDH17/PECAM1/ROBO4/TRO/CD84 | 31 |
| BP | GO:0030203 | glycosaminoglycan metabolic process | 30/941 | 158/18862 | 2.19E-10 | 1.63E-08 | 1.17E-08 | ANGPT1/CHST15/CHST3/CSGALNACT2/CSPG4/DCN/DSEL/GPC6/HGF/HSPG2/OMD/PRELP/SDC2/BGN/CHST1/CHST14/CHSY3/DSE/EGFLAM/HS3ST3A1/LUM/PDGFB/PDGFRB/VCAN/CHST11/CHST2/HS3ST2/HS3ST3B1/ST3GAL6/TGFB1 | 30 |
| BP | GO:0045216 | cell-cell junction organization | 35/941 | 209/18862 | 2.65E-10 | 1.94E-08 | 1.39E-08 | CADM1/CAV1/CDH2/CNTNAP1/FERMT2/FLNA/GJA1/GJA5/GJC1/JAM3/NLGN4X/PARD6G/RDX/SDK1/SNAI2/TGFB3/TLN1/VCL/CDH11/CDH13/CDH5/CDH6/DCHS1/ESAM/F2R/GJA4/HEG1/KIFC3/NLGN2/NUMBL/PECAM1/RAMP2/TRPV4/CSF1R/TGFB1 | 35 |
| BP | GO:0001935 | endothelial cell proliferation | 33/941 | 191/18862 | 3.72E-10 | 2.67E-08 | 1.92E-08 | AKT3/CAV1/CAV2/FGFR1/GJA1/HTR2B/ITGB3/MEF2C/NRP1/NRP2/PRKD1/PTPRM/TEK/THBS4/TNFSF12/VEGFC/APLNR/CD34/CDH13/FLT1/FLT4/KDR/LOXL2/MMP14/PDGFB/SPARC/SULF1/THBS1/TIE1/VASH1/WNT2/APOE/CCL2 | 33 |
| BP | GO:0001706 | endoderm formation | 17/941 | 53/18862 | 3.76E-10 | 2.67E-08 | 1.92E-08 | ITGA5/SOX7/COL11A1/COL12A1/COL4A2/COL5A1/COL5A2/COL6A1/COL7A1/COL8A1/FN1/INHBA/MMP14/MMP2/SOX17/ITGB2/MMP9 | 17 |
| BP | GO:0006022 | aminoglycan metabolic process | 31/941 | 172/18862 | 4.29E-10 | 3.01E-08 | 2.16E-08 | ANGPT1/CHST15/CHST3/CSGALNACT2/CSPG4/DCN/DSEL/GPC6/HGF/HSPG2/OMD/PRELP/SDC2/B3GNT9/BGN/CHST1/CHST14/CHSY3/DSE/EGFLAM/HS3ST3A1/LUM/PDGFB/PDGFRB/VCAN/CHST11/CHST2/HS3ST2/HS3ST3B1/ST3GAL6/TGFB1 | 31 |
| BP | GO:0048705 | skeletal system morphogenesis | 35/941 | 213/18862 | 4.51E-10 | 3.05E-08 | 2.19E-08 | ANXA6/FGFR1/GLI3/GREM1/MEF2C/MGP/MMP16/NKX3-2/PDGFRA/RAB23/RARB/ROR2/SFRP2/SOX11/SOX5/TEK/TGFB3/TMEM119/ALPL/BMP1/COL11A1/COL13A1/COL1A1/COMP/LTBP3/MMP14/MMP2/PRRX1/SERPINH1/SFRP4/STC1/TRPV4/TWIST1/CHST11/FGR | 35 |
| BP | GO:0030574 | collagen catabolic process | 16/941 | 47/18862 | 4.54E-10 | 3.05E-08 | 2.19E-08 | ADAMTS3/ITGB1/MMP16/ADAMTS14/ADAMTS2/COL13A1/COL15A1/CTSK/FAP/MMP14/MMP19/MMP2/MRC2/CTSB/CTSL/MMP9 | 16 |
| BP | GO:0003018 | vascular process in circulatory system | 38/941 | 245/18862 | 4.54E-10 | 3.05E-08 | 2.19E-08 | ABCC9/ACTA2/AKAP12/ANGPT1/ATP2B4/AVPR1A/CAV1/FERMT2/GJA1/GJA5/HTR2B/ITGA1/KCNJ8/PRKG1/RGS2/SLC16A2/SLC24A3/SLC8A1/SLIT2/TEK/CDH5/COMP/EDNRA/F2R/GPR4/NPR1/RAMP2/SLC2A3/SLC6A1/TBXA2R/TRPV4/APOE/DOCK4/HRH2/ICAM1/PTAFR/SLC1A3/TGFB1 | 38 |
| BP | GO:0150063 | visual system development | 48/941 | 363/18862 | 6.74E-10 | 4.46E-08 | 3.20E-08 | ATP2B4/CLIC4/CYP1B1/EFEMP1/FGF10/FZD4/GLI3/MAF/MEIS1/MFAP5/MITF/NOTCH2/NRP1/PBX3/PDGFRA/PRPH2/PTPRM/RARB/RHOJ/SDK1/SERPINF1/SMARCD3/SOX11/TENM3/TUB/VIM/ALDH1A3/ARHGEF15/C3/COL4A1/COL5A1/COL5A2/COL8A1/COL8A2/FBN1/FLT1/INHBA/LAMB2/MEIS3/MFAP2/PDGFRB/SH3PXD2B/SPRED3/THY1/TWIST1/WNT2/C1QA/ITGAM | 48 |
| BP | GO:0022604 | regulation of cell morphogenesis | 43/941 | 305/18862 | 7.19E-10 | 4.70E-08 | 3.37E-08 | CDC42EP3/DLC1/FBLN1/FERMT2/FGD5/FLNA/FYN/FZD4/MSN/NRP1/PALMD/PARVA/PLEKHO1/PLXNA4/RDX/RHOJ/RHOQ/ZNF135/ATP10A/DLG4/FMNL3/FN1/KDR/MYH10/NTNG2/PDPN/PLXND1/POSTN/SPARC/WTIP/CASS4/CCL2/CSF1R/CXCR4/FES/FGR/FMNL1/HCK/ICAM1/ITGB2/LST1/PLXNC1/PREX1 | 43 |
| BP | GO:0002576 | platelet degranulation | 26/941 | 128/18862 | 7.63E-10 | 4.92E-08 | 3.53E-08 | A2M/F8/FLNA/HGF/ITGB3/PCDH7/SERPING1/TGFB3/TLN1/VCL/VEGFC/FN1/ISLR/PDGFB/PECAM1/SELP/SERPINE1/SPARC/THBS1/TIMP3/VWF/FERMT3/PLEK/PSAP/SRGN/TGFB1 | 26 |
| BP | GO:0045123 | cellular extravasation | 19/941 | 70/18862 | 8.65E-10 | 5.51E-08 | 3.95E-08 | CCL21/CD99L2/IL1R1/ITGA1/ITGB1/JAM3/VCAM1/PECAM1/PLVAP/SELE/SELP/SIRPA/THY1/ADAM8/CCL2/ICAM1/ITGB2/PTAFR/SELPLG | 19 |
| BP | GO:0032330 | regulation of chondrocyte differentiation | 16/941 | 49/18862 | 9.19E-10 | 5.72E-08 | 4.10E-08 | EFEMP1/GDF6/GLI3/GREM1/MAF/NKX3-2/RARB/SNAI2/SOX5/TRPS1/ADAMTS12/ADAMTS7/GLI2/LOXL2/LTBP3/PTHLH | 16 |
| BP | GO:0071560 | cellular response to transforming growth factor beta stimulus | 38/941 | 251/18862 | 9.22E-10 | 5.72E-08 | 4.10E-08 | ASPN/CAV1/CAV2/FERMT2/FYN/LATS2/LTBP1/MEF2C/NR3C1/SOX11/SOX5/TGFB1I1/TGFB3/CDH5/COL1A1/COL1A2/COL3A1/COL4A2/DKK3/EMILIN1/ENG/FBN1/HTRA1/HTRA3/LOX/LRRC32/LTBP2/LTBP3/NOX4/NREP/POSTN/SPRED3/THBS1/VASN/WNT2/CHST11/NRROS/TGFB1 | 38 |
| BP | GO:0031532 | actin cytoskeleton reorganization | 23/941 | 103/18862 | 1.03E-09 | 6.23E-08 | 4.47E-08 | EFS/FGF10/FGF7/FLNA/NOTCH2/NRP1/PARVA/PDGFRA/S1PR1/TEK/THSD7A/ANTXR1/ESAM/SYDE1/TRPV4/CASS4/CSF1R/FES/GMFG/HCK/PARVG/PLEK/S1PR2 | 23 |
| BP | GO:0050921 | positive regulation of chemotaxis | 27/941 | 139/18862 | 1.03E-09 | 6.23E-08 | 4.47E-08 | CCL21/FGF10/FGFR1/NRP1/PRKD1/S1PR1/SLIT2/THBS4/VEGFC/CDH13/KDR/PDGFB/PDGFRB/PPM1F/SERPINE1/THBS1/AIF1/C3AR1/C5AR1/CCR1/CSF1/CSF1R/CXCR4/GPSM3/PLA2G7/TGFB1/TREM2 | 27 |
| BP | GO:0010811 | positive regulation of cell-substrate adhesion | 25/941 | 121/18862 | 1.11E-09 | 6.63E-08 | 4.76E-08 | CCDC80/CCL21/ECM2/EDIL3/FBLN2/FERMT2/FLNA/ITGA5/NRP1/TEK/CDH13/COL16A1/COL8A1/EFEMP2/EGFLAM/EMILIN1/FN1/KDR/NID1/PDGFB/PPM1F/THY1/CASS4/CSF1/PREX1 | 25 |
| BP | GO:0048880 | sensory system development | 48/941 | 369/18862 | 1.17E-09 | 6.93E-08 | 4.97E-08 | ATP2B4/CLIC4/CYP1B1/EFEMP1/FGF10/FZD4/GLI3/MAF/MEIS1/MFAP5/MITF/NOTCH2/NRP1/PBX3/PDGFRA/PRPH2/PTPRM/RARB/RHOJ/SDK1/SERPINF1/SMARCD3/SOX11/TENM3/TUB/VIM/ALDH1A3/ARHGEF15/C3/COL4A1/COL5A1/COL5A2/COL8A1/COL8A2/FBN1/FLT1/INHBA/LAMB2/MEIS3/MFAP2/PDGFRB/SH3PXD2B/SPRED3/THY1/TWIST1/WNT2/C1QA/ITGAM | 48 |
| BP | GO:0022409 | positive regulation of cell-cell adhesion | 40/941 | 276/18862 | 1.25E-09 | 7.28E-08 | 5.22E-08 | BCL6/CAV1/CCL21/FYN/GLI3/HLX/IL6ST/VCAM1/ADAM19/EFNB3/FSTL3/GLI2/KIF26B/LGALS1/PDPN/SELE/SELP/SIRPA/THY1/TNFSF4/ADAM8/AIF1/CCL2/CD4/CD86/FOXP3/HAVCR2/HLA-DPB1/ICAM1/ITGB2/LILRB1/LILRB2/LILRB4/NLRP3/PDCD1LG2/PIK3R6/PTAFR/SIRPB1/TNFSF13B/TNFSF14 | 40 |
| BP | GO:0070372 | regulation of ERK1 and ERK2 cascade | 42/941 | 301/18862 | 1.56E-09 | 9.00E-08 | 6.46E-08 | AKAP12/ANGPT1/CCL21/FBLN1/FERMT2/FGF10/HTR2B/NOTCH2/NRP1/PDGFC/PDGFRA/TEK/EMILIN1/F2R/FLT4/FN1/GNAI2/KDR/MAP3K12/PDGFB/PDGFRB/RAMP3/SEMA7A/SIRPA/SPRED3/TIMP3/APOE/C5AR1/C5AR2/CCL18/CCL2/CCR1/CD4/CSF1R/GLIPR2/GPNMB/HAVCR2/ICAM1/MARCO/P2RY6/TGFB1/TREM2 | 42 |
| BP | GO:0001649 | osteoblast differentiation | 35/941 | 223/18862 | 1.60E-09 | 9.12E-08 | 6.55E-08 | DDR2/FERMT2/FZD1/GLI1/GLI3/GREM1/HGF/IGFBP5/IL6ST/MEF2C/PDLIM7/PRKD1/SFRP2/SNAI2/SOX11/TMEM119/TNC/TWIST2/VEGFC/WWTR1/ALPL/CHRD/COL1A1/COL6A1/CTHRC1/GLI2/IGFBP3/ITGA11/LOX/MRC2/PTHLH/SEMA7A/TWIST1/VCAN/CLEC5A | 35 |
| BP | GO:1903039 | positive regulation of leukocyte cell-cell adhesion | 36/941 | 234/18862 | 1.63E-09 | 9.21E-08 | 6.61E-08 | BCL6/CAV1/CCL21/FYN/GLI3/HLX/IL6ST/VCAM1/EFNB3/GLI2/LGALS1/SELE/SELP/SIRPA/THY1/TNFSF4/ADAM8/AIF1/CCL2/CD4/CD86/FOXP3/HAVCR2/HLA-DPB1/ICAM1/ITGB2/LILRB1/LILRB2/LILRB4/NLRP3/PDCD1LG2/PIK3R6/PTAFR/SIRPB1/TNFSF13B/TNFSF14 | 36 |
| BP | GO:0050678 | regulation of epithelial cell proliferation | 48/941 | 374/18862 | 1.84E-09 | 1.03E-07 | 7.36E-08 | AKT3/CAV1/CAV2/CYP7B1/FGF1/FGF10/FGF7/FGFR1/GJA1/GLI1/HTR2B/ITGB3/KLF9/LAMC1/MCC/MEF2C/NOTCH2/NRP1/NRP2/PRKD1/PTPRM/SERPINF1/SFRP2/SNAI2/SOX11/TEK/THBS4/TNFSF12/VEGFC/APLNR/CDH13/FLT1/FLT4/HTRA1/KDR/PDGFB/SPARC/SULF1/THBS1/TIE1/TWIST1/VASH1/WNT2/APOE/C5AR1/C5AR2/CCL2/TGFB1 | 48 |
| BP | GO:0042119 | neutrophil activation | 58/941 | 500/18862 | 1.89E-09 | 1.04E-07 | 7.47E-08 | CRISPLD2/STOM/TIMP2/VCL/C3/CD93/PECAM1/PLAU/RAB31/SIRPA/SLC2A3/TNFAIP6/ADAM8/C3AR1/C5AR1/CD14/CD300A/CD33/CD53/CLEC5A/CTSB/CYBB/DOK3/FCER1G/FCGR2A/FCGR2B/FCN1/FGR/FPR1/GMFG/GPR84/HK3/ITGAM/ITGAX/ITGB2/LAIR1/LILRA2/LILRB2/LILRB3/MCEMP1/MMP9/MNDA/NFAM1/OLR1/OSCAR/PLEKHO2/PRAM1/PREX1/PSAP/PTAFR/RNASE2/S100A8/SIGLEC14/SIGLEC9/SIRPB1/SLC11A1/TLR2/TYROBP | 58 |
| BP | GO:0022617 | extracellular matrix disassembly | 20/941 | 81/18862 | 1.94E-09 | 1.06E-07 | 7.58E-08 | A2M/DDR2/LAMC1/MMP16/TIMP2/ADAMTS4/ADAMTS5/BMP1/CTSK/FAP/HTRA1/MMP14/MMP19/MMP2/PDPN/SH3PXD2B/ADAM8/CTSL/MMP9/TGFB1 | 20 |
| BP | GO:0085029 | extracellular matrix assembly | 15/941 | 45/18862 | 2.23E-09 | 1.20E-07 | 8.62E-08 | FBLN5/PHLDB1/PHLDB2/ANTXR1/COL1A2/EFEMP2/EMILIN1/LAMB2/LOX/LTBP3/NTNG2/PXDN/RAMP2/TIE1/TGFB1 | 15 |
| BP | GO:0060349 | bone morphogenesis | 21/941 | 90/18862 | 2.33E-09 | 1.24E-07 | 8.93E-08 | ANXA6/GLI3/MEF2C/MMP16/RAB23/RARB/SFRP2/TEK/TGFB3/TMEM119/ALPL/COL13A1/COL1A1/COMP/LTBP3/MMP14/SERPINH1/SFRP4/STC1/TRPV4/TWIST1 | 21 |
| BP | GO:0002685 | regulation of leukocyte migration | 33/941 | 205/18862 | 2.43E-09 | 1.28E-07 | 9.18E-08 | CCL21/CD99L2/GREM1/IL1R1/JAM3/MSN/SLIT2/THBS4/VEGFC/EMILIN1/MMP14/PLVAP/SELE/SELP/SERPINE1/THBS1/THY1/ADAM8/AIF1/C3AR1/C5AR1/C5AR2/CCL2/CCR1/CD300A/CSF1/CSF1R/GPSM3/ICAM1/PLA2G7/PTAFR/SLAMF8/TREM2 | 33 |
| BP | GO:1903037 | regulation of leukocyte cell-cell adhesion | 44/941 | 330/18862 | 2.69E-09 | 1.40E-07 | 1.01E-07 | BCL6/CAV1/CCL21/FYN/GLI3/HLX/IL6ST/VCAM1/EFNB3/GLI2/LGALS1/LOXL3/LRRC32/SELE/SELP/SIRPA/THY1/TNFSF4/ADAM8/AIF1/CCL2/CD300A/CD4/CD86/FCGR2B/FOXP3/GPNMB/HAVCR2/HLA-DPB1/ICAM1/ITGB2/LAPTM5/LILRB1/LILRB2/LILRB4/NLRP3/PDCD1LG2/PIK3R6/PTAFR/SIRPB1/TNFAIP8L2/TNFSF13B/TNFSF14/VSIG4 | 44 |
| BP | GO:0001763 | morphogenesis of a branching structure | 31/941 | 186/18862 | 3.13E-09 | 1.61E-07 | 1.16E-07 | ADAMTS16/CLIC4/FAT4/FGF1/FGF10/FGF7/GLI3/GREM1/HGF/LRRK2/NRP1/PKD2/RSPO3/SFRP2/SLIT2/TNC/COL13A1/COL4A1/DCHS1/EDNRA/ENG/GLI2/KDR/MMP14/NOTCH4/PLXND1/SULF1/TIE1/WNT2/CSF1/SOCS3 | 31 |
| BP | GO:0001936 | regulation of endothelial cell proliferation | 30/941 | 177/18862 | 3.73E-09 | 1.90E-07 | 1.37E-07 | AKT3/CAV1/CAV2/FGFR1/GJA1/HTR2B/ITGB3/MEF2C/NRP1/NRP2/PRKD1/PTPRM/TEK/THBS4/TNFSF12/VEGFC/APLNR/CDH13/FLT1/FLT4/KDR/PDGFB/SPARC/SULF1/THBS1/TIE1/VASH1/WNT2/APOE/CCL2 | 30 |
| BP | GO:0007178 | transmembrane receptor protein serine/threonine kinase signaling pathway | 46/941 | 358/18862 | 3.87E-09 | 1.96E-07 | 1.40E-07 | ASPN/CAV1/CAV2/FERMT2/FGF10/FST/GDF6/GREM1/LATS2/LTBP1/NOTCH2/ROR2/SFRP2/SOX11/TGFB1I1/TGFB3/VIM/WWTR1/BMP8A/CDH5/CHRD/COL1A2/COL3A1/COMP/DKK3/EMILIN1/ENG/FBN1/FSTL1/FSTL3/HTRA1/HTRA3/INHBA/LOX/LRRC32/LTBP2/LTBP3/NREP/SFRP4/SPRED3/SULF1/THBS1/VASN/CHST11/NRROS/TGFB1 | 46 |
| BP | GO:0002283 | neutrophil activation involved in immune response | 56/941 | 488/18862 | 5.34E-09 | 2.67E-07 | 1.92E-07 | CRISPLD2/STOM/TIMP2/VCL/C3/CD93/PECAM1/PLAU/RAB31/SIRPA/SLC2A3/TNFAIP6/ADAM8/C3AR1/C5AR1/CD14/CD300A/CD33/CD53/CLEC5A/CTSB/CYBB/DOK3/FCER1G/FCGR2A/FCN1/FGR/FPR1/GMFG/GPR84/HK3/ITGAM/ITGAX/ITGB2/LAIR1/LILRA2/LILRB2/LILRB3/MCEMP1/MMP9/MNDA/NFAM1/OLR1/OSCAR/PLEKHO2/PRAM1/PSAP/PTAFR/RNASE2/S100A8/SIGLEC14/SIGLEC9/SIRPB1/SLC11A1/TLR2/TYROBP | 56 |
| BP | GO:0032835 | glomerulus development | 17/941 | 62/18862 | 5.63E-09 | 2.76E-07 | 1.98E-07 | ACTA2/ANGPT1/BASP1/MEF2C/NOTCH2/PDGFRA/TEK/WWTR1/CD34/GPR4/HEYL/LAMB2/NID1/PDGFB/PDGFRB/PECAM1/SULF1 | 17 |
| BP | GO:0050867 | positive regulation of cell activation | 50/941 | 412/18862 | 5.69E-09 | 2.76E-07 | 1.98E-07 | AXL/BCL6/CAV1/CCL21/FGF10/FYN/GLI3/HLX/IL6ST/LRRK2/MEF2C/VCAM1/EFNB3/GLI2/LGALS1/MMP14/PDGFRB/SELP/SIRPA/THBS1/THY1/TNFSF4/ADAM8/AIF1/CCL2/CD4/CD86/CLEC7A/FGR/FOXP3/HAVCR2/HLA-DPB1/ITGAM/ITGB2/LILRA2/LILRA5/LILRB1/LILRB2/LILRB4/NLRP3/PDCD1LG2/PIK3R6/PLEK/PTAFR/SIRPB1/TGFB1/TNFSF13B/TNFSF14/TREM2/TYROBP | 50 |
| BP | GO:1902105 | regulation of leukocyte differentiation | 39/941 | 279/18862 | 5.69E-09 | 2.76E-07 | 1.98E-07 | AXL/BCL6/GLI3/HLX/LRRC17/MITF/NOTCH2/ROR2/FBN1/FSTL3/GLI2/GPR68/INHBA/LOXL3/MMP14/TNFSF4/ZBTB46/ADAM8/C1QC/CCR1/CD4/CD86/CSF1/FCGR2B/FES/FOXP3/HLA-DOA/LILRB1/LILRB2/LILRB3/LILRB4/MAFB/NFAM1/NLRP3/PIK3R6/SLAMF8/TGFB1/TREM2/TYROBP | 39 |
| BP | GO:0030282 | bone mineralization | 23/941 | 113/18862 | 6.93E-09 | 3.33E-07 | 2.39E-07 | ASPN/DDR2/GREM1/MEF2C/MGP/OMD/ROR2/S1PR1/SLC24A3/SLC8A1/TGFB3/TMEM119/COL1A2/COMP/LOX/LTBP3/PTHLH/TWIST1/CCR1/FGR/P2RX7/SRGN/TGFB1 | 23 |
| BP | GO:0002695 | negative regulation of leukocyte activation | 30/941 | 182/18862 | 7.31E-09 | 3.48E-07 | 2.50E-07 | AXL/BCL6/GLI3/HLX/SOX11/SYT11/INHBA/LOXL3/LRRC32/TNFSF4/CD300A/CD84/CD86/FCGR2B/FGR/FOXP3/GPNMB/HAVCR2/LAPTM5/LILRB1/LILRB2/LILRB4/LST1/MILR1/MNDA/PDCD1LG2/SAMSN1/TNFAIP8L2/TYROBP/VSIG4 | 30 |
| BP | GO:0110148 | biomineralization | 28/941 | 162/18862 | 7.88E-09 | 3.71E-07 | 2.67E-07 | ASPN/DDR2/GREM1/MEF2C/MGP/OMD/ROR2/S1PR1/SLC24A3/SLC8A1/TGFB3/TMEM119/ALPL/COL1A1/COL1A2/COMP/FAM20C/FBLN7/LOX/LTBP3/PTHLH/TWIST1/CCR1/FAM20A/FGR/P2RX7/SRGN/TGFB1 | 28 |
| BP | GO:0071711 | basement membrane organization | 12/941 | 30/18862 | 8.33E-09 | 3.89E-07 | 2.79E-07 | CAV1/CAV2/FLRT2/PHLDB1/PHLDB2/COL4A1/LAMB2/NID1/NID2/NTNG2/PXDN/RAMP2 | 12 |
| BP | GO:0061138 | morphogenesis of a branching epithelium | 29/941 | 173/18862 | 8.77E-09 | 4.06E-07 | 2.91E-07 | ADAMTS16/CLIC4/FAT4/FGF1/FGF10/FGF7/GLI3/GREM1/HGF/NRP1/PKD2/RSPO3/SFRP2/SLIT2/TNC/COL4A1/DCHS1/EDNRA/ENG/GLI2/KDR/MMP14/NOTCH4/PLXND1/SULF1/TIE1/WNT2/CSF1/SOCS3 | 29 |
| BP | GO:0051250 | negative regulation of lymphocyte activation | 27/941 | 153/18862 | 9.16E-09 | 4.17E-07 | 2.99E-07 | AXL/BCL6/GLI3/HLX/SOX11/INHBA/LOXL3/LRRC32/TNFSF4/CD300A/CD86/FCGR2B/FGR/FOXP3/GPNMB/HAVCR2/LAPTM5/LILRB1/LILRB2/LILRB4/LST1/MNDA/PDCD1LG2/SAMSN1/TNFAIP8L2/TYROBP/VSIG4 | 27 |
| BP | GO:0051924 | regulation of calcium ion transport | 35/941 | 238/18862 | 9.18E-09 | 4.17E-07 | 2.99E-07 | CACNA2D1/CAV1/FYN/GEM/GJA1/PKD2/REM1/RGS4/SLC8A1/TRPC1/TRPC6/APLNR/EHD3/F2R/F2RL3/GNAI2/PDGFB/PDGFRB/RAMP3/STC1/THY1/CCL2/CCR1/CD33/CD4/CD84/CXCR4/ICAM1/LILRA2/LILRA5/LILRB1/LILRB2/P2RX7/P2RY6/TRPV2 | 35 |
| BP | GO:1903510 | mucopolysaccharide metabolic process | 23/941 | 115/18862 | 9.87E-09 | 4.40E-07 | 3.16E-07 | ANGPT1/CHST15/CHST3/CSGALNACT2/CSPG4/DCN/DSEL/HGF/OMD/PRELP/BGN/CHST1/CHST14/CHSY3/DSE/EGFLAM/LUM/PDGFB/VCAN/CHST11/CHST2/ST3GAL6/TGFB1 | 23 |
| BP | GO:0070371 | ERK1 and ERK2 cascade | 42/941 | 320/18862 | 9.88E-09 | 4.40E-07 | 3.16E-07 | AKAP12/ANGPT1/CCL21/FBLN1/FERMT2/FGF10/HTR2B/NOTCH2/NRP1/PDGFC/PDGFRA/TEK/EMILIN1/F2R/FLT4/FN1/GNAI2/KDR/MAP3K12/PDGFB/PDGFRB/RAMP3/SEMA7A/SIRPA/SPRED3/TIMP3/APOE/C5AR1/C5AR2/CCL18/CCL2/CCR1/CD4/CSF1R/GLIPR2/GPNMB/HAVCR2/ICAM1/MARCO/P2RY6/TGFB1/TREM2 | 42 |
| BP | GO:0003007 | heart morphogenesis | 35/941 | 239/18862 | 1.02E-08 | 4.53E-07 | 3.25E-07 | ADAMTS1/DLC1/FAT4/FLNA/FLRT2/GJA5/HTR2B/MEF2C/NOTCH2/NRP1/NRP2/PARVA/PKD2/RARB/S1PR1/SFRP2/SLIT2/SMARCD3/SNAI2/SOX11/TEK/ZFPM2/ALPK2/COL11A1/COL5A1/DCHS1/ELN/ENG/HEG1/HEYL/PLXND1/SOX17/TWIST1/WNT2/TGFB1 | 35 |
| BP | GO:0090092 | regulation of transmembrane receptor protein serine/threonine kinase signaling pathway | 36/941 | 251/18862 | 1.11E-08 | 4.83E-07 | 3.47E-07 | ASPN/CAV1/CAV2/FGF10/FST/GDF6/GREM1/LATS2/LTBP1/NOTCH2/SFRP2/SOX11/TGFB1I1/TGFB3/BMP8A/CDH5/CHRD/DKK3/EMILIN1/ENG/FBN1/FSTL1/FSTL3/HTRA1/HTRA3/INHBA/LOX/NREP/SFRP4/SPRED3/SULF1/THBS1/VASN/CHST11/NRROS/TGFB1 | 36 |
| BP | GO:0043312 | neutrophil degranulation | 55/941 | 485/18862 | 1.11E-08 | 4.83E-07 | 3.47E-07 | CRISPLD2/STOM/TIMP2/VCL/C3/CD93/PECAM1/PLAU/RAB31/SIRPA/SLC2A3/TNFAIP6/ADAM8/C3AR1/C5AR1/CD14/CD300A/CD33/CD53/CLEC5A/CTSB/CYBB/DOK3/FCER1G/FCGR2A/FCN1/FGR/FPR1/GMFG/GPR84/HK3/ITGAM/ITGAX/ITGB2/LAIR1/LILRB2/LILRB3/MCEMP1/MMP9/MNDA/NFAM1/OLR1/OSCAR/PLEKHO2/PRAM1/PSAP/PTAFR/RNASE2/S100A8/SIGLEC14/SIGLEC9/SIRPB1/SLC11A1/TLR2/TYROBP | 55 |
| BP | GO:0007204 | positive regulation of cytosolic calcium ion concentration | 41/941 | 310/18862 | 1.19E-08 | 5.10E-07 | 3.66E-07 | ATP2B4/AVPR1A/CACNA2D1/CAV1/CCL21/FYN/GJA1/HTR2B/ITPR1/PDGFRA/PKD2/PTGER3/PTGFR/S1PR1/S1PR3/SLC8A1/TRPC1/TRPC6/APLNR/DLG4/EDNRA/F2R/F2RL3/GPR4/PTGIR/RAMP3/TBXA2R/THY1/TRPV4/C3AR1/C5AR1/C5AR2/CCR1/CCR8/CD4/CXCR4/FPR1/FPR3/P2RX7/P2RY6/TRPV2 | 41 |
| BP | GO:0001654 | eye development | 45/941 | 359/18862 | 1.23E-08 | 5.26E-07 | 3.78E-07 | ATP2B4/CLIC4/CYP1B1/EFEMP1/FGF10/FZD4/GLI3/MAF/MEIS1/MFAP5/MITF/NOTCH2/NRP1/PBX3/PDGFRA/PRPH2/PTPRM/RARB/RHOJ/SDK1/SERPINF1/SMARCD3/SOX11/TENM3/TUB/VIM/ALDH1A3/ARHGEF15/COL4A1/COL5A1/COL5A2/COL8A1/COL8A2/FBN1/FLT1/INHBA/LAMB2/MEIS3/MFAP2/PDGFRB/SH3PXD2B/SPRED3/THY1/TWIST1/WNT2 | 45 |
| BP | GO:0060541 | respiratory system development | 30/941 | 188/18862 | 1.58E-08 | 6.59E-07 | 4.73E-07 | BASP1/CRISPLD2/FGF1/FGF10/FGF7/GLI1/GLI3/IGFBP5/PDGFRA/SOX11/TGFB3/TNC/ZFPM2/ADAMTS2/ALDH1A3/FLT4/FSTL3/GLI2/HEG1/LOX/LOXL3/LTBP3/MMP14/MSC/PDGFRB/PDPN/RCN3/SPARC/WNT2/HSD11B1 | 30 |
| BP | GO:0001885 | endothelial cell development | 17/941 | 66/18862 | 1.59E-08 | 6.59E-07 | 4.73E-07 | CLIC4/MSN/RDX/S1PR3/VCL/CDH5/COL15A1/COL18A1/COL22A1/ENG/HEG1/NOTCH4/PECAM1/ROBO4/STC1/ICAM1/S1PR2 | 17 |
| BP | GO:0061035 | regulation of cartilage development | 17/941 | 66/18862 | 1.59E-08 | 6.59E-07 | 4.73E-07 | EFEMP1/GDF6/GLI3/GREM1/MAF/NKX3-2/RARB/SNAI2/SOX5/TRPS1/ADAMTS12/ADAMTS7/BMP1/GLI2/LOXL2/LTBP3/PTHLH | 17 |
| BP | GO:0061437 | renal system vasculature development | 11/941 | 26/18862 | 1.74E-08 | 7.12E-07 | 5.11E-07 | ACTA2/ANGPT1/NOTCH2/PDGFRA/PKD2/TEK/CD34/GPR4/PDGFB/PDGFRB/PECAM1 | 11 |
| BP | GO:0061440 | kidney vasculature development | 11/941 | 26/18862 | 1.74E-08 | 7.12E-07 | 5.11E-07 | ACTA2/ANGPT1/NOTCH2/PDGFRA/PKD2/TEK/CD34/GPR4/PDGFB/PDGFRB/PECAM1 | 11 |
| BP | GO:0072006 | nephron development | 25/941 | 138/18862 | 1.87E-08 | 7.59E-07 | 5.45E-07 | ACTA2/ADAMTS16/ANGPT1/BASP1/FAT4/FGF1/GLI3/GREM1/MEF2C/NOTCH2/PDGFRA/PKD2/TEK/WWTR1/CD34/DCHS1/GPR4/HEYL/KIF26B/LAMB2/NID1/PDGFB/PDGFRB/PECAM1/SULF1 | 25 |
| BP | GO:0014812 | muscle cell migration | 21/941 | 101/18862 | 2.10E-08 | 8.44E-07 | 6.06E-07 | ADAMTS1/IGFBP5/ITGB3/MEF2C/NRP1/PARVA/PLEKHO1/PRKG1/SLIT2/SSH1/THBS4/IGFBP3/NOX4/PDGFB/PDGFRB/PLAT/PLAU/POSTN/SERPINE1/AIF1/DOCK4 | 21 |
| BP | GO:0010959 | regulation of metal ion transport | 35/941 | 247/18862 | 2.42E-08 | 9.64E-07 | 6.92E-07 | CACNA2D1/CAV1/FYN/GEM/GJA1/PKD2/REM1/RGS4/SLC8A1/TRPC1/TRPC6/APLNR/EHD3/F2R/F2RL3/GNAI2/PDGFB/PDGFRB/RAMP3/STC1/THY1/CCL2/CCR1/CD33/CD4/CD84/CXCR4/ICAM1/LILRA2/LILRA5/LILRB1/LILRB2/P2RX7/P2RY6/TRPV2 | 35 |
| BP | GO:0031214 | biomineral tissue development | 27/941 | 160/18862 | 2.46E-08 | 9.72E-07 | 6.98E-07 | ASPN/DDR2/GREM1/MEF2C/MGP/OMD/ROR2/S1PR1/SLC24A3/SLC8A1/TGFB3/TMEM119/ALPL/COL1A1/COL1A2/COMP/FAM20C/LOX/LTBP3/PTHLH/TWIST1/CCR1/FAM20A/FGR/P2RX7/SRGN/TGFB1 | 27 |
| BP | GO:0007492 | endoderm development | 18/941 | 76/18862 | 2.52E-08 | 9.82E-07 | 7.05E-07 | ITGA5/LAMC1/SOX7/COL11A1/COL12A1/COL4A2/COL5A1/COL5A2/COL6A1/COL7A1/COL8A1/FN1/INHBA/MMP14/MMP2/SOX17/ITGB2/MMP9 | 18 |
| BP | GO:0042116 | macrophage activation | 21/941 | 102/18862 | 2.52E-08 | 9.82E-07 | 7.05E-07 | LRRK2/RORA/SYT11/CD93/DYSF/THBS1/AIF1/C1QA/C5AR1/FCGR2B/HAVCR2/ITGAM/ITGB2/SLC11A1/SPHK1/TLR1/TLR2/TLR8/TREM2/TYROBP/VSIG4 | 21 |
| BP | GO:0006909 | phagocytosis | 46/941 | 381/18862 | 2.73E-08 | 1.05E-06 | 7.55E-07 | AXL/C4B/COLEC12/FYN/ITGB1/ITGB3/RAB34/SYT11/TUB/WIPF1/C3/CD93/DYSF/MFGE8/PECAM1/RAB31/SIRPA/THBS1/AIF1/CCL2/CD14/CD300A/CEACAM4/CLEC7A/FCER1G/FCGR1A/FCGR2A/FCGR2B/FCGR3A/FCN1/FGR/HCK/ITGAM/ITGB2/MARCO/MSR1/MYO1G/NCF2/P2RY6/RAB7B/SIRPB1/SLC11A1/SPHK1/TLR2/TREM2/TYROBP | 46 |
| BP | GO:0032102 | negative regulation of response to external stimulus | 47/941 | 394/18862 | 2.80E-08 | 1.07E-06 | 7.70E-07 | A2M/C1QTNF3/CALCRL/GJA1/GREM1/HGF/NRP1/PDGFRA/PHLDB2/PRKG1/PTGIS/RORA/SERPINF1/SERPING1/SLIT2/SYT11/TEK/CD34/CDH5/FAP/FNDC4/HTRA1/PDGFB/PLAT/PLAU/SEMA6B/SEMA7A/SERPINE1/SIRPA/THBD/THBS1/TNFAIP6/AIF1/APOE/C5AR2/CCL2/FCGR2B/FOXP3/HAVCR2/LILRA2/LILRB1/NLRP3/SAMHD1/SLAMF8/SOCS3/TNFAIP8L2/VSIG4 | 47 |
| BP | GO:0051480 | regulation of cytosolic calcium ion concentration | 43/941 | 344/18862 | 2.83E-08 | 1.07E-06 | 7.71E-07 | ATP2B4/AVPR1A/CACNA2D1/CAV1/CAV2/CCL21/FYN/GJA1/HTR2B/ITPR1/PDGFRA/PKD2/PTGER3/PTGFR/S1PR1/S1PR3/SLC8A1/TRPC1/TRPC4/TRPC6/APLNR/DLG4/EDNRA/F2R/F2RL3/GPR4/PTGIR/RAMP3/TBXA2R/THY1/TRPV4/C3AR1/C5AR1/C5AR2/CCR1/CCR8/CD4/CXCR4/FPR1/FPR3/P2RX7/P2RY6/TRPV2 | 43 |
| BP | GO:0002446 | neutrophil mediated immunity | 55/941 | 499/18862 | 2.99E-08 | 1.13E-06 | 8.08E-07 | CRISPLD2/STOM/TIMP2/VCL/C3/CD93/PECAM1/PLAU/RAB31/SIRPA/SLC2A3/TNFAIP6/ADAM8/C3AR1/C5AR1/CD14/CD300A/CD33/CD53/CLEC5A/CTSB/CYBB/DOK3/FCER1G/FCGR2A/FCN1/FGR/FPR1/GMFG/GPR84/HK3/ITGAM/ITGAX/ITGB2/LAIR1/LILRB2/LILRB3/MCEMP1/MMP9/MNDA/NFAM1/OLR1/OSCAR/PLEKHO2/PRAM1/PSAP/PTAFR/RNASE2/S100A8/SIGLEC14/SIGLEC9/SIRPB1/SLC11A1/TLR2/TYROBP | 55 |
| BP | GO:0050764 | regulation of phagocytosis | 20/941 | 94/18862 | 3.01E-08 | 1.13E-06 | 8.09E-07 | C4B/SYT11/TUB/C3/DYSF/MFGE8/RAB31/SIRPA/CCL2/CD300A/CLEC7A/FCER1G/FCGR2B/FGR/HCK/SIRPB1/SLC11A1/SPHK1/TLR2/TREM2 | 20 |
| BP | GO:0050730 | regulation of peptidyl-tyrosine phosphorylation | 36/941 | 262/18862 | 3.44E-08 | 1.28E-06 | 9.16E-07 | ANGPT1/CAV1/CSPG4/FGF10/FGF7/FYN/GREM1/HGF/IL6ST/ITGA5/ITGB3/NRP1/PDGFC/SFRP2/SH3BP5/THBS4/DLG4/GPRC5B/NOX4/PDGFB/PECAM1/THY1/CASS4/CD300A/CD4/CLEC7A/CSF1R/FCGR1A/ICAM1/ITGB2/LILRA5/LILRB4/SAMSN1/SOCS3/TGFB1/TREM2 | 36 |
| BP | GO:0030324 | lung development | 27/941 | 163/18862 | 3.68E-08 | 1.36E-06 | 9.74E-07 | CRISPLD2/FGF1/FGF10/FGF7/GLI1/GLI3/IGFBP5/PDGFRA/SOX11/TGFB3/TNC/ZFPM2/ADAMTS2/FLT4/FSTL3/GLI2/HEG1/LOX/LOXL3/LTBP3/MMP14/PDGFRB/PDPN/RCN3/SPARC/WNT2/HSD11B1 | 27 |
| BP | GO:0001837 | epithelial to mesenchymal transition | 26/941 | 153/18862 | 3.84E-08 | 1.41E-06 | 1.01E-06 | EPHA3/FERMT2/FGFR1/FLNA/GREM1/HGF/PHLDB1/PHLDB2/SFRP2/SNAI2/TGFB1I1/TGFB3/WWTR1/COL1A1/ENG/HEYL/LOXL2/LOXL3/NOTCH4/PDPN/SPRED3/TWIST1/VASN/WNT2/GLIPR2/TGFB1 | 26 |
| BP | GO:0002687 | positive regulation of leukocyte migration | 24/941 | 133/18862 | 3.89E-08 | 1.41E-06 | 1.01E-06 | CCL21/CD99L2/IL1R1/JAM3/THBS4/VEGFC/MMP14/PLVAP/SELP/SERPINE1/THBS1/THY1/ADAM8/AIF1/C3AR1/C5AR1/CCR1/CSF1/CSF1R/GPSM3/ICAM1/PLA2G7/PTAFR/TREM2 | 24 |
| BP | GO:0002064 | epithelial cell development | 31/941 | 207/18862 | 4.19E-08 | 1.50E-06 | 1.08E-06 | ACTA2/ADAMTSL4/CDH2/CLIC4/FLNA/IL6ST/MSN/NKX3-2/NOTCH2/PALLD/RARB/RDX/S1PR3/VCL/VIM/CDH5/COL15A1/COL18A1/COL22A1/ENG/GPR4/HEG1/LAMB2/NOTCH4/PDGFB/PECAM1/ROBO4/STC1/CXCR4/ICAM1/S1PR2 | 31 |
| BP | GO:0050808 | synapse organization | 48/941 | 412/18862 | 4.19E-08 | 1.50E-06 | 1.08E-06 | CDH2/FLNA/FLRT2/FYN/FZD1/GLRB/GPC6/LGI2/LRRK2/MAP1B/MDGA1/MEF2C/NLGN4X/NRP2/PCDHB4/PCDHB5/PCDHGC3/SDK1/SPARCL1/SSH1/SYNPO/TNC/TUBA1A/ARHGEF15/C3/COL4A1/DLG4/F2R/LAMB2/MYH10/NLGN2/NTNG2/PCDH17/PLXND1/SLC6A1/SPARC/SYNDIG1/APOE/C1QA/C1QB/C1QC/C5AR1/FCGR2B/ITGAM/LILRB2/SRGN/TLR2/TREM2 | 48 |
| BP | GO:0048010 | vascular endothelial growth factor receptor signaling pathway | 20/941 | 96/18862 | 4.38E-08 | 1.56E-06 | 1.12E-06 | AXL/FGF10/FYN/FZD4/ITGA5/ITGB3/NRP1/NRP2/PRKD1/VEGFC/CLEC14A/EMILIN1/FLT1/FLT4/KDR/MMRN2/SULF1/TMEM204/CYBB/NCF2 | 20 |
| BP | GO:0014909 | smooth muscle cell migration | 19/941 | 88/18862 | 5.18E-08 | 1.83E-06 | 1.31E-06 | ADAMTS1/IGFBP5/ITGB3/MEF2C/NRP1/PARVA/PRKG1/SLIT2/SSH1/IGFBP3/NOX4/PDGFB/PDGFRB/PLAT/PLAU/POSTN/SERPINE1/AIF1/DOCK4 | 19 |
| BP | GO:0035265 | organ growth | 27/941 | 166/18862 | 5.45E-08 | 1.91E-06 | 1.37E-06 | ANXA6/BASP1/BNC2/DDR2/EVC/FGF10/FGF7/GJA1/GLI1/HLX/LATS2/MEF2C/MEIS1/NLGN4X/RARB/RGS2/RGS4/S1PR1/WWC2/ZFPM2/COMP/HEG1/PDGFRB/STC1/TENM4/WNT2/PSAP | 27 |
| BP | GO:0032331 | negative regulation of chondrocyte differentiation | 10/941 | 23/18862 | 5.75E-08 | 2.00E-06 | 1.44E-06 | EFEMP1/GREM1/NKX3-2/RARB/SNAI2/ADAMTS12/ADAMTS7/GLI2/LTBP3/PTHLH | 10 |
| BP | GO:0061298 | retina vasculature development in camera-type eye | 9/941 | 18/18862 | 5.96E-08 | 2.06E-06 | 1.48E-06 | CLIC4/CYP1B1/FZD4/NRP1/PDGFRA/RHOJ/ARHGEF15/COL4A1/PDGFRB | 9 |
| BP | GO:0050727 | regulation of inflammatory response | 44/941 | 366/18862 | 6.20E-08 | 2.11E-06 | 1.52E-06 | BCL6/C1QTNF3/CALCRL/HGF/IL1R1/IL6ST/LRRK2/OSMR/PTGER3/PTGIS/RORA/SERPINF1/SYT11/TEK/ADAMTS12/BCL6B/C3/CDH5/FNDC4/GPR4/GPRC5B/SELE/SEMA7A/SERPINE1/SIRPA/TNFAIP6/TNFSF4/ADAM8/APOE/FCGR2B/FOXP3/GPSM3/HCK/LILRA5/MMP9/NLRP3/PLA2G7/S100A8/SLAMF8/SOCS3/SPHK1/TLR2/TNFAIP8L2/TREM2 | 44 |
| BP | GO:0030323 | respiratory tube development | 27/941 | 167/18862 | 6.20E-08 | 2.11E-06 | 1.52E-06 | CRISPLD2/FGF1/FGF10/FGF7/GLI1/GLI3/IGFBP5/PDGFRA/SOX11/TGFB3/TNC/ZFPM2/ADAMTS2/FLT4/FSTL3/GLI2/HEG1/LOX/LOXL3/LTBP3/MMP14/PDGFRB/PDPN/RCN3/SPARC/WNT2/HSD11B1 | 27 |
| BP | GO:0033627 | cell adhesion mediated by integrin | 17/941 | 72/18862 | 6.45E-08 | 2.16E-06 | 1.55E-06 | CCL21/CYP1B1/ITGA5/ITGB1/ITGB3/JAM3/SFRP2/SNAI2/COL16A1/FBN1/ITGA11/ITGBL1/PLAU/SERPINE1/FERMT3/ICAM1/ITGB2 | 17 |
| BP | GO:0035924 | cellular response to vascular endothelial growth factor stimulus | 17/941 | 72/18862 | 6.45E-08 | 2.16E-06 | 1.55E-06 | ADAMTS3/ATP2B4/DCN/GAS1/NRP1/NRP2/PDGFRA/PRKD1/VCAM1/VEGFC/ADAMTS12/FLT1/FLT4/KDR/PDGFRB/RAMP2/SPHK1 | 17 |
| BP | GO:0050870 | positive regulation of T cell activation | 31/941 | 212/18862 | 7.33E-08 | 2.44E-06 | 1.75E-06 | BCL6/CAV1/CCL21/FYN/GLI3/HLX/IL6ST/VCAM1/EFNB3/GLI2/LGALS1/SIRPA/THY1/TNFSF4/ADAM8/AIF1/CCL2/CD4/CD86/FOXP3/HAVCR2/HLA-DPB1/LILRB1/LILRB2/LILRB4/NLRP3/PDCD1LG2/PIK3R6/SIRPB1/TNFSF13B/TNFSF14 | 31 |
| BP | GO:0014068 | positive regulation of phosphatidylinositol 3-kinase signaling | 19/941 | 90/18862 | 7.59E-08 | 2.52E-06 | 1.81E-06 | ANGPT1/DCN/FGFR1/FYN/HGF/PDGFC/PDGFRA/ROR2/TEK/F2R/FLT1/FN1/KDR/PDGFB/PDGFRB/SELP/UNC5B/FGR/HCST | 19 |
| BP | GO:0051928 | positive regulation of calcium ion transport | 22/941 | 118/18862 | 7.77E-08 | 2.56E-06 | 1.84E-06 | CACNA2D1/CAV1/PKD2/TRPC1/TRPC6/APLNR/EHD3/F2R/F2RL3/PDGFB/PDGFRB/RAMP3/STC1/THY1/CCL2/CCR1/CD4/LILRA2/LILRA5/P2RX7/P2RY6/TRPV2 | 22 |
| BP | GO:0030595 | leukocyte chemotaxis | 32/941 | 226/18862 | 9.75E-08 | 3.19E-06 | 2.29E-06 | CCL21/CYP7B1/GREM1/ITGA1/JAM3/S1PR1/SLIT2/THBS4/VEGFC/FLT1/PDGFB/SERPINE1/THBS1/ADAM8/AIF1/C3AR1/C5AR1/C5AR2/CCL18/CCL2/CCR1/CSF1/CSF1R/CSF3R/CXCR4/FCER1G/GPSM3/ITGB2/PLA2G7/PREX1/S100A8/SLAMF8 | 32 |
| BP | GO:0050654 | chondroitin sulfate proteoglycan metabolic process | 13/941 | 43/18862 | 9.89E-08 | 3.21E-06 | 2.30E-06 | CHST15/CHST3/CSGALNACT2/CSPG4/DCN/DSEL/BGN/CHSY3/DSE/EGFLAM/VCAN/CHST11/CYTL1 | 13 |
| BP | GO:0050650 | chondroitin sulfate proteoglycan biosynthetic process | 11/941 | 30/18862 | 1.02E-07 | 3.30E-06 | 2.37E-06 | CHST15/CHST3/CSGALNACT2/CSPG4/DCN/BGN/CHSY3/DSE/VCAN/CHST11/CYTL1 | 11 |
| BP | GO:0055074 | calcium ion homeostasis | 50/941 | 454/18862 | 1.31E-07 | 4.20E-06 | 3.01E-06 | ANXA6/ATP2B4/AVPR1A/CACNA2D1/CAV1/CAV2/CCL21/FYN/GJA1/HTR2B/ITPR1/PDGFRA/PKD2/PTGER3/PTGFR/S1PR1/S1PR3/SLC24A3/SLC8A1/SV2A/TRPC1/TRPC4/TRPC6/APLNR/CDH5/DLG4/EDNRA/F2R/F2RL3/GPR4/PTGIR/RAMP3/STC1/TBXA2R/THY1/TRPV4/APOE/C3AR1/C5AR1/C5AR2/CCR1/CCR8/CD4/CXCR4/FAM20A/FPR1/FPR3/P2RX7/P2RY6/TRPV2 | 50 |
| BP | GO:1900046 | regulation of hemostasis | 16/941 | 67/18862 | 1.32E-07 | 4.21E-06 | 3.02E-06 | CAV1/PDGFRA/PRKG1/SERPING1/CD34/COMP/F2R/FAP/PDGFB/PLAT/PLAU/SERPINE1/TBXA2R/THBD/THBS1/APOE | 16 |
| BP | GO:0010863 | positive regulation of phospholipase C activity | 13/941 | 44/18862 | 1.34E-07 | 4.24E-06 | 3.04E-06 | AVPR1A/FGFR1/HTR2B/PDGFRA/EDNRA/FLT1/PDGFRB/SELE/C5AR1/CD86/P2RY6/PTAFR/RASGRP4 | 13 |
| BP | GO:0010975 | regulation of neuron projection development | 48/941 | 428/18862 | 1.35E-07 | 4.25E-06 | 3.05E-06 | CCDC88A/CDH2/CRMP1/DENND5A/DPYSL3/EPHA3/FEZ1/FLNA/FYN/FZD1/HGF/ISLR2/LRRK2/MAP1B/NRP1/PLXNA4/PRKD1/RGS2/ROR2/SCN1B/SDC2/SERPINF1/SFRP2/SLIT2/TENM3/TRPC6/VIM/CHN1/EFNB3/FN1/HECW2/LGALS1/LZTS1/NTNG2/P3H1/PLXND1/PRRX1/SEMA6B/SEMA7A/SPOCK1/THY1/TRPV4/APOE/FES/PLXNC1/PREX1/SCARF1/TRPV2 | 48 |
| BP | GO:1903706 | regulation of hemopoiesis | 47/941 | 415/18862 | 1.36E-07 | 4.26E-06 | 3.06E-06 | AXL/BCL6/GLI3/HLX/LRRC17/MEF2C/MEIS1/MITF/NOTCH2/RBFOX2/ROR2/FBN1/FSTL3/GLI2/GPR68/INHBA/LOX/LOXL3/MMP14/THBS1/TNFSF4/ZBTB46/ADAM8/C1QC/CCR1/CD4/CD86/CSF1/CSF3R/FCGR2B/FES/FOXP3/HLA-DOA/LILRB1/LILRB2/LILRB3/LILRB4/MAFB/NFAM1/NLRP3/PIK3R6/RAB7B/SLAMF8/SPI1/TGFB1/TREM2/TYROBP | 47 |
| BP | GO:0006874 | cellular calcium ion homeostasis | 49/941 | 442/18862 | 1.43E-07 | 4.44E-06 | 3.19E-06 | ANXA6/ATP2B4/AVPR1A/CACNA2D1/CAV1/CAV2/CCL21/FYN/GJA1/HTR2B/ITPR1/PDGFRA/PKD2/PTGER3/PTGFR/S1PR1/S1PR3/SLC24A3/SLC8A1/SV2A/TRPC1/TRPC4/TRPC6/APLNR/CDH5/DLG4/EDNRA/F2R/F2RL3/GPR4/PTGIR/RAMP3/STC1/TBXA2R/THY1/TRPV4/APOE/C3AR1/C5AR1/C5AR2/CCR1/CCR8/CD4/CXCR4/FPR1/FPR3/P2RX7/P2RY6/TRPV2 | 49 |
| BP | GO:0030206 | chondroitin sulfate biosynthetic process | 10/941 | 25/18862 | 1.50E-07 | 4.60E-06 | 3.30E-06 | CHST15/CHST3/CSGALNACT2/CSPG4/DCN/BGN/CHSY3/DSE/VCAN/CHST11 | 10 |
| BP | GO:0072012 | glomerulus vasculature development | 10/941 | 25/18862 | 1.50E-07 | 4.60E-06 | 3.30E-06 | ACTA2/ANGPT1/NOTCH2/PDGFRA/TEK/CD34/GPR4/PDGFB/PDGFRB/PECAM1 | 10 |
| BP | GO:0050863 | regulation of T cell activation | 40/941 | 327/18862 | 1.55E-07 | 4.71E-06 | 3.38E-06 | BCL6/CAV1/CCL21/FYN/GLI3/HLX/IL6ST/VCAM1/EFNB3/GLI2/LGALS1/LOXL3/LRRC32/SIRPA/THY1/TNFSF4/ADAM8/AIF1/CCL2/CD300A/CD4/CD86/FCGR2B/FOXP3/GPNMB/HAVCR2/HLA-DOA/HLA-DPB1/LAPTM5/LILRB1/LILRB2/LILRB4/NLRP3/PDCD1LG2/PIK3R6/SIRPB1/TNFAIP8L2/TNFSF13B/TNFSF14/VSIG4 | 40 |
| BP | GO:0097485 | neuron projection guidance | 36/941 | 278/18862 | 1.57E-07 | 4.75E-06 | 3.41E-06 | BOC/CRMP1/DOK6/EPHA3/FEZ1/FLRT2/FYN/GLI3/LAMA2/NEXN/NOTCH2/NRP1/NRP2/PALLD/PDLIM7/PLXNA4/PTPRM/SCN1B/SLIT2/CHN1/DOK5/DPYSL4/EFNB3/EGR2/GLI2/LAMB2/NOTCH3/PLXND1/ROBO4/SEMA6B/SEMA7A/UNC5B/CSF1R/CXCR4/DOK2/PLXNC1 | 36 |
| BP | GO:0060350 | endochondral bone morphogenesis | 14/941 | 52/18862 | 1.61E-07 | 4.84E-06 | 3.48E-06 | ANXA6/MEF2C/MMP16/RARB/TEK/TMEM119/ALPL/COL13A1/COL1A1/COMP/MMP14/SERPINH1/STC1/TRPV4 | 14 |
| BP | GO:0048754 | branching morphogenesis of an epithelial tube | 24/941 | 143/18862 | 1.62E-07 | 4.85E-06 | 3.48E-06 | ADAMTS16/CLIC4/FAT4/FGF1/FGF10/GLI3/GREM1/NRP1/PKD2/SFRP2/SLIT2/TNC/COL4A1/DCHS1/EDNRA/ENG/GLI2/KDR/MMP14/NOTCH4/PLXND1/TIE1/WNT2/CSF1 | 24 |
| BP | GO:0042476 | odontogenesis | 22/941 | 123/18862 | 1.67E-07 | 4.96E-06 | 3.56E-06 | AQP1/ASPN/FGF10/FST/GLI3/PDGFRA/TGFB3/TNC/ADAMTS5/ALPL/CD34/COL1A1/COL1A2/FAM20C/GLI2/HTRA1/INHBA/SERPINE1/TWIST1/CSF1/CSF3R/FAM20A | 22 |
| BP | GO:0006898 | receptor-mediated endocytosis | 40/941 | 328/18862 | 1.68E-07 | 4.96E-06 | 3.56E-06 | ANGPT1/CALCRL/CAV1/CAV2/CCL21/COLEC12/GREM1/HSPG2/HTR2B/ITGB1/ITGB3/PDLIM7/SYT11/C3/DLG4/RAB31/RAMP2/RAMP3/SELE/SERPINE1/SFRP4/SGIP1/SPARC/APOE/CD14/CD163/CTSL/FCER1G/FCGR1A/FCGR2B/ITGAM/ITGB2/LILRB1/LILRB4/MARCO/MRC1/MSR1/SCARF1/SIGLEC1/STAB1 | 40 |
| BP | GO:1902107 | positive regulation of leukocyte differentiation | 25/941 | 154/18862 | 1.75E-07 | 5.09E-06 | 3.65E-06 | AXL/BCL6/GLI3/HLX/NOTCH2/ROR2/GLI2/GPR68/MMP14/TNFSF4/ZBTB46/ADAM8/CCR1/CD4/CD86/CSF1/FES/FOXP3/LILRB2/LILRB4/NLRP3/PIK3R6/TGFB1/TREM2/TYROBP | 25 |
| BP | GO:1903708 | positive regulation of hemopoiesis | 25/941 | 154/18862 | 1.75E-07 | 5.09E-06 | 3.65E-06 | AXL/BCL6/GLI3/HLX/NOTCH2/ROR2/GLI2/GPR68/MMP14/TNFSF4/ZBTB46/ADAM8/CCR1/CD4/CD86/CSF1/FES/FOXP3/LILRB2/LILRB4/NLRP3/PIK3R6/TGFB1/TREM2/TYROBP | 25 |
| BP | GO:0050651 | dermatan sulfate proteoglycan biosynthetic process | 8/941 | 15/18862 | 1.75E-07 | 5.09E-06 | 3.65E-06 | CSGALNACT2/CSPG4/DCN/DSEL/BGN/CHST14/DSE/VCAN | 8 |
| BP | GO:0030204 | chondroitin sulfate metabolic process | 12/941 | 38/18862 | 1.80E-07 | 5.19E-06 | 3.73E-06 | CHST15/CHST3/CSGALNACT2/CSPG4/DCN/DSEL/BGN/CHSY3/DSE/EGFLAM/VCAN/CHST11 | 12 |
| BP | GO:0010632 | regulation of epithelial cell migration | 37/941 | 293/18862 | 2.02E-07 | 5.78E-06 | 4.15E-06 | AKT3/ANGPT1/ATP2B4/DCN/FGF1/FGF10/FGF7/FGFR1/ITGB3/MAP3K3/MCC/MEF2C/MEOX2/NRP1/NRP2/PRKD1/PTPRM/RHOJ/SASH1/SERPINF1/SLIT2/TEK/VEGFC/FLT4/KDR/MMRN2/PDGFB/PPM1F/SPARC/STC1/TBXA2R/THBS1/VASH1/APOE/GLIPR2/MMP9/TGFB1 | 37 |
| BP | GO:0061299 | retina vasculature morphogenesis in camera-type eye | 7/941 | 11/18862 | 2.08E-07 | 5.93E-06 | 4.26E-06 | CLIC4/CYP1B1/FZD4/NRP1/RHOJ/ARHGEF15/COL4A1 | 7 |
| BP | GO:0033002 | muscle cell proliferation | 31/941 | 222/18862 | 2.11E-07 | 5.99E-06 | 4.30E-06 | ADAMTS1/ANGPT1/CALCRL/GJA1/GLI1/IGFBP5/MEF2C/MEIS1/NPR3/PDE1A/PRKG1/S1PR1/TGFB3/ZFPM2/CDH13/EFEMP2/ELN/GNAI2/IGFBP3/MMP2/NPR1/PDGFB/PDGFRB/PTGIR/TENM4/THBS1/WNT2/AIF1/MMP9/P2RY6/PTAFR | 31 |
| BP | GO:0001704 | formation of primary germ layer | 21/941 | 115/18862 | 2.21E-07 | 6.22E-06 | 4.47E-06 | GJA1/ITGA5/ITGB1/ITGB3/SFRP2/SOX7/COL11A1/COL12A1/COL4A2/COL5A1/COL5A2/COL6A1/COL7A1/COL8A1/FN1/INHBA/MMP14/MMP2/SOX17/ITGB2/MMP9 | 21 |
| BP | GO:0001952 | regulation of cell-matrix adhesion | 22/941 | 125/18862 | 2.24E-07 | 6.29E-06 | 4.51E-06 | BCL6/CCL21/DLC1/EPHA3/FERMT2/GREM1/NRP1/PHLDB2/TEK/VCL/CDH13/COL16A1/EFEMP2/KDR/MMP14/PLAU/POSTN/PPM1F/SERPINE1/THBS1/THY1/CSF1 | 22 |
| BP | GO:0006816 | calcium ion transport | 46/941 | 409/18862 | 2.27E-07 | 6.33E-06 | 4.54E-06 | ANXA6/ATP2B4/CACNA2D1/CALCRL/CAV1/CCL21/FYN/GEM/GJA1/HTR2B/ITPR1/PKD2/REM1/RGS4/SLC24A3/SLC8A1/TRPC1/TRPC4/TRPC6/APLNR/EHD3/F2R/F2RL3/GJA4/GNAI2/PDGFB/PDGFRB/RAMP2/RAMP3/STC1/THY1/TRPV4/CCL2/CCR1/CD33/CD4/CD84/CXCR4/ICAM1/LILRA2/LILRA5/LILRB1/LILRB2/P2RX7/P2RY6/TRPV2 | 46 |
| BP | GO:0006029 | proteoglycan metabolic process | 18/941 | 87/18862 | 2.30E-07 | 6.37E-06 | 4.57E-06 | CHST15/CHST3/CSGALNACT2/CSPG4/DCN/DSEL/ADAMTS12/BGN/CHST14/CHSY3/COL11A1/DSE/EGFLAM/SULF1/VCAN/CHST11/CYTL1/HS3ST3B1 | 18 |
| BP | GO:1900274 | regulation of phospholipase C activity | 13/941 | 46/18862 | 2.39E-07 | 6.59E-06 | 4.73E-06 | AVPR1A/FGFR1/HTR2B/PDGFRA/EDNRA/FLT1/PDGFRB/SELE/C5AR1/CD86/P2RY6/PTAFR/RASGRP4 | 13 |
| BP | GO:0060562 | epithelial tube morphogenesis | 38/941 | 309/18862 | 2.75E-07 | 7.53E-06 | 5.40E-06 | ADAMTS16/CLIC4/DLC1/FAT4/FGF1/FGF10/GLI3/GREM1/MEF2C/NOTCH2/NRP1/PKD2/PRICKLE1/SFRP2/SLIT2/SOX11/TNC/ADAMTS12/COL4A1/CTHRC1/DCHS1/EDNRA/ENG/GLI2/KDR/KIF26B/MMP14/NOTCH4/PLXND1/SOX17/TIE1/TWIST1/WNT2/CSF1/CSF1R/CXCR4/ITGAX/TGFB1 | 38 |
| BP | GO:0007179 | transforming growth factor beta receptor signaling pathway | 29/941 | 202/18862 | 2.86E-07 | 7.79E-06 | 5.59E-06 | ASPN/CAV1/CAV2/FERMT2/LATS2/LTBP1/SOX11/TGFB1I1/TGFB3/CDH5/COL1A2/COL3A1/DKK3/EMILIN1/ENG/FBN1/HTRA1/HTRA3/LOX/LRRC32/LTBP2/LTBP3/NREP/SPRED3/THBS1/VASN/CHST11/NRROS/TGFB1 | 29 |
| BP | GO:0017015 | regulation of transforming growth factor beta receptor signaling pathway | 22/941 | 127/18862 | 2.99E-07 | 8.10E-06 | 5.81E-06 | ASPN/CAV1/CAV2/LATS2/LTBP1/SOX11/TGFB1I1/TGFB3/DKK3/EMILIN1/ENG/FBN1/HTRA1/HTRA3/LOX/NREP/SPRED3/THBS1/VASN/CHST11/NRROS/TGFB1 | 22 |
| BP | GO:0072503 | cellular divalent inorganic cation homeostasis | 51/941 | 480/18862 | 3.01E-07 | 8.12E-06 | 5.83E-06 | ANXA6/ATP2B4/AVPR1A/CACNA2D1/CAV1/CAV2/CCL21/FYN/GJA1/HTR2B/ITPR1/PDGFRA/PKD2/PTGER3/PTGFR/S1PR1/S1PR3/SLC24A3/SLC8A1/SV2A/TRPC1/TRPC4/TRPC6/APLNR/CDH5/DLG4/EDNRA/F2R/F2RL3/GPR4/PTGIR/RAMP3/STC1/TBXA2R/THY1/TRPV4/APOE/C3AR1/C5AR1/C5AR2/CCR1/CCR8/CD4/CXCR4/FPR1/FPR3/P2RX7/P2RY6/S100A8/SLC11A1/TRPV2 | 51 |
| BP | GO:0097530 | granulocyte migration | 24/941 | 148/18862 | 3.14E-07 | 8.32E-06 | 5.97E-06 | CCL21/CD99L2/IL1R1/ITGA1/JAM3/SLIT2/THBS4/PECAM1/THBS1/ADAM8/C3AR1/C5AR1/C5AR2/CCL18/CCL2/CD300A/CSF1/CSF1R/CSF3R/FCER1G/ITGB2/PREX1/S100A8/SLAMF8 | 24 |
| BP | GO:0001774 | microglial cell activation | 13/941 | 47/18862 | 3.16E-07 | 8.32E-06 | 5.97E-06 | LRRK2/SYT11/AIF1/C1QA/C5AR1/ITGAM/ITGB2/SPHK1/TLR1/TLR2/TLR8/TREM2/TYROBP | 13 |
| BP | GO:0007157 | heterophilic cell-cell adhesion via plasma membrane cell adhesion molecules | 13/941 | 47/18862 | 3.16E-07 | 8.32E-06 | 5.97E-06 | CADM1/CDH2/FAT4/HMCN1/ITGA5/TENM3/VCAM1/DCHS1/SCARF2/SELE/SELP/TENM4/ICAM1 | 13 |
| BP | GO:0030225 | macrophage differentiation | 13/941 | 47/18862 | 3.16E-07 | 8.32E-06 | 5.97E-06 | ROR2/TSPAN2/INHBA/ZBTB46/C1QC/CD4/CSF1/CSF1R/MMP9/NRROS/SPI1/TGFB1/TLR2 | 13 |
| BP | GO:0050679 | positive regulation of epithelial cell proliferation | 29/941 | 203/18862 | 3.18E-07 | 8.34E-06 | 5.99E-06 | AKT3/CAV2/CYP7B1/FGF1/FGF10/FGF7/FGFR1/HTR2B/ITGB3/LAMC1/NOTCH2/NRP1/NRP2/PRKD1/SOX11/TEK/THBS4/TNFSF12/VEGFC/APLNR/CDH13/FLT4/HTRA1/KDR/PDGFB/TWIST1/WNT2/C5AR1/C5AR2 | 29 |
| BP | GO:0002696 | positive regulation of leukocyte activation | 45/941 | 401/18862 | 3.27E-07 | 8.54E-06 | 6.13E-06 | AXL/BCL6/CAV1/CCL21/FGF10/FYN/GLI3/HLX/IL6ST/LRRK2/MEF2C/VCAM1/EFNB3/GLI2/LGALS1/MMP14/SIRPA/THBS1/THY1/TNFSF4/ADAM8/AIF1/CCL2/CD4/CD86/CLEC7A/FGR/FOXP3/HAVCR2/HLA-DPB1/ITGAM/ITGB2/LILRB1/LILRB2/LILRB4/NLRP3/PDCD1LG2/PIK3R6/PTAFR/SIRPB1/TGFB1/TNFSF13B/TNFSF14/TREM2/TYROBP | 45 |
| BP | GO:0002693 | positive regulation of cellular extravasation | 8/941 | 16/18862 | 3.35E-07 | 8.65E-06 | 6.21E-06 | CD99L2/IL1R1/JAM3/PLVAP/THY1/ADAM8/ICAM1/PTAFR | 8 |
| BP | GO:0050655 | dermatan sulfate proteoglycan metabolic process | 8/941 | 16/18862 | 3.35E-07 | 8.65E-06 | 6.21E-06 | CSGALNACT2/CSPG4/DCN/DSEL/BGN/CHST14/DSE/VCAN | 8 |
| BP | GO:0034446 | substrate adhesion-dependent cell spreading | 20/941 | 108/18862 | 3.37E-07 | 8.66E-06 | 6.21E-06 | AXL/FBLN1/FERMT2/FLNA/FZD4/ITGB3/LAMC1/NRP1/PARVA/TEK/ANTXR1/FN1/LAMB2/NTNG2/PDPN/POSTN/CASS4/FERMT3/PARVG/PREX1 | 20 |
| BP | GO:0002761 | regulation of myeloid leukocyte differentiation | 21/941 | 118/18862 | 3.47E-07 | 8.86E-06 | 6.36E-06 | LRRC17/MITF/NOTCH2/ROR2/FBN1/FSTL3/GPR68/INHBA/ZBTB46/C1QC/CCR1/CD4/CSF1/FES/LILRB1/LILRB3/LILRB4/MAFB/TGFB1/TREM2/TYROBP | 21 |
| BP | GO:0002573 | myeloid leukocyte differentiation | 29/941 | 204/18862 | 3.54E-07 | 8.98E-06 | 6.45E-06 | LRRC17/MITF/NOTCH2/PDE1B/ROR2/TSPAN2/FBN1/FSTL3/GPR68/INHBA/ZBTB46/C1QC/CCR1/CD4/CSF1/CSF1R/FES/LILRB1/LILRB3/LILRB4/MAFB/MMP9/NRROS/OSCAR/SPI1/TGFB1/TLR2/TREM2/TYROBP | 29 |
| BP | GO:0048660 | regulation of smooth muscle cell proliferation | 25/941 | 160/18862 | 3.71E-07 | 9.38E-06 | 6.73E-06 | ADAMTS1/CALCRL/GJA1/IGFBP5/MEF2C/NPR3/PDE1A/PRKG1/S1PR1/TGFB3/CDH13/EFEMP2/ELN/GNAI2/IGFBP3/MMP2/NPR1/PDGFB/PDGFRB/PTGIR/THBS1/AIF1/MMP9/P2RY6/PTAFR | 25 |
| BP | GO:0030111 | regulation of Wnt signaling pathway | 42/941 | 365/18862 | 4.12E-07 | 1.04E-05 | 7.43E-06 | ANKRD6/APCDD1L/BICC1/CAV1/CDH2/CDK14/DAAM2/FGF10/FZD4/GLI1/GLI3/GREM1/IGFBP6/LATS2/LRRK2/MCC/MDFIC/NXN/PRICKLE1/RBMS3/RECK/ROR2/RSPO3/SFRP2/SNAI2/SOX7/WWTR1/ALPK2/COL1A1/CTHRC1/DACT1/DKK3/GPRC5B/HIC1/SFRP4/SOX17/SULF1/APOE/FOXP3/NFATC1/TGFB1/TLR2 | 42 |
| BP | GO:0072507 | divalent inorganic cation homeostasis | 52/941 | 499/18862 | 4.22E-07 | 1.05E-05 | 7.55E-06 | ANXA6/ATP2B4/AVPR1A/CACNA2D1/CAV1/CAV2/CCL21/FYN/GJA1/HTR2B/ITPR1/PDGFRA/PKD2/PTGER3/PTGFR/S1PR1/S1PR3/SLC24A3/SLC8A1/SV2A/TRPC1/TRPC4/TRPC6/APLNR/CDH5/DLG4/EDNRA/F2R/F2RL3/GPR4/PTGIR/RAMP3/STC1/TBXA2R/THY1/TRPV4/APOE/C3AR1/C5AR1/C5AR2/CCR1/CCR8/CD4/CXCR4/FAM20A/FPR1/FPR3/P2RX7/P2RY6/S100A8/SLC11A1/TRPV2 | 52 |
| BP | GO:0007411 | axon guidance | 35/941 | 277/18862 | 4.23E-07 | 1.05E-05 | 7.55E-06 | BOC/CRMP1/DOK6/EPHA3/FEZ1/FLRT2/FYN/GLI3/LAMA2/NEXN/NOTCH2/NRP1/NRP2/PALLD/PDLIM7/PLXNA4/PTPRM/SCN1B/SLIT2/CHN1/DOK5/EFNB3/EGR2/GLI2/LAMB2/NOTCH3/PLXND1/ROBO4/SEMA6B/SEMA7A/UNC5B/CSF1R/CXCR4/DOK2/PLXNC1 | 35 |
| BP | GO:0007044 | cell-substrate junction assembly | 19/941 | 100/18862 | 4.36E-07 | 1.08E-05 | 7.75E-06 | DLC1/EPHA3/FERMT2/GREM1/ITGA5/LAMC1/NRP1/PHLDB2/TEK/TLN1/VCL/COL16A1/FN1/KDR/MMP14/PPM1F/THBS1/THSD1/THY1 | 19 |
| BP | GO:0002691 | regulation of cellular extravasation | 11/941 | 34/18862 | 4.46E-07 | 1.10E-05 | 7.89E-06 | CCL21/CD99L2/IL1R1/JAM3/PLVAP/SELE/SELP/THY1/ADAM8/ICAM1/PTAFR | 11 |
| BP | GO:1903844 | regulation of cellular response to transforming growth factor beta stimulus | 22/941 | 130/18862 | 4.55E-07 | 1.11E-05 | 8.00E-06 | ASPN/CAV1/CAV2/LATS2/LTBP1/SOX11/TGFB1I1/TGFB3/DKK3/EMILIN1/ENG/FBN1/HTRA1/HTRA3/LOX/NREP/SPRED3/THBS1/VASN/CHST11/NRROS/TGFB1 | 22 |
| BP | GO:0048659 | smooth muscle cell proliferation | 25/941 | 162/18862 | 4.72E-07 | 1.15E-05 | 8.27E-06 | ADAMTS1/CALCRL/GJA1/IGFBP5/MEF2C/NPR3/PDE1A/PRKG1/S1PR1/TGFB3/CDH13/EFEMP2/ELN/GNAI2/IGFBP3/MMP2/NPR1/PDGFB/PDGFRB/PTGIR/THBS1/AIF1/MMP9/P2RY6/PTAFR | 25 |
| BP | GO:0007409 | axonogenesis | 49/941 | 460/18862 | 4.77E-07 | 1.15E-05 | 8.29E-06 | ADARB1/BOC/CDH2/CRMP1/DOK6/EPHA3/FEZ1/FLRT2/FYN/GLI3/ISLR2/LAMA2/MAP1A/MAP1B/NDN/NEXN/NOTCH2/NRP1/NRP2/PALLD/PDLIM7/PLXNA4/PTPRM/SCN1B/SLIT2/VCL/CDH11/CHN1/DOK5/EFNB3/EGR2/FN1/GLI2/LAMB2/NOTCH3/NTNG2/NUMBL/PLXND1/ROBO4/SEMA6B/SEMA7A/THY1/UNC5B/APOE/CSF1R/CXCR4/DOK2/PLXNC1/TRPV2 | 49 |
| BP | GO:0030208 | dermatan sulfate biosynthetic process | 7/941 | 12/18862 | 4.78E-07 | 1.15E-05 | 8.29E-06 | CSPG4/DCN/DSEL/BGN/CHST14/DSE/VCAN | 7 |
| BP | GO:0010594 | regulation of endothelial cell migration | 31/941 | 231/18862 | 5.16E-07 | 1.24E-05 | 8.90E-06 | AKT3/ANGPT1/ATP2B4/DCN/FGF1/FGFR1/ITGB3/MAP3K3/MEF2C/MEOX2/NRP1/NRP2/PRKD1/PTPRM/RHOJ/SASH1/SERPINF1/SLIT2/TEK/VEGFC/FLT4/KDR/MMRN2/PDGFB/SPARC/STC1/TBXA2R/THBS1/VASH1/APOE/TGFB1 | 31 |
| BP | GO:0061037 | negative regulation of cartilage development | 10/941 | 28/18862 | 5.25E-07 | 1.26E-05 | 9.01E-06 | EFEMP1/GREM1/NKX3-2/RARB/SNAI2/ADAMTS12/ADAMTS7/GLI2/LTBP3/PTHLH | 10 |
| BP | GO:1900047 | negative regulation of hemostasis | 13/941 | 49/18862 | 5.37E-07 | 1.28E-05 | 9.18E-06 | PDGFRA/PRKG1/SERPING1/CD34/COMP/FAP/PDGFB/PLAT/PLAU/SERPINE1/THBD/THBS1/APOE | 13 |
| BP | GO:0071219 | cellular response to molecule of bacterial origin | 29/941 | 209/18862 | 5.92E-07 | 1.40E-05 | 1.01E-05 | AXL/LDOC1/MEF2C/SASH1/VIM/SERPINE1/SIRPA/TBXA2R/TNFSF4/CCL2/CD14/CD86/FCGR2B/HAVCR2/HCK/ICAM1/LILRA2/LILRB1/LILRB2/LY86/LY96/MRC1/NLRP3/PDCD1LG2/PTAFR/TGFB1/TLR1/TLR2/TREM2 | 29 |
| BP | GO:0043114 | regulation of vascular permeability | 12/941 | 42/18862 | 6.11E-07 | 1.44E-05 | 1.03E-05 | AKAP12/ANGPT1/FERMT2/SLIT2/TEK/CDH5/GPR4/NPR1/RAMP2/TRPV4/APOE/TGFB1 | 12 |
| BP | GO:0030193 | regulation of blood coagulation | 15/941 | 66/18862 | 6.49E-07 | 1.52E-05 | 1.09E-05 | CAV1/PDGFRA/PRKG1/SERPING1/CD34/F2R/FAP/PDGFB/PLAT/PLAU/SERPINE1/TBXA2R/THBD/THBS1/APOE | 15 |
| BP | GO:0090066 | regulation of anatomical structure size | 51/941 | 493/18862 | 6.80E-07 | 1.59E-05 | 1.14E-05 | ACTA2/AKT3/AQP1/ARHGAP28/AVPR1A/CAV1/CCL21/CDC42EP3/GJA1/GJA5/HTR2B/ISLR2/ITGA1/LRRK2/MAP1B/MSN/NRP1/PLXNA4/PRKG1/RDX/RGS2/SLC8A1/SLIT2/SSH1/TMOD2/COMP/EDNRA/ELN/ESAM/F2R/FN1/KANK3/NPR1/PLEKHG2/PRR16/SEMA6B/SEMA7A/SLC12A4/TBXA2R/TRPV4/APOE/DOCK4/GMFG/HCK/HRH2/ICAM1/P2RX7/PLEK/PREX1/PTAFR/TRPV2 | 51 |
| BP | GO:0061005 | cell differentiation involved in kidney development | 14/941 | 58/18862 | 6.98E-07 | 1.62E-05 | 1.16E-05 | ACTA2/BASP1/FAT4/GLI3/GREM1/MEF2C/NOTCH2/WWTR1/CD34/GLIS2/GPR4/LAMB2/PDGFB/MMP9 | 14 |
| BP | GO:0043010 | camera-type eye development | 37/941 | 310/18862 | 8.25E-07 | 1.91E-05 | 1.37E-05 | ATP2B4/CLIC4/CYP1B1/EFEMP1/FGF10/FZD4/GLI3/MAF/MEIS1/MITF/NOTCH2/NRP1/PDGFRA/PRPH2/PTPRM/RHOJ/SDK1/SERPINF1/SMARCD3/SOX11/TENM3/TUB/VIM/ALDH1A3/ARHGEF15/COL4A1/COL8A1/COL8A2/FBN1/FLT1/INHBA/LAMB2/PDGFRB/SPRED3/THY1/TWIST1/WNT2 | 37 |
| BP | GO:0030500 | regulation of bone mineralization | 16/941 | 76/18862 | 8.36E-07 | 1.93E-05 | 1.38E-05 | DDR2/GREM1/MEF2C/MGP/OMD/S1PR1/SLC8A1/TGFB3/TMEM119/COMP/LTBP3/TWIST1/CCR1/P2RX7/SRGN/TGFB1 | 16 |
| BP | GO:0032964 | collagen biosynthetic process | 13/941 | 51/18862 | 8.88E-07 | 2.03E-05 | 1.46E-05 | ADAMTS3/TGFB3/VIM/COL1A1/COL5A1/EMILIN1/ENG/F2R/P3H3/PDGFRB/RCN3/SERPINH1/TGFB1 | 13 |
| BP | GO:0071674 | mononuclear cell migration | 27/941 | 190/18862 | 8.96E-07 | 2.04E-05 | 1.47E-05 | CCL21/CD99L2/CYP7B1/GREM1/MSN/S1PR1/SLIT2/FLT1/PDGFB/PECAM1/SERPINE1/SIRPA/THBS1/ADAM8/AIF1/C3AR1/C5AR1/CCL18/CCL2/CCR1/CSF1/CSF1R/CXCR4/ICAM1/MYO1G/PLA2G7/SLAMF8 | 27 |
| BP | GO:0060284 | regulation of cell development | 50/941 | 485/18862 | 9.68E-07 | 2.20E-05 | 1.58E-05 | DAAM2/FBLN1/FERMT2/FLNA/GLI3/IL6ST/ISLR2/LRRK2/MAP1B/NOTCH2/NPR2/NRP1/PLXNA4/RECK/S1PR3/SERPINF1/SLIT2/SMARCD3/SOX11/TRPC6/VCL/VEGFC/CDH5/EFNB3/EGR2/FBN1/FN1/GPR68/HEYL/NUMBL/PLXND1/POSTN/PTHLH/SEMA6B/SEMA7A/TENM4/THY1/WNT2/CASS4/CLEC7A/CXCR4/LILRB1/PLXNC1/PREX1/S1PR2/SLAMF8/TGFB1/TLR2/TRPV2/TYROBP | 50 |
| BP | GO:0001886 | endothelial cell morphogenesis | 7/941 | 13/18862 | 9.91E-07 | 2.23E-05 | 1.60E-05 | CLIC4/COL15A1/COL18A1/COL22A1/HEG1/NOTCH4/STC1 | 7 |
| BP | GO:0030205 | dermatan sulfate metabolic process | 7/941 | 13/18862 | 9.91E-07 | 2.23E-05 | 1.60E-05 | CSPG4/DCN/DSEL/BGN/CHST14/DSE/VCAN | 7 |
| BP | GO:0110149 | regulation of biomineralization | 18/941 | 96/18862 | 1.07E-06 | 2.40E-05 | 1.72E-05 | ASPN/DDR2/GREM1/MEF2C/MGP/OMD/S1PR1/SLC8A1/TGFB3/TMEM119/COMP/FBLN7/LTBP3/TWIST1/CCR1/P2RX7/SRGN/TGFB1 | 18 |
| BP | GO:0150115 | cell-substrate junction organization | 19/941 | 106/18862 | 1.11E-06 | 2.47E-05 | 1.78E-05 | DLC1/EPHA3/FERMT2/GREM1/ITGA5/LAMC1/NRP1/PHLDB2/TEK/TLN1/VCL/COL16A1/FN1/KDR/MMP14/PPM1F/THBS1/THSD1/THY1 | 19 |
| BP | GO:0042110 | T cell activation | 49/941 | 474/18862 | 1.15E-06 | 2.55E-05 | 1.83E-05 | BCL6/CAV1/CCL21/FYN/GLI3/HLX/IL6ST/MSN/RORA/VCAM1/EFNB3/GLI2/LGALS1/LOXL3/LRRC32/SIRPA/THY1/TNFSF4/ADAM8/AIF1/CCL2/CD300A/CD4/CD86/CLEC7A/CTSL/FCER1G/FCGR2B/FOXP3/GPNMB/HAVCR2/HLA-DOA/HLA-DPB1/ICAM1/LAPTM5/LILRB1/LILRB2/LILRB4/MAFB/NLRP3/PDCD1LG2/PIK3R6/PREX1/SIRPB1/SLC11A1/TNFAIP8L2/TNFSF13B/TNFSF14/VSIG4 | 49 |
| BP | GO:0050731 | positive regulation of peptidyl-tyrosine phosphorylation | 27/941 | 193/18862 | 1.22E-06 | 2.70E-05 | 1.94E-05 | ANGPT1/CSPG4/FGF10/FGF7/FYN/GREM1/HGF/IL6ST/ITGA5/ITGB3/NRP1/THBS4/DLG4/GPRC5B/NOX4/PDGFB/PECAM1/CASS4/CD4/CLEC7A/CSF1R/FCGR1A/ICAM1/LILRA5/SOCS3/TGFB1/TREM2 | 27 |
| BP | GO:0070664 | negative regulation of leukocyte proliferation | 17/941 | 88/18862 | 1.37E-06 | 3.02E-05 | 2.17E-05 | GREM1/SOX11/LRRC32/CD300A/CD86/FCGR2B/FOXP3/GPNMB/HAVCR2/LILRB1/LILRB2/LILRB4/LST1/MNDA/PDCD1LG2/TYROBP/VSIG4 | 17 |
| BP | GO:0045667 | regulation of osteoblast differentiation | 21/941 | 128/18862 | 1.39E-06 | 3.05E-05 | 2.19E-05 | DDR2/FERMT2/GLI1/GLI3/GREM1/HGF/IGFBP5/IL6ST/MEF2C/PDLIM7/PRKD1/SFRP2/SNAI2/SOX11/TMEM119/TWIST2/VEGFC/WWTR1/CHRD/CTHRC1/TWIST1 | 21 |
| BP | GO:0003179 | heart valve morphogenesis | 13/941 | 53/18862 | 1.43E-06 | 3.12E-05 | 2.24E-05 | GJA5/MEF2C/NOTCH2/SLIT2/SNAI2/DCHS1/ELN/EMILIN1/HEYL/TIE1/TWIST1/NFATC1/TGFB1 | 13 |
| BP | GO:0030166 | proteoglycan biosynthetic process | 14/941 | 62/18862 | 1.66E-06 | 3.60E-05 | 2.59E-05 | CHST15/CHST3/CSGALNACT2/CSPG4/DCN/DSEL/BGN/CHST14/CHSY3/DSE/VCAN/CHST11/CYTL1/HS3ST3B1 | 14 |
| BP | GO:0050818 | regulation of coagulation | 15/941 | 71/18862 | 1.75E-06 | 3.78E-05 | 2.71E-05 | CAV1/PDGFRA/PRKG1/SERPING1/CD34/F2R/FAP/PDGFB/PLAT/PLAU/SERPINE1/TBXA2R/THBD/THBS1/APOE | 15 |
| BP | GO:0071216 | cellular response to biotic stimulus | 30/941 | 233/18862 | 1.89E-06 | 4.06E-05 | 2.91E-05 | AXL/LDOC1/MEF2C/SASH1/VIM/SERPINE1/SIRPA/TBXA2R/TNFSF4/CCL2/CD14/CD86/CLEC7A/FCGR2B/HAVCR2/HCK/ICAM1/LILRA2/LILRB1/LILRB2/LY86/LY96/MRC1/NLRP3/PDCD1LG2/PTAFR/TGFB1/TLR1/TLR2/TREM2 | 30 |
| BP | GO:0010717 | regulation of epithelial to mesenchymal transition | 18/941 | 100/18862 | 1.99E-06 | 4.26E-05 | 3.05E-05 | EPHA3/FERMT2/GREM1/PHLDB1/PHLDB2/SFRP2/TGFB1I1/TGFB3/WWTR1/COL1A1/ENG/LOXL2/PDPN/SPRED3/TWIST1/VASN/GLIPR2/TGFB1 | 18 |
| BP | GO:0045670 | regulation of osteoclast differentiation | 14/941 | 63/18862 | 2.04E-06 | 4.32E-05 | 3.10E-05 | LRRC17/MITF/NOTCH2/FBN1/FSTL3/GPR68/CCR1/CSF1/LILRB1/LILRB3/LILRB4/MAFB/TREM2/TYROBP | 14 |
| BP | GO:0014910 | regulation of smooth muscle cell migration | 16/941 | 81/18862 | 2.05E-06 | 4.32E-05 | 3.10E-05 | ADAMTS1/IGFBP5/MEF2C/NRP1/PRKG1/SLIT2/SSH1/IGFBP3/NOX4/PDGFB/PDGFRB/PLAU/POSTN/SERPINE1/AIF1/DOCK4 | 16 |
| BP | GO:0050672 | negative regulation of lymphocyte proliferation | 16/941 | 81/18862 | 2.05E-06 | 4.32E-05 | 3.10E-05 | SOX11/LRRC32/CD300A/CD86/FCGR2B/FOXP3/GPNMB/HAVCR2/LILRB1/LILRB2/LILRB4/LST1/MNDA/PDCD1LG2/TYROBP/VSIG4 | 16 |
| BP | GO:0061041 | regulation of wound healing | 21/941 | 131/18862 | 2.05E-06 | 4.32E-05 | 3.10E-05 | CAV1/FERMT2/GJA1/ITGB1/PDGFRA/PHLDB2/PRKG1/SERPING1/CD34/F2R/FAP/PDGFB/PLAT/PLAU/SERPINE1/TBXA2R/THBD/THBS1/APOE/CLEC7A/CXCR4 | 21 |
| BP | GO:1903555 | regulation of tumor necrosis factor superfamily cytokine production | 24/941 | 164/18862 | 2.11E-06 | 4.42E-05 | 3.17E-05 | ANGPT1/AXL/SYT11/CD34/SIRPA/THBS1/TWIST1/ADAM8/C5AR2/CD14/CD33/CD86/CLEC7A/FOXP3/HAVCR2/LILRA2/LILRA5/LILRB1/LY96/PTAFR/TLR1/TLR2/TREM2/TYROBP | 24 |
| BP | GO:0032970 | regulation of actin filament-based process | 42/941 | 389/18862 | 2.21E-06 | 4.61E-05 | 3.31E-05 | ARHGAP28/CAV1/CCDC88A/CCL21/CDC42EP3/DLC1/EPHA3/FERMT2/FLNA/GJA5/JAM3/NOTCH2/NRP1/PDGFRA/PHLDB2/RDX/RGS4/RHOQ/S1PR1/SLIT2/SSH1/SYNPO/TEK/TGFB3/TMOD2/ARHGEF15/ELN/ESAM/KANK3/NOX4/PDGFRB/PDPN/PLEKHG2/PPM1F/STC1/CSF1R/FES/GMFG/HCK/ICAM1/PLEK/PREX1 | 42 |
| BP | GO:0022612 | gland morphogenesis | 19/941 | 111/18862 | 2.29E-06 | 4.75E-05 | 3.41E-05 | CAV1/CYP7B1/FGF1/FGF10/FGF7/GLI1/GLI3/HGF/IGFBP5/MSN/NOTCH2/TGFB3/TNC/GLI2/PLXND1/SULF1/CSF1/CSF1R/TGFB1 | 19 |
| BP | GO:1902106 | negative regulation of leukocyte differentiation | 18/941 | 101/18862 | 2.31E-06 | 4.77E-05 | 3.43E-05 | BCL6/GLI3/HLX/LRRC17/FBN1/FSTL3/GPR68/INHBA/LOXL3/TNFSF4/ZBTB46/C1QC/FCGR2B/FOXP3/LILRB1/LILRB3/LILRB4/MAFB | 18 |
| BP | GO:0030001 | metal ion transport | 49/941 | 486/18862 | 2.35E-06 | 4.84E-05 | 3.47E-05 | ANXA6/ATP2B4/CACNA2D1/CALCRL/CAV1/CCL21/FYN/GEM/GJA1/HTR2B/ITPR1/PKD2/REM1/RGS4/SLC24A3/SLC8A1/TRPC1/TRPC4/TRPC6/TUSC3/APLNR/EHD3/F2R/F2RL3/GJA4/GNAI2/KCNE4/PDGFB/PDGFRB/RAMP2/RAMP3/STC1/THY1/TRPV4/CCL2/CCR1/CD33/CD4/CD84/CXCR4/ICAM1/LILRA2/LILRA5/LILRB1/LILRB2/P2RX7/P2RY6/SLC11A1/TRPV2 | 49 |
| BP | GO:0032945 | negative regulation of mononuclear cell proliferation | 16/941 | 82/18862 | 2.43E-06 | 4.99E-05 | 3.58E-05 | SOX11/LRRC32/CD300A/CD86/FCGR2B/FOXP3/GPNMB/HAVCR2/LILRB1/LILRB2/LILRB4/LST1/MNDA/PDCD1LG2/TYROBP/VSIG4 | 16 |
| BP | GO:0051893 | regulation of focal adhesion assembly | 14/941 | 64/18862 | 2.49E-06 | 5.05E-05 | 3.63E-05 | DLC1/EPHA3/FERMT2/GREM1/NRP1/PHLDB2/TEK/VCL/COL16A1/KDR/MMP14/PPM1F/THBS1/THY1 | 14 |
| BP | GO:0090109 | regulation of cell-substrate junction assembly | 14/941 | 64/18862 | 2.49E-06 | 5.05E-05 | 3.63E-05 | DLC1/EPHA3/FERMT2/GREM1/NRP1/PHLDB2/TEK/VCL/COL16A1/KDR/MMP14/PPM1F/THBS1/THY1 | 14 |
| BP | GO:0030178 | negative regulation of Wnt signaling pathway | 28/941 | 212/18862 | 2.49E-06 | 5.05E-05 | 3.63E-05 | ANKRD6/APCDD1L/BICC1/CAV1/CDH2/FZD4/GLI1/GLI3/GREM1/IGFBP6/LATS2/MCC/NXN/PRICKLE1/RBMS3/ROR2/SFRP2/SNAI2/WWTR1/ALPK2/CTHRC1/DACT1/DKK3/HIC1/SFRP4/SOX17/APOE/NFATC1 | 28 |
| BP | GO:0003151 | outflow tract morphogenesis | 15/941 | 73/18862 | 2.54E-06 | 5.12E-05 | 3.67E-05 | GJA5/MEF2C/NRP1/NRP2/PARVA/RARB/SFRP2/SOX11/ZFPM2/ELN/ENG/HEYL/PLXND1/SOX17/TWIST1 | 15 |
| BP | GO:0030278 | regulation of ossification | 19/941 | 112/18862 | 2.63E-06 | 5.28E-05 | 3.79E-05 | DDR2/GREM1/MEF2C/MGP/OMD/S1PR1/SLC8A1/SOX11/TGFB3/TMEM119/COMP/EGR2/LTBP3/TWIST1/CCR1/CSF1/P2RX7/SRGN/TGFB1 | 19 |
| BP | GO:0022408 | negative regulation of cell-cell adhesion | 26/941 | 189/18862 | 2.64E-06 | 5.28E-05 | 3.79E-05 | BCL6/CCL21/GLI3/HLX/PRKG1/RDX/LOXL3/LRRC32/NOTCH4/PPM1F/TNFSF4/TRPV4/CD300A/CD86/FCGR2B/FOXP3/GPNMB/HAVCR2/LAPTM5/LILRB1/LILRB2/LILRB4/PDCD1LG2/TGFB1/TNFAIP8L2/VSIG4 | 26 |
| BP | GO:0001945 | lymph vessel development | 9/941 | 26/18862 | 2.67E-06 | 5.32E-05 | 3.82E-05 | PTPN14/VEGFC/CLEC14A/FLT4/HEG1/PDPN/TIE1/TMEM204/VASH1 | 9 |
| BP | GO:0150146 | cell junction disassembly | 8/941 | 20/18862 | 2.74E-06 | 5.45E-05 | 3.91E-05 | SNAI2/TGFB3/C3/C1QA/C1QB/C1QC/ITGAM/TREM2 | 8 |
| BP | GO:1990266 | neutrophil migration | 20/941 | 123/18862 | 2.85E-06 | 5.64E-05 | 4.05E-05 | CCL21/CD99L2/IL1R1/ITGA1/JAM3/SLIT2/THBS4/PECAM1/ADAM8/C3AR1/C5AR1/C5AR2/CCL18/CCL2/CSF3R/FCER1G/ITGB2/PREX1/S100A8/SLAMF8 | 20 |
| BP | GO:0030195 | negative regulation of blood coagulation | 12/941 | 48/18862 | 2.92E-06 | 5.66E-05 | 4.07E-05 | PDGFRA/PRKG1/SERPING1/CD34/FAP/PDGFB/PLAT/PLAU/SERPINE1/THBD/THBS1/APOE | 12 |
| BP | GO:0035850 | epithelial cell differentiation involved in kidney development | 12/941 | 48/18862 | 2.92E-06 | 5.66E-05 | 4.07E-05 | ACTA2/BASP1/FAT4/GREM1/MEF2C/NOTCH2/WWTR1/CD34/GPR4/LAMB2/PDGFB/MMP9 | 12 |
| BP | GO:0071706 | tumor necrosis factor superfamily cytokine production | 24/941 | 167/18862 | 2.92E-06 | 5.66E-05 | 4.07E-05 | ANGPT1/AXL/SYT11/CD34/SIRPA/THBS1/TWIST1/ADAM8/C5AR2/CD14/CD33/CD86/CLEC7A/FOXP3/HAVCR2/LILRA2/LILRA5/LILRB1/LY96/PTAFR/TLR1/TLR2/TREM2/TYROBP | 24 |
| BP | GO:0071772 | response to BMP | 24/941 | 167/18862 | 2.92E-06 | 5.66E-05 | 4.07E-05 | FST/GDF6/GREM1/NOTCH2/ROR2/SFRP2/SOX11/TGFB3/ADAMTS12/ADAMTS7/BMP8A/CDH5/CHRD/COMP/ENG/FBN1/FSTL1/FSTL3/HEYL/HTRA1/HTRA3/SFRP4/SULF1/TGFB1 | 24 |
| BP | GO:0071773 | cellular response to BMP stimulus | 24/941 | 167/18862 | 2.92E-06 | 5.66E-05 | 4.07E-05 | FST/GDF6/GREM1/NOTCH2/ROR2/SFRP2/SOX11/TGFB3/ADAMTS12/ADAMTS7/BMP8A/CDH5/CHRD/COMP/ENG/FBN1/FSTL1/FSTL3/HEYL/HTRA1/HTRA3/SFRP4/SULF1/TGFB1 | 24 |
| BP | GO:0035633 | maintenance of blood-brain barrier | 10/941 | 33/18862 | 2.95E-06 | 5.69E-05 | 4.09E-05 | ANGPT1/GJA1/ITGB1/JAM3/LAMA2/LAMC1/VCL/CDH5/ESAM/PECAM1 | 10 |
| BP | GO:0007369 | gastrulation | 25/941 | 179/18862 | 3.10E-06 | 5.96E-05 | 4.28E-05 | GJA1/ITGA5/ITGB1/ITGB3/PHLDB1/PHLDB2/SFRP2/SOX7/APLNR/COL11A1/COL12A1/COL4A2/COL5A1/COL5A2/COL6A1/COL7A1/COL8A1/FN1/INHBA/MMP14/MMP2/SOX17/TENM4/ITGB2/MMP9 | 25 |
| BP | GO:0016525 | negative regulation of angiogenesis | 22/941 | 146/18862 | 3.40E-06 | 6.52E-05 | 4.68E-05 | ADAMTS1/ATP2B4/DCN/ECSCR/HSPG2/PTPRM/SERPINF1/TEK/THBS4/COL4A2/EMILIN1/GPR4/ISM1/NPR1/SERPINE1/SPARC/SULF1/THBS1/THBS2/TIE1/VASH1/STAB1 | 22 |
| BP | GO:0002697 | regulation of immune effector process | 47/941 | 465/18862 | 3.52E-06 | 6.71E-05 | 4.82E-05 | A2M/ANGPT1/BCL6/C1R/C1S/C4B/CADM1/CFH/HLX/IL1R1/SERPING1/TGFB3/C3/GPRC5B/HTRA1/LOXL3/SEMA7A/TNFSF4/C1QA/C1QB/C1QC/C3AR1/C5AR1/C5AR2/CD300A/CD84/CD86/CLEC7A/FCGR2B/FES/FGR/FOXP3/HAVCR2/ICAM1/ITGAM/ITGB2/LAPTM5/LILRB1/LILRB4/NLRP3/PIK3R6/PRAM1/PTAFR/SLAMF8/TGFB1/TYROBP/VSIG4 | 47 |
| BP | GO:0070167 | regulation of biomineral tissue development | 17/941 | 94/18862 | 3.56E-06 | 6.76E-05 | 4.85E-05 | ASPN/DDR2/GREM1/MEF2C/MGP/OMD/S1PR1/SLC8A1/TGFB3/TMEM119/COMP/LTBP3/TWIST1/CCR1/P2RX7/SRGN/TGFB1 | 17 |
| BP | GO:1905314 | semi-lunar valve development | 11/941 | 41/18862 | 3.57E-06 | 6.77E-05 | 4.86E-05 | GJA5/NOTCH2/SLIT2/SNAI2/ELN/EMILIN1/HEYL/TIE1/TWIST1/NFATC1/TGFB1 | 11 |
| BP | GO:0034332 | adherens junction organization | 14/941 | 66/18862 | 3.67E-06 | 6.92E-05 | 4.97E-05 | CADM1/CDH2/FERMT2/JAM3/RDX/VCL/CDH11/CDH13/CDH5/CDH6/DCHS1/KIFC3/NUMBL/RAMP2 | 14 |
| BP | GO:1902903 | regulation of supramolecular fiber organization | 40/941 | 370/18862 | 3.72E-06 | 6.99E-05 | 5.02E-05 | ARHGAP28/CCDC88A/CCL21/CDC42EP3/CLIP3/DLC1/FERMT2/FLNA/MAP1A/MAP1B/NAV3/NRP1/PHLDB2/RDX/RGS4/S1PR1/SLIT2/SSH1/SYNPO/TGFB3/TMOD2/AEBP1/ARHGEF15/CDH5/EFEMP2/ELN/EMILIN1/ESAM/KANK3/NOX4/PLEKHG2/PPM1F/TRPV4/APOE/FES/GMFG/HCK/ICAM1/PLEK/PREX1 | 40 |
| BP | GO:0032675 | regulation of interleukin-6 production | 23/941 | 158/18862 | 3.75E-06 | 7.01E-05 | 5.03E-05 | C1QTNF3/HGF/SYT11/F2R/SIRPA/TNFSF4/TWIST1/AIF1/C5AR2/CLEC7A/FOXP3/HAVCR2/LILRA2/LILRA5/LILRB2/LILRB4/PTAFR/RAB7B/TLR1/TLR2/TLR8/TREM2/TYROBP | 23 |
| BP | GO:0003206 | cardiac chamber morphogenesis | 19/941 | 115/18862 | 3.94E-06 | 7.35E-05 | 5.27E-05 | ADAMTS1/MEF2C/NOTCH2/NRP1/NRP2/PARVA/RARB/SFRP2/SLIT2/SMARCD3/SOX11/TEK/ZFPM2/COL11A1/ENG/HEG1/HEYL/WNT2/TGFB1 | 19 |
| BP | GO:1903707 | negative regulation of hemopoiesis | 18/941 | 105/18862 | 4.11E-06 | 7.62E-05 | 5.47E-05 | BCL6/GLI3/HLX/LRRC17/FBN1/FSTL3/GPR68/INHBA/LOXL3/TNFSF4/ZBTB46/C1QC/FCGR2B/FOXP3/LILRB1/LILRB3/LILRB4/MAFB | 18 |
| BP | GO:2000181 | negative regulation of blood vessel morphogenesis | 22/941 | 148/18862 | 4.28E-06 | 7.91E-05 | 5.68E-05 | ADAMTS1/ATP2B4/DCN/ECSCR/HSPG2/PTPRM/SERPINF1/TEK/THBS4/COL4A2/EMILIN1/GPR4/ISM1/NPR1/SERPINE1/SPARC/SULF1/THBS1/THBS2/TIE1/VASH1/STAB1 | 22 |
| BP | GO:0060070 | canonical Wnt signaling pathway | 37/941 | 333/18862 | 4.55E-06 | 8.39E-05 | 6.02E-05 | ANKRD6/BICC1/CAV1/CDH2/CDK14/DAAM2/FGF10/FZD1/FZD4/GLI1/GLI3/GREM1/IGFBP6/LATS2/LRRK2/MCC/MITF/PRICKLE1/PYGO1/RBMS3/RECK/ROR2/RSPO3/SFRP2/SNAI2/SOX7/WWTR1/COL1A1/CTHRC1/DACT1/DKK3/GPRC5B/SFRP4/SOX17/WNT2/APOE/TGFB1 | 37 |
| BP | GO:0043405 | regulation of MAP kinase activity | 35/941 | 307/18862 | 4.62E-06 | 8.43E-05 | 6.05E-05 | CAV1/CSPG4/FGF1/FGF10/FGFR1/FZD4/HGF/HTR2B/IGFBP6/ITGA1/LRRK2/MDFIC/PDGFC/RGS2/RGS4/ROR2/SASH1/SFRP2/TGFB3/FLT1/MAP3K12/PDGFB/PDGFRB/PEA15/SPRED3/THBS1/ADAM8/APOE/C5AR1/CD300A/CXCR4/FPR1/PIK3R6/S1PR2/TGFB1 | 35 |
| BP | GO:0071604 | transforming growth factor beta production | 11/941 | 42/18862 | 4.63E-06 | 8.43E-05 | 6.05E-05 | LTBP1/CD34/FN1/LRRC32/LTBP3/LUM/THBS1/FOXP3/LILRB1/NRROS/TYROBP | 11 |
| BP | GO:1902622 | regulation of neutrophil migration | 11/941 | 42/18862 | 4.63E-06 | 8.43E-05 | 6.05E-05 | CCL21/CD99L2/IL1R1/JAM3/SLIT2/THBS4/ADAM8/C3AR1/C5AR1/C5AR2/SLAMF8 | 11 |
| BP | GO:0014066 | regulation of phosphatidylinositol 3-kinase signaling | 20/941 | 127/18862 | 4.73E-06 | 8.52E-05 | 6.12E-05 | ANGPT1/DCN/FGFR1/FYN/HGF/PDGFC/PDGFRA/ROR2/TEK/F2R/FLT1/FN1/KDR/PDGFB/PDGFRB/SELP/TWIST1/UNC5B/FGR/HCST | 20 |
| BP | GO:0035296 | regulation of tube diameter | 20/941 | 127/18862 | 4.73E-06 | 8.52E-05 | 6.12E-05 | ACTA2/AVPR1A/CAV1/GJA1/GJA5/HTR2B/ITGA1/PRKG1/RGS2/SLC8A1/COMP/EDNRA/F2R/NPR1/TBXA2R/APOE/DOCK4/HRH2/ICAM1/PTAFR | 20 |
| BP | GO:0097746 | blood vessel diameter maintenance | 20/941 | 127/18862 | 4.73E-06 | 8.52E-05 | 6.12E-05 | ACTA2/AVPR1A/CAV1/GJA1/GJA5/HTR2B/ITGA1/PRKG1/RGS2/SLC8A1/COMP/EDNRA/F2R/NPR1/TBXA2R/APOE/DOCK4/HRH2/ICAM1/PTAFR | 20 |
| BP | GO:1901343 | negative regulation of vasculature development | 22/941 | 149/18862 | 4.78E-06 | 8.59E-05 | 6.17E-05 | ADAMTS1/ATP2B4/DCN/ECSCR/HSPG2/PTPRM/SERPINF1/TEK/THBS4/COL4A2/EMILIN1/GPR4/ISM1/NPR1/SERPINE1/SPARC/SULF1/THBS1/THBS2/TIE1/VASH1/STAB1 | 22 |
| BP | GO:0002040 | sprouting angiogenesis | 25/941 | 184/18862 | 5.12E-06 | 9.15E-05 | 6.57E-05 | AKT3/ANGPT1/FGF1/GREM1/ITGA5/MAP3K3/MEOX2/NRP1/PARVA/RECK/RHOJ/RSPO3/SLIT2/TEK/VEGFC/APLNR/CDH13/CLEC14A/FLT4/KDR/LOXL2/MMRN2/RAMP2/TBXA2R/THBS1 | 25 |
| BP | GO:0048017 | inositol lipid-mediated signaling | 26/941 | 196/18862 | 5.21E-06 | 9.28E-05 | 6.66E-05 | ANGPT1/DCN/FGFR1/FYN/HGF/HTR2B/NPR3/PDGFC/PDGFRA/PREX2/ROR2/TEK/F2R/FLT1/FN1/KDR/PDGFB/PDGFRB/SELP/TWIST1/UNC5B/ADAP2/CSF1R/FGR/HCST/PTAFR | 26 |
| BP | GO:0035150 | regulation of tube size | 20/941 | 128/18862 | 5.34E-06 | 9.49E-05 | 6.81E-05 | ACTA2/AVPR1A/CAV1/GJA1/GJA5/HTR2B/ITGA1/PRKG1/RGS2/SLC8A1/COMP/EDNRA/F2R/NPR1/TBXA2R/APOE/DOCK4/HRH2/ICAM1/PTAFR | 20 |
| BP | GO:0010575 | positive regulation of vascular endothelial growth factor production | 9/941 | 28/18862 | 5.39E-06 | 9.54E-05 | 6.85E-05 | CYP1B1/IL6ST/RORA/C3/FLT4/SULF1/C3AR1/C5AR1/TGFB1 | 9 |
| BP | GO:0098883 | synapse pruning | 6/941 | 11/18862 | 5.65E-06 | 9.93E-05 | 7.12E-05 | C3/C1QA/C1QB/C1QC/ITGAM/TREM2 | 6 |
| BP | GO:1902563 | regulation of neutrophil activation | 6/941 | 11/18862 | 5.65E-06 | 9.93E-05 | 7.12E-05 | CD300A/FCGR2B/ITGAM/ITGB2/PRAM1/PTAFR | 6 |
| BP | GO:0071222 | cellular response to lipopolysaccharide | 26/941 | 197/18862 | 5.72E-06 | 0.0001 | 7.19E-05 | AXL/LDOC1/MEF2C/SASH1/VIM/SERPINE1/SIRPA/TBXA2R/TNFSF4/CCL2/CD14/CD86/HAVCR2/HCK/ICAM1/LILRA2/LILRB1/LILRB2/LY86/LY96/MRC1/NLRP3/PDCD1LG2/PTAFR/TGFB1/TLR2 | 26 |
| BP | GO:0032635 | interleukin-6 production | 23/941 | 162/18862 | 5.76E-06 | 0.0001 | 7.21E-05 | C1QTNF3/HGF/SYT11/F2R/SIRPA/TNFSF4/TWIST1/AIF1/C5AR2/CLEC7A/FOXP3/HAVCR2/LILRA2/LILRA5/LILRB2/LILRB4/PTAFR/RAB7B/TLR1/TLR2/TLR8/TREM2/TYROBP | 23 |
| BP | GO:0016339 | calcium-dependent cell-cell adhesion via plasma membrane cell adhesion molecules | 11/941 | 43/18862 | 5.94E-06 | 0.000103 | 7.41E-05 | CDH2/PCDHB4/PCDHB5/PCDHGC3/CDH11/CDH13/CDH5/CDH6/DCHS1/PCDH12/SELP | 11 |
| BP | GO:0001570 | vasculogenesis | 15/941 | 78/18862 | 6.03E-06 | 0.000104 | 7.50E-05 | CAV1/FZD4/GJC1/QKI/ZFPM2/APLNR/CD34/ENG/HEG1/KDR/PDGFRB/RAMP2/SOX17/TIE1/TGFB1 | 15 |
| BP | GO:0001938 | positive regulation of endothelial cell proliferation | 18/941 | 108/18862 | 6.20E-06 | 0.000107 | 7.68E-05 | AKT3/CAV2/FGFR1/HTR2B/ITGB3/NRP1/NRP2/PRKD1/TEK/THBS4/TNFSF12/VEGFC/APLNR/CDH13/FLT4/KDR/PDGFB/WNT2 | 18 |
| BP | GO:0010518 | positive regulation of phospholipase activity | 13/941 | 60/18862 | 6.35E-06 | 0.000109 | 7.81E-05 | AVPR1A/FGFR1/HTR2B/PDGFRA/EDNRA/FLT1/PDGFRB/SELE/C5AR1/CD86/P2RY6/PTAFR/RASGRP4 | 13 |
| BP | GO:0032653 | regulation of interleukin-10 production | 13/941 | 60/18862 | 6.35E-06 | 0.000109 | 7.81E-05 | HGF/CD34/TNFSF4/CLEC7A/FCGR2B/FOXP3/LILRA5/LILRB1/LILRB4/PDCD1LG2/TLR2/TREM2/TYROBP | 13 |
| BP | GO:0150116 | regulation of cell-substrate junction organization | 14/941 | 69/18862 | 6.38E-06 | 0.000109 | 7.82E-05 | DLC1/EPHA3/FERMT2/GREM1/NRP1/PHLDB2/TEK/VCL/COL16A1/KDR/MMP14/PPM1F/THBS1/THY1 | 14 |
| BP | GO:0002688 | regulation of leukocyte chemotaxis | 19/941 | 119/18862 | 6.61E-06 | 0.000112 | 8.01E-05 | CCL21/GREM1/JAM3/SLIT2/THBS4/VEGFC/SERPINE1/THBS1/AIF1/C3AR1/C5AR1/C5AR2/CCL2/CCR1/CSF1/CSF1R/GPSM3/PLA2G7/SLAMF8 | 19 |
| BP | GO:0050868 | negative regulation of T cell activation | 19/941 | 119/18862 | 6.61E-06 | 0.000112 | 8.01E-05 | BCL6/GLI3/HLX/LOXL3/LRRC32/TNFSF4/CD300A/CD86/FCGR2B/FOXP3/GPNMB/HAVCR2/LAPTM5/LILRB1/LILRB2/LILRB4/PDCD1LG2/TNFAIP8L2/VSIG4 | 19 |
| BP | GO:0070661 | leukocyte proliferation | 35/941 | 312/18862 | 6.63E-06 | 0.000112 | 8.01E-05 | BCL6/FGF10/FYN/GREM1/IL6ST/MEF2C/MSN/NPR3/SOX11/VCAM1/LRRC32/TNFSF4/AIF1/CD300A/CD4/CD86/CSF1/CSF1R/FCGR2B/FOXP3/GPNMB/HAVCR2/HLA-DPB1/LILRB1/LILRB2/LILRB4/LST1/MNDA/PDCD1LG2/SLC11A1/TNFSF13B/TNFSF14/TREM2/TYROBP/VSIG4 | 35 |
| BP | GO:0010634 | positive regulation of epithelial cell migration | 24/941 | 175/18862 | 6.68E-06 | 0.000112 | 8.01E-05 | AKT3/ANGPT1/FGF1/FGF10/FGF7/FGFR1/ITGB3/MAP3K3/NRP1/NRP2/PRKD1/RHOJ/SASH1/TEK/VEGFC/FLT4/KDR/PDGFB/PPM1F/SPARC/THBS1/GLIPR2/MMP9/TGFB1 | 24 |
| BP | GO:0051897 | positive regulation of protein kinase B signaling | 24/941 | 175/18862 | 6.68E-06 | 0.000112 | 8.01E-05 | ANGPT1/AXL/CCL21/FERMT2/FGF1/FGF10/FGF7/FGFR1/FYN/HGF/IGFBP5/ITGB1/PDGFRA/TEK/TSPYL5/ENG/PDGFB/PDGFRB/RAMP3/THBS1/ADAM8/CASS4/CD86/TGFB1 | 24 |
| BP | GO:0006936 | muscle contraction | 38/941 | 352/18862 | 6.69E-06 | 0.000112 | 8.01E-05 | ACTA2/ANXA6/ATP2B4/CACNA2D1/CALCRL/CALD1/CAV1/FLNA/GJA1/GJA5/GJC1/HTR2B/ITGA1/KCNJ8/PRKG1/PTGER3/RGS2/SCN1B/SLC8A1/SSPN/TLN1/TMOD2/VCL/VIM/COMP/DYSF/EDNRA/EHD3/F2R/KCNE4/STC1/SULF1/TBXA2R/CXCR4/DOCK4/KCNJ5/PTAFR/SPHK1 | 38 |
| BP | GO:0032956 | regulation of actin cytoskeleton organization | 38/941 | 352/18862 | 6.69E-06 | 0.000112 | 8.01E-05 | ARHGAP28/CCDC88A/CCL21/CDC42EP3/DLC1/EPHA3/FERMT2/FLNA/JAM3/NOTCH2/NRP1/PDGFRA/PHLDB2/RDX/RGS4/RHOQ/S1PR1/SLIT2/SSH1/SYNPO/TEK/TGFB3/TMOD2/ARHGEF15/ELN/ESAM/KANK3/NOX4/PDGFRB/PLEKHG2/PPM1F/CSF1R/FES/GMFG/HCK/ICAM1/PLEK/PREX1 | 38 |
| BP | GO:1903034 | regulation of response to wounding | 23/941 | 164/18862 | 7.09E-06 | 0.000118 | 8.45E-05 | CAV1/FERMT2/FLNA/GJA1/ITGB1/PDGFRA/PHLDB2/PRKG1/SERPING1/CD34/F2R/FAP/PDGFB/PLAT/PLAU/SERPINE1/TBXA2R/THBD/THBS1/APOE/CLEC7A/CXCR4/SCARF1 | 23 |
| BP | GO:0042310 | vasoconstriction | 15/941 | 79/18862 | 7.11E-06 | 0.000118 | 8.45E-05 | ACTA2/AVPR1A/CAV1/GJA1/GJA5/HTR2B/SLC8A1/COMP/EDNRA/F2R/TBXA2R/DOCK4/HRH2/ICAM1/PTAFR | 15 |
| BP | GO:0060828 | regulation of canonical Wnt signaling pathway | 33/941 | 287/18862 | 7.17E-06 | 0.000118 | 8.49E-05 | ANKRD6/BICC1/CAV1/CDH2/CDK14/DAAM2/FGF10/FZD4/GLI1/GLI3/GREM1/IGFBP6/LATS2/LRRK2/MCC/PRICKLE1/RBMS3/RECK/ROR2/RSPO3/SFRP2/SNAI2/SOX7/WWTR1/COL1A1/CTHRC1/DACT1/DKK3/GPRC5B/SFRP4/SOX17/APOE/TGFB1 | 33 |
| BP | GO:0050819 | negative regulation of coagulation | 12/941 | 52/18862 | 7.19E-06 | 0.000118 | 8.49E-05 | PDGFRA/PRKG1/SERPING1/CD34/FAP/PDGFB/PLAT/PLAU/SERPINE1/THBD/THBS1/APOE | 12 |
| BP | GO:0010517 | regulation of phospholipase activity | 14/941 | 70/18862 | 7.61E-06 | 0.000125 | 8.95E-05 | AVPR1A/FGFR1/HTR2B/PDGFRA/RGS2/EDNRA/FLT1/PDGFRB/SELE/C5AR1/CD86/P2RY6/PTAFR/RASGRP4 | 14 |
| BP | GO:0003170 | heart valve development | 13/941 | 61/18862 | 7.70E-06 | 0.000126 | 9.04E-05 | GJA5/MEF2C/NOTCH2/SLIT2/SNAI2/DCHS1/ELN/EMILIN1/HEYL/TIE1/TWIST1/NFATC1/TGFB1 | 13 |
| BP | GO:0014065 | phosphatidylinositol 3-kinase signaling | 22/941 | 154/18862 | 8.25E-06 | 0.000134 | 9.65E-05 | ANGPT1/DCN/FGFR1/FYN/HGF/HTR2B/PDGFC/PDGFRA/PREX2/ROR2/TEK/F2R/FLT1/FN1/KDR/PDGFB/PDGFRB/SELP/TWIST1/UNC5B/FGR/HCST | 22 |
| BP | GO:0006939 | smooth muscle contraction | 17/941 | 100/18862 | 8.48E-06 | 0.000138 | 9.88E-05 | ACTA2/ATP2B4/CALCRL/CAV1/HTR2B/PRKG1/PTGER3/RGS2/SLC8A1/COMP/EDNRA/F2R/SULF1/TBXA2R/DOCK4/PTAFR/SPHK1 | 17 |
| BP | GO:0051251 | positive regulation of lymphocyte activation | 38/941 | 356/18862 | 8.70E-06 | 0.000141 | 0.000101 | AXL/BCL6/CAV1/CCL21/FGF10/FYN/GLI3/HLX/IL6ST/MEF2C/VCAM1/EFNB3/GLI2/LGALS1/MMP14/SIRPA/THY1/TNFSF4/ADAM8/AIF1/CCL2/CD4/CD86/CLEC7A/FOXP3/HAVCR2/HLA-DPB1/LILRB1/LILRB2/LILRB4/NLRP3/PDCD1LG2/PIK3R6/SIRPB1/TGFB1/TNFSF13B/TNFSF14/TYROBP | 38 |
| BP | GO:0032613 | interleukin-10 production | 13/941 | 62/18862 | 9.31E-06 | 0.00015 | 0.000108 | HGF/CD34/TNFSF4/CLEC7A/FCGR2B/FOXP3/LILRA5/LILRB1/LILRB4/PDCD1LG2/TLR2/TREM2/TYROBP | 13 |
| BP | GO:0001946 | lymphangiogenesis | 7/941 | 17/18862 | 9.42E-06 | 0.000151 | 0.000109 | PTPN14/VEGFC/CLEC14A/FLT4/PDPN/TIE1/VASH1 | 7 |
| BP | GO:0070663 | regulation of leukocyte proliferation | 29/941 | 241/18862 | 1.06E-05 | 0.00017 | 0.000122 | BCL6/FGF10/GREM1/IL6ST/MEF2C/SOX11/VCAM1/LRRC32/TNFSF4/AIF1/CD300A/CD4/CD86/CSF1/CSF1R/FCGR2B/FOXP3/GPNMB/HAVCR2/HLA-DPB1/LILRB1/LILRB2/LILRB4/LST1/MNDA/PDCD1LG2/TNFSF13B/TYROBP/VSIG4 | 29 |
| BP | GO:0032490 | detection of molecule of bacterial origin | 6/941 | 12/18862 | 1.08E-05 | 0.000173 | 0.000124 | C4B/SSC5D/LY96/TLR1/TLR2/TREM2 | 6 |
| BP | GO:0090101 | negative regulation of transmembrane receptor protein serine/threonine kinase signaling pathway | 20/941 | 134/18862 | 1.08E-05 | 0.000173 | 0.000124 | ASPN/CAV1/CAV2/FST/GREM1/LTBP1/SFRP2/TGFB1I1/TGFB3/CHRD/EMILIN1/FBN1/FSTL3/HTRA1/HTRA3/SPRED3/VASN/CHST11/NRROS/TGFB1 | 20 |
| BP | GO:0048015 | phosphatidylinositol-mediated signaling | 25/941 | 192/18862 | 1.09E-05 | 0.000173 | 0.000124 | ANGPT1/DCN/FGFR1/FYN/HGF/HTR2B/NPR3/PDGFC/PDGFRA/PREX2/ROR2/TEK/F2R/FLT1/FN1/KDR/PDGFB/PDGFRB/SELP/TWIST1/UNC5B/CSF1R/FGR/HCST/PTAFR | 25 |
| BP | GO:0001656 | metanephros development | 15/941 | 82/18862 | 1.14E-05 | 0.000181 | 0.00013 | BASP1/FAT4/FGF10/GLI3/GREM1/PDGFRA/PKD2/WWTR1/CD34/FBN1/KIF26B/LAMB2/PDGFB/PDGFRB/SOX17 | 15 |
| BP | GO:0030316 | osteoclast differentiation | 16/941 | 92/18862 | 1.15E-05 | 0.000182 | 0.00013 | LRRC17/MITF/NOTCH2/FBN1/FSTL3/GPR68/CCR1/CSF1/CSF1R/LILRB1/LILRB3/LILRB4/MAFB/OSCAR/TREM2/TYROBP | 16 |
| BP | GO:0009595 | detection of biotic stimulus | 10/941 | 38/18862 | 1.20E-05 | 0.000189 | 0.000135 | C4B/SSC5D/FAP/CLEC7A/LY96/NLRC4/NLRP3/TLR1/TLR2/TREM2 | 10 |
| BP | GO:0043406 | positive regulation of MAP kinase activity | 28/941 | 230/18862 | 1.21E-05 | 0.000189 | 0.000136 | CSPG4/FGF1/FGF10/FGFR1/FZD4/HGF/HTR2B/IGFBP6/ITGA1/LRRK2/MDFIC/PDGFC/ROR2/SASH1/TGFB3/FLT1/MAP3K12/PDGFB/PDGFRB/PEA15/THBS1/ADAM8/C5AR1/CXCR4/FPR1/PIK3R6/S1PR2/TGFB1 | 28 |
| BP | GO:0030100 | regulation of endocytosis | 26/941 | 206/18862 | 1.29E-05 | 0.000201 | 0.000144 | ANGPT1/AXL/CAV1/CCL21/CLIP3/EPHA3/GREM1/ITGB3/LRRK2/PRKD1/STON1/SYT11/C3/CDH13/DLG4/RAB31/SELE/SERPINE1/SFRP4/SGIP1/APOE/CD14/CD300A/LILRB1/SPHK1/TREM2 | 26 |
| BP | GO:0007043 | cell-cell junction assembly | 21/941 | 147/18862 | 1.32E-05 | 0.000205 | 0.000147 | CAV1/CDH2/CNTNAP1/GJA1/GJA5/GJC1/JAM3/PARD6G/SNAI2/TLN1/VCL/CDH11/CDH5/CDH6/DCHS1/ESAM/GJA4/HEG1/PECAM1/RAMP2/TRPV4 | 21 |
| BP | GO:0098581 | detection of external biotic stimulus | 8/941 | 24/18862 | 1.34E-05 | 0.000208 | 0.000149 | C4B/SSC5D/CLEC7A/LY96/NLRC4/TLR1/TLR2/TREM2 | 8 |
| BP | GO:0002698 | negative regulation of immune effector process | 19/941 | 125/18862 | 1.37E-05 | 0.000211 | 0.000152 | A2M/ANGPT1/BCL6/HLX/SERPING1/TGFB3/HTRA1/LOXL3/TNFSF4/CD300A/CD84/FCGR2B/FOXP3/HAVCR2/LILRB1/LILRB4/SLAMF8/TGFB1/VSIG4 | 19 |
| BP | GO:0003180 | aortic valve morphogenesis | 9/941 | 31/18862 | 1.37E-05 | 0.000211 | 0.000152 | SLIT2/SNAI2/ELN/EMILIN1/HEYL/TIE1/TWIST1/NFATC1/TGFB1 | 9 |
| BP | GO:0045907 | positive regulation of vasoconstriction | 9/941 | 31/18862 | 1.37E-05 | 0.000211 | 0.000152 | AVPR1A/CAV1/GJA1/GJA5/F2R/TBXA2R/HRH2/ICAM1/PTAFR | 9 |
| BP | GO:1901888 | regulation of cell junction assembly | 25/941 | 195/18862 | 1.43E-05 | 0.00022 | 0.000158 | CAV1/DLC1/EPHA3/FERMT2/FLRT2/GJA1/GPC6/GREM1/MDGA1/MEF2C/NRP1/PHLDB2/SNAI2/TEK/VCL/COL16A1/KDR/MMP14/NLGN2/NTNG2/PPM1F/SYNDIG1/THBS1/THY1/TLR2 | 25 |
| BP | GO:0002523 | leukocyte migration involved in inflammatory response | 7/941 | 18/18862 | 1.47E-05 | 0.000225 | 0.000162 | JAM3/SELE/ADAM8/ITGB2/NLRP3/S100A8/SLAMF8 | 7 |
| BP | GO:0061045 | negative regulation of wound healing | 14/941 | 74/18862 | 1.49E-05 | 0.000227 | 0.000163 | GJA1/PDGFRA/PHLDB2/PRKG1/SERPING1/CD34/FAP/PDGFB/PLAT/PLAU/SERPINE1/THBD/THBS1/APOE | 14 |
| BP | GO:1903053 | regulation of extracellular matrix organization | 11/941 | 47/18862 | 1.50E-05 | 0.000227 | 0.000163 | DDR2/PHLDB1/PHLDB2/AEBP1/ANTXR1/EFEMP2/EMILIN1/FAP/PDPN/TIE1/TGFB1 | 11 |
| BP | GO:0032680 | regulation of tumor necrosis factor production | 22/941 | 160/18862 | 1.53E-05 | 0.000232 | 0.000166 | ANGPT1/AXL/SYT11/CD34/SIRPA/THBS1/TWIST1/C5AR2/CD14/CD33/CLEC7A/FOXP3/HAVCR2/LILRA2/LILRA5/LILRB1/LY96/PTAFR/TLR1/TLR2/TREM2/TYROBP | 22 |
| BP | GO:1903038 | negative regulation of leukocyte cell-cell adhesion | 20/941 | 138/18862 | 1.69E-05 | 0.000255 | 0.000183 | BCL6/CCL21/GLI3/HLX/LOXL3/LRRC32/TNFSF4/CD300A/CD86/FCGR2B/FOXP3/GPNMB/HAVCR2/LAPTM5/LILRB1/LILRB2/LILRB4/PDCD1LG2/TNFAIP8L2/VSIG4 | 20 |
| BP | GO:0098739 | import across plasma membrane | 22/941 | 161/18862 | 1.69E-05 | 0.000255 | 0.000183 | ABCC9/ATP2B4/CACNA2D1/FYN/ITGB1/KCNJ8/RGS2/RGS4/SLC16A2/SLC8A1/RAMP3/SLC12A4/SLC2A3/SLC6A1/THBS1/TRPV4/FOLR2/KCNJ15/KCNJ5/SLC15A3/SLC1A3/TRPV2 | 22 |
| BP | GO:0048041 | focal adhesion assembly | 15/941 | 85/18862 | 1.79E-05 | 0.000269 | 0.000193 | DLC1/EPHA3/FERMT2/GREM1/NRP1/PHLDB2/TEK/VCL/COL16A1/KDR/MMP14/PPM1F/THBS1/THSD1/THY1 | 15 |
| BP | GO:0098801 | regulation of renal system process | 9/941 | 32/18862 | 1.83E-05 | 0.000273 | 0.000196 | AVPR1A/GJA1/GJA5/CORIN/F2R/GNAI2/NPR1/PDGFB/STC1 | 9 |
| BP | GO:0002762 | negative regulation of myeloid leukocyte differentiation | 11/941 | 48/18862 | 1.86E-05 | 0.000276 | 0.000198 | LRRC17/FBN1/FSTL3/GPR68/INHBA/ZBTB46/C1QC/LILRB1/LILRB3/LILRB4/MAFB | 11 |
| BP | GO:0038084 | vascular endothelial growth factor signaling pathway | 11/941 | 48/18862 | 1.86E-05 | 0.000276 | 0.000198 | ADAMTS3/DCN/NRP1/NRP2/PDGFRA/PRKD1/VEGFC/FLT1/FLT4/KDR/PDGFRB | 11 |
| BP | GO:0051051 | negative regulation of transport | 43/941 | 438/18862 | 1.86E-05 | 0.000276 | 0.000198 | ANGPT1/CAV1/EPHA3/FRMD4A/GEM/GJA1/ITGB3/LRRK2/MAP1B/MDFIC/PKD2/PRKD1/PTGER3/RAB23/REM1/RGS2/RGS4/SYT11/BMP8A/DLG4/DYSF/F2R/GNAI2/HECW2/INHBA/KCNE4/PEA15/PPM1F/SFRP4/SIRPA/STC1/THBS1/TWIST1/APOE/CD300A/CD33/CD84/FCGR2B/ICAM1/LILRB1/LILRB2/MMP9/TLR2 | 43 |
| BP | GO:0032640 | tumor necrosis factor production | 22/941 | 162/18862 | 1.87E-05 | 0.000276 | 0.000198 | ANGPT1/AXL/SYT11/CD34/SIRPA/THBS1/TWIST1/C5AR2/CD14/CD33/CLEC7A/FOXP3/HAVCR2/LILRA2/LILRA5/LILRB1/LY96/PTAFR/TLR1/TLR2/TREM2/TYROBP | 22 |
| BP | GO:0033622 | integrin activation | 8/941 | 25/18862 | 1.88E-05 | 0.000277 | 0.000199 | FERMT2/JAM3/TLN1/COL16A1/FN1/SELP/FERMT3/PLEK | 8 |
| BP | GO:0032944 | regulation of mononuclear cell proliferation | 27/941 | 223/18862 | 1.90E-05 | 0.000278 | 0.0002 | BCL6/FGF10/IL6ST/MEF2C/SOX11/VCAM1/LRRC32/TNFSF4/AIF1/CD300A/CD4/CD86/CSF1/FCGR2B/FOXP3/GPNMB/HAVCR2/HLA-DPB1/LILRB1/LILRB2/LILRB4/LST1/MNDA/PDCD1LG2/TNFSF13B/TYROBP/VSIG4 | 27 |
| BP | GO:0031295 | T cell costimulation | 12/941 | 57/18862 | 1.96E-05 | 0.000287 | 0.000206 | CAV1/CCL21/FYN/EFNB3/LGALS1/TNFSF4/CD86/LILRB2/LILRB4/PDCD1LG2/TNFSF13B/TNFSF14 | 12 |
| BP | GO:0003205 | cardiac chamber development | 21/941 | 151/18862 | 2.00E-05 | 0.000291 | 0.000209 | ADAMTS1/GJA5/MEF2C/NOTCH2/NRP1/NRP2/PARVA/RARB/SFRP2/SLIT2/SMARCD3/SOX11/TEK/ZFPM2/COL11A1/ENG/HEG1/HEYL/PLXND1/WNT2/TGFB1 | 21 |
| BP | GO:0030512 | negative regulation of transforming growth factor beta receptor signaling pathway | 15/941 | 86/18862 | 2.07E-05 | 0.000301 | 0.000216 | ASPN/CAV1/CAV2/LTBP1/TGFB1I1/TGFB3/EMILIN1/FBN1/HTRA1/HTRA3/SPRED3/VASN/CHST11/NRROS/TGFB1 | 15 |
| BP | GO:0043534 | blood vessel endothelial cell migration | 23/941 | 175/18862 | 2.09E-05 | 0.000303 | 0.000217 | AKT3/ANGPT1/ATP2B4/FGFR1/GREM1/MAP3K3/MEF2C/MEOX2/NRP1/PRKD1/RHOJ/SLIT2/VEGFC/CDH5/CLEC14A/KDR/MMRN2/PDGFB/TBXA2R/THBS1/VASH1/APOE/TGFB1 | 23 |
| BP | GO:0048568 | embryonic organ development | 41/941 | 412/18862 | 2.11E-05 | 0.000305 | 0.000219 | EFEMP1/FGF10/GLI1/GLI3/HLX/MEF2C/MFAP5/MMP16/NKX3-2/NOTCH2/PBX3/PDGFC/PDGFRA/PKD2/RARB/ROR2/RSPO3/SOX11/TGFB3/ZFPM2/ALDH1A3/COL11A1/COL13A1/CTHRC1/ENG/FBN1/GLI2/KDR/MFAP2/MMP14/PCDH12/PDGFB/PRRX1/SOX17/TWIST1/VASH1/WNT2/CHST11/MAFB/SOCS3/TGFB1 | 41 |
| BP | GO:0060537 | muscle tissue development | 38/941 | 371/18862 | 2.22E-05 | 0.00032 | 0.00023 | CAV1/CAV2/DCN/GJA1/GJC1/GLI1/GREM1/HLX/IGFBP5/MEF2C/MEIS1/MEOX2/PDGFRA/PKD2/RARB/RGS2/RGS4/S1PR1/SGCD/SLC8A1/SOX11/ZFPM2/ALPK2/COL11A1/EFEMP2/EGR2/ELN/ENG/HEG1/HEYL/LOX/MSC/PDGFRB/TENM4/TWIST1/WNT2/TGFB1/VAMP5 | 38 |
| BP | GO:0045669 | positive regulation of osteoblast differentiation | 13/941 | 67/18862 | 2.26E-05 | 0.000323 | 0.000232 | DDR2/FERMT2/GLI3/HGF/IL6ST/MEF2C/PDLIM7/PRKD1/SFRP2/SOX11/TMEM119/WWTR1/CTHRC1 | 13 |
| BP | GO:0050766 | positive regulation of phagocytosis | 13/941 | 67/18862 | 2.26E-05 | 0.000323 | 0.000232 | C4B/TUB/C3/MFGE8/RAB31/SIRPA/CCL2/CLEC7A/FCER1G/FCGR2B/SIRPB1/SLC11A1/TREM2 | 13 |
| BP | GO:0010720 | positive regulation of cell development | 32/941 | 290/18862 | 2.27E-05 | 0.000324 | 0.000233 | FERMT2/FLNA/GLI3/IL6ST/ISLR2/MAP1B/NRP1/PLXNA4/SERPINF1/SLIT2/SMARCD3/SOX11/VEGFC/CDH5/EGR2/FN1/GPR68/NUMBL/PLXND1/SEMA7A/TENM4/WNT2/CASS4/CLEC7A/CXCR4/PLXNC1/PREX1/S1PR2/TGFB1/TLR2/TRPV2/TYROBP | 32 |
| BP | GO:0031346 | positive regulation of cell projection organization | 36/941 | 344/18862 | 2.29E-05 | 0.000326 | 0.000234 | CCDC88A/CCL21/CDC42EP3/DPYSL3/DZIP1/EPHA3/FEZ1/FLNA/FYN/FZD1/FZD4/HGF/ISLR2/MAP1B/NRP1/PLXNA4/PRKD1/RGS2/RHOQ/ROR2/SCN1B/SERPINF1/SLIT2/TENM3/TGFB3/DLG4/FN1/P3H1/PLXND1/SEMA7A/APOE/FES/P2RX7/PLXNC1/SCARF1/TRPV2 | 36 |
| BP | GO:0043552 | positive regulation of phosphatidylinositol 3-kinase activity | 9/941 | 33/18862 | 2.40E-05 | 0.00034 | 0.000244 | CCL21/PDGFRA/PRKD1/TEK/FLT1/PDGFB/PDGFRB/FGR/TGFB1 | 9 |
| BP | GO:0050869 | negative regulation of B cell activation | 9/941 | 33/18862 | 2.40E-05 | 0.00034 | 0.000244 | BCL6/INHBA/CD300A/FCGR2B/FOXP3/LAPTM5/MNDA/SAMSN1/TYROBP | 9 |
| BP | GO:0001101 | response to acid chemical | 18/941 | 119/18862 | 2.45E-05 | 0.000346 | 0.000249 | ATP2B4/AVPR1A/CDO1/FYN/GLRB/PDGFC/PDGFRA/PKD2/COL16A1/COL18A1/COL1A1/COL1A2/COL4A1/COL5A2/COL6A1/MMP2/CYBB/ICAM1 | 18 |
| BP | GO:0030509 | BMP signaling pathway | 21/941 | 154/18862 | 2.70E-05 | 0.00038 | 0.000273 | FST/GDF6/GREM1/NOTCH2/ROR2/SFRP2/SOX11/TGFB3/BMP8A/CDH5/CHRD/COMP/ENG/FBN1/FSTL1/FSTL3/HTRA1/HTRA3/SFRP4/SULF1/TGFB1 | 21 |
| BP | GO:0031294 | lymphocyte costimulation | 12/941 | 59/18862 | 2.83E-05 | 0.000397 | 0.000285 | CAV1/CCL21/FYN/EFNB3/LGALS1/TNFSF4/CD86/LILRB2/LILRB4/PDCD1LG2/TNFSF13B/TNFSF14 | 12 |
| BP | GO:0048592 | eye morphogenesis | 20/941 | 143/18862 | 2.87E-05 | 0.000402 | 0.000289 | EFEMP1/GLI3/MEIS1/MFAP5/NOTCH2/PTPRM/RARB/SDK1/SOX11/TENM3/ALDH1A3/COL5A1/COL5A2/COL8A1/COL8A2/FBN1/MFAP2/THY1/TWIST1/WNT2 | 20 |
| BP | GO:0019932 | second-messenger-mediated signaling | 33/941 | 307/18862 | 2.90E-05 | 0.000405 | 0.000291 | AQP1/ATP2B4/AVPR1A/HTR2B/ITPR1/LRRK2/NPR2/PRKG1/PTGFR/RGS2/SLC8A1/VCAM1/APLNR/CDH13/KDR/LMCD1/NPR1/SAMD14/SELE/SELP/THBS1/APOE/CCR1/CCR8/CD4/CXCR4/FPR1/LAT2/NFATC1/P2RX7/PLEK/SPHK1/TREM2 | 33 |
| BP | GO:0090090 | negative regulation of canonical Wnt signaling pathway | 23/941 | 179/18862 | 3.01E-05 | 0.000418 | 0.0003 | ANKRD6/BICC1/CAV1/CDH2/FZD4/GLI1/GLI3/GREM1/IGFBP6/LATS2/MCC/PRICKLE1/RBMS3/ROR2/SFRP2/SNAI2/WWTR1/CTHRC1/DACT1/DKK3/SFRP4/SOX17/APOE | 23 |
| BP | GO:0010595 | positive regulation of endothelial cell migration | 19/941 | 132/18862 | 3.01E-05 | 0.000418 | 0.0003 | AKT3/ANGPT1/FGF1/FGFR1/ITGB3/MAP3K3/NRP1/NRP2/PRKD1/RHOJ/SASH1/TEK/VEGFC/FLT4/KDR/PDGFB/SPARC/THBS1/TGFB1 | 19 |
| BP | GO:0010543 | regulation of platelet activation | 9/941 | 34/18862 | 3.12E-05 | 0.000432 | 0.00031 | PDGFRA/PRKG1/PDGFB/PDPN/SELP/THBD/APOE/FCER1G/PLEK | 9 |
| BP | GO:0071229 | cellular response to acid chemical | 13/941 | 69/18862 | 3.13E-05 | 0.000433 | 0.000311 | AVPR1A/FYN/PDGFC/PDGFRA/PKD2/COL16A1/COL1A1/COL1A2/COL4A1/COL5A2/COL6A1/MMP2/CYBB | 13 |
| BP | GO:0048608 | reproductive structure development | 40/941 | 405/18862 | 3.15E-05 | 0.000434 | 0.000311 | ADAMTS1/AXL/BASP1/CYP7B1/DCN/FGF10/FLNA/FST/FZD4/GJA1/GLI1/GLI3/NOTCH2/PDGFRA/PKD2/ROR2/RSPO3/SERPINF1/SFRP2/SLIT2/TNC/ZFPM2/ADAM19/C3/FSTL3/GLI2/HTRA1/INHBA/MMP14/MMP19/PCDH12/PDGFB/PDGFRB/STC1/SULF1/VASH1/WNT2/ICAM1/PSAP/SOCS3 | 40 |
| BP | GO:0046849 | bone remodeling | 15/941 | 89/18862 | 3.16E-05 | 0.000434 | 0.000311 | GJA1/GREM1/ITGB3/MITF/NOTCH2/S1PR1/TGFB3/TMEM119/CTHRC1/LTBP3/ACP5/ADAM8/CSF1R/P2RX7/TGFB1 | 15 |
| BP | GO:0010935 | regulation of macrophage cytokine production | 6/941 | 14/18862 | 3.23E-05 | 0.000442 | 0.000317 | TGFB3/GPRC5B/SEMA7A/LAPTM5/LILRB1/TGFB1 | 6 |
| BP | GO:0002577 | regulation of antigen processing and presentation | 7/941 | 20/18862 | 3.29E-05 | 0.000448 | 0.000322 | CCL21/THBS1/FCGR2B/HLA-DOA/LILRB2/SLC11A1/TREM2 | 7 |
| BP | GO:0098703 | calcium ion import across plasma membrane | 7/941 | 20/18862 | 3.29E-05 | 0.000448 | 0.000322 | ATP2B4/CACNA2D1/FYN/SLC8A1/RAMP3/TRPV4/TRPV2 | 7 |
| BP | GO:0007188 | adenylate cyclase-modulating G protein-coupled receptor signaling pathway | 27/941 | 230/18862 | 3.30E-05 | 0.000448 | 0.000322 | ABCA1/AKAP12/ATP2B4/CALCRL/FLNA/GNG2/GPR161/NPR3/PTGER3/PTGFR/RGS2/S1PR1/S1PR3/EDNRA/GNA12/GNAI2/GPR176/GPR4/PTGIR/PTHLH/RAMP2/RAMP3/TBXA2R/FPR1/MARCO/PSAP/S1PR2 | 27 |
| BP | GO:0010718 | positive regulation of epithelial to mesenchymal transition | 11/941 | 51/18862 | 3.41E-05 | 0.000461 | 0.000331 | FERMT2/TGFB1I1/TGFB3/WWTR1/COL1A1/ENG/LOXL2/PDPN/TWIST1/GLIPR2/TGFB1 | 11 |
| BP | GO:0043277 | apoptotic cell clearance | 11/941 | 51/18862 | 3.41E-05 | 0.000461 | 0.000331 | AXL/C4B/ITGB3/C3/MFGE8/THBS1/CCL2/FCN1/MARCO/TREM2/TYROBP | 11 |
| BP | GO:0031623 | receptor internalization | 17/941 | 111/18862 | 3.47E-05 | 0.000467 | 0.000335 | ANGPT1/CALCRL/CAV1/GREM1/HTR2B/ITGB1/ITGB3/DLG4/RAB31/RAMP2/RAMP3/SELE/SFRP4/FCER1G/ITGB2/LILRB1/LILRB4 | 17 |
| BP | GO:0071675 | regulation of mononuclear cell migration | 17/941 | 111/18862 | 3.47E-05 | 0.000467 | 0.000335 | CCL21/CD99L2/GREM1/MSN/SLIT2/SERPINE1/THBS1/ADAM8/AIF1/C3AR1/C5AR1/CCL2/CCR1/CSF1/CSF1R/PLA2G7/SLAMF8 | 17 |
| BP | GO:0042063 | gliogenesis | 31/941 | 283/18862 | 3.49E-05 | 0.000467 | 0.000336 | CDH2/CNTNAP1/CSPG4/DAAM2/FGF10/GLI3/IL6ST/LAMA2/NDN/ROR2/SOX11/SRGAP2C/TRPC4/TSPAN2/VIM/EGR2/LAMB2/MMP14/MXRA8/TENM4/C1QA/C5AR1/CCL2/CSF1/CSF1R/CXCR4/NRROS/S100A8/TGFB1/TLR2/TREM2 | 31 |
| BP | GO:1901654 | response to ketone | 24/941 | 193/18862 | 3.50E-05 | 0.000468 | 0.000336 | AQP1/AVPR1A/CAV1/CCL21/FIBIN/GNG2/KLF9/MSN/NR3C1/PTGFR/SERPINF1/SLIT2/TGFB3/TNC/IGFBP7/RAMP2/TBXA2R/THBS1/TNFSF4/CYBB/ICAM1/PTAFR/TGFB1/TLR2 | 24 |
| BP | GO:0090596 | sensory organ morphogenesis | 28/941 | 244/18862 | 3.59E-05 | 0.000479 | 0.000344 | EFEMP1/FGF10/GLI3/MEIS1/MFAP5/NKX3-2/NOTCH2/PTPRM/RARB/ROR2/SDK1/SOX11/TENM3/ALDH1A3/COL11A1/COL5A1/COL5A2/COL8A1/COL8A2/CTHRC1/FBN1/GLI2/MFAP2/PRRX1/THY1/TWIST1/WNT2/MAFB | 28 |
| BP | GO:0006801 | superoxide metabolic process | 13/941 | 70/18862 | 3.67E-05 | 0.000488 | 0.00035 | FBLN5/GNAI2/NOX4/SH3PXD2B/CLEC7A/CYBB/ITGAM/ITGB2/NCF2/NRROS/PREX1/TGFB1/TYROBP | 13 |
| BP | GO:0061458 | reproductive system development | 40/941 | 408/18862 | 3.72E-05 | 0.000493 | 0.000354 | ADAMTS1/AXL/BASP1/CYP7B1/DCN/FGF10/FLNA/FST/FZD4/GJA1/GLI1/GLI3/NOTCH2/PDGFRA/PKD2/ROR2/RSPO3/SERPINF1/SFRP2/SLIT2/TNC/ZFPM2/ADAM19/C3/FSTL3/GLI2/HTRA1/INHBA/MMP14/MMP19/PCDH12/PDGFB/PDGFRB/STC1/SULF1/VASH1/WNT2/ICAM1/PSAP/SOCS3 | 40 |
| BP | GO:0071902 | positive regulation of protein serine/threonine kinase activity | 33/941 | 311/18862 | 3.76E-05 | 0.000497 | 0.000357 | ATP2B4/CSPG4/FERMT2/FGF1/FGF10/FGFR1/FZD4/HGF/HTR2B/IGFBP6/ITGA1/LRRK2/MDFIC/PDGFC/PKD2/ROR2/SASH1/TGFB3/FLT1/MAP3K12/PDGFB/PDGFRB/PEA15/THBS1/ADAM8/C5AR1/CSF1R/CXCR4/FAM20A/FPR1/PIK3R6/S1PR2/TGFB1 | 33 |
| BP | GO:0045637 | regulation of myeloid cell differentiation | 29/941 | 258/18862 | 3.85E-05 | 0.000508 | 0.000365 | LRRC17/MEF2C/MEIS1/MITF/NOTCH2/RBFOX2/ROR2/FBN1/FSTL3/GPR68/INHBA/LOX/THBS1/ZBTB46/C1QC/CCR1/CD4/CSF1/CSF3R/FES/LILRB1/LILRB3/LILRB4/MAFB/RAB7B/SPI1/TGFB1/TREM2/TYROBP | 29 |
| BP | GO:0001818 | negative regulation of cytokine production | 37/941 | 367/18862 | 3.99E-05 | 0.000524 | 0.000376 | ANGPT1/AXL/BCL6/C1QTNF3/HGF/NAV3/SSC5D/SYT11/TGFB3/CD34/FN1/INHBA/LRRC32/SIRPA/THBS1/TNFSF4/TWIST1/C5AR2/CD33/CD84/FCGR2B/FOXP3/GPNMB/HAVCR2/LAPTM5/LILRA5/LILRB1/LILRB4/NLRP3/PDCD1LG2/SLC11A1/SRGN/TGFB1/TLR8/TREM2/TYROBP/VSIG4 | 37 |
| BP | GO:0032943 | mononuclear cell proliferation | 31/941 | 285/18862 | 4.00E-05 | 0.000524 | 0.000376 | BCL6/FGF10/FYN/IL6ST/MEF2C/MSN/SOX11/VCAM1/LRRC32/TNFSF4/AIF1/CD300A/CD4/CD86/CSF1/FCGR2B/FOXP3/GPNMB/HAVCR2/HLA-DPB1/LILRB1/LILRB2/LILRB4/LST1/MNDA/PDCD1LG2/SLC11A1/TNFSF13B/TNFSF14/TYROBP/VSIG4 | 31 |
| BP | GO:0042554 | superoxide anion generation | 9/941 | 35/18862 | 4.02E-05 | 0.000525 | 0.000377 | GNAI2/NOX4/CLEC7A/CYBB/ITGAM/ITGB2/NCF2/TGFB1/TYROBP | 9 |
| BP | GO:0002886 | regulation of myeloid leukocyte mediated immunity | 11/941 | 52/18862 | 4.13E-05 | 0.000538 | 0.000386 | C3/CD300A/CD84/FCGR2B/FES/FGR/ITGAM/ITGB2/PRAM1/PTAFR/TYROBP | 11 |
| BP | GO:0030510 | regulation of BMP signaling pathway | 15/941 | 91/18862 | 4.13E-05 | 0.000538 | 0.000386 | FST/GREM1/NOTCH2/SFRP2/SOX11/CDH5/CHRD/ENG/FBN1/FSTL1/FSTL3/HTRA1/HTRA3/SFRP4/SULF1 | 15 |
| BP | GO:0071621 | granulocyte chemotaxis | 18/941 | 124/18862 | 4.31E-05 | 0.000559 | 0.000401 | CCL21/ITGA1/JAM3/SLIT2/THBS4/THBS1/C3AR1/C5AR1/C5AR2/CCL18/CCL2/CSF1/CSF1R/CSF3R/FCER1G/ITGB2/PREX1/S100A8 | 18 |
| BP | GO:1903131 | mononuclear cell differentiation | 40/941 | 411/18862 | 4.38E-05 | 0.000567 | 0.000407 | AXL/BCL6/GLI3/HLX/ITGB1/NOTCH2/PDE1B/RORA/VCAM1/GLI2/GPR68/INHBA/LGALS1/LOXL3/MMP14/TNFSF4/ZBTB46/ADAM8/CD4/CD86/CSF1/CSF1R/CTSL/FCER1G/FCGR2B/FES/FOXP3/HLA-DOA/LILRB1/LILRB2/LILRB4/LYL1/MAFB/NFAM1/NLRP3/PIK3R6/PREX1/SLAMF8/SPI1/TREM2 | 40 |
| BP | GO:0050670 | regulation of lymphocyte proliferation | 26/941 | 221/18862 | 4.42E-05 | 0.00057 | 0.000409 | BCL6/FGF10/IL6ST/MEF2C/SOX11/VCAM1/LRRC32/TNFSF4/AIF1/CD300A/CD4/CD86/FCGR2B/FOXP3/GPNMB/HAVCR2/HLA-DPB1/LILRB1/LILRB2/LILRB4/LST1/MNDA/PDCD1LG2/TNFSF13B/TYROBP/VSIG4 | 26 |
| BP | GO:0036303 | lymph vessel morphogenesis | 7/941 | 21/18862 | 4.72E-05 | 0.000605 | 0.000434 | PTPN14/VEGFC/CLEC14A/FLT4/PDPN/TIE1/VASH1 | 7 |
| BP | GO:1902656 | calcium ion import into cytosol | 7/941 | 21/18862 | 4.72E-05 | 0.000605 | 0.000434 | ATP2B4/CACNA2D1/FYN/SLC8A1/RAMP3/TRPV4/TRPV2 | 7 |
| BP | GO:0001958 | endochondral ossification | 8/941 | 28/18862 | 4.74E-05 | 0.000605 | 0.000434 | MEF2C/MMP16/TEK/TMEM119/ALPL/COL13A1/COL1A1/MMP14 | 8 |
| BP | GO:0036075 | replacement ossification | 8/941 | 28/18862 | 4.74E-05 | 0.000605 | 0.000434 | MEF2C/MMP16/TEK/TMEM119/ALPL/COL13A1/COL1A1/MMP14 | 8 |
| BP | GO:0048546 | digestive tract morphogenesis | 10/941 | 44/18862 | 4.80E-05 | 0.00061 | 0.000438 | FGF10/GLI1/GLI3/HLX/PDGFRA/SFRP2/SOX11/DACT1/GLI2/SOX17 | 10 |
| BP | GO:0048565 | digestive tract development | 18/941 | 125/18862 | 4.80E-05 | 0.00061 | 0.000438 | CLMP/FAT4/FGF10/GLI1/GLI3/HLX/IL6ST/NKX3-2/PDGFC/PDGFRA/RARB/SFRP2/SOX11/TGFB3/DACT1/DCHS1/GLI2/SOX17 | 18 |
| BP | GO:0032651 | regulation of interleukin-1 beta production | 16/941 | 103/18862 | 4.91E-05 | 0.000622 | 0.000446 | S1PR3/F2R/SIRPA/CD33/CLEC7A/LILRA2/LILRA5/LILRB4/MNDA/NLRC4/NLRP3/P2RX7/SPHK1/TLR8/TREM2/TYROBP | 16 |
| BP | GO:0010524 | positive regulation of calcium ion transport into cytosol | 11/941 | 53/18862 | 4.98E-05 | 0.000628 | 0.000451 | CAV1/PKD2/TRPC1/APLNR/F2R/F2RL3/RAMP3/THY1/CD4/P2RX7/P2RY6 | 11 |
| BP | GO:1905517 | macrophage migration | 11/941 | 53/18862 | 4.98E-05 | 0.000628 | 0.000451 | ROR2/EMILIN1/MMP14/THBS1/C3AR1/C5AR1/CCL2/CSF1/CSF1R/SLAMF8/TREM2 | 11 |
| BP | GO:0003176 | aortic valve development | 9/941 | 36/18862 | 5.12E-05 | 0.000639 | 0.000459 | SLIT2/SNAI2/ELN/EMILIN1/HEYL/TIE1/TWIST1/NFATC1/TGFB1 | 9 |
| BP | GO:0010763 | positive regulation of fibroblast migration | 6/941 | 15/18862 | 5.15E-05 | 0.000639 | 0.000459 | AKAP12/DDR2/ITGB1/SLC8A1/THBS1/TGFB1 | 6 |
| BP | GO:0010934 | macrophage cytokine production | 6/941 | 15/18862 | 5.15E-05 | 0.000639 | 0.000459 | TGFB3/GPRC5B/SEMA7A/LAPTM5/LILRB1/TGFB1 | 6 |
| BP | GO:0038065 | collagen-activated signaling pathway | 6/941 | 15/18862 | 5.15E-05 | 0.000639 | 0.000459 | DDR2/COL1A1/COL4A1/COL4A2/ITGA11/OSCAR | 6 |
| BP | GO:0061484 | hematopoietic stem cell homeostasis | 6/941 | 15/18862 | 5.15E-05 | 0.000639 | 0.000459 | ARMCX1/EMCN/GPRASP2/MYCT1/FSTL1/GLIS2 | 6 |
| BP | GO:0070831 | basement membrane assembly | 6/941 | 15/18862 | 5.15E-05 | 0.000639 | 0.000459 | PHLDB1/PHLDB2/LAMB2/NTNG2/PXDN/RAMP2 | 6 |
| BP | GO:0003015 | heart process | 31/941 | 289/18862 | 5.21E-05 | 0.000645 | 0.000463 | ABCC9/ATP2B4/AVPR1A/CACNA2D1/CAV1/FLNA/FYN/GJA1/GJA5/GJC1/ITPR1/KCNJ8/NPR2/RGS2/RGS4/SCN1B/SGCD/SLC8A1/TRPC1/WWTR1/CORIN/EHD3/GNAI2/KCND2/KCNE4/NOX4/NPR1/RAMP3/STC1/CXCR4/KCNJ5 | 31 |
| BP | GO:0019722 | calcium-mediated signaling | 24/941 | 198/18862 | 5.31E-05 | 0.000656 | 0.000471 | ATP2B4/AVPR1A/HTR2B/ITPR1/LRRK2/PTGFR/SLC8A1/VCAM1/CDH13/KDR/LMCD1/SAMD14/SELE/SELP/CCR1/CCR8/CD4/CXCR4/LAT2/NFATC1/P2RX7/PLEK/SPHK1/TREM2 | 24 |
| BP | GO:0007517 | muscle organ development | 33/941 | 317/18862 | 5.49E-05 | 0.000677 | 0.000486 | ADARB1/BASP1/CAV1/CAV2/DCN/EVC/HLX/LAMA2/MEF2C/MEOX2/MRAS/POU6F1/S1PR1/SGCD/SGCE/SOX11/ZFPM2/COL11A1/COL6A3/EFEMP2/EGR2/ELN/ENG/FHL3/HEG1/HEYL/ITGA11/LOX/MSC/TWIST1/WNT2/TGFB1/VAMP5 | 33 |
| BP | GO:0060193 | positive regulation of lipase activity | 13/941 | 73/18862 | 5.81E-05 | 0.000715 | 0.000513 | AVPR1A/FGFR1/HTR2B/PDGFRA/EDNRA/FLT1/PDGFRB/SELE/C5AR1/CD86/P2RY6/PTAFR/RASGRP4 | 13 |
| BP | GO:0014911 | positive regulation of smooth muscle cell migration | 10/941 | 45/18862 | 5.89E-05 | 0.000722 | 0.000518 | ADAMTS1/IGFBP5/NRP1/SSH1/NOX4/PDGFB/PDGFRB/POSTN/AIF1/DOCK4 | 10 |
| BP | GO:0001954 | positive regulation of cell-matrix adhesion | 11/941 | 54/18862 | 5.98E-05 | 0.000731 | 0.000524 | CCL21/FERMT2/NRP1/TEK/CDH13/COL16A1/EFEMP2/KDR/PPM1F/THY1/CSF1 | 11 |
| BP | GO:0045666 | positive regulation of neuron differentiation | 15/941 | 94/18862 | 6.10E-05 | 0.000743 | 0.000534 | BCL6/BEND6/FEZ1/FGFR1/MAP1B/MEF2C/RARB/SOX11/TCF4/TIMP2/TRPC6/ZEB1/GLI2/GPRC5B/HEYL | 15 |
| BP | GO:0051056 | regulation of small GTPase mediated signal transduction | 33/941 | 319/18862 | 6.22E-05 | 0.000756 | 0.000543 | A2M/ABCA1/ARHGAP24/ARHGAP28/ARHGAP31/BCL6/CYTH3/DLC1/FGF10/IQSEC3/NOTCH2/NRP1/RDX/RHOJ/RHOQ/SLIT2/TIMP2/ARHGEF17/CHN1/COL3A1/F2R/F2RL3/GPR4/HEG1/PDGFRB/PLEKHG2/STARD8/SYDE1/APOE/CSF1/CYTH4/PREX1/RASGRP4 | 33 |
| BP | GO:0048251 | elastic fiber assembly | 5/941 | 10/18862 | 6.24E-05 | 0.000758 | 0.000544 | FBLN5/EFEMP2/EMILIN1/LOX/LTBP3 | 5 |
| BP | GO:0090218 | positive regulation of lipid kinase activity | 9/941 | 37/18862 | 6.47E-05 | 0.000784 | 0.000562 | CCL21/PDGFRA/PRKD1/TEK/FLT1/PDGFB/PDGFRB/FGR/TGFB1 | 9 |
| BP | GO:0002718 | regulation of cytokine production involved in immune response | 14/941 | 84/18862 | 6.53E-05 | 0.000788 | 0.000566 | ANGPT1/BCL6/IL1R1/TGFB3/GPRC5B/SEMA7A/TNFSF4/CLEC7A/FOXP3/LAPTM5/LILRB1/LILRB4/NLRP3/TGFB1 | 14 |
| BP | GO:0042692 | muscle cell differentiation | 36/941 | 362/18862 | 6.70E-05 | 0.000807 | 0.000579 | AVPR1A/BOC/CAV2/CDH2/EHD2/FGF10/GREM1/IGFBP5/MEF2C/PDGFRA/PLEKHO1/QKI/RARB/RGS2/RGS4/RORA/SLC8A1/SMARCD3/SYNE1/TMEM119/TMOD2/ADAM12/ALPK2/COMP/DYSF/EFEMP2/ENG/LOX/MMP14/PDGFB/PDGFRB/RAMP2/TMEM204/CD53/NFATC1/TGFB1 | 36 |
| BP | GO:0046718 | viral entry into host cell | 19/941 | 140/18862 | 6.86E-05 | 0.000824 | 0.000591 | AXL/CAV1/CAV2/ITGA5/ITGB1/ITGB3/NRP1/EFNB3/LGALS1/CD4/CD86/CLEC5A/CTSL/CXCR4/FCN1/ICAM1/MRC1/SELPLG/SIGLEC1 | 19 |
| BP | GO:0060840 | artery development | 15/941 | 95/18862 | 6.91E-05 | 0.000828 | 0.000594 | AKT3/GJA5/GLI3/NRP1/PKD2/PRICKLE1/COMP/EFEMP2/ENG/LOX/LOXL1/PDGFRB/PLXND1/PRRX1/APOE | 15 |
| BP | GO:0060402 | calcium ion transport into cytosol | 20/941 | 152/18862 | 6.95E-05 | 0.000831 | 0.000597 | ATP2B4/CACNA2D1/CAV1/CCL21/FYN/HTR2B/ITPR1/PKD2/SLC8A1/TRPC1/APLNR/F2R/F2RL3/RAMP3/THY1/TRPV4/CD4/P2RX7/P2RY6/TRPV2 | 20 |
| BP | GO:0072009 | nephron epithelium development | 16/941 | 106/18862 | 7.01E-05 | 0.000836 | 0.0006 | ADAMTS16/BASP1/FAT4/FGF1/GLI3/GREM1/MEF2C/NOTCH2/PKD2/WWTR1/CD34/DCHS1/HEYL/KIF26B/LAMB2/PECAM1 | 16 |
| BP | GO:0008038 | neuron recognition | 10/941 | 46/18862 | 7.20E-05 | 0.000852 | 0.000612 | EPHA3/NCAM2/NDN/NEXN/PALLD/EFNB3/NTM/PCDH12/ROBO4/CXCR4 | 10 |
| BP | GO:0048146 | positive regulation of fibroblast proliferation | 10/941 | 46/18862 | 7.20E-05 | 0.000852 | 0.000612 | AQP1/DDR2/FGF10/PDGFC/PDGFRA/FN1/PDGFB/PDGFRB/WNT2/SPHK1 | 10 |
| BP | GO:0048483 | autonomic nervous system development | 10/941 | 46/18862 | 7.20E-05 | 0.000852 | 0.000612 | ADARB1/HLX/NRP1/NRP2/PLXNA4/SOX11/VCAM1/EDNRA/EGR2/FN1 | 10 |
| BP | GO:0007015 | actin filament organization | 41/941 | 435/18862 | 7.28E-05 | 0.00086 | 0.000618 | ARHGAP28/CALD1/CCDC88A/CCL21/CDC42EP3/DLC1/DPYSL3/EFS/FERMT2/FLNA/GAS7/MYO5A/NRP1/PHLDB2/RDX/RGS4/S1PR1/SLIT2/SSH1/SYNPO/TGFB3/TMOD2/WIPF1/ARHGEF15/ELN/ESAM/KANK3/NOX4/PLEKHG2/PPM1F/SAMD14/TRPV4/AIF1/CASS4/GMFG/HCK/ICAM1/MYO1F/MYO1G/PLEK/PREX1 | 41 |
| BP | GO:0048661 | positive regulation of smooth muscle cell proliferation | 15/941 | 96/18862 | 7.82E-05 | 0.000921 | 0.000661 | ADAMTS1/CALCRL/GJA1/IGFBP5/S1PR1/CDH13/GNAI2/MMP2/PDGFB/PDGFRB/THBS1/AIF1/MMP9/P2RY6/PTAFR | 15 |
| BP | GO:0032649 | regulation of interferon-gamma production | 16/941 | 107/18862 | 7.87E-05 | 0.000923 | 0.000663 | AXL/IL1R1/INHBA/SIRPA/TNFSF4/CD14/CLEC7A/FOXP3/HAVCR2/HLA-DPB1/LAPTM5/LILRB1/LILRB4/PDCD1LG2/SLC11A1/TLR8 | 16 |
| BP | GO:0044764 | multi-organism cellular process | 6/941 | 16/18862 | 7.89E-05 | 0.000923 | 0.000663 | CAV1/CAV2/CD4/CTSL/CXCR4/SIGLEC1 | 6 |
| BP | GO:1901201 | regulation of extracellular matrix assembly | 6/941 | 16/18862 | 7.89E-05 | 0.000923 | 0.000663 | PHLDB1/PHLDB2/ANTXR1/EMILIN1/TIE1/TGFB1 | 6 |
| BP | GO:0046651 | lymphocyte proliferation | 30/941 | 282/18862 | 7.96E-05 | 0.000928 | 0.000666 | BCL6/FGF10/FYN/IL6ST/MEF2C/MSN/SOX11/VCAM1/LRRC32/TNFSF4/AIF1/CD300A/CD4/CD86/FCGR2B/FOXP3/GPNMB/HAVCR2/HLA-DPB1/LILRB1/LILRB2/LILRB4/LST1/MNDA/PDCD1LG2/SLC11A1/TNFSF13B/TNFSF14/TYROBP/VSIG4 | 30 |
| BP | GO:0014706 | striated muscle tissue development | 35/941 | 351/18862 | 7.97E-05 | 0.000928 | 0.000666 | CAV1/CAV2/DCN/GJA1/GJC1/GLI1/GREM1/HLX/MEF2C/MEIS1/MEOX2/PDGFRA/RARB/RGS2/RGS4/S1PR1/SGCD/SLC8A1/SOX11/ZFPM2/ALPK2/COL11A1/EGR2/ELN/ENG/HEG1/HEYL/LOX/MSC/PDGFRB/TENM4/TWIST1/WNT2/TGFB1/VAMP5 | 35 |
| BP | GO:2000249 | regulation of actin cytoskeleton reorganization | 9/941 | 38/18862 | 8.11E-05 | 0.00094 | 0.000675 | NOTCH2/NRP1/PDGFRA/TEK/ESAM/CSF1R/FES/GMFG/HCK | 9 |
| BP | GO:0046683 | response to organophosphorus | 18/941 | 130/18862 | 8.11E-05 | 0.00094 | 0.000675 | ABCC9/AQP1/CDO1/IGFBP5/PKD2/SLC8A1/SSH1/TEK/COL1A1/MMP19/NOX4/SPARC/STC1/THBD/AQP9/P2RX7/P2RY6/PTAFR | 18 |
| BP | GO:0032755 | positive regulation of interleukin-6 production | 14/941 | 86/18862 | 8.51E-05 | 0.000984 | 0.000706 | F2R/TNFSF4/TWIST1/AIF1/CLEC7A/LILRA2/LILRA5/LILRB2/PTAFR/RAB7B/TLR1/TLR2/TLR8/TYROBP | 14 |
| BP | GO:0032652 | regulation of interleukin-1 production | 17/941 | 119/18862 | 8.55E-05 | 0.000986 | 0.000708 | S1PR3/F2R/SIRPA/CD33/CLEC7A/HAVCR2/LILRA2/LILRA5/LILRB4/MNDA/NLRC4/NLRP3/P2RX7/SPHK1/TLR8/TREM2/TYROBP | 17 |
| BP | GO:0003012 | muscle system process | 42/941 | 453/18862 | 8.65E-05 | 0.000996 | 0.000715 | ACTA2/ANXA6/ATP2B4/CACNA2D1/CALCRL/CALD1/CAV1/FLNA/GJA1/GJA5/GJC1/HTR2B/IGFBP5/IL6ST/ITGA1/KCNJ8/PRKG1/PTGER3/RGS2/RGS4/SCN1B/SLC8A1/SSPN/TLN1/TMOD2/VCL/VIM/COMP/DYSF/EDNRA/EHD3/F2R/KCNE4/LMCD1/STC1/SULF1/TBXA2R/CXCR4/DOCK4/KCNJ5/PTAFR/SPHK1 | 42 |
| BP | GO:0032611 | interleukin-1 beta production | 16/941 | 108/18862 | 8.81E-05 | 0.001011 | 0.000726 | S1PR3/F2R/SIRPA/CD33/CLEC7A/LILRA2/LILRA5/LILRB4/MNDA/NLRC4/NLRP3/P2RX7/SPHK1/TLR8/TREM2/TYROBP | 16 |
| BP | GO:0045807 | positive regulation of endocytosis | 15/941 | 97/18862 | 8.83E-05 | 0.001011 | 0.000726 | ANGPT1/AXL/CCL21/CLIP3/GREM1/LRRK2/C3/RAB31/SELE/SERPINE1/SFRP4/SGIP1/APOE/CD14/TREM2 | 15 |
| BP | GO:0072073 | kidney epithelium development | 18/941 | 131/18862 | 8.97E-05 | 0.001025 | 0.000736 | ADAMTS16/BASP1/FAT4/FGF1/GLI3/GREM1/MEF2C/NOTCH2/PKD2/RARB/SLIT2/WWTR1/CD34/DCHS1/HEYL/KIF26B/LAMB2/PECAM1 | 18 |
| BP | GO:0003416 | endochondral bone growth | 7/941 | 23/18862 | 9.12E-05 | 0.001037 | 0.000745 | ANXA6/BNC2/DDR2/EVC/RARB/COMP/STC1 | 7 |
| BP | GO:1903055 | positive regulation of extracellular matrix organization | 7/941 | 23/18862 | 9.12E-05 | 0.001037 | 0.000745 | DDR2/PHLDB1/PHLDB2/EFEMP2/EMILIN1/PDPN/TGFB1 | 7 |
| BP | GO:0043112 | receptor metabolic process | 21/941 | 168/18862 | 9.82E-05 | 0.001114 | 0.0008 | ANGPT1/CALCRL/CAV1/GREM1/HTR2B/ITGB1/ITGB3/DLG4/EHD3/RAB31/RAMP2/RAMP3/SELE/SFRP4/APOE/FCER1G/ITGB2/LAPTM5/LILRB1/LILRB4/TGFB1 | 21 |
| BP | GO:0043551 | regulation of phosphatidylinositol 3-kinase activity | 11/941 | 57/18862 | 0.0001 | 0.001138 | 0.000817 | CCL21/PDGFRA/PRKD1/TEK/FLT1/PDGFB/PDGFRB/FGR/PIK3R6/SOCS3/TGFB1 | 11 |
| BP | GO:1905521 | regulation of macrophage migration | 9/941 | 39/18862 | 0.000101 | 0.001139 | 0.000817 | EMILIN1/MMP14/THBS1/C3AR1/C5AR1/CSF1/CSF1R/SLAMF8/TREM2 | 9 |
| BP | GO:0010812 | negative regulation of cell-substrate adhesion | 12/941 | 67/18862 | 0.000105 | 0.001183 | 0.000849 | BCL6/DLC1/FBLN1/FZD4/PHLDB2/COL1A1/LGALS1/MMP14/POSTN/SERPINE1/SPOCK1/THBS1 | 12 |
| BP | GO:0003382 | epithelial cell morphogenesis | 8/941 | 31/18862 | 0.000105 | 0.001184 | 0.000849 | CLIC4/PALLD/COL15A1/COL18A1/COL22A1/HEG1/NOTCH4/STC1 | 8 |
| BP | GO:0061028 | establishment of endothelial barrier | 10/941 | 48/18862 | 0.000105 | 0.001184 | 0.000849 | MSN/RDX/S1PR3/VCL/CDH5/ENG/PECAM1/ROBO4/ICAM1/S1PR2 | 10 |
| BP | GO:0110053 | regulation of actin filament organization | 29/941 | 273/18862 | 0.000107 | 0.001195 | 0.000858 | ARHGAP28/CCDC88A/CCL21/CDC42EP3/DLC1/FERMT2/FLNA/NRP1/PHLDB2/RDX/RGS4/S1PR1/SLIT2/SSH1/SYNPO/TGFB3/TMOD2/ARHGEF15/ELN/ESAM/KANK3/NOX4/PLEKHG2/PPM1F/GMFG/HCK/ICAM1/PLEK/PREX1 | 29 |
| BP | GO:0045628 | regulation of T-helper 2 cell differentiation | 5/941 | 11/18862 | 0.00011 | 0.001226 | 0.00088 | BCL6/HLX/TNFSF4/CD86/NLRP3 | 5 |
| BP | GO:0001894 | tissue homeostasis | 28/941 | 260/18862 | 0.00011 | 0.001228 | 0.000882 | AKT3/ANGPT1/GJA1/ITGB1/ITGB3/JAM3/LAMA2/LAMC1/LCA5/LDB2/S1PR1/TMEM119/TUB/VCL/WWTR1/CD34/CDH5/ESAM/F2R/LTBP3/PECAM1/RCN3/ACP5/ADAM8/CSF1/CSF1R/CYTL1/P2RX7 | 28 |
| BP | GO:0001764 | neuron migration | 19/941 | 145/18862 | 0.000111 | 0.00123 | 0.000883 | AXL/FGFR1/FLNA/FLRT2/FYN/MAP1B/MDGA1/MEF2C/NAV1/NDN/NRP1/NRP2/PRKG1/SRGAP2C/COL3A1/NTNG2/SPOCK1/TWIST1/CXCR4 | 19 |
| BP | GO:0097094 | craniofacial suture morphogenesis | 6/941 | 17/18862 | 0.000117 | 0.001296 | 0.00093 | GLI3/MMP16/RAB23/TGFB3/MMP14/TWIST1 | 6 |
| BP | GO:2000351 | regulation of endothelial cell apoptotic process | 11/941 | 58/18862 | 0.000118 | 0.001311 | 0.000941 | ANGPT1/ECSCR/TEK/CD248/CDH5/KDR/RAMP2/SERPINE1/THBS1/CCL2/ICAM1 | 11 |
| BP | GO:0007266 | Rho protein signal transduction | 18/941 | 134/18862 | 0.000121 | 0.001329 | 0.000954 | ABCA1/BCL6/CDC42EP3/DLC1/NRP1/RHOJ/CDH13/COL1A2/COL3A1/F2R/F2RL3/GNA12/GPR4/HEG1/PDGFRB/PDPN/STARD8/APOE | 18 |
| BP | GO:0055123 | digestive system development | 18/941 | 134/18862 | 0.000121 | 0.001329 | 0.000954 | CLMP/FAT4/FGF10/GLI1/GLI3/HLX/IL6ST/NKX3-2/PDGFC/PDGFRA/RARB/SFRP2/SOX11/TGFB3/DACT1/DCHS1/GLI2/SOX17 | 18 |
| BP | GO:0002548 | monocyte chemotaxis | 12/941 | 68/18862 | 0.000122 | 0.001338 | 0.00096 | CCL21/GREM1/SLIT2/FLT1/PDGFB/SERPINE1/AIF1/CCL18/CCL2/CCR1/PLA2G7/SLAMF8 | 12 |
| BP | GO:0003094 | glomerular filtration | 7/941 | 24/18862 | 0.000123 | 0.001346 | 0.000966 | GJA1/GJA5/CD34/F2R/MCAM/PDGFB/SULF1 | 7 |
| BP | GO:0006929 | substrate-dependent cell migration | 7/941 | 24/18862 | 0.000123 | 0.001346 | 0.000966 | CSPG4/NRP1/SLIT2/VEGFC/FN1/ITGA11/ADAM8 | 7 |
| BP | GO:1905523 | positive regulation of macrophage migration | 7/941 | 24/18862 | 0.000123 | 0.001346 | 0.000966 | MMP14/THBS1/C3AR1/C5AR1/CSF1/CSF1R/TREM2 | 7 |
| BP | GO:0071634 | regulation of transforming growth factor beta production | 9/941 | 40/18862 | 0.000124 | 0.001355 | 0.000972 | LTBP1/CD34/FN1/LRRC32/LUM/THBS1/FOXP3/LILRB1/TYROBP | 9 |
| BP | GO:0002703 | regulation of leukocyte mediated immunity | 24/941 | 209/18862 | 0.000125 | 0.001361 | 0.000977 | BCL6/CADM1/IL1R1/C3/TNFSF4/CD300A/CD84/CLEC7A/FCGR2B/FES/FGR/FOXP3/HAVCR2/ICAM1/ITGAM/ITGB2/LILRB1/LILRB4/NLRP3/PIK3R6/PRAM1/PTAFR/TGFB1/TYROBP | 24 |
| BP | GO:0071622 | regulation of granulocyte chemotaxis | 10/941 | 49/18862 | 0.000127 | 0.001374 | 0.000986 | CCL21/JAM3/SLIT2/THBS4/THBS1/C3AR1/C5AR1/C5AR2/CSF1/CSF1R | 10 |
| BP | GO:0031348 | negative regulation of defense response | 26/941 | 236/18862 | 0.000132 | 0.001433 | 0.001028 | A2M/C1QTNF3/CALCRL/HGF/PTGIS/RORA/SERPINF1/SERPING1/SYT11/TEK/CDH5/FNDC4/HTRA1/SIRPA/TNFAIP6/APOE/FCGR2B/FOXP3/HAVCR2/LILRB1/NLRP3/SAMHD1/SLAMF8/SOCS3/TNFAIP8L2/VSIG4 | 26 |
| BP | GO:0007202 | activation of phospholipase C activity | 8/941 | 32/18862 | 0.000134 | 0.001447 | 0.001038 | AVPR1A/HTR2B/EDNRA/SELE/C5AR1/CD86/P2RY6/RASGRP4 | 8 |
| BP | GO:0048048 | embryonic eye morphogenesis | 8/941 | 32/18862 | 0.000134 | 0.001447 | 0.001038 | EFEMP1/MFAP5/RARB/SOX11/ALDH1A3/FBN1/MFAP2/TWIST1 | 8 |
| BP | GO:0010001 | glial cell differentiation | 24/941 | 210/18862 | 0.000135 | 0.00145 | 0.001041 | CDH2/CNTNAP1/DAAM2/FGF10/GLI3/IL6ST/LAMA2/ROR2/SOX11/TRPC4/TSPAN2/VIM/EGR2/LAMB2/MXRA8/TENM4/C1QA/C5AR1/CXCR4/NRROS/S100A8/TGFB1/TLR2/TREM2 | 24 |
| BP | GO:0071900 | regulation of protein serine/threonine kinase activity | 44/941 | 492/18862 | 0.000135 | 0.001453 | 0.001043 | ATP2B4/CAV1/CSPG4/FERMT2/FGF1/FGF10/FGFR1/FZD4/HGF/HTR2B/IGFBP6/ITGA1/LATS2/LRRK2/MDFIC/PDGFC/PKD2/RGS2/RGS4/ROR2/SASH1/SFRP2/SLC8A1/TGFB3/FLT1/HEG1/MAP3K12/PDGFB/PDGFRB/PEA15/SPRED3/THBS1/THY1/ADAM8/APOE/C5AR1/CD300A/CSF1R/CXCR4/FAM20A/FPR1/PIK3R6/S1PR2/TGFB1 | 44 |
| BP | GO:0032609 | interferon-gamma production | 16/941 | 112/18862 | 0.000137 | 0.001464 | 0.001051 | AXL/IL1R1/INHBA/SIRPA/TNFSF4/CD14/CLEC7A/FOXP3/HAVCR2/HLA-DPB1/LAPTM5/LILRB1/LILRB4/PDCD1LG2/SLC11A1/TLR8 | 16 |
| BP | GO:0048562 | embryonic organ morphogenesis | 29/941 | 277/18862 | 0.000138 | 0.001471 | 0.001056 | EFEMP1/FGF10/GLI1/GLI3/HLX/MEF2C/MFAP5/MMP16/NKX3-2/NOTCH2/PDGFRA/PKD2/RARB/ROR2/SOX11/TGFB3/ALDH1A3/COL11A1/CTHRC1/ENG/FBN1/GLI2/MFAP2/MMP14/PRRX1/SOX17/TWIST1/CHST11/MAFB | 29 |
| BP | GO:1903035 | negative regulation of response to wounding | 14/941 | 90/18862 | 0.000141 | 0.001496 | 0.001073 | GJA1/PDGFRA/PHLDB2/PRKG1/SERPING1/CD34/FAP/PDGFB/PLAT/PLAU/SERPINE1/THBD/THBS1/APOE | 14 |
| BP | GO:1903557 | positive regulation of tumor necrosis factor superfamily cytokine production | 14/941 | 90/18862 | 0.000141 | 0.001496 | 0.001073 | THBS1/TWIST1/ADAM8/CD14/CD86/CLEC7A/HAVCR2/LILRA2/LILRA5/LY96/PTAFR/TLR1/TLR2/TYROBP | 14 |
| BP | GO:0030099 | myeloid cell differentiation | 39/941 | 419/18862 | 0.000141 | 0.001496 | 0.001073 | BCL6/LRRC17/MEF2C/MEIS1/MITF/NOTCH2/PDE1B/RBFOX2/ROR2/TSPAN2/FBN1/FSTL3/GPR68/INHBA/LOX/THBS1/ZBTB46/C1QC/CCR1/CD4/CSF1/CSF1R/CSF3R/FES/LILRB1/LILRB3/LILRB4/MAFB/MMP9/NRROS/OSCAR/RAB7B/RASGRP4/SPI1/TGFB1/TLR2/TREM2/TYROBP/ZNF385A | 39 |
| BP | GO:0030850 | prostate gland development | 9/941 | 41/18862 | 0.000152 | 0.00161 | 0.001155 | CYP7B1/FGF10/GLI1/GLI3/SERPINF1/TNC/GLI2/SULF1/PSAP | 9 |
| BP | GO:0032689 | negative regulation of interferon-gamma production | 9/941 | 41/18862 | 0.000152 | 0.00161 | 0.001155 | AXL/INHBA/TNFSF4/FOXP3/HAVCR2/LAPTM5/LILRB1/LILRB4/PDCD1LG2 | 9 |
| BP | GO:0032231 | regulation of actin filament bundle assembly | 15/941 | 102/18862 | 0.000158 | 0.001664 | 0.001194 | ARHGAP28/CCDC88A/DLC1/FERMT2/FLNA/NRP1/PHLDB2/RDX/S1PR1/SYNPO/TGFB3/ARHGEF15/NOX4/PPM1F/PLEK | 15 |
| BP | GO:0061337 | cardiac conduction | 19/941 | 149/18862 | 0.000159 | 0.001675 | 0.001202 | ABCC9/ATP2B4/CACNA2D1/CAV1/FLNA/GJA1/GJA5/GJC1/ITPR1/NPR2/SCN1B/SLC8A1/TRPC1/CORIN/EHD3/KCND2/KCNE4/NPR1/KCNJ5 | 19 |
| BP | GO:0034113 | heterotypic cell-cell adhesion | 11/941 | 60/18862 | 0.000163 | 0.001709 | 0.001227 | ITGA5/ITGB1/ITGB3/JAM3/PARVA/VCAM1/SIRPA/THY1/ITGAX/ITGB2/LILRB2 | 11 |
| BP | GO:0042730 | fibrinolysis | 7/941 | 25/18862 | 0.000164 | 0.001709 | 0.001227 | SERPING1/FAP/PLAT/PLAU/SERPINE1/THBD/THBS1 | 7 |
| BP | GO:0051894 | positive regulation of focal adhesion assembly | 7/941 | 25/18862 | 0.000164 | 0.001709 | 0.001227 | FERMT2/NRP1/TEK/COL16A1/KDR/PPM1F/THY1 | 7 |
| BP | GO:0097205 | renal filtration | 7/941 | 25/18862 | 0.000164 | 0.001709 | 0.001227 | GJA1/GJA5/CD34/F2R/MCAM/PDGFB/SULF1 | 7 |
| BP | GO:0032930 | positive regulation of superoxide anion generation | 6/941 | 18/18862 | 0.000168 | 0.001747 | 0.001254 | GNAI2/CLEC7A/ITGAM/ITGB2/TGFB1/TYROBP | 6 |
| BP | GO:0030947 | regulation of vascular endothelial growth factor receptor signaling pathway | 8/941 | 33/18862 | 0.00017 | 0.001761 | 0.001264 | FGF10/FZD4/ITGA5/ITGB3/VEGFC/EMILIN1/MMRN2/TMEM204 | 8 |
| BP | GO:0030048 | actin filament-based movement | 19/941 | 150/18862 | 0.000174 | 0.001803 | 0.001294 | CACNA2D1/CAV1/FLNA/GJA1/GJA5/GJC1/KCNJ8/MYO5A/PARVA/SCN1B/VIM/WIPF1/KCNE4/MYH10/PDPN/STC1/KCNJ5/MYO1F/MYO1G | 19 |
| BP | GO:0032612 | interleukin-1 production | 17/941 | 126/18862 | 0.000175 | 0.001807 | 0.001297 | S1PR3/F2R/SIRPA/CD33/CLEC7A/HAVCR2/LILRA2/LILRA5/LILRB4/MNDA/NLRC4/NLRP3/P2RX7/SPHK1/TLR8/TREM2/TYROBP | 17 |
| BP | GO:0030593 | neutrophil chemotaxis | 15/941 | 103/18862 | 0.000176 | 0.001819 | 0.001305 | CCL21/ITGA1/JAM3/SLIT2/THBS4/C3AR1/C5AR1/C5AR2/CCL18/CCL2/CSF3R/FCER1G/ITGB2/PREX1/S100A8 | 15 |
| BP | GO:0072593 | reactive oxygen species metabolic process | 29/941 | 281/18862 | 0.000177 | 0.001819 | 0.001305 | ATP2B4/CAV1/CYP1B1/FBLN5/FYN/LRRK2/PKD2/PTGIS/RORA/CD34/GNAI2/NOX4/PDGFB/PDGFRB/PXDN/SH3PXD2B/SIRPA/THBS1/CLEC7A/CYBB/ICAM1/ITGAM/ITGB2/NCF2/NRROS/PREX1/TGFB1/TLR2/TYROBP | 29 |
| BP | GO:1904645 | response to amyloid-beta | 10/941 | 51/18862 | 0.00018 | 0.00185 | 0.001327 | CACNA2D1/FYN/GJA1/VCAM1/MMP2/RAMP3/FCGR2B/ICAM1/MMP9/TREM2 | 10 |
| BP | GO:2001204 | regulation of osteoclast development | 5/941 | 12/18862 | 0.00018 | 0.001851 | 0.001329 | NOTCH2/FBN1/GPR68/LILRB1/TYROBP | 5 |
| BP | GO:0007265 | Ras protein signal transduction | 33/941 | 338/18862 | 0.000187 | 0.001918 | 0.001376 | ABCA1/ARHGAP24/BCL6/CDC42EP3/CYTH3/DLC1/FGF10/IQSEC3/MRAS/NOTCH2/NRP1/PRKD1/RDX/RERG/RHOJ/TIMP2/CDH13/COL1A2/COL3A1/F2R/F2RL3/GNA12/GPR4/HEG1/PDGFRB/PDPN/STARD8/AIF1/APOE/CSF1/CYTH4/DOK2/RASGRP4 | 33 |
| BP | GO:0071230 | cellular response to amino acid stimulus | 11/941 | 61/18862 | 0.00019 | 0.001938 | 0.001391 | FYN/PDGFC/PDGFRA/COL16A1/COL1A1/COL1A2/COL4A1/COL5A2/COL6A1/MMP2/CYBB | 11 |
| BP | GO:0002367 | cytokine production involved in immune response | 14/941 | 93/18862 | 0.000201 | 0.002047 | 0.001469 | ANGPT1/BCL6/IL1R1/TGFB3/GPRC5B/SEMA7A/TNFSF4/CLEC7A/FOXP3/LAPTM5/LILRB1/LILRB4/NLRP3/TGFB1 | 14 |
| BP | GO:0051591 | response to cAMP | 14/941 | 93/18862 | 0.000201 | 0.002047 | 0.001469 | AQP1/CDO1/IGFBP5/PKD2/SLC8A1/TEK/COL1A1/MMP19/NOX4/SPARC/STC1/THBD/AQP9/PTAFR | 14 |
| BP | GO:0046578 | regulation of Ras protein signal transduction | 22/941 | 190/18862 | 0.000209 | 0.002124 | 0.001524 | ABCA1/ARHGAP24/BCL6/CYTH3/DLC1/FGF10/IQSEC3/NOTCH2/NRP1/RDX/TIMP2/COL3A1/F2R/F2RL3/GPR4/HEG1/PDGFRB/STARD8/APOE/CSF1/CYTH4/RASGRP4 | 22 |
| BP | GO:0035637 | multicellular organismal signaling | 23/941 | 203/18862 | 0.00021 | 0.002124 | 0.001524 | ABCC9/ATP2B4/AVPR1A/CACNA2D1/CAV1/CNTNAP1/FLNA/GJA1/GJA5/GJC1/ITPR1/JAM3/NPR2/S1PR1/SCN1B/SLC8A1/TRPC1/CORIN/EHD3/KCND2/KCNE4/NPR1/KCNJ5 | 23 |
| BP | GO:0001569 | branching involved in blood vessel morphogenesis | 8/941 | 34/18862 | 0.000212 | 0.002144 | 0.001539 | NRP1/SFRP2/COL4A1/EDNRA/ENG/KDR/NOTCH4/PLXND1 | 8 |
| BP | GO:0002719 | negative regulation of cytokine production involved in immune response | 7/941 | 26/18862 | 0.000215 | 0.002152 | 0.001544 | ANGPT1/BCL6/TGFB3/FOXP3/LILRB1/LILRB4/TGFB1 | 7 |
| BP | GO:0060351 | cartilage development involved in endochondral bone morphogenesis | 7/941 | 26/18862 | 0.000215 | 0.002152 | 0.001544 | ANXA6/RARB/COL1A1/COMP/SERPINH1/STC1/TRPV4 | 7 |
| BP | GO:0061082 | myeloid leukocyte cytokine production | 7/941 | 26/18862 | 0.000215 | 0.002152 | 0.001544 | BCL6/TGFB3/GPRC5B/SEMA7A/LAPTM5/LILRB1/TGFB1 | 7 |
| BP | GO:0098868 | bone growth | 7/941 | 26/18862 | 0.000215 | 0.002152 | 0.001544 | ANXA6/BNC2/DDR2/EVC/RARB/COMP/STC1 | 7 |
| BP | GO:0006027 | glycosaminoglycan catabolic process | 11/941 | 62/18862 | 0.00022 | 0.002201 | 0.00158 | CSPG4/DCN/GPC6/HSPG2/OMD/PRELP/SDC2/BGN/LUM/VCAN/TGFB1 | 11 |
| BP | GO:0070527 | platelet aggregation | 11/941 | 62/18862 | 0.00022 | 0.002201 | 0.00158 | FLNA/ITGB3/PDGFRA/PRKG1/TLN1/VCL/COMP/FN1/PDPN/FERMT3/PLEK | 11 |
| BP | GO:0043300 | regulation of leukocyte degranulation | 9/941 | 43/18862 | 0.000224 | 0.002234 | 0.001603 | CD300A/CD84/FCGR2B/FES/FGR/ITGAM/ITGB2/PRAM1/PTAFR | 9 |
| BP | GO:0070509 | calcium ion import | 13/941 | 83/18862 | 0.000225 | 0.002237 | 0.001605 | ATP2B4/CACNA2D1/FYN/PKD2/SLC8A1/TRPC4/PDGFB/PDGFRB/RAMP3/STC1/TRPV4/CCL2/TRPV2 | 13 |
| BP | GO:0044409 | entry into host | 19/941 | 153/18862 | 0.000226 | 0.002237 | 0.001605 | AXL/CAV1/CAV2/ITGA5/ITGB1/ITGB3/NRP1/EFNB3/LGALS1/CD4/CD86/CLEC5A/CTSL/CXCR4/FCN1/ICAM1/MRC1/SELPLG/SIGLEC1 | 19 |
| BP | GO:0061097 | regulation of protein tyrosine kinase activity | 14/941 | 94/18862 | 0.000226 | 0.002237 | 0.001605 | CAV1/FYN/GREM1/SH3BP5/DLG4/GPRC5B/NOX4/PDGFB/THY1/CASS4/CSF1R/FCGR1A/LILRA5/LILRB4 | 14 |
| BP | GO:0051017 | actin filament bundle assembly | 19/941 | 154/18862 | 0.000246 | 0.002428 | 0.001742 | ARHGAP28/CALD1/CCDC88A/DLC1/DPYSL3/FERMT2/FLNA/NRP1/PHLDB2/RDX/S1PR1/SYNPO/TGFB3/ARHGEF15/ELN/NOX4/PPM1F/AIF1/PLEK | 19 |
| BP | GO:0043491 | protein kinase B signaling | 28/941 | 273/18862 | 0.000251 | 0.002478 | 0.001779 | ANGPT1/AXL/CCL21/FERMT2/FGF1/FGF10/FGF7/FGFR1/FYN/HGF/IGFBP5/ITGB1/PDGFRA/TEK/TSPYL5/ENG/KDR/LOX/PDGFB/PDGFRB/RAMP3/RCN3/THBS1/ADAM8/CASS4/CCL2/CD86/TGFB1 | 28 |
| BP | GO:0032602 | chemokine production | 14/941 | 95/18862 | 0.000253 | 0.00249 | 0.001787 | C1QTNF3/SNAI2/POSTN/SIRPA/TNFSF4/TWIST1/AIF1/CLEC7A/CSF1R/HAVCR2/LILRB4/S100A8/TLR2/TREM2 | 14 |
| BP | GO:0014031 | mesenchymal cell development | 13/941 | 84/18862 | 0.000254 | 0.002498 | 0.001793 | ANXA6/CDH2/HTR2B/NRP1/NRP2/SNAI2/SOX11/EDNRA/FN1/HEYL/SEMA6B/SEMA7A/TWIST1 | 13 |
| BP | GO:0031345 | negative regulation of cell projection organization | 21/941 | 180/18862 | 0.00026 | 0.002552 | 0.001832 | ARHGAP24/CCL21/CRMP1/DENND5A/DPYSL3/FLNA/FYN/LRRK2/NRP1/SLIT2/SRGAP2C/TRPC6/VIM/EFNB3/LGALS1/SEMA6B/SEMA7A/SPOCK1/THY1/TRPV4/APOE | 21 |
| BP | GO:0044272 | sulfur compound biosynthetic process | 22/941 | 193/18862 | 0.000262 | 0.002561 | 0.001838 | ANGPT1/CDO1/CHST15/CHST3/CSGALNACT2/CSPG4/DCN/DSEL/ELOVL4/OMD/PRELP/BGN/CHST1/CHST14/CHSY3/DSE/GGT5/LUM/VCAN/CHST11/CHST2/ST3GAL6 | 22 |
| BP | GO:0097305 | response to alcohol | 25/941 | 233/18862 | 0.000267 | 0.002604 | 0.001869 | AVPR1A/CCL21/CDO1/FYN/GNG2/KLF9/PTGFR/RGS2/RGS4/SLIT2/TNC/VCAM1/IGFBP7/INHBA/SPARC/TBXA2R/TNFSF4/CD14/CYBB/ICAM1/NLRP3/RGS19/S100A8/SPI1/TGFB1 | 25 |
| BP | GO:0002526 | acute inflammatory response | 15/941 | 107/18862 | 0.000271 | 0.002638 | 0.001893 | F8/IL6ST/OSMR/PTGER3/VCAM1/C3/FN1/TNFSF4/ADAM8/CD163/FCGR2B/ICAM1/NLRP3/S100A8/TREM1 | 15 |
| BP | GO:0045671 | negative regulation of osteoclast differentiation | 7/941 | 27/18862 | 0.000277 | 0.002692 | 0.001932 | LRRC17/FBN1/FSTL3/LILRB1/LILRB3/LILRB4/MAFB | 7 |
| BP | GO:0090025 | regulation of monocyte chemotaxis | 7/941 | 27/18862 | 0.000277 | 0.002692 | 0.001932 | GREM1/SLIT2/SERPINE1/AIF1/CCR1/PLA2G7/SLAMF8 | 7 |
| BP | GO:0002430 | complement receptor mediated signaling pathway | 5/941 | 13/18862 | 0.000281 | 0.002714 | 0.001948 | C3AR1/C5AR1/C5AR2/FPR1/FPR3 | 5 |
| BP | GO:0032905 | transforming growth factor beta1 production | 5/941 | 13/18862 | 0.000281 | 0.002714 | 0.001948 | LUM/THBS1/FOXP3/NRROS/TYROBP | 5 |
| BP | GO:1902285 | semaphorin-plexin signaling pathway involved in neuron projection guidance | 5/941 | 13/18862 | 0.000281 | 0.002714 | 0.001948 | NRP1/NRP2/PLXNA4/PLXND1/PLXNC1 | 5 |
| BP | GO:0034109 | homotypic cell-cell adhesion | 13/941 | 85/18862 | 0.000287 | 0.002761 | 0.001982 | FLNA/ITGB3/PDGFRA/PRKG1/RDX/TLN1/VCL/COMP/FN1/LGALS1/PDPN/FERMT3/PLEK | 13 |
| BP | GO:0006968 | cellular defense response | 10/941 | 54/18862 | 0.000294 | 0.00282 | 0.002024 | ITGB1/RAB23/C5AR1/CD300C/CLEC5A/LILRB2/LY96/MNDA/NCF2/TYROBP | 10 |
| BP | GO:0086002 | cardiac muscle cell action potential involved in contraction | 10/941 | 54/18862 | 0.000294 | 0.00282 | 0.002024 | CACNA2D1/CAV1/FLNA/GJA1/GJA5/GJC1/KCNJ8/SCN1B/KCNE4/KCNJ5 | 10 |
| BP | GO:0072577 | endothelial cell apoptotic process | 11/941 | 64/18862 | 0.000294 | 0.00282 | 0.002024 | ANGPT1/ECSCR/TEK/CD248/CDH5/KDR/RAMP2/SERPINE1/THBS1/CCL2/ICAM1 | 11 |
| BP | GO:0014074 | response to purine-containing compound | 18/941 | 144/18862 | 0.000301 | 0.002876 | 0.002064 | ABCC9/AQP1/CDO1/IGFBP5/PKD2/SLC8A1/SSH1/TEK/COL1A1/MMP19/NOX4/SPARC/STC1/THBD/AQP9/P2RX7/P2RY6/PTAFR | 18 |
| BP | GO:1903522 | regulation of blood circulation | 29/941 | 290/18862 | 0.000301 | 0.002876 | 0.002064 | ABCC9/ATP2B4/AVPR1A/CACNA2D1/CAV1/FLNA/GJA1/GJA5/GJC1/ITPR1/NPR2/RGS2/RGS4/SCN1B/SLC8A1/TRPC1/CORIN/EHD3/F2R/KCND2/KCNE4/NPR1/STC1/TBXA2R/DOCK4/HRH2/ICAM1/KCNJ5/PTAFR | 29 |
| BP | GO:0010596 | negative regulation of endothelial cell migration | 14/941 | 97/18862 | 0.000316 | 0.002998 | 0.002152 | ATP2B4/DCN/MEF2C/MEOX2/PTPRM/SERPINF1/SLIT2/MMRN2/STC1/TBXA2R/THBS1/VASH1/APOE/TGFB1 | 14 |
| BP | GO:0060191 | regulation of lipase activity | 14/941 | 97/18862 | 0.000316 | 0.002998 | 0.002152 | AVPR1A/FGFR1/HTR2B/PDGFRA/RGS2/EDNRA/FLT1/PDGFRB/SELE/C5AR1/CD86/P2RY6/PTAFR/RASGRP4 | 14 |
| BP | GO:0001937 | negative regulation of endothelial cell proliferation | 12/941 | 75/18862 | 0.000317 | 0.003007 | 0.002158 | CAV1/CAV2/GJA1/MEF2C/PTPRM/FLT1/SPARC/SULF1/THBS1/VASH1/APOE/CCL2 | 12 |
| BP | GO:0032928 | regulation of superoxide anion generation | 6/941 | 20/18862 | 0.000322 | 0.003038 | 0.002181 | GNAI2/CLEC7A/ITGAM/ITGB2/TGFB1/TYROBP | 6 |
| BP | GO:0090280 | positive regulation of calcium ion import | 6/941 | 20/18862 | 0.000322 | 0.003038 | 0.002181 | PDGFB/PDGFRB/RAMP3/STC1/CCL2/TRPV2 | 6 |
| BP | GO:0034103 | regulation of tissue remodeling | 13/941 | 86/18862 | 0.000323 | 0.003042 | 0.002183 | DDR2/GREM1/ITGB3/S1PR1/THBS4/TMEM119/FLT4/LTBP3/ADAM8/CSF1R/GPNMB/P2RX7/TGFB1 | 13 |
| BP | GO:0060041 | retina development in camera-type eye | 18/941 | 145/18862 | 0.000328 | 0.003087 | 0.002216 | ATP2B4/CLIC4/CYP1B1/FZD4/NRP1/PDGFRA/PRPH2/PTPRM/RHOJ/SDK1/SERPINF1/SMARCD3/TUB/ARHGEF15/COL4A1/LAMB2/PDGFRB/THY1 | 18 |
| BP | GO:0061572 | actin filament bundle organization | 19/941 | 158/18862 | 0.000342 | 0.003211 | 0.002304 | ARHGAP28/CALD1/CCDC88A/DLC1/DPYSL3/FERMT2/FLNA/NRP1/PHLDB2/RDX/S1PR1/SYNPO/TGFB3/ARHGEF15/ELN/NOX4/PPM1F/AIF1/PLEK | 19 |
| BP | GO:0032703 | negative regulation of interleukin-2 production | 7/941 | 28/18862 | 0.000354 | 0.003318 | 0.002382 | NAV3/CD34/FOXP3/HAVCR2/LAPTM5/LILRB4/VSIG4 | 7 |
| BP | GO:0090288 | negative regulation of cellular response to growth factor stimulus | 15/941 | 110/18862 | 0.000367 | 0.003435 | 0.002465 | ATP2B4/DCN/GREM1/SFRP2/SLIT2/ADAMTS12/CHRD/EMILIN1/FBN1/FSTL3/HTRA1/HTRA3/MMRN2/SULF1/THBS1 | 15 |
| BP | GO:0097553 | calcium ion transmembrane import into cytosol | 17/941 | 134/18862 | 0.000368 | 0.003435 | 0.002465 | ATP2B4/CACNA2D1/CCL21/FYN/HTR2B/ITPR1/PKD2/SLC8A1/TRPC1/APLNR/F2R/F2RL3/RAMP3/THY1/TRPV4/P2RY6/TRPV2 | 17 |
| BP | GO:0061515 | myeloid cell development | 11/941 | 66/18862 | 0.000388 | 0.003621 | 0.002599 | BCL6/MEIS1/NOTCH2/TSPAN2/FBN1/GPR68/LILRB1/NRROS/TLR2/TYROBP/ZNF385A | 11 |
| BP | GO:0050728 | negative regulation of inflammatory response | 19/941 | 160/18862 | 0.000401 | 0.003732 | 0.002679 | C1QTNF3/CALCRL/HGF/PTGIS/RORA/SERPINF1/SYT11/TEK/CDH5/FNDC4/SIRPA/TNFAIP6/APOE/FCGR2B/FOXP3/NLRP3/SLAMF8/SOCS3/TNFAIP8L2 | 19 |
| BP | GO:0060993 | kidney morphogenesis | 13/941 | 88/18862 | 0.000406 | 0.003766 | 0.002703 | ADAMTS16/BASP1/FAT4/FGF1/FGF10/GLI3/GREM1/LRRK2/PKD2/WWTR1/DCHS1/KIF26B/PDGFRB | 13 |
| BP | GO:0060401 | cytosolic calcium ion transport | 20/941 | 173/18862 | 0.000407 | 0.003766 | 0.002703 | ATP2B4/CACNA2D1/CAV1/CCL21/FYN/HTR2B/ITPR1/PKD2/SLC8A1/TRPC1/APLNR/F2R/F2RL3/RAMP3/THY1/TRPV4/CD4/P2RX7/P2RY6/TRPV2 | 20 |
| BP | GO:0046209 | nitric oxide metabolic process | 12/941 | 77/18862 | 0.000407 | 0.003766 | 0.002703 | ATP2B4/CAV1/CYP1B1/PKD2/PTGIS/RORA/CD34/SIRPA/CLEC7A/ICAM1/ITGB2/TLR2 | 12 |
| BP | GO:0046777 | protein autophosphorylation | 24/941 | 226/18862 | 0.000408 | 0.003767 | 0.002704 | CAV1/DDR2/FGFR1/GREM1/LRRK2/MAP3K3/PDGFC/PDGFRA/PRKD1/TEK/VEGFC/ENG/FLT1/FLT4/KDR/MAP3K12/MEX3B/PDGFB/PDGFRB/CSF1R/FES/FGR/GPNMB/HCK | 24 |
| BP | GO:0018158 | protein oxidation | 5/941 | 14/18862 | 0.00042 | 0.003852 | 0.002765 | LOXL4/LOX/LOXL1/LOXL2/LOXL3 | 5 |
| BP | GO:0045217 | cell-cell junction maintenance | 5/941 | 14/18862 | 0.00042 | 0.003852 | 0.002765 | FERMT2/F2R/KIFC3/NLGN2/CSF1R | 5 |
| BP | GO:0086103 | G protein-coupled receptor signaling pathway involved in heart process | 5/941 | 14/18862 | 0.00042 | 0.003852 | 0.002765 | ATP2B4/CAV1/RGS2/GNAI2/RAMP3 | 5 |
| BP | GO:0050918 | positive chemotaxis | 11/941 | 67/18862 | 0.000444 | 0.004063 | 0.002916 | ANGPT1/FGF10/FGF7/HGF/NRP1/S1PR1/VEGFC/CDH13/KDR/PDGFB/GPNMB | 11 |
| BP | GO:1904062 | regulation of cation transmembrane transport | 32/941 | 340/18862 | 0.000446 | 0.004063 | 0.002916 | ATP2B4/CACNA2D1/CAV1/FLNA/FYN/GEM/ITGB1/MEF2C/PKD2/REM1/RGS2/RGS4/SCN1B/SLC8A1/STOM/TRPC1/APLNR/CNIH3/DLG4/EHD3/F2R/F2RL3/HECW2/KCNE4/NLGN2/RAMP3/THY1/TWIST1/CCL2/MMP9/P2RY6/TREM2 | 32 |
| BP | GO:0002063 | chondrocyte development | 7/941 | 29/18862 | 0.000447 | 0.004063 | 0.002916 | SFRP2/COL11A1/COMP/PTHLH/SERPINH1/SULF1/CHST11 | 7 |
| BP | GO:0060055 | angiogenesis involved in wound healing | 7/941 | 29/18862 | 0.000447 | 0.004063 | 0.002916 | ITGB3/CD34/GPR4/KDR/MCAM/SERPINE1/CXCR4 | 7 |
| BP | GO:1902624 | positive regulation of neutrophil migration | 7/941 | 29/18862 | 0.000447 | 0.004063 | 0.002916 | CCL21/CD99L2/IL1R1/THBS4/ADAM8/C3AR1/C5AR1 | 7 |
| BP | GO:0010712 | regulation of collagen metabolic process | 9/941 | 47/18862 | 0.000453 | 0.004113 | 0.002952 | ITGB1/TGFB3/VIM/EMILIN1/ENG/F2R/FAP/PDGFRB/TGFB1 | 9 |
| BP | GO:2001057 | reactive nitrogen species metabolic process | 12/941 | 78/18862 | 0.00046 | 0.004166 | 0.00299 | ATP2B4/CAV1/CYP1B1/PKD2/PTGIS/RORA/CD34/SIRPA/CLEC7A/ICAM1/ITGB2/TLR2 | 12 |
| BP | GO:0048008 | platelet-derived growth factor receptor signaling pathway | 10/941 | 57/18862 | 0.000464 | 0.004197 | 0.003012 | CSPG4/NRP1/PDGFC/PDGFRA/PDGFRL/LOX/PDGFB/PDGFRB/PLAT/PTGIR | 10 |
| BP | GO:0097242 | amyloid-beta clearance | 8/941 | 38/18862 | 0.000479 | 0.004321 | 0.003101 | C3/APOE/C5AR1/ITGAM/ITGB2/MARCO/MSR1/TREM2 | 8 |
| BP | GO:0045621 | positive regulation of lymphocyte differentiation | 14/941 | 101/18862 | 0.000481 | 0.004339 | 0.003114 | AXL/BCL6/GLI3/HLX/GLI2/MMP14/TNFSF4/ADAM8/CD86/FOXP3/LILRB2/LILRB4/NLRP3/PIK3R6 | 14 |
| BP | GO:0043535 | regulation of blood vessel endothelial cell migration | 18/941 | 150/18862 | 0.000497 | 0.004462 | 0.003203 | AKT3/ANGPT1/ATP2B4/FGFR1/MAP3K3/MEF2C/MEOX2/PRKD1/RHOJ/VEGFC/KDR/MMRN2/PDGFB/TBXA2R/THBS1/VASH1/APOE/TGFB1 | 18 |
| BP | GO:0050777 | negative regulation of immune response | 18/941 | 150/18862 | 0.000497 | 0.004462 | 0.003203 | A2M/BCL6/HLX/SERPING1/COL3A1/LOXL3/TNFSF4/CD300A/CD84/FCGR2B/FOXP3/HAVCR2/LILRB1/LILRB4/SAMHD1/SAMSN1/SLAMF8/VSIG4 | 18 |
| BP | GO:0006026 | aminoglycan catabolic process | 11/941 | 68/18862 | 0.000506 | 0.004528 | 0.00325 | CSPG4/DCN/GPC6/HSPG2/OMD/PRELP/SDC2/BGN/LUM/VCAN/TGFB1 | 11 |
| BP | GO:0014033 | neural crest cell differentiation | 13/941 | 90/18862 | 0.000507 | 0.004528 | 0.00325 | ANXA6/CDH2/HTR2B/MEF2C/NRP1/NRP2/SNAI2/SOX11/EDNRA/FN1/SEMA6B/SEMA7A/TWIST1 | 13 |
| BP | GO:0061326 | renal tubule development | 13/941 | 90/18862 | 0.000507 | 0.004528 | 0.00325 | ADAMTS16/FAT4/FGF1/GLI3/GREM1/MEF2C/NOTCH2/PKD2/WWTR1/COL4A1/DCHS1/HEYL/KIF26B | 13 |
[truncated: 141,172 more chars]
