# Supplementary figures and images for: Identification of a novel lymphangiogenesis signature associated with immune cell infiltration in colorectal cancer based on bioinformatics analysis
Source: BMC Med Genomics. 2024 Jan 2;17:2. doi: 10.1186/s12920-023-01781-8 (PMC10763205; doi:10.1186/s12920-023-01781-8)

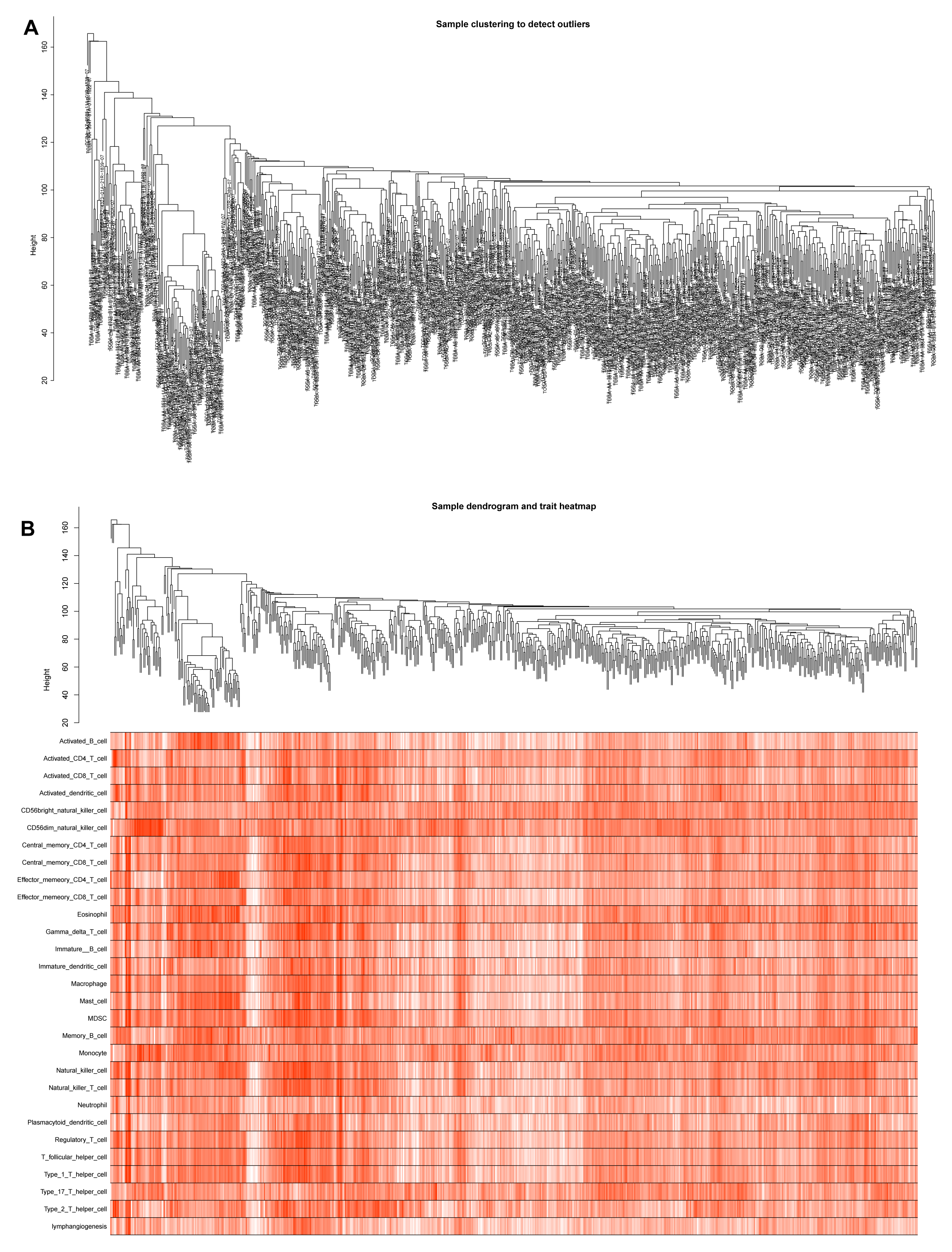

Supplement: Supplementary file 2 — Supplementary Material 2: Figure S1. Construction of a WGCNA. A. Sample clustering of genes from the TCGA database to identify outliers. B. Clustering dendrogram of CRC samples and associated clinical traits that included Lymphangiogenesis score (LymScore) and immune cell infiltration levels [file 12920_2023_1781_MOESM2_ESM.png]

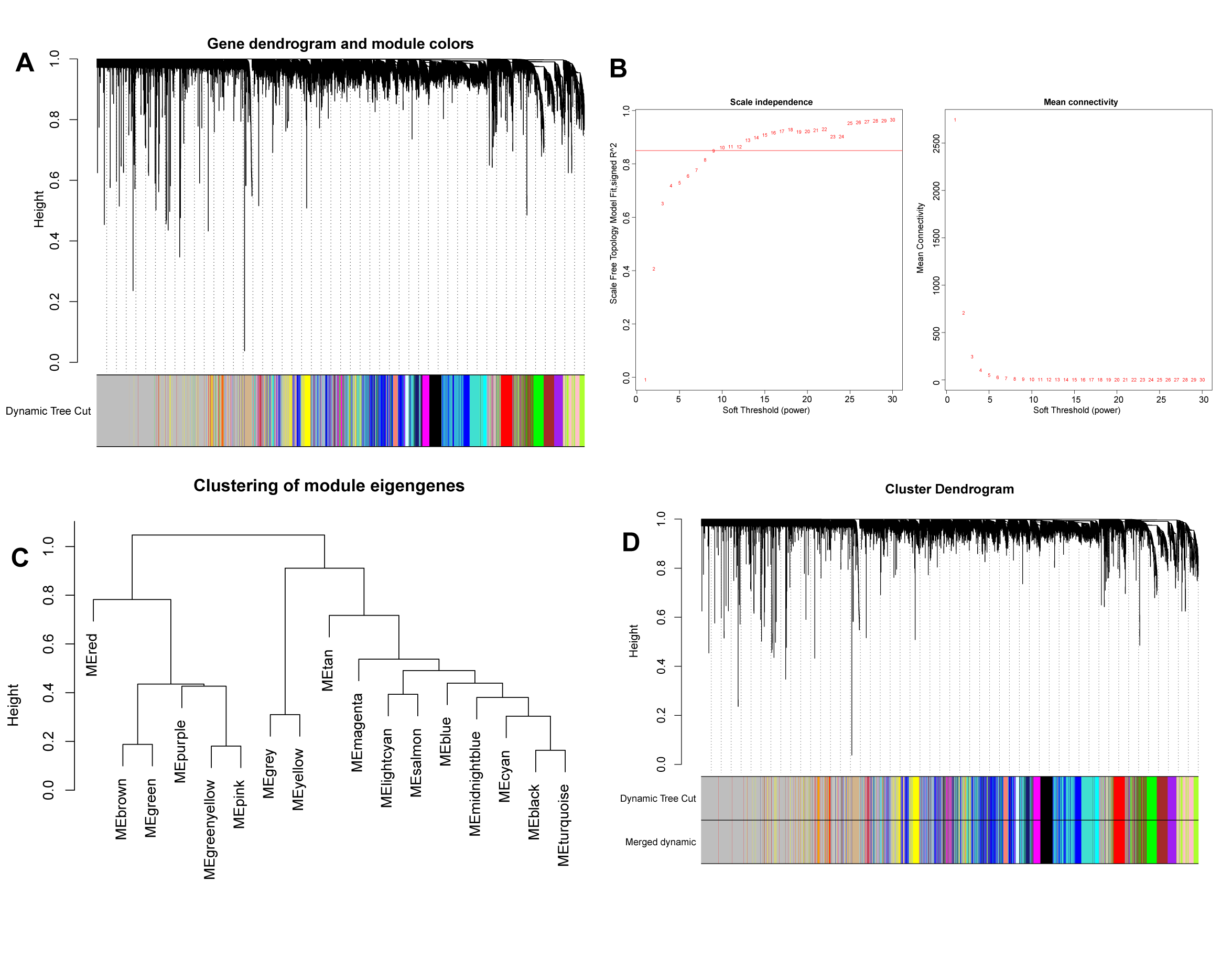

Supplement: Supplementary file 3 — Supplementary Material 3: Figure S2. Construction of a co-expression network of Lymscore and immune cell infiltration. A. Clustering dendrograms of genes based on the topological overlap and together with assigned module colors. B. The scale-free fit index for soft-thresholding powers. C. Clustering dendrograms of module eigengenes based on the topological overlap. D. Hierarchical clustering dendrograms of genes based on optimal soft-thresholding power [file 12920_2023_1781_MOESM3_ESM.png]
